# Potassium Hydroxide as a Cost-Effective Catalyst for Broad-Scope Silylation with TMSCF_3_

**DOI:** 10.1021/acs.joc.5c01625

**Published:** 2025-09-10

**Authors:** Martyna Markwitz, Kacper Łyczek, Fabio Bellina, Antonio Del Vecchio, Krzysztof Kuciński

**Affiliations:** † Faculty of Chemistry, Adam Mickiewicz University, Poznań, Uniwersytetu Poznańskiego St. 8, 61-614 Poznań, Poland; https://www.kucinskilab.com; ‡ Dipartimento di Chimica e Chimica Industriale, 154932 Università di Pisa, Via Moruzzi 13, 56124 Pisa, Italy

## Abstract

The development of efficient and broadly applicable silylation methodologies remains a central goal in synthetic organic and organosilicon chemistry. Traditionally, silylation reactions employ chlorosilanes or hydrosilanes, often necessitating the use of moisture-sensitive and corrosive reagents. Herein, we report a high-yielding, operationally simple, rapid, and economical silylation platform based on trifluoromethyltrimethylsilane (TMSCF_3_) and catalytic potassium hydroxide (KOH). This reaction system enables access to a broad array of substrates—including terminal alkynes and alcohols—exhibiting high functional group tolerance and facilitating the efficient silylation of pharmaceutically relevant molecules.

## Graphical abstract

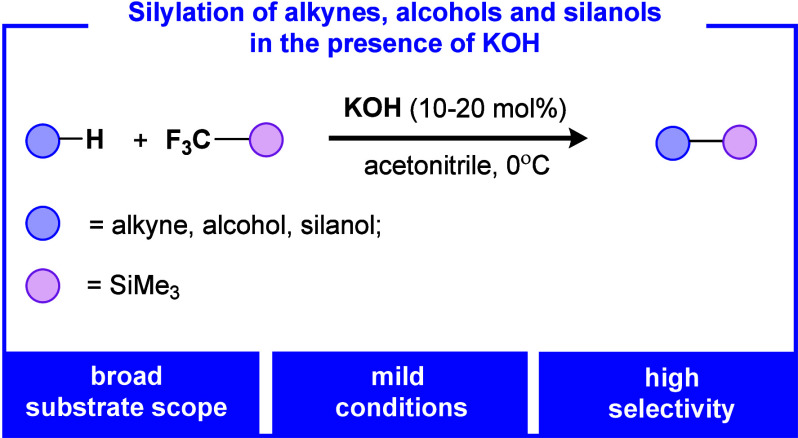

The development of efficient, selective, and broadly applicable silylation methodologies continues to represent an important objective in synthetic organic and organosilicon chemistry. Trimethylsilyl (TMS) group, in particular, is invaluable as protective and functional group across a wide spectrum of chemical transformations (Scheme 1, part a).^1,2^ With increasing emphasis on sustainability and operational simplicity, there is a strong drive within the chemistry community to create silylation protocols that are not only effective but also inexpensive, and compatible with standard laboratory equipment.^3,4^ Traditionally, silylations have been performed using chlorosilanes in the presence of strong bases or amines (Scheme 1, part b1);^5,6^ however, these reagents are often moisture-sensitive and corrosive. Hydrosilanes offer an alternative approach.^7–9^ While hydrosilylation is a versatile method for synthesizing various important organosilicon compounds,^10–13^ the scope of trimethylsilylation is significantly limited by the physicochemical properties of trimethylsilane (Me_3_SiH). This compound is a low-boiling (<7 °C), highly volatile, and flammable gas, which poses serious challenges in terms of safe handling, precise dosing, and scalability – particularly under standard laboratory conditions. Moreover, its low reactivity under neutral or mild catalytic conditions further restricts its utility in practical synthetic applications (Scheme 1, part b2). More recently, carbosilanes (e.g., alkynylsilanes,^14,15^ silacyclobutanes,^16^ *etc*.), silyl esters^17,18^ and others^19–22^ have been employed as silylating agents, though their application typically requires activation steps.

**Scheme 1:**
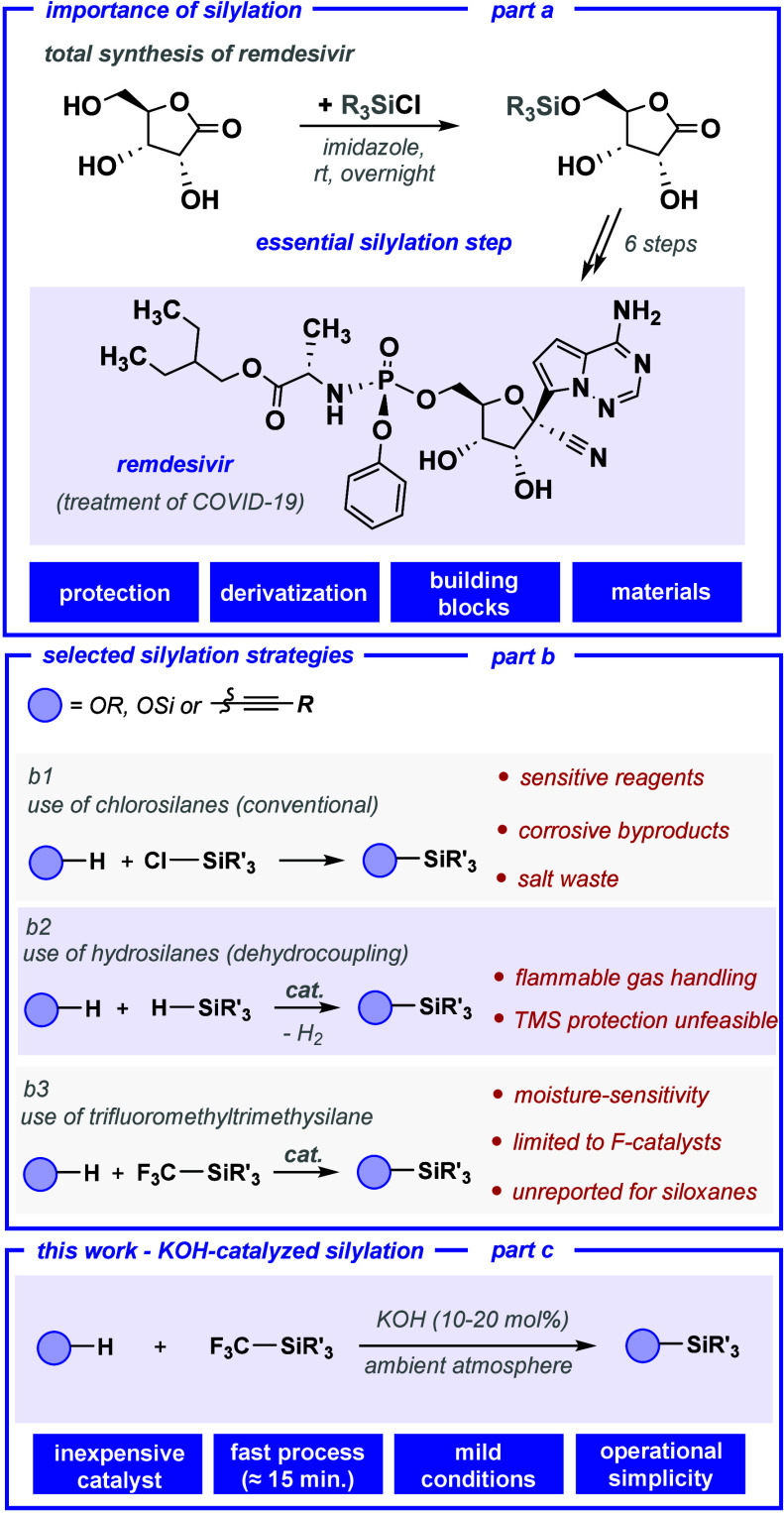
Context of the Investigation

Trifluoromethyltrimethylsilane (TMSCF_3_, also known as the Ruppert–Prakash reagent) is best known for its role in trifluoromethylation chemistry,^23,24^ but its potential as a silylating agent remains comparatively underexplored.^4^ While earlier studies have shown that TMSCF_3_ can silylate alkynes, these transformations typically rely on fluoride-based catalysts such as tetrabutylammonium fluoride (TBAF)^25^ or cesium fluoride (CsF).^26^ Despite their effectiveness, these systems are hampered by practical limitations including the need for rigorously dry conditions due to moisture sensitivity. Other approaches, such as the use of sodium hydride combined with *N*-heterocyclic carbenes (NaH/NHC),^27^ introduce further complexity by requiring glovebox techniques and handling of air-sensitive species. Similarly, the use of high-boiling solvents like *N,N’*-dimethylpropyleneurea (DMPU),^28^ while sometimes effective, poses concerns due to toxicity and environmental impact, in addition to complicating product isolation and purification. When it comes to the silylation of alcohols with TMSCF_3_, the previous work is limited to one example.^25^ Remarkably, there are no reported strategies for the direct silylation of silanols with TMSCF_3_, representing a clear gap in the scope of this reagent’s synthetic applications (Scheme 1, part b3).

In this context, the present study introduces a novel silylation strategy employing potassium hydroxide (KOH) as an efficient, readily available, and cost-effective catalyst in combination with TMSCF_3_ (Scheme 1, part c). This method enables the direct trimethylsilylation of a broad array of substrates – including alkynes, alcohols, and, for the first time, silanols – under operationally simple conditions, without the need for glovebox techniques or rigorously dry environments. The use of KOH, a widely used base, not only simplifies the procedure but also eliminates the reliance on fluoride salts or other additives. This catalytic system offers a cleaner and scalable alternative to existing silylation methods, while still harnessing the reactivity of trifluoromethyltrimethylsilane. Overall, this work significantly broadens the utility of TMSCF_3_ and establishes a new, unified platform for a practical and versatile silylation.

## Results and Discussion

Initial studies on the use of TMSCF_3_ as a silylating reagent focused on potassium (or sodium) bis­(trimethylsilyl)­amide and potassium *tert*-amylate as catalysts (Table 1, entries 1–3). However, potassium hydroxide proved to be an effective alternative due to its low cost, wide availability, and lower sensitivity to conditions. The latter was handled under ambient conditions with no special treatment other than grinding in a mortar beforehand. Table 1 summarizes the optimized conditions for the reaction of phenylacetylene (**1a**) with trifluoromethyltrimethylsilane (**2a**) using different bases and solvents. All experiments were carried out with new glassware and stir bars to avoid contamination.^29,30^

**Table 1:**
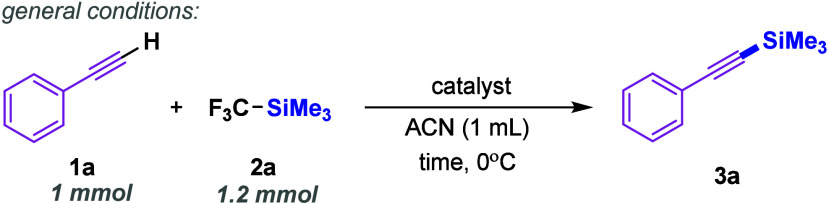
Optimization Studies for KOH-Catalyzed Silylation of Alkynes with Trifluoromethyltrimethylsilane^a^

entry	variation of conditions^b^	time [min.]	conversion of **1a** [isolated yield,%]^b^
1	5 mol % KHMDS^c^	15	96
2	5 mol % NaHMDS^c^	15	85
3	5 mol % KO^t^Am^d^	15	98
**4**	**10**mol %**KOH**	**15**	**99 [96]**
5	5 mol % KOH	120	93
6	10 mol % NaOH	15	97
7	10 mol % LiOH	15	12
8	10 mol % KF	15	98 [93]
9	neat	60	96 [89]
10	in toluene	60	56
11	in dioxane	15	99
12	in 2-MeTHF	60	95
13	in CH_2_Cl_2_	60	52
14	under argon atmosphere	30	98
15	no catalyst	120	0

a General reaction conditions: **1a** (1.0 equiv., 1 mmol, 0.102 g), **2a** (1.2 equiv., 1.2 mmol, 0.17 g), KOH (0.1 equiv., 0.1 mmol, 0.0056 g), acetonitrile (ACN, 1 mL), under air atmosphere, 0 °C, 15 min.

b Conversion determined by GC with *n*-dodecane as the internal standard.

c Used as 1 M solution in tetrahydrofuran.

d Used as 0.9 M solution in cyclohexane.

Optimal reaction conditions were established using 10 mol % potassium hydroxide at 0 °C (in acetonitrile – ACN), affording product **3a** in an excellent 96% isolated yield after just 15 min (Table 1, entry 4). Reducing the amount of KOH to 5 mol % resulted in a slightly lower, yet still high conversion of 93% after 4 h (Table 1, entry 5). Sodium hydroxide exhibits similarly high activity, yielding a slightly lower result (Table 1, entry 6). Lithium hydroxide, being a weaker base, exhibits significantly lower efficiency, with only 12% conversion of **1a** observed after 15 min (Table 1, entry 7).^8^ Although the use of alkali fluorides has been previously reported in the literature, potassium fluoride was also tested and confirmed to exhibit high catalytic activity (Table 1, entry 8).^26^ However, considering the hazards, it is definitely a less attractive option. Finally, the reaction also proceeded efficiently under neat conditions, as well as in solvents like dioxane and 2-methyltetrahydrofuran (2-MeTHF). In contrast, the use of toluene or dichloromethane led to reduced conversions (Table 1, entries 10 and 13). Additionally, the transformation was shown to be compatible under argon atmosphere (Table 1, entry 14). Such flexibility underscores the method’s potential for broader applicability in various experimental setups.

With the optimized conditions in hand, we next investigated the substrate scope of the silylation of terminal alkynes (Scheme 2, part a). First, product **3a** was also successfully obtained on a 10-times larger scale, resulting in 1.69 g of product with a yield of approximately 97%. We also investigated the possibility of using another commercially available substrate, namely trifluoromethyltriethylsilane **2a’**. In this case as well, the process proceeded with high efficiency, enabling the protection of the terminal alkyne with a triethylsilyl group **3a’**.

**Scheme 2:**
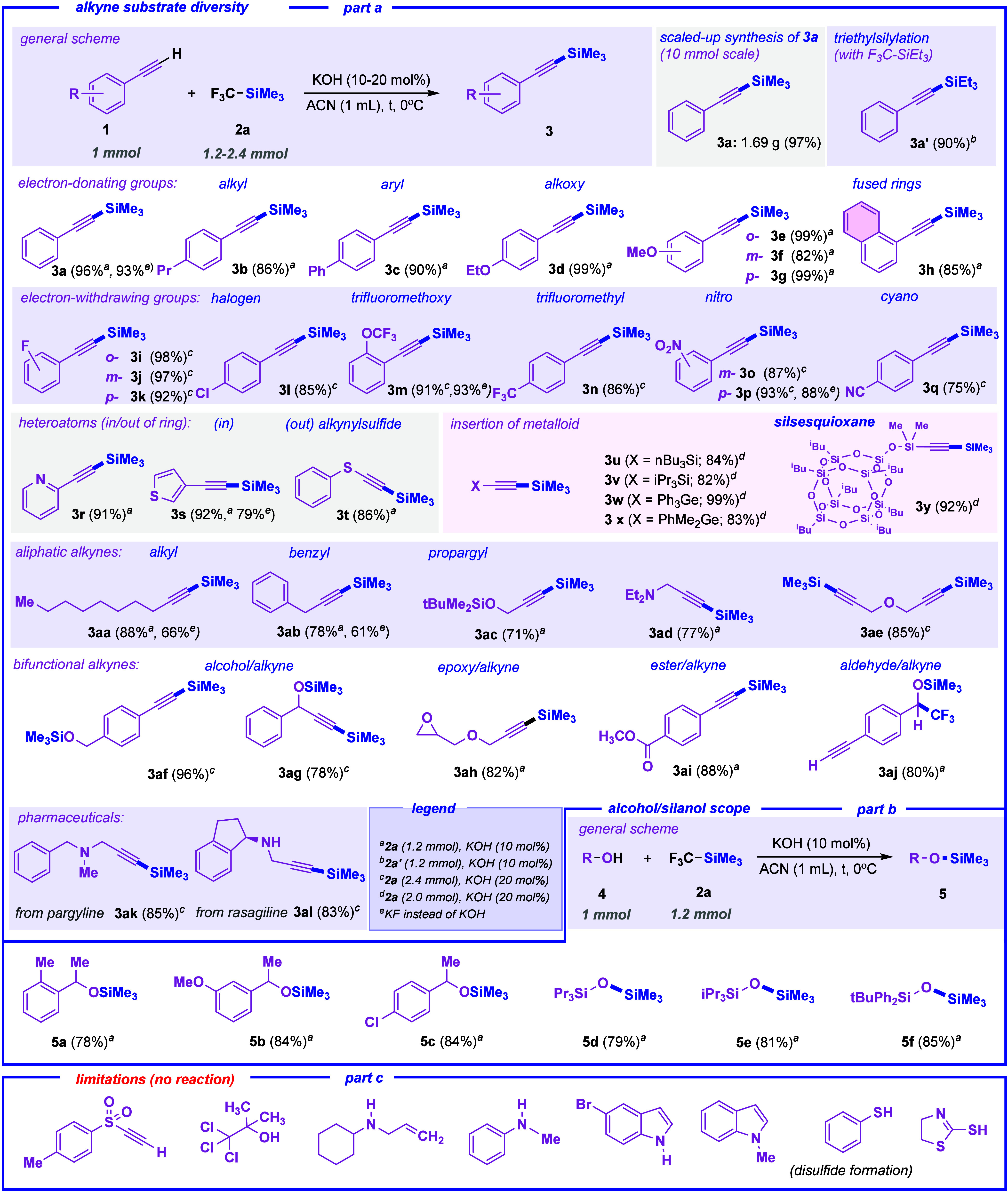
Substrate Scope for Silylation of Alkynes, Alcohols, and Silanols

Subsequently, we examined a range of phenylacetylene derivatives bearing electron-donating substituents. All tested substrates were efficiently converted into their corresponding trimethylsilylated derivatives (products **3b**–**3h**). The influence of substituent position on the aryl ring was also investigated, and no significant effect on the reaction outcome was observed (products **3e**–**3g**). We then extended the study to derivatives of **1a** containing electron-withdrawing groups, including trifluoromethyl, trifluoromethoxy, cyano, nitro, and halogen substituents. In these cases, an increased amount of trifluoromethyltrimethylsilane (2.4 equiv) was required to achieve complete conversion of the alkyne. Notably, the position of the fluorine atom (*ortho*-, *meta*-, or *para*-) did not significantly affect the reactivity, with comparable results observed across all cases. Importantly, in our previous studies using bis­(trimethylsilyl)­acetylene,^14^ we observed no reaction for derivatives bearing a nitro group (NO_2_). Moreover, substitution of the acetylenic proton in nitrophenylacetylene has proven challenging, with only a few successful examples reported in the literature. These include a single case employing hydrosilane with a gold-based catalyst,^31^ one using NaHMDS,^32^ and one utilizing the conventional sequence of deprotonation with organolithium reagents followed by reaction with a chlorosilane.^33^ The application of trifluoromethyltrimethylsilane effectively overcomes this limitation, allowing both *m*- and *p*-nitrophenylacetylene derivatives to be smoothly converted into desired products with high yields (**3o** and **3p**, 87% and 93%, respectively). This additional evidence demonstrates the high robustness of this reaction manifold.

Subsequently, we explored alkynes containing heteroatoms, both within and outside the aromatic ring. In each case, the desired products were obtained in high yields (86–92%), including derivatives of pyridine (**3r**), thiophene (**3s**), and alkynylsulfide (**3t**). Building on these findings, we next investigated the influence of metalloids positioned in close proximity to the carbon–carbon triple bond. A range of alkynylsilanes and alkynylgermanes were efficiently transformed into the corresponding products **3u**-**3x** in very good yields (82–99%). Notably, the methodology also proved applicable to the derivatization of silsesquioxanes (**3y**) – compounds of significant importance in materials chemistry.^34,35^ Despite the presence of strong base, no evidence of decomposition of the organosilicon cage was observed.^36^

We next examined aliphatic alkynes, which also exhibited high reactivity. Both a linear alkyne and a benzyl-substituted alkyne afforded the desired products **3aa** and **3ab** in 88% and 78% yield, respectively. Moreover, synthetically valuable propargyl derivatives were well tolerated, affording silylated products **3ac**–**3ae** in good yields (71–85%). Encouraged by these results, we then investigated the use of bifunctional acetylenes. Substrates with a hydroxyl group and alkyne underwent dual substitution, yielding **3af** and **3ag**. It is worth noting that in these cases, 2.4 equiv of TMSCF_3_ were required. Notably, glycidyl propargyl ether – a substrate of particular importance in polymer chemistry^37^ and a precursor to poly­(carbonate)­s – underwent silylation without affecting the epoxide group (**3ah**, 82%). Interestingly, methyl 4-ethynylbenzoate was also silylated (**3ai**, 88%) and any traces of ester hydrolysis were observed. On the other hand, when we used equimolar amount of 4-ethynylbenzaldehyde and TMSCF_3_ – a selective addition to carbonyl group took place instead the silylation of acetylenic position (**3aj**).^38,39^ Finally, our strategy also enabled the late-stage functionalization of biorelevant compounds such as pargyline^40^ and rasagiline^41^ (**3ak** and **3al**). The excellent outcomes observed for the tested substrates clearly demonstrated the elevated functional group tolerance of the protocol. The transformation allowed for the preparation of a large cohort of challenging products bearing EW- and EDGs, as well as sterically hindered motifs.

Building on the results obtained for alkynes bearing hydroxyl functionalities, we next explored a selection of readily available alcohols. This investigation was particularly relevant, as, to the best of our knowledge, TMSCF_3_ has never been directly employed for the synthesis of silyl ethers.^25^ Notably, as illustrated in Scheme 2 (part b), all tested alcohols, featuring both electron-donating and electron-withdrawing substituents, underwent efficient silylation, affording the corresponding alkoxysilanes **5a**-**5c** in good yields (78–84%). Due to the similarities between alcohols and silanols, subsequent studies focused on the reactivity of the latter, enabling the synthesis of unsymmetrical siloxanes, which are typically challenging to access using conventional methods.^3^ To our knowledge, the use of trifluoromethyltrimethylsilane for this transformation is unprecedented. In all cases, regardless of steric demand, the desired products **5d**-**5f** were obtained in high yields (79–85%).

Aiming to explore the potential of the reaction manifold, we investigated the silylation of a selected series of compounds containing alkynylsulfones, aliphatic and aromatic amines, as well as thiols. This helped us to identify the limitations of our method. The results are summarized in Scheme 2, part c. Among the tested alkynes and alcohols, ethynyl *p*-tolyl sulfone and 1,1,1-trichloro-2-methylpropan-2-ol showed complete lack of reactivity. We next investigated whether amines could undergo silylation to access highly challenging and sensitive aminosilanes.^42^ Unfortunately, no reaction products were observed for any of the substrates tested. This prompted us to evaluate the reactivity of indoles, considering both N–H silylation and functionalization at other positions.^27,43,44^ Again, only unreacted starting materials were detected. Lastly, we examined the reactivity of thiols. In the case of benzenethiol, we exclusively observed formation of the corresponding symmetrical disulfide, consistent with literature reports.^45^ For the thiazole derivative, the starting material remained unreacted.

We then carried out a series of experiments to gain further insights into the reaction mechanism. To evaluate the possible involvement of radical intermediates, the standard reaction was conducted in the presence of two radical scavengers: 2,2,6,6-tetramethylpiperidine-1-oxyl (TEMPO) and galvinoxyl (Scheme 3, part a).^46^

**Scheme 3:**
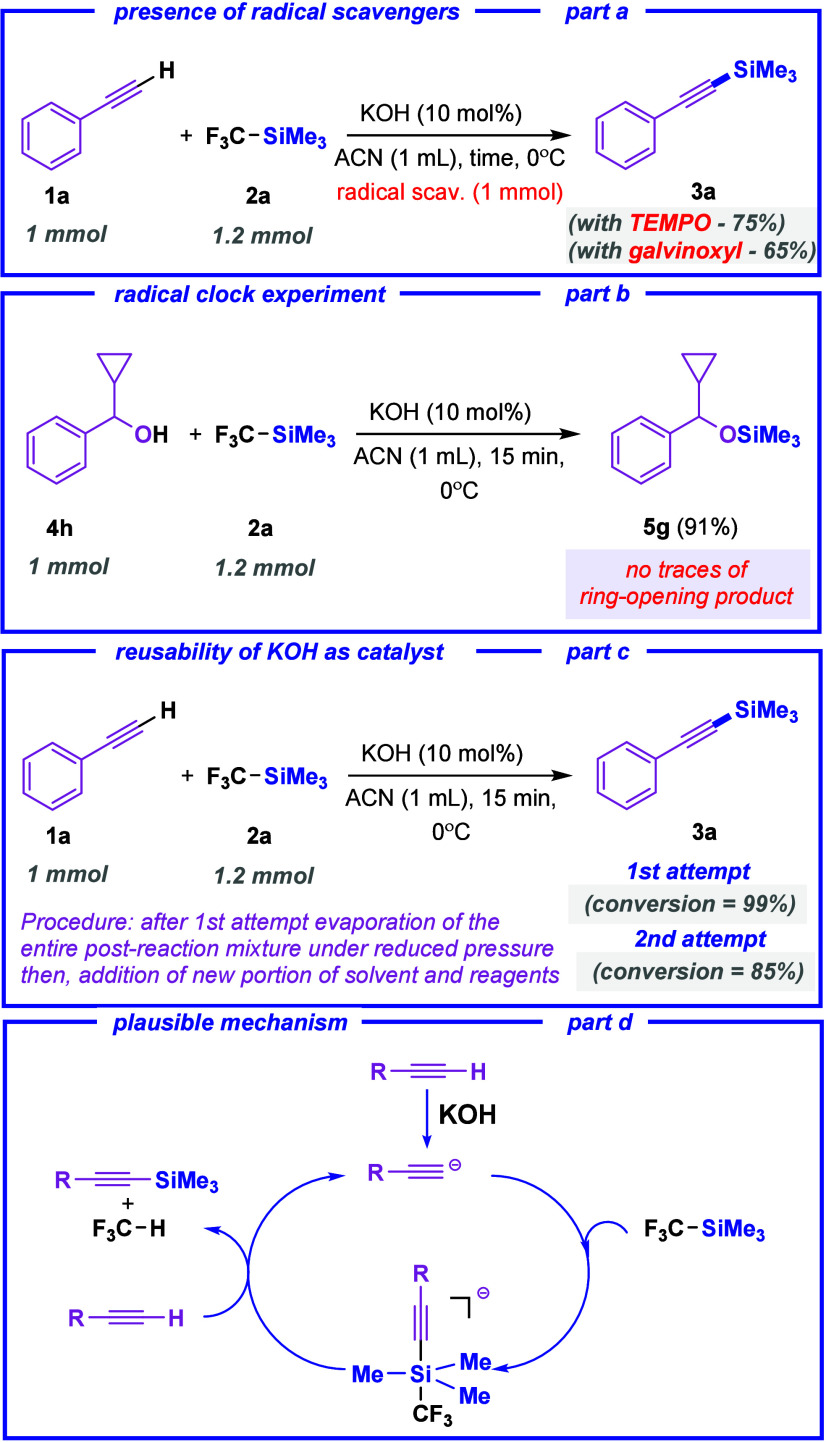
Preliminary Mechanistic Studies

When phenylacetylene was subjected to the optimized conditions with 1.0 equiv of each scavenger, the formation of 1-phenyl-2-trimethylsilylacetylene was only moderately suppressed, giving the desired product in 75% yield with TEMPO and 65% yield with galvinoxyl. The scavengers may act primarily by promoting CF_3_ radical abstraction; consequently, we observed a decrease in yield due to the consumption of TMSCF_3_ in their presence.^47^ These findings suggest that the reaction likely proceeds *via* a nonradical pathway; however, to confirm this, further investigations were undertaken. A radical clock experiment was performed using α-cyclopropylbenzyl alcohol as the probe (Scheme 3, part b).^48,49^ No ring-opening product was detected, providing additional evidence against the involvement of a radical mechanism.

Despite the scarce solubility of KOH in ACN and the formation of a white deposit at the bottom of the vessel, the reaction procedeed with excellent proficiency. Given the heterogeneous nature of the reaction manifold, we therefore planned a recycling experiment consisting on the reuse of the deposited base for two consecutive reactions, upon appropriate filtration of the soluble components. Gratifyingly, after the second reaction cycle, the desired product **3a** was observed with 85% conversion. Thus, the system can be reused (Scheme 3, part c).

Accordingly, we propose a plausible mechanism for the trimethylsilylation of terminal alkynes, as illustrated in Scheme 3, part d.^26,43,44,50,51^ This mechanism is also applicable to alcohols and silanols. The process begins with the reaction of an alk-1-yne with KOH, forming an acetylide anion. The anion then reacts with TMSCF_3_ to yield a pentacoordinated silicate species^52–54^ containing the acetylide group. Subsequent addition of another alkyne molecule results in the formation of a 1-trimethylsilylalkyne and fluoroform.

## Conclusion

In summary, we have developed a highly efficient method for the synthesis of a wide range of silylated alkynes, silyl ethers, and siloxanes. This strategy offers several notable advantages, including the use of inexpensive and readily available potassium hydroxide as the catalyst, a straightforward synthetic and product isolation procedure, mild reaction conditions (conducted under ambient atmosphere), a short reaction time (typically around 15 min), and, most importantly, exceptional tolerance toward various functional groups.

## Safety Statement

Potassium hydroxide (KOH, CAS No. 1310-58-3) is a strong base. It should be treated carefully and manipulated under ventilated workstations, avoiding direct contact with skin and eyes (H314 and H318).

Trimethyl­(trifluoromethyl)­silane (TMSCF_3_, CAS No. 81290-20-2) is a highly flammable, moisture-sensitive reagent (H225) that should be stored at 2–8 °C and handled in a ventilated workspace. Its thermal degradation may generate HF, though no safety issues were observed under the applied reaction protocol.

The trimethylsilylation reaction is exothermic due to the reagent properties. Alkynes, slightly acidic from their *sp*-hybridized electrons, react with KOH to form a stabilized alkyne anion, releasing heat. Proper precautions are necessary to control this exothermicity and ensure safe handling.

## Supplementary Material



## Data Availability

The data underlying this study are available in the published article and its Supporting Information.

## Notes

Experimental section, characterization of the obtained products and their spectra, and additional references 55–71 (PDF)
