# Supplementary material for: Potassium Hydroxide as a Cost-Effective Catalyst for Broad-Scope Silylation with TMSCF3
Source: J Org Chem. 2025 Sep 10;90(37):13040–6. doi: 10.1021/acs.joc.5c01625 (PMC12455661; doi:10.1021/acs.joc.5c01625)
Supplement: Supplementary file 1 [file jo5c01625_si_001.pdf]

# Supporting Information

## Potassium Hydroxide as a Cost-Effective Catalyst for Broad-Scope Silylation with $TMSCF_3$

Martyna Markwitz,[a] Kacper Łyczek,[a] Fabio Bellina,[b] Antonio Del Vecchio*,[b] and Krzysztof Kuciński*,[a]

[a] Faculty of Chemistry, Adam Mickiewicz University, Poznań, Uniwersytetu Poznańskiego St. 8, 61-614 Poznań, Poland.

[b]Dipartimento di Chimica e Chimica Industriale, Università di Pisa, Via Moruzzi 13, 56124 Pisa, Italy.

*email: kucinski.k@amu.edu.pl, antonio.delvecchio@unipi.it;

Twitter account: @SiRCH_Kucina (KK), @Cactus0410 (ADV);

https://www.kucinskilab.com/

ORCID iDs:

0009-0005-3251-6858 (Martyna Markwitz, marmar80@st.amu.edu.pl)

0009-0006-1549-499X (Kacper Łyczek, kaclyc@st.amu.edu.pl)

0000-0002-4939-7008 (Fabio Bellina, fabio.bellina@unipi.it)

0000-0001-9759-4420 (Antonio Del Vecchio, antonio.delvecchio@unipi.it)

0000-0002-9339-6395 (Krzysztof Kuciński, kucinski.k@amu.edu.pl)

## Content

GENERAL INFORMATION ........ S8

OPTIMIZATION OF REACTION CONDITIONS ........ S9

GENERAL SYNTHETIC PROCEDURES ........ S10

Synthesis of compounds **3a-3h** ........ S10

Synthesis of compound **3a’** ........ S10

Synthesis of compounds **3i-3q** ........ S10

Synthesis of compounds **3r-3t** ........ S10

Synthesis of compounds **3u-3x** ........ S10

Synthesis of compound **3y** ........ S11

Synthesis of compounds **3aa-3ad**, and **3ah-3aj** ........ S11

Synthesis of compounds **3ae-3ag** and **3ak-3am** ........ S11

Synthesis of compounds **5a-5g** ........ S11

Scaled-up synthesis of **3a** ........ S12

Experiments in the presence of radical scavengers ........ S12

Radical clock experiment ........ S12

CHARACTERIZATION DATA FOR ALL PRODUCTS ........ S13

Trimethyl(phenylethynyl)silane (**3a**) ........ S13

Trimethyl((4-propylphenyl)ethynyl)silane (**3b**) ........ S13

([1,1'-Biphenyl]-4-ylethynyl)trimethylsilane (**3c**) ........ S13

((4-Ethoxyphenyl)ethynyl)trimethylsilane (**3d**) ........ S13

((2-Methoxyphenyl)ethynyl)trimethylsilane (**3e**) ........ S14

((3-Methoxyphenyl)ethynyl)trimethylsilane (**3f**) ........ S14

((4-Methoxyphenyl)ethynyl)trimethylsilane (**3g**) ........ S14

Trimethyl(naphthalen-1-ylethynyl)silane (**3h**) ........ S15

((2-Fluorophenyl)ethynyl)trimethylsilane (**3i**) ........ S15

((3-Fluorophenyl)ethynyl)trimethylsilane (**3j**) ........ S15

((4-Fluorophenyl)ethynyl)trimethylsilane (**3k**) ........ S15

((4-Chlorophenyl)ethynyl)trimethylsilane (**3l**) ........ S16

Trimethyl((2-(trifluoromethoxy)phenyl)ethynyl)silane (**3m**) ........ S16

Trimethyl((4-(trifluoromethyl)phenyl)ethynyl)silane (**3n**) ........ S16

Trimethyl((3-nitrophenyl)ethynyl)silane (**3o**) ........ S17

Trimethyl((4-nitrophenyl)ethynyl)silane (**3p**) ........ S17

4-((Trimethylsilyl)ethynyl)benzonitrile (**3q**) ........ S17

2-((Trimethylsilyl)ethynyl)pyridine (**3r**) ........ S17

Trimethyl(thiophen-3-ylethynyl)silane (**3s**) ........ S18

Trimethyl((phenylthio)ethynyl)silane (**3t**) ........ S18

Tributyl((trimethylsilyl)ethynyl)silane (**3u**) ........ S18

Trimethyl((triisopropylsilyl)ethynyl)silane (**3v**) ........ S18

Trimethyl((triphenylgermyl)ethynyl)silane (**3w**)....S19
(Dimethyl(phenyl)germyl)ethynyl)trimethylsilane (**3x**)....S19
1-((Dimethyl((trimethylsilyl)ethynyl)silyl)oxy)-3,5,7,9,11,13,15-heptaisobutyl-2,4,6,8,10,12,14,16,17,18,19,20-dodecaoxa-1,3,5,7,9,11,13,15-octasilapentacyclo[9.5.1.13,9.15,15.17,13]icosane (**3y**)....S19
Triethyl(phenylethynyl)silane (**3a'**)....S19
Dec-1-yn-1-yltrimethylsilane (**3aa**)....S20
Trimethyl(3-phenylprop-1-yn-1-yl)silane (**3ab**)....S20
*Tert*-butyldimethyl((3-(trimethylsilyl)prop-2-yn-1-yl)oxy)silane (**3ac**)....S20
*N,N*-diethyl-3-(trimethylsilyl)prop-2-yn-1-amine (**3ad**)....S20
(Oxybis(prop-1-yne-3,1-diyl))bis(trimethylsilane) (**3ae**)....S21
Trimethyl((4-((trimethylsilyl)ethynyl)benzyl)oxy)silane (**3af**)....S21
Trimethyl(3-phenyl-3-((trimethylsilyl)oxy)prop-1-yn-1-yl)silane (**3ag**)....S21
Trimethyl(3-(oxiran-2-ylmethoxy)prop-1-yn-1-yl)silane (**3ah**)....S21
Methyl 4-((trimethylsilyl)ethynyl)benzoate (**3ai**)....S22
(1-(4-Ethynylphenyl)-2,2,2-trifluoroethoxy)trimethylsilane (**3aj**)....S22
*N*-benzyl-N-methyl-3-(trimethylsilyl)prop-2-yn-1-amine (**3ak**)....S22
*(R)-N*-(3-(trimethylsilyl)prop-2-yn-1-yl)-2,3-dihydro-1H-inden-1-amine (**3al**)....S22
Trimethyl(1-(*o*-tolyl)ethoxy)silane (**5a**)....S23
(1-(3-Methoxyphenyl)ethoxy)trimethylsilane (**5b**)....S23
(1-(4-Chlorophenyl)ethoxy)trimethylsilane (**5c**)....S23
1,1,1-Trimethyl-3,3,3-tripropyldisiloxane (**5d**)....S23
1,1,1-Trimethyl-3,3,3-triisopropyldisiloxane (**5e**)....S24
1-(*Tert*-butyl)-3,3,3-trimethyl-1,1-diphenyldisiloxane (**5f**)....S24
(Cyclopropyl(phenyl)methoxy)trimethylsilane (**5g**)....S24
SPECTRA FOR ALL PRODUCTS....S25
Trimethyl(phenylethynyl)silane (**3a**)....S26
Figure S1. $^1$H NMR (400 MHz, Chloroform-d, 25°C) of trimethyl(phenylethynyl)silane (**3a**)....S26
Figure S2. $^{13}$C{$^1$H} NMR (101 MHz, Chloroform-d, 25°C) of trimethyl(phenylethynyl)silane (**3a**)....S26
Figure S3. $^{29}$Si NMR (79 MHz, Chloroform-d, 25°C) of trimethyl(phenylethynyl)silane (**3a**)....S27
Trimethyl((4-propylphenyl)ethynyl)silane (**3b**)....S28
Figure S4. $^1$H NMR (400 MHz, Chloroform-d, 25°C) of trimethyl((4-propylphenyl)ethynyl)silane (**3b**)....S28
Figure S5. $^{13}$C{$^1$H} NMR (101 MHz, Chloroform-d, 25°C) of trimethyl((4-propylphenyl)ethynyl)silane (**3b**)....S28
Figure S6. $^{29}$Si NMR (79 MHz, Chloroform-d, 25°C) of trimethyl((4-propylphenyl)ethynyl)silane (**3b**)....S29
([1,1'-Biphenyl]-4-ylethynyl)trimethylsilane (**3c**)....S30
Figure S7. $^1$H NMR (400 MHz, Chloroform-d, 25°C) of ([1,1'-biphenyl]-4-ylethynyl)trimethylsilane (**3c**)....S30
Figure S8. $^{13}$C{$^1$H} NMR (101 MHz, Chloroform-d, 25°C) of ([1,1'-biphenyl]-4-ylethynyl)trimethylsilane (**3c**)....S30
Figure S9. $^{29}$Si NMR (79 MHz, Chloroform-d, 25°C) of ([1,1'-biphenyl]-4-ylethynyl)trimethylsilane (**3c**)....S31
((4-Ethoxyphenyl)ethynyl)trimethylsilane (**3d**)....S32
Figure S10. $^1$H NMR (400 MHz, Chloroform-d, 25°C) of ((4-ethoxyphenyl)ethynyl)trimethylsilane (**3d**)....S32
Figure S11. $^{13}$C{$^1$H} NMR (101 MHz, Chloroform-d, 25°C) of ((4-ethoxyphenyl)ethynyl)trimethylsilane (**3d**)....S32
Figure S12. $^{29}$Si NMR (79 MHz, Chloroform-d, 25°C) of ((4-ethoxyphenyl)ethynyl)trimethylsilane (**3d**)....S33

((2-Methoxyphenyl)ethynyl)trimethylsilane (**3e**)....S34
Figure S13. $^{1}H$ NMR (400 MHz, Chloroform-d, 25°C) of ((2-methoxyphenyl)ethynyl)trimethylsilane (**3e**). ....S34
Figure S14. $^{13}C\{^{1}H\}$ NMR (101 MHz, Chloroform-d, 25°C) of ((2-methoxyphenyl)ethynyl)trimethylsilane (**3e**). ....S34
Figure S15. $^{29}Si$ NMR (79 MHz, Chloroform-d, 25°C) of ((2-methoxyphenyl)ethynyl)trimethylsilane (**3e**). ....S35
((3-Methoxyphenyl)ethynyl)trimethylsilane (**3f**) ....S36
Figure S16. $^{1}H$ NMR (400 MHz, Chloroform-d, 25°C) of ((3-methoxyphenyl)ethynyl)trimethylsilane (**3f**)....S36
Figure S17. $^{13}C\{^{1}H\}$ NMR (101 MHz, Chloroform-d, 25°C) of ((3-methoxyphenyl)ethynyl)trimethylsilane (**3f**) ....S36
Figure S18. $^{29}Si$ NMR (79 MHz, Chloroform-d, 25°C) of ((3-methoxyphenyl)ethynyl)trimethylsilane (**3f**)....S37
((4-Methoxyphenyl)ethynyl)trimethylsilane (**3g**) ....S38
Figure S19. $^{1}H$ NMR (400 MHz, Chloroform-d, 25°C) of ((4-methoxyphenyl)ethynyl)trimethylsilane (**3g**) ....S38
Figure S20. $^{13}C\{^{1}H\}$ NMR (101 MHz, Chloroform-d, 25°C) of ((4-methoxyphenyl)ethynyl)trimethylsilane (**3g**)....S38
Figure S21. $^{29}Si$ NMR (79 MHz, Chloroform-d, 25°C) of ((4-methoxyphenyl)ethynyl)trimethylsilane (**3g**). ....S39
Trimethyl(naphthalen-1-ylethynyl)silane (**3h**) ....S40
Figure S22. $^{1}H$ NMR (400 MHz, Chloroform-d, 25°C) of trimethyl(naphthalen-1-ylethynyl)silane (**3h**). ....S40
Figure S23. $^{13}C\{^{1}H\}$ NMR (101 MHz, Chloroform-d, 25°C) of trimethyl(naphthalen-1-ylethynyl)silane (**3h**). ....S40
Figure S24. $^{29}Si$ NMR (79 MHz, Chloroform-d, 25°C) of trimethyl(naphthalen-1-ylethynyl)silane (**3h**). ....S41
((2-Fluorophenyl)ethynyl)trimethylsilane (**3i**)....SS42
Figure S24. $^{1}H$ NMR (400 MHz, Chloroform-d, 25°C) of ((2-fluorophenyl)ethynyl)trimethylsilane (**3i**). ....S42
Figure S25. $^{13}C\{^{1}H\}$ NMR (101 MHz, Chloroform-d, 25°C) of ((2-fluorophenyl)ethynyl)trimethylsilane (**3i**). ....SS42
Figure S26. $^{19}F$ NMR (377 MHz, Chloroform-d, 25°C) of ((2-fluorophenyl)ethynyl)trimethylsilane (**3i**). ....S43
Figure S27. $^{29}Si$ NMR (79 MHz, Chloroform-d, 25°C) of ((2-fluorophenyl)ethynyl)trimethylsilane (**3i**). ....SS43
((3-Fluorophenyl)ethynyl)trimethylsilane (**3j**) ....S44
Figure S28. $^{1}H$ NMR (400 MHz, Chloroform-d, 25°C) of ((3-fluorophenyl)ethynyl)trimethylsilane (**3j**). ....S44
Figure S29. $^{13}C\{^{1}H\}$ NMR (101 MHz, Chloroform-d, 25°C) of ((3-fluorophenyl)ethynyl)trimethylsilane (**3j**). ....S44
Figure S30. $^{19}F$ NMR (377 MHz, Chloroform-d, 25°C) of ((3-fluorophenyl)ethynyl)trimethylsilane (**3j**). ....S45
Figure S31. $^{29}Si$ NMR (79 MHz, Chloroform-d, 25°C) of ((3-fluorophenyl)ethynyl)trimethylsilane (**3j**). ....S45
((4-Fluorophenyl)ethynyl)trimethylsilane (**3k**) ....S46
Figure S32. $^{1}H$ NMR (400 MHz, Chloroform-d, 25°C) of ((4-fluorophenyl)ethynyl)trimethylsilane (**3k**). ....S46
Figure S33. $^{13}C\{^{1}H\}$ NMR (101 MHz, Chloroform-d, 25°C) of ((4-fluorophenyl)ethynyl)trimethylsilane (**3k**). ....S46
Figure S34. $^{19}F$ NMR (377 MHz, Chloroform-d, 25°C) of ((4-fluorophenyl)ethynyl)trimethylsilane (**3k**). ....S47
Figure S35. $^{29}Si$ NMR (79 MHz, Chloroform-d, 25°C) of ((4-fluorophenyl)ethynyl)trimethylsilane (**3k**). ....S47
((4-Chlorophenyl)ethynyl)trimethylsilane (**3l**)....S48
Figure S36. $^{1}H$ NMR (400 MHz, Chloroform-d, 25°C) of ((4-chlorophenyl)ethynyl)trimethylsilane (**3l**). ....S48
Figure S37. $^{13}C\{^{1}H\}$ NMR (101 MHz, Chloroform-d, 25°C) of ((4-chlorophenyl)ethynyl)trimethylsilane (**3l**). ....S48
Figure S38. $^{29}Si$ NMR (79 MHz, Chloroform-d, 25°C) of ((4-chlorophenyl)ethynyl)trimethylsilane (**3l**). ....S49
Trimethyl((2-(trifluoromethoxy)phenyl)ethynyl)silane (**3m**) ....S50
Figure S39. $^{1}H$ NMR (400 MHz, Chloroform-d, 25°C) of trimethyl((2-(trifluoromethoxy)phenyl)ethynyl)silane (**3m**). ....S50
Figure S40. $^{13}C\{^{1}H\}$ NMR (101 MHz, Chloroform-d, 25°C) of trimethyl((2-(trifluoromethoxy)phenyl)ethynyl)silane (**3m**). ....S50
Figure S41. $^{19}F$ NMR (377 MHz, Chloroform-d, 25°C) of trimethyl((2-(trifluoromethoxy)phenyl)ethynyl)silane (**3m**). ....S51
Figure S42. $^{29}Si$ NMR (79 MHz, Chloroform-d, 25°C) of trimethyl((2-(trifluoromethoxy)phenyl)ethynyl)silane (**3m**). ....S51
Trimethyl((4-(trifluoromethyl)phenyl)ethynyl)silane (**3n**) ....S52
Figure S43. $^{1}H$ NMR (400 MHz, Chloroform-d, 25°C) of trimethyl((4-(trifluoromethyl)phenyl)ethynyl)silane (**3n**). ....S52
Figure S44. $^{13}C\{^{1}H\}$ NMR (101 MHz, Chloroform-d, 25°C) of trimethyl((4-(trifluoromethyl)phenyl)ethynyl)silane (**3n**). ....S52
Figure S45. $^{19}F$ NMR (377 MHz, Chloroform-d, 25°C) of trimethyl((4-(trifluoromethyl)phenyl)ethynyl)silane (**3n**). ....S53
Figure S46. $^{29}Si$ NMR (79 MHz, Chloroform-d, 25°C) of trimethyl((4-(trifluoromethyl)phenyl)ethynyl)silane (**3n**). ....S53

Trimethyl((3-nitrophenyl)ethynyl)silane (**3o**) ........ S54
Figure S47. $^1$H NMR (400 MHz, Chloroform-d, 25°C) of trimethyl((3-nitrophenyl)ethynyl)silane (**3o**). ........ S54
Figure S48. $^{13}$C{$^1$H} NMR (101 MHz, Chloroform-d, 25°C) of trimethyl((3-nitrophenyl)ethynyl)silane (**3o**). ........ S54
Figure S49. $^{29}$Si NMR (79 MHz, Chloroform-d, 25°C) of trimethyl((3-nitrophenyl)ethynyl)silane (**3o**). ........ S55
Trimethyl((4-nitrophenyl)ethynyl)silane (**3p**) ........ S56
Figure S50. $^1$H NMR (400 MHz, Chloroform-d, 25°C) of trimethyl((4-nitrophenyl)ethynyl)silane (**3p**). ........ S56
Figure S51. $^{13}$C{$^1$H} NMR (101 MHz, Chloroform-d, 25°C) of trimethyl((4-nitrophenyl)ethynyl)silane (**3p**). ........ S56
Figure S52. $^{29}$Si NMR (79 MHz, Chloroform-d, 25°C) of trimethyl((4-nitrophenyl)ethynyl)silane (**3p**). ........ S57
4-((Trimethylsilyl)ethynyl)benzonitrile (**3q**) ........ S58
Figure S53. $^1$H NMR (400 MHz, Chloroform-d, 25°C) of 4-((trimethylsilyl)ethynyl)benzonitrile (**3q**). ........ S58
Figure S54. $^{13}$C{$^1$H} NMR (101 MHz, Chloroform-d, 25°C) of 4-((trimethylsilyl)ethynyl)benzonitrile (**3q**). ........ S58
Figure S55. $^{29}$Si NMR (79 MHz, Chloroform-d, 25°C) of 4-((trimethylsilyl)ethynyl)benzonitrile (**3q**). ........ S59
2-((Trimethylsilyl)ethynyl)pyridine (**3r**) ........ S60
Figure S56. $^1$H NMR (400 MHz, Chloroform-d, 25°C) of 2-((trimethylsilyl)ethynyl)pyridine (**3r**). ........ S60
Figure S57. $^{13}$C{$^1$H} NMR (101 MHz, Chloroform-d, 25°C) of 2-((trimethylsilyl)ethynyl)pyridine (**3r**). ........ S60
Figure S58. $^{29}$Si NMR (79 MHz, Chloroform-d, 25°C) of 2-((trimethylsilyl)ethynyl)pyridine (**3r**). ........ S61
Trimethyl(thiophen-3-ylethynyl)silane (**3s**) ........ S62
Figure S59. $^1$H NMR (400 MHz, Chloroform-d, 25°C) of trimethyl(thiophen-3-ylethynyl)silane (**3s**). ........ S62
Figure S60. $^{13}$C{$^1$H} NMR (101 MHz, Chloroform-d, 25°C) of trimethyl(thiophen-3-ylethynyl)silane (**3s**). ........ S62
Figure S61. $^{29}$Si NMR (79 MHz, Chloroform-d, 25°C) of trimethyl(thiophen-3-ylethynyl)silane (**3s**). ........ S63
Trimethyl((phenylthio)ethynyl)silane (**3t**) ........ S64
Figure S62. $^1$H NMR (400 MHz, Chloroform-d, 25°C) of trimethyl((phenylthio)ethynyl)silane (**3t**). ........ S64
Figure S63. $^{13}$C{$^1$H} NMR (101 MHz, Chloroform-d, 25°C) of trimethyl((phenylthio)ethynyl)silane (**3t**). ........ S64
Figure S64. $^{29}$Si NMR (79 MHz, Chloroform-d, 25°C) of trimethyl((phenylthio)ethynyl)silane (**3t**). ........ S65
Tributyl((trimethylsilyl)ethynyl)silane (**3u**) ........ S66
Figure S65. $^1$H NMR (400 MHz, Chloroform-d, 25°C) of tributyl((trimethylsilyl)ethynyl)silane (**3u**). ........ S66
Figure S66. $^{13}$C{$^1$H} NMR (101 MHz, Chloroform-d, 25°C) of tributyl((trimethylsilyl)ethynyl)silane (**3u**). ........ S66
Figure S67. $^{29}$Si NMR (79 MHz, Chloroform-d, 25°C) of tributyl((trimethylsilyl)ethynyl)silane (**3u**). ........ S67
Trimethyl((triisopropylsilyl)ethynyl)silane (**3v**) ........ S68
Figure S68. $^1$H NMR (400 MHz, Chloroform-d, 25°C) of trimethyl((triisopropylsilyl)ethynyl)silane (**3v**). ........ S68
Figure S69. $^{13}$C{$^1$H} NMR (101 MHz, Chloroform-d, 25°C) of trimethyl((triisopropylsilyl)ethynyl)silane (**3v**). ........ S68
Figure S70. $^{29}$Si NMR (79 MHz, Chloroform-d, 25°C) of trimethyl((triisopropylsilyl)ethynyl)silane (**3v**). ........ S69
Trimethyl((triphenylgermyl)ethynyl)silane (**3w**) ........ S70
Figure S71. $^1$H NMR (400 MHz, Chloroform-d, 25°C) of trimethyl((triphenylgermyl)ethynyl)silane (**3w**). ........ S70
Figure S72. $^{13}$C{$^1$H} NMR (101 MHz, Chloroform-d, 25°C) of trimethyl((triphenylgermyl)ethynyl)silane (**3w**). ........ S70
Figure S73. $^{29}$Si NMR (79 MHz, Chloroform-d, 25°C) of trimethyl((triphenylgermyl)ethynyl)silane (**3w**). ........ S71
(Dimethyl(phenyl)germyl)ethynyl)trimethylsilane (**3x**) ........ S72
Figure S74. $^1$H NMR (400 MHz, Chloroform-d, 25°C) of (dimethyl(phenyl)germyl)ethynyl)trimethylsilane (**3x**). ........ S72
Figure S75. $^{13}$C{$^1$H} NMR (101 MHz, Chloroform-d, 25°C) of (dimethyl(phenyl)germyl)ethynyl)trimethylsilane (**3x**). ........ S72
Figure S76. $^{29}$Si NMR (79 MHz, Chloroform-d, 25°C) of (dimethyl(phenyl)germyl)ethynyl)trimethylsilane (**3x**). ........ S73
1-((Dimethyl((trimethylsilyl)ethynyl)silyl)oxy)-3,5,7,9,11,13,15-heptaisobutyl-2,4,6,8,10,12,14,16,17,18,19,20-dodecaoxa-1,3,5,7,9,11,13,15-octasilapentacyclo[9.5.1.13,9.15,15.17,13]icosane (**3y**) ........ S74
Figure S77. $^1$H NMR (400 MHz, Chloroform-d, 25°C) of **3y**. ........ S74
Figure S78. $^{13}$C{$^1$H} NMR (101 MHz, Chloroform-d, 25°C) of **3y**. ........ S74

Figure S79. $^{29}$Si NMR (79 MHz, Chloroform-d, 25°C) of **3y**. ..... S75

Triethyl(phenylethynyl)silane (**3a'**) ..... S76

Figure S80. $^{1}$H NMR (400 MHz, Chloroform-d, 25°C) of triethyl(phenylethynyl)silane (**3a'**). ..... S76

Figure S81. $^{13}$C{$^{1}$H} NMR (101 MHz, Chloroform-d, 25°C) of triethyl(phenylethynyl)silane (**3a'**). ..... S76

Figure S82. $^{29}$Si NMR (79 MHz, Chloroform-d, 25°C) of triethyl(phenylethynyl)silane (**3a'**). ..... S77

Dec-1-yn-1-yltrimethylsilane (**3aa**) ..... S78

Figure S83. $^{1}$H NMR (400 MHz, Chloroform-d, 25°C) of dec-1-yn-1-yltrimethylsilane (**3aa**). ..... S78

Figure S84. $^{13}$C{$^{1}$H} NMR (101 MHz, Chloroform-d, 25°C) of dec-1-yn-1-yltrimethylsilane (**3aa**). ..... S78

Figure S85. $^{29}$Si NMR (79 MHz, Chloroform-d, 25°C) of dec-1-yn-1-yltrimethylsilane (**3aa**). ..... S79

Trimethyl(3-phenylprop-1-yn-1-yl)silane (**3ab**) ..... S80

Figure S86. $^{1}$H NMR (400 MHz, Chloroform-d, 25°C) of trimethyl(3-phenylprop-1-yn-1-yl)silane (**3ab**). ..... S80

Figure S87. $^{13}$C{$^{1}$H} NMR (101 MHz, Chloroform-d, 25°C) of trimethyl(3-phenylprop-1-yn-1-yl)silane (**3ab**). ..... S80

Figure S88. $^{29}$Si NMR (79 MHz, Chloroform-d, 25°C) of trimethyl(3-phenylprop-1-yn-1-yl)silane (**3ab**). ..... S81

*Tert*-butyldimethyl((3-(trimethylsilyl)prop-2-yn-1-yl)oxy)silane (**3ac**) ..... S82

Figure S89. $^{1}$H NMR (400 MHz, Chloroform-d, 25°C) of *tert*-butyldimethyl((3-(trimethylsilyl)prop-2-yn-1-yl)oxy)silane (**3ac**). S82

Figure S90. $^{13}$C{$^{1}$H} NMR (101 MHz, Chloroform-d, 25°C) of *tert*-butyldimethyl((3-(trimethylsilyl)prop-2-yn-1-yl)oxy)silane (**3ac**). ..... S82

Figure S91. $^{29}$Si NMR (79 MHz, Chloroform-d, 25°C) of *tert*-butyldimethyl((3-(trimethylsilyl)prop-2-yn-1-yl)oxy)silane (**3ac**).. S83

*N,N*-diethyl-3-(trimethylsilyl)prop-2-yn-1-amine (**3ad**) ..... S84

Figure S92. $^{1}$H NMR (400 MHz, Chloroform-d, 25°C) of *N,N*-diethyl-3-(trimethylsilyl)prop-2-yn-1-amine (**3ad**). ..... S84

Figure S93. $^{13}$C{$^{1}$H} NMR (101 MHz, Chloroform-d, 25°C) of *N,N*-diethyl-3-(trimethylsilyl)prop-2-yn-1-amine (**3ad**). ..... S84

Figure S94. $^{29}$Si NMR (79 MHz, Chloroform-d, 25°C) of *N,N*-diethyl-3-(trimethylsilyl)prop-2-yn-1-amine (**3ad**). ..... S85

(Oxybis(prop-1-yne-3,1-diyl))bis(trimethylsilane) (**3ae**) ..... S86

Figure S95. $^{1}$H NMR (400 MHz, Chloroform-d, 25°C) of (oxybis(prop-1-yne-3,1-diyl))bis(trimethylsilane) (**3ae**). ..... S86

Figure S96. $^{13}$C{$^{1}$H} NMR (101 MHz, Chloroform-d, 25°C) of (oxybis(prop-1-yne-3,1-diyl))bis(trimethylsilane) (**3ae**). ..... S86

Figure S97. $^{29}$Si NMR (79 MHz, Chloroform-d, 25°C) of (oxybis(prop-1-yne-3,1-diyl))bis(trimethylsilane) (**3ae**). ..... S87

Trimethyl((4-((trimethylsilyl)ethynyl)benzyl)oxy)silane (**3af**) ..... S88

Figure S98. $^{1}$H NMR (400 MHz, Chloroform-d, 25°C) of trimethyl((4-((trimethylsilyl)ethynyl)benzyl)oxy)silane (**3af**). ..... S88

Figure S99. $^{13}$C{$^{1}$H} NMR (101 MHz, Chloroform-d, 25°C) of trimethyl((4-((trimethylsilyl)ethynyl)benzyl)oxy)silane (**3af**). ..... S88

Figure S100. $^{29}$Si NMR (79 MHz, Chloroform-d, 25°C) of trimethyl((4-((trimethylsilyl)ethynyl)benzyl)oxy)silane (**3af**). ..... S89

Trimethyl(3-phenyl-3-((trimethylsilyl)oxy)prop-1-yn-1-yl)silane (**3ag**) ..... S90

Figure S101. $^{1}$H NMR (400 MHz, Chloroform-d, 25°C) of trimethyl(3-phenyl-3-((trimethylsilyl)oxy)prop-1-yn-1-yl)silane (**3ag**). ..... S90

Figure S102. $^{13}$C{$^{1}$H} NMR (101 MHz, Chloroform-d, 25°C) of trimethyl(3-phenyl-3-((trimethylsilyl)oxy)prop-1-yn-1-yl)silane (**3ag**). ..... S90

Figure S103. $^{29}$Si NMR (79 MHz, Chloroform-d, 25°C) of trimethyl(3-phenyl-3-((trimethylsilyl)oxy)prop-1-yn-1-yl)silane (**3ag**). ..... S91

Trimethyl(3-(oxiran-2-ylmethoxy)prop-1-yn-1-yl)silane (**3ah**) ..... S92

Figure S104. $^{1}$H NMR (400 MHz, Chloroform-d, 25°C) of trimethyl(3-(oxiran-2-ylmethoxy)prop-1-yn-1-yl)silane (**3ah**). ..... S92

Figure S105. $^{13}$C{$^{1}$H} NMR (101 MHz, Chloroform-d, 25°C) of trimethyl(3-(oxiran-2-ylmethoxy)prop-1-yn-1-yl)silane (**3ah**).. S92

Figure S106. $^{29}$Si NMR (79 MHz, Chloroform-d, 25°C) of trimethyl(3-(oxiran-2-ylmethoxy)prop-1-yn-1-yl)silane (**3ah**). ..... S93

Methyl 4-((trimethylsilyl)ethynyl)benzoate (**3ai**) ..... S94

Figure S107. $^{1}$H NMR (400 MHz, Chloroform-d, 25°C) of methyl 4-((trimethylsilyl)ethynyl)benzoate (**3ai**). ..... S94

Figure S108. $^{13}$C{$^{1}$H} NMR (101 MHz, Chloroform-d, 25°C) of methyl 4-((trimethylsilyl)ethynyl)benzoate (**3ai**). ..... S94

Figure S109. $^{29}$Si NMR (79 MHz, Chloroform-d, 25°C) of methyl 4-((trimethylsilyl)ethynyl)benzoate (**3ai**). ..... S95

(1-(4-Ethynylphenyl)-2,2,2-trifluoroethoxy)trimethylsilane (**3aj**) ..... S96

Figure S110. $^{1}H$ NMR (400 MHz, Chloroform-d, 25°C) of (1-(4-ethynylphenyl)-2,2,2-trifluoroethoxy)trimethylsilane (**3aj**)........S96
Figure S111. $^{13}C\{^{1}H\}$ NMR (101 MHz, Chloroform-d, 25°C) of (1-(4-ethynylphenyl)-2,2,2-trifluoroethoxy)trimethylsilane (**3aj**). ........S96
Figure S112. $^{29}Si$ NMR (79 MHz, Chloroform-d, 25°C) of (1-(4-ethynylphenyl)-2,2,2-trifluoroethoxy)trimethylsilane (**3aj**).......S97

*N*-benzyl-N-methyl-3-(trimethylsilyl)prop-2-yn-1-amine (**3ak**) ........S98

Figure S113. $^{1}H$ NMR (400 MHz, Chloroform-d, 25°C) of *N*-benzyl-N-methyl-3-(trimethylsilyl)prop-2-yn-1-amine (**3ak**).........S98
Figure S114. $^{13}C\{^{1}H\}$ NMR (101 MHz, Chloroform-d, 25°C) of *N*-benzyl-N-methyl-3-(trimethylsilyl)prop-2-yn-1-amine (**3ak**).S98
Figure S115. $^{29}Si$ NMR (79 MHz, Chloroform-d, 25°C) of *N*-benzyl-N-methyl-3-(trimethylsilyl)prop-2-yn-1-amine (**3ak**). ........S99

*(R)-N*-(3-(trimethylsilyl)prop-2-yn-1-yl)-2,3-dihydro-1H-inden-1-amine (**3al**)........S100

Figure S116. $^{1}H$ NMR (400 MHz, Chloroform-d, 25°C) of *(R)-N*-(3-(trimethylsilyl)prop-2-yn-1-yl)-2,3-dihydro-1H-inden-1-amine (**3al**). ........S100
Figure S117. $^{13}C\{^{1}H\}$ NMR (101 MHz, Chloroform-d, 25°C) of *(R)-N*-(3-(trimethylsilyl)prop-2-yn-1-yl)-2,3-dihydro-1H-inden-1-amine (**3al**). ........S100
Figure S118. $^{29}Si$ NMR (79 MHz, Chloroform-d, 25°C) of *(R)-N*-(3-(trimethylsilyl)prop-2-yn-1-yl)-2,3-dihydro-1H-inden-1-amine (**3al**). ........S101

Trimethyl(1-(*o*-tolyl)ethoxy)silane (**5a**) ........S102

Figure S119. $^{1}H$ NMR (400 MHz, Chloroform-d, 25°C) of trimethyl(1-(*o*-tolyl)ethoxy)silane (**5a**). ........S102
Figure S120. $^{13}C\{^{1}H\}$ NMR (101 MHz, Chloroform-d, 25°C) of trimethyl(1-(*o*-tolyl)ethoxy)silane (**5a**). ........S102
Figure S121. $^{29}Si$ NMR (79 MHz, Chloroform-d, 25°C) of trimethyl(1-(*o*-tolyl)ethoxy)silane (**5a**). ........S103

(1-(3-Methoxyphenyl)ethoxy)trimethylsilane (**5b**) ........S104

Figure S122. $^{1}H$ NMR (400 MHz, Chloroform-d, 25°C) of (1-(3-methoxyphenyl)ethoxy)trimethylsilane (**5b**). ........S104
Figure S123. $^{13}C\{^{1}H\}$ NMR (101 MHz, Chloroform-d, 25°C) of (1-(3-methoxyphenyl)ethoxy)trimethylsilane (**5b**). ........S104
Figure S124. $^{29}Si$ NMR (79 MHz, Chloroform-d, 25°C) of (1-(3-methoxyphenyl)ethoxy)trimethylsilane (**5b**). ........S105

(1-(4-Chlorophenyl)ethoxy)trimethylsilane (**5c**) ........S106

Figure S125. $^{1}H$ NMR (400 MHz, Chloroform-d, 25°C) of (1-(4-chlorophenyl)ethoxy)trimethylsilane (**5c**). ........S106
Figure S126. $^{13}C\{^{1}H\}$ NMR (101 MHz, Chloroform-d, 25°C) of (1-(4-chlorophenyl)ethoxy)trimethylsilane (**5c**). ........S106
Figure S127. $^{29}Si$ NMR (79 MHz, Chloroform-d, 25°C) of (1-(4-chlorophenyl)ethoxy)trimethylsilane (**5c**). ........S107

1,1,1-Trimethyl-3,3,3-tripropyldisiloxane (**5d**) ........S108

Figure S128. $^{1}H$ NMR (400 MHz, Chloroform-d, 25°C) of 1,1,1-trimethyl-3,3,3-tripropyldisiloxane (**5d**). ........S108
Figure S129. $^{13}C\{^{1}H\}$ NMR (101 MHz, Chloroform-d, 25°C) of 1,1,1-trimethyl-3,3,3-tripropyldisiloxane (**5d**). ........S108
Figure S130. $^{29}Si$ NMR (79 MHz, Chloroform-d, 25°C) of 1,1,1-trimethyl-3,3,3-tripropyldisiloxane (**5d**). ........S109

1,1,1-Trimethyl-3,3,3-triisopropyldisiloxane (**5e**) ........S110

Figure S131. $^{1}H$ NMR (400 MHz, Chloroform-d, 25°C) of 1,1,1-trimethyl-3,3,3-triisopropyldisiloxane (**5e**). ........S110
Figure S132. $^{13}C\{^{1}H\}$ NMR (101 MHz, Chloroform-d, 25°C) of 1,1,1-trimethyl-3,3,3-triisopropyldisiloxane (**5e**). ........S110
Figure S133. $^{29}Si$ NMR (79 MHz, Chloroform-d, 25°C) of 1,1,1-trimethyl-3,3,3-triisopropyldisiloxane (**5e**). ........S111

1-(*Tert*-butyl)-3,3,3-trimethyl-1,1-diphenyldisiloxane (**5f**) ........S112

Figure S134. $^{1}H$ NMR (400 MHz, Chloroform-d, 25°C) of 1-(*tert*-butyl)-3,3,3-trimethyl-1,1-diphenyldisiloxane (**5f**). ........S112
Figure S135. $^{13}C\{^{1}H\}$ NMR (101 MHz, Chloroform-d, 25°C) of 1-(*tert*-butyl)-3,3,3-trimethyl-1,1-diphenyldisiloxane (**5f**). ........S112
Figure S136. $^{29}Si$ NMR (79 MHz, Chloroform-d, 25°C) of 1-(*tert*-butyl)-3,3,3-trimethyl-1,1-diphenyldisiloxane (**5f**). ........S113

(Cyclopropyl(phenyl)methoxy)trimethylsilane (**5g**) ........S114

Figure S137. $^{1}H$ NMR (400 MHz, Chloroform-d, 25°C) of (cyclopropyl(phenyl)methoxy)trimethylsilane (**5g**). ........S114
Figure S138. $^{13}C\{^{1}H\}$ NMR (101 MHz, Chloroform-d, 25°C) of (cyclopropyl(phenyl)methoxy)trimethylsilane (**5g**). ........S114
Figure S139. $^{29}Si$ NMR (79 MHz, Chloroform-d, 25°C) of (cyclopropyl(phenyl)methoxy)trimethylsilane (**5g**). ........S115

REFERENCES ........S116

# GENERAL INFORMATION

Trifluorotrimethylsilane and trifluorotriethylsilane were obtained from Fluorochem and used as received, without further purification. Solvents used in the experiments were obtained from Honeywell, dried over calcium hydride ($CaH_2$), and purified by distillation. Alkynes, alcohols, silanols, amines, and thiols were sourced from various suppliers, including Sigma-Aldrich, Ambeed, Fisher, Acros, Fluorochem, Angene, and TCI, and were used as received. Reaction progress – primarily the conversion of terminal alkyne – was monitored by gas chromatography (GC) using a Bruker Scion 460-GC and an Agilent 5977B GC/MSD equipped with an Agilent 8860 GC system. The structures of products were determined by NMR spectroscopy, and mass spectrometry (GC-MS). The $^{1}$H NMR (400 or 600 MHz), $^{13}$C NMR (101 or 151 MHz), and $^{29}$Si NMR (79 or 119 MHz) spectra were recorded on Bruker Avance III HD NanoBay spectrometer, using chloroform-d ($CDCl_3$) or benzene-d6 ($C_6D_6$) as the solvent. Deuterated solvent was purchased from Sigma Aldrich (Merck) ($CDCl_3$ 99.8 atom% D) and used as received.

# OPTIMIZATION OF REACTION CONDITIONS

**Table 1** Optimization studies for a KOH-catalyzed silylation of alkynes with trifluoromethyltrimethylsilane[a]

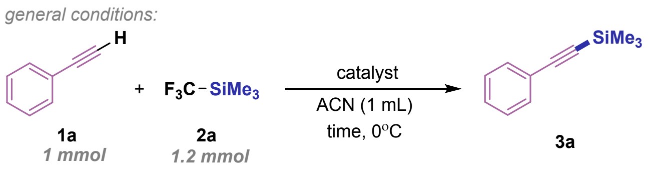


| entry | variation of conditions[b] | Time [min.] | conversion of **1a** [isolated yield, %][b] |
|---|---|---|---|
| 1 | 5 mol% of KHMDS[c] | 15 | 96 |
| 2 | 5 mol% of NaHMDS[c] | 15 | 85 |
| 3 | 5 mol% of $KO^tAm$[d] | 15 | 98 |
| **4** | **10 mol% of KOH** | **15** | **99 [96]** |
| 5 | 5 mol% of KOH | 120 | 93 |
| 6 | 10 mol% of NaOH | 15 | 97 |
| 7 | 10 mol% of LiOH | 15 | 12 |
| 8 | 10 mol% of LiOH | 1440 | 17 |
| 9 | 10 mol% of KF | 15 | 98 [93] |
| 10 | neat | 60 | 96 [89] |
| 11 | in toluene | 60 | 56 |
| 12 | in dioxane | 15 | 99 |
| 13 | in 2-MeTHF | 60 | 95 |
| 14 | in $CH_2Cl_2$ | 60 | 52 |
| 15 | under argon atmosphere | 30 | 98 |
| 16 | no catalyst | 120 | 0 |

[a] (General reaction conditions: **1a** (1.0 equiv., 1 mmol, 0.102 g), **2a** (1.2 equiv., 1.2 mmol, 0.17 g), KOH (0.1 equiv., 0.1 mmol, 0.0056 g), acetonitrile (ACN, 1 mL), under air atmosphere, 0°C, 15 min.) [b] (Conversion determined by GC with n-dodecane as internal standard.) [c] (Used as 1M solution in tetrahydrofuran.) [d] (Used as 0.9M solution in cyclohexane.)

# GENERAL SYNTHETIC PROCEDURES

## Synthesis of compounds **3a-3h**

To a 5 mL vial equipped with a magnetic stirring bar, potassium hydroxide (0.1 mmol, 0.0056 g) was added. Then, terminal alkyne (**1a-1h**) (1 mmol), and acetonitrile (1 mL) have been placed in a vial, and the entire reaction system was cooled to 0°C. Next, trifluorotrimethylsilane (**2a**) (1.2 mmol, 0.170 g) was added. In most cases, the reaction required stirring for a period of 15 minutes. Please note, that the reaction is highly exothermic.

After this time, acetonitrile was evaporated under reduced pressure and the remaining reaction mixture was dissolved in diethyl ether ($Et_2O$) and filtered over Celite. After that, $Et_2O$ was evaporated under reduced pressure to give a desired product. The pure products **3a**-**3h** were identified by $^{1}H$ NMR, $^{13}C$ NMR, $^{29}Si$ NMR spectroscopy, and mass spectrometry.

## Synthesis of compound **3a'**

To a 5 mL vial equipped with a magnetic stirring bar, potassium hydroxide (0.1 mmol, 0.0056 g) was added. Then, phenylacetylene (**1a**) (1 mmol, 0.102 g), and acetonitrile (1 mL) have been placed in a vial, and the entire reaction system was cooled to 0°C. Next, trifluorotriethylsilane (**2a'**) (1.2 mmol, 0.221 g) was added. In most cases, the reaction required stirring for a period of 15 minutes. Please note, that the reaction is highly exothermic.

After this time, acetonitrile was evaporated under reduced pressure and the remaining reaction mixture was dissolved in diethyl ether ($Et_2O$) and filtered over Celite. After that, $Et_2O$ was evaporated under reduced pressure to give a desired product **3a'**. The pure product was identified by $^{1}H$ NMR, $^{13}C$ NMR, $^{29}Si$ NMR spectroscopy, and mass spectrometry.

## Synthesis of compounds **3i-3q**

To a 5 mL vial equipped with a magnetic stirring bar, potassium hydroxide (0.2 mmol, 0.0112 g) was added. Then, terminal alkyne (**1i-1q**) (1 mmol), and acetonitrile (1 mL) have been placed in a vial, and the entire reaction system was cooled to 0°C. Next, trifluorotrimethylsilane (**2a**) (2.4 mmol, 0.340 g) was added. In most cases, the reaction required stirring for a period of 15 minutes. Please note, that the reaction is highly exothermic.

After this time, acetonitrile was evaporated under reduced pressure and the remaining reaction mixture was dissolved in diethyl ether ($Et_2O$) and filtered over Celite. After that, $Et_2O$ was evaporated under reduced pressure to give a desired product. The pure products **3i-3q** were identified by $^{1}H$ NMR, $^{13}C$ NMR, $^{29}Si$ NMR spectroscopy, and mass spectrometry.

## Synthesis of compounds **3r-3t**

To a 5 mL vial equipped with a magnetic stirring bar, potassium hydroxide (0.1 mmol, 0.0056 g) was added. Then, terminal alkyne (**1r-1t**) (1 mmol), and acetonitrile (1 mL) have been placed in a vial, and the entire reaction system was cooled to 0°C. Next, trifluorotrimethylsilane (**2a**) (1.2 mmol, 0.170 g) was added. In most cases, the reaction required stirring for a period of 15 minutes. Please note, that the reaction is highly exothermic.

After this time, acetonitrile was evaporated under reduced pressure and the remaining reaction mixture was dissolved in diethyl ether ($Et_2O$) and filtered over Celite. After that, $Et_2O$ was evaporated under reduced pressure to give a desired product. The pure products **3r**-**3t** were identified by $^{1}H$ NMR, $^{13}C$ NMR, $^{29}Si$ NMR spectroscopy, and mass spectrometry.

## Synthesis of compounds **3u-3x**

To a 5 mL vial equipped with a magnetic stirring bar, potassium hydroxide (0.2 mmol, 0.0112 g) was added. Then, terminal alkyne (**1u-1x**) (1 mmol), and acetonitrile (1 mL) have been placed in a vial, and the entire reaction system was

cooled to 0°C. Next, trifluorotrimethylsilane (**2a**) (2.0 mmol, 0.284 g) was added. In most cases, the reaction required stirring for a period of 15 minutes. Please note, that the reaction is highly exothermic.

After this time, acetonitrile was evaporated under reduced pressure and the remaining reaction mixture was dissolved in diethyl ether($Et_2O$) and filtered over Celite. After that, $Et_2O$ was evaporated under reduced pressure to give a desired product. The pure products **3u-3x** were identified by $^1$H NMR, $^{13}$C NMR, $^{29}$Si NMR spectroscopy, and mass spectrometry.

## Synthesis of compound **3y**

To a 5 mL vial equipped with a magnetic stirring bar, potassium hydroxide (0.2 mmol, 0.0112 g) was added. Then, terminal alkyne (**1y**) (1 mmol), and acetonitrile (1 mL) and toluene (1 mL) have been placed in a vial, and the entire reaction system was cooled to 0°C. Next, trifluorotrimethylsilane (**2a**) (2.0 mmol, 0.284 g) was added. The reaction was stirred for 60 minutes to ensure high conversion rate. Please note, that the reaction is highly exothermic.

After this time, acetonitrile and toluene were evaporated under reduced pressure and the remaining reaction mixture was dissolved in diethyl ether ($Et_2O$) and filtered over Celite. After that, $Et_2O$ was evaporated under reduced pressure to give a desired product. The pure product **3y** was identified by $^1$H NMR, $^{13}$C NMR, and $^{29}$Si NMR spectroscopy.

## Synthesis of compounds **3aa**-**3ad**, and **3ah-3aj**

To a 5 mL vial equipped with a magnetic stirring bar, potassium hydroxide (0.1 mmol, 0.0056 g) was added. Then, terminal alkyne (**1aa-1ad, 1ah-1aj**) (1 mmol), and acetonitrile (1 mL) have been placed in a vial, and the entire reaction system was cooled to 0°C. Next, trifluorotrimethylsilane (**2a**) (1.2 mmol, 0.170 g) was added. In most cases, the reaction required stirring for a period of 15 minutes. Please note, that the reaction is highly exothermic.

After this time, acetonitrile was evaporated under reduced pressure and the remaining reaction mixture was dissolved in diethyl ether ($Et_2O$) and filtered over Celite. After that, $Et_2O$ was evaporated under reduced pressure to give a desired product. The pure products **3aa-3ad, and 3ah-3aj** were identified by $^1$H NMR, $^{13}$C NMR, $^{29}$Si NMR spectroscopy, and mass spectrometry.

## Synthesis of compounds **3ae-3ag** and **3ak-3am**

To a 5 mL vial equipped with a magnetic stirring bar, potassium hydroxide (0.2 mmol, 0.0112 g) was added. Then, terminal alkyne (**1ae-1ag, 1ak-1am**) (1 mmol), and acetonitrile (1 mL) have been placed in a vial, and the entire reaction system was cooled to 0°C. Next, trifluorotrimethylsilane (**2a**) (2.4 mmol, 0.340 g) was added. In most cases, the reaction required stirring for a period of 15 minutes. Please note, that the reaction is highly exothermic.

After this time, acetonitrile was evaporated under reduced pressure and the remaining reaction mixture was dissolved in diethyl ether ($Et_2O$) and filtered over Celite. After that, $Et_2O$ was evaporated under reduced pressure to give a desired product. The pure products **3ae-3ag and 3ak-3am** were identified by $^1$H NMR, $^{13}$C NMR, $^{29}$Si NMR spectroscopy, and mass spectrometry.

## Synthesis of compounds **5a-5g**

To a 5 mL vial equipped with a magnetic stirring bar, potassium hydroxide (0.1 mmol, 0.0056 g) was added. Then, alcohol/silanol (**4a-4g**) (1 mmol), and acetonitrile (1 mL) have been placed in a vial, and the entire reaction system was cooled to 0°C. Next, trifluorotrimethylsilane (**2a**) (1.2 mmol, 0.170 g) was added. In most cases, the reaction required stirring for a period of 15 minutes. Please note, that the reaction is highly exothermic.

After this time, acetonitrile was evaporated under reduced pressure and the remaining reaction mixture was dissolved in diethyl ether ($Et_2O$) and filtered over Celite. After that, $Et_2O$ was evaporated under reduced pressure to give a desired product. The pure products **5a-5g** were identified by $^1$H NMR, $^{13}$C NMR, $^{29}$Si NMR spectroscopy, and mass spectrometry.

## Scaled-up synthesis of **3a**

To a 25 mL vial equipped with a magnetic stirring bar, potassium hydroxide (1 mmol, 0.0560 g) was added. Then, phenylacetylene (**1a**) (10 mmol, 1.02 g), and acetonitrile (5 mL) have been placed in a vial, and the entire reaction system was cooled to 0°C. Next, trifluorotrimethylsilane (**2a'**) (12 mmol, 1.70 g) was added. In most cases, the reaction required stirring for a period of 15 minutes. Please note, that the reaction is highly exothermic.

After this time, acetonitrile was evaporated under reduced pressure and the remaining reaction mixture was dissolved in diethyl ether ($Et_2O$) and filtered over Celite. After that, $Et_2O$ was evaporated under reduced pressure to give a desired product **3a**. The pure product **3a** was obtained in 97% yield (1.69 g).

## Experiments in the presence of radical scavengers

To a 5 mL vial equipped with a magnetic stirring bar, potassium hydroxide (0.1 mmol, 0.0056 g) and radical scavenger (TEMPO or galvinoxyl, 1 mmol) were added. Then, phenylacetylene (**1a**) (1.0 mmol, 0.102 g), and acetonitrile (1 mL) have been placed in a vial, and the entire reaction system was cooled to 0°C. Next, trifluorotrimethylsilane (**2a'**) (1.2 mmol, 0.170 g) was added. The reactions were stirred for 60 minutes. After this time, the reaction mixture was checked *via* GC and GC-MS.

## Radical clock experiment

To a 5 mL vial equipped with a magnetic stirring bar, potassium hydroxide (0.1 mmol, 0.0056 g) was added. Then, (**4h**) (1 mmol), and acetonitrile (1 mL) have been placed in a vial, and the entire reaction system was cooled to 0°C. Next, trifluorotrimethylsilane (**2a**) (1.2 mmol, 0.170 g) was added. The reaction was stirred for 60 minutes to ensure high conversion rate. Please note, that the reaction is highly exothermic.

After this time, acetonitrile was evaporated under reduced pressure and the remaining reaction mixture was dissolved in diethyl ether ($Et_2O$) and filtered over Celite. After that, $Et_2O$ was evaporated under reduced pressure to give a desired product. The pure product **5h** was identified by $^{1}H$ NMR, $^{13}C$ NMR, $^{29}Si$ NMR spectroscopy, and mass spectrometry.

# CHARACTERIZATION DATA FOR ALL PRODUCTS

## Trimethyl(phenylethynyl)silane (**3a**)

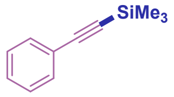


Trimethyl(phenylethynyl)silane was obtained in 96% yield as a pale-yellow oil (0.167 g). The title compound was known in the literature, and all spectroscopic data are in agreement.[1]

$^{1}$H NMR: (400 MHz, $CDCl_3$) δ 7.54 – 7.42 (m, 2H), 7.34 – 7.24 (m, 3H), 0.25 (s, 9H).

$^{13}$C{$^{1}$H} NMR: (101 MHz, $CDCl_3$) δ 132.1, 128.6, 128.3, 123.3, 105.3, 94.2, 0.1.

$^{29}$Si NMR: (79 MHz, $CDCl_3$) δ -17.8.

EI-MS m/z (rel. int.): 174 ([M]$^+$, 25%), 159 (100), 129 (15), 115 (35).

## Trimethyl((4-propylphenyl)ethynyl)silane (**3b**)

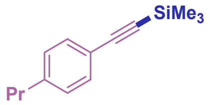


Trimethyl((4-propylphenyl)ethynyl)silane was obtained in 86% yield as a pale-yellow oil (0.186 g). The title compound was known in the literature, and all spectroscopic data are in agreement.[1]

$^{1}$H NMR: (400 MHz, $CDCl_3$) δ 7.96 – 7.34 (m, 2H), 7.18 – 6.85 (m, 2H), 2.61 – 2.47 (m, 2H), 1.84 – 1.41 (m, 2H), 1.11 – 0.73 (m, 3H), 0.25 (s, 9H).

$^{13}$C{$^{1}$H} NMR: (101 MHz, $CDCl_3$) δ 143.8, 132.3, 128.8, 105.9, 93.7, 38.4, 24.7, 14.1, 0.5.

$^{29}$Si NMR: (79 MHz, $CDCl_3$) δ -18.0.

EI-MS m/z (rel. int.): 216 ([M]$^+$, 25%), 201 (100), 172 (15), 115 (5).

## ([1,1'-Biphenyl]-4-ylethynyl)trimethylsilane (**3c**)

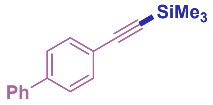


([1,1'-Biphenyl]-4-ylethynyl)trimethylsilane was obtained in 90% yield as a pale-yellow oil (0.225 g). The title compound was known in the literature, and all spectroscopic data are in agreement.[1]

$^{1}$H NMR: (400 MHz, $CDCl_3$) δ 7.61 – 7.57 (m, 2H), 7.54 (s, 3H), 7.47 – 7.41 (m, 2H), 7.39 – 7.32 (m, 1H), 0.27 (s, 9H).

$^{13}$C{$^{1}$H} NMR: (101 MHz, $CDCl_3$) δ 141.3, 140.5, 132.5, 129.0, 127.8, 127.2, 127.0, 122.2, 105.1, 95.0, 0.1.

$^{29}$Si NMR: (79 MHz, $CDCl_3$) δ -17.7.

EI-MS m/z (rel. int.): 250 ([M]$^+$, 45%), 235 (100), 205 (5), 165 (5).

## ((4-Ethoxyphenyl)ethynyl)trimethylsilane (**3d**)

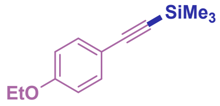


((4-Ethoxyphenyl)ethynyl)trimethylsilane was obtained in 99% yield as a pale-yellow oil (0.215 g). The title compound was known in the literature, and all spectroscopic data are in agreement.[2]

$^{1}H$ NMR: (400 MHz, $CDCl_3$) δ 7.58 – 7.31 (m, 2H), 6.98 – 6.68 (m, 2H), 4.02 (q, *J* = 7.0 Hz, 2H), 1.41 (t, *J* = 7.0 Hz, 3H), 0.24 (s, 9H).

$^{13}C\{^{1}H\}$ NMR: (101 MHz, $CDCl_3$) δ 159.3, 133.6, 115.2, 114.5, 105.4, 92.5, 63.6, 14.9, 0.2.

$^{29}Si$ NMR: (79 MHz, $CDCl_3$) δ -18.2.

EI-MS m/z (rel. int.): 218 ($[M]^+$, 35%), 203 (100), 175 (25), 146 (10).

## ((2-Methoxyphenyl)ethynyl)trimethylsilane (**3e**)

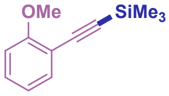


((2-Methoxyphenyl)ethynyl)trimethylsilane was obtained in 99% yield as a pale-yellow oil (0.202 g). The title compound was known in the literature, and all spectroscopic data are in agreement.[3]

$^{1}H$ NMR: (400 MHz, $CDCl_3$) δ 7.31 – 7.24 (m, 1H), 7.15 – 7.03 (m, 1H), 6.79 – 6.64 (m, 2H), 3.71 (s, 3H), 0.10 (s, 9H).

$^{13}C\{^{1}H\}$ NMR: (101 MHz, $CDCl_3$) δ 160.8, 134.6, 130.4, 120.8, 112.8, 111.2, 101.8, 98.9, 56.3, 0.6.

$^{29}Si$ NMR: (79 MHz, $CDCl_3$) δ -17.8.

EI-MS m/z (rel. int.): 204 ($[M]^+$, 50%), 189 (100), 159 (35), 115 (30).

## ((3-Methoxyphenyl)ethynyl)trimethylsilane (**3f**)

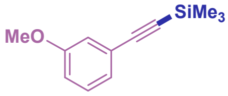


((3-Methoxyphenyl)ethynyl)trimethylsilane was obtained in 82% yield as a pale-yellow oil (0.167 g). The title compound was known in the literature, and all spectroscopic data are in agreement.[4]

$^{1}H$ NMR: (400 MHz, $CDCl_3$) δ 7.24 – 7.15 (m, 1H), 7.10 – 7.03 (m, 1H), 7.00 – 6.95 (m, 1H), 6.90 – 6.85 (m, 1H), 3.80 (s, 3H), 0.25 (s, 9H).

$^{13}C\{^{1}H\}$ NMR: (101 MHz, $CDCl_3$) δ 159.7, 129.7, 125.0, 124.6, 117.0, 115.8, 105.5, 94.4, 55.7, 0.4.

$^{29}Si$ NMR: (79 MHz, $CDCl_3$) δ -17.7.

EI-MS m/z (rel. int.): 204 ($[M]^+$, 35%), 189 (100), 146 (15), 114 (5).

## ((4-Methoxyphenyl)ethynyl)trimethylsilane (**3g**)

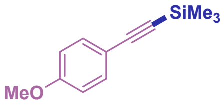


((3-Methoxyphenyl)ethynyl)trimethylsilane was obtained in 99% yield as a pale-yellow oil (0.201 g). The title compound was known in the literature, and all spectroscopic data are in agreement.[1]

$^{1}H$ NMR: (400 MHz, $CDCl_3$) δ 7.41 (d, *J* = 8.9 Hz, 2H), 6.82 (d, *J* = 8.9 Hz, 2H), 3.80 (s, 3H), 0.24 (s, 9H).

$^{13}C\{^{1}H\}$ NMR: (101 MHz, $CDCl_3$) δ 159.9, 133.6, 115.4, 113.9, 105.3, 92.5, 55.4, 0.2.

$^{29}Si$ NMR: (79 MHz, $CDCl_3$) δ -18.2.

EI-MS m/z (rel. int.): 204 ($[M]^+$, 40%), 189 (100), 174 (15), 143 (20).

## Trimethyl(naphthalen-1-ylethynyl)silane (**3h**)

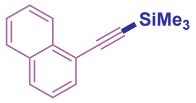


Trimethyl(naphthalen-1-ylethynyl)silane was obtained in 85% yield as an oil (0.190 g). The title compound was known in the literature, and all spectroscopic data are in agreement.[1]

$^1$H NMR: (400 MHz, $CDCl_3$) δ 8.40 – 8.33 (m, 1H), 7.89 – 7.81 (m, 2H), 7.72 (dd, *J* = 7.2, 1.2 Hz, 1H), 7.64 – 7.48 (m, 2H), 7.47 – 7.38 (m, 1H), 0.36 (s, 9H).

$^{13}$C{$^1$H} NMR: (101 MHz, $CDCl_3$) δ 133.5, 133.2, 131.0, 129.1, 128.4, 127.0, 126.5, 126.3, 125.3, 120.9, 103.2, 99.6, 0.3.

$^{29}$Si NMR: (79 MHz, $CDCl_3$) δ -17.5.

EI-MS m/z (rel. int.): 209 ($[M-CH_3]^+$, 20%), 193 (100), 163 (5), 115 (10).

## ((2-Fluorophenyl)ethynyl)trimethylsilane (**3i**)

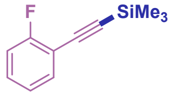


((2-Fluorophenyl)ethynyl)trimethylsilane was obtained in 98% yield as an oil (0.188 g). The title compound was known in the literature, and all spectroscopic data are in agreement.[1]

$^1$H NMR: (400 MHz, $CDCl_3$) δ 7.49 – 7.40 (m, 1H), 7.35 – 7.20 (m, 1H), 7.10 – 6.96 (m, 2H), 0.25 (s, 9H).

$^{13}$C{$^1$H} NMR: (101 MHz, $CDCl_3$) δ 163.1 (d, *J* = 252.0 Hz), 134.00 (d, *J* = 1.4 Hz), 130.29 (d, *J* = 8.0 Hz), 123.92 (d, *J* = 3.8 Hz), 115.54 (d, *J* = 21.0 Hz), 111.88 (d, *J* = 15.8 Hz), 100.2, 98.0, 0.0.

$^{19}$F NMR: (377 MHz, $CDCl_3$) δ -109.7.

$^{29}$Si NMR: (79 MHz, $CDCl_3$) δ –17.2.

EI-MS m/z (rel. int.): 192 ($[M]^+$, 45%), 177 (100), 149 (55), 115 (75).

## ((3-Fluorophenyl)ethynyl)trimethylsilane (**3j**)

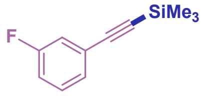


((3-Fluorophenyl)ethynyl)trimethylsilane was obtained in 97% yield as an oil (0.186 g). The title compound was known in the literature, and all spectroscopic data are in agreement.[5]

$^1$H NMR: (400 MHz, $CDCl_3$) δ 7.25 – 7.20 (m, 2H), 7.17 – 7.10 (m, 1H), 7.04 – 6.95 (m, 1H), 0.23 (s, 9H).

$^{13}$C{$^1$H} NMR: (101 MHz, $CDCl_3$) δ 162.4 (d, *J* = 246.6 Hz), 129.9 (d, *J* = 8.6 Hz), 128.0 (d, *J* = 3.1 Hz), 125.1 (d, *J* = 9.4 Hz), 118.8 (d, *J* = 22.8 Hz), 116.0 (d, *J* = 21.1 Hz), 103.7, 95.5, 0.0.

$^{19}$F NMR: (377 MHz, $CDCl_3$) δ -113.1.

$^{29}$Si NMR: (79 MHz, $CDCl_3$) δ –17.4.

EI-MS m/z (rel. int.): 192 ($[M]^+$, 15%), 177 (100), 147 (10), 133 (10).

## ((4-Fluorophenyl)ethynyl)trimethylsilane (**3k**)

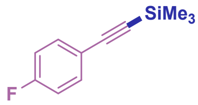


((4-Fluorophenyl)ethynyl)trimethylsilane was obtained in 92% yield as an oil (0.177 g). The title compound was known in the literature, and all spectroscopic data are in agreement.[5]

$^{1}H$ NMR: (400 MHz, $CDCl_3$) δ 7.48 – 7.42 (m, 2H), 6.99 (t, $J$ = 8.7 Hz, 2H), 0.25 (s, 9H).

$^{13}C\{^{1}H\}$ NMR: (101 MHz, $CDCl_3$) δ 162.7 (d, $J$ = 249.6 Hz), 134.0 (d, $J$ = 8.4 Hz), 119.0 (d, $J$ = 3.5 Hz), 115.6 (d, $J$ = 22.0 Hz), 104.1, 94.0, 0.1.

$^{19}F$ NMR: (377 MHz, $CDCl_3$) δ -110.5.

$^{29}Si$ NMR: (79 MHz, $CDCl_3$) δ -17.7.

EI-MS m/z (rel. int.): 192 ($[M]^+$, 15%), 177 (100), 147 (15), 133 (20).

## ((4-Chlorophenyl)ethynyl)trimethylsilane (**3l**)

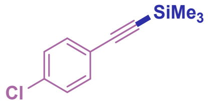


((4-Chlorophenyl)ethynyl)trimethylsilane was obtained in 85% yield as an oil (0.176 g). The title compound was known in the literature, and all spectroscopic data are in agreement.[1]

$^{1}H$ NMR: (400 MHz, $CDCl_3$) δ 7.28 – 7.22 (m, 2H), 7.15 – 7.10 (m, 2H), 0.11 (s, 9H).

$^{13}C\{^{1}H\}$ NMR: (101 MHz, $CDCl_3$) δ 134.6, 133.3, 128.7, 121.8, 104.0, 95.5, 0.0.

$^{29}Si$ NMR: (79 MHz, $CDCl_3$) δ -17.5.

EI-MS m/z (rel. int.): 208 ($[M]^+$, 20%), 193 (100), 163 (5), 115 (10).

## Trimethyl((2-(trifluoromethoxy)phenyl)ethynyl)silane (**3m**)

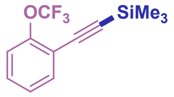


Trimethyl((2-(trifluoromethoxy)phenyl)ethynyl)silane was obtained in 91% yield as a pale-yellow oil (0.235 g). The title compound was known in the literature, and all spectroscopic data are in agreement.[1]

$^{1}H$ NMR: (400 MHz, $CDCl_3$) δ 7.54 (dd, $J$ = 7.5, 1.6 Hz, 1H), 7.35 (dd, $J$ = 7.1, 1.5 Hz, 1H), 7.30 – 7.22 (m, 2H), 0.29 (s, 9H).

$^{13}C\{^{1}H\}$ NMR: (101 MHz, $CDCl_3$) δ 150.3, 134.2, 130.1, 127.1, 121.8, 121.11 (q, $J$ = 258.1 Hz), 118.5, 101.1, 99.1, 0.2.

$^{19}F$ NMR: (377 MHz, $CDCl_3$) δ -57.6.

$^{29}Si$ NMR: (79 MHz, $CDCl_3$) δ -16.9.

EI-MS m/z (rel. int.): 258 ($[M]^+$, 20%), 149 (15), 115 (100), 69 (30).

## Trimethyl((4-(trifluoromethyl)phenyl)ethynyl)silane (**3n**)

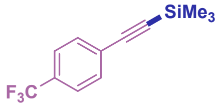


Trimethyl((4-(trifluoromethyl)phenyl)ethynyl)silane was obtained in 86% yield as a pale-yellow oil (0.208 g). The title compound was known in the literature, and all spectroscopic data are in agreement.[1]

$^{1}H$ NMR: (400 MHz, $CDCl_3$) δ 7.56 (s, 4H), 0.26 (s, 9H)

$^{13}C\{^{1}H\}$ NMR: (101 MHz, $CDCl_3$) δ 132.00, 130.0 (q, $J$ = 32.7 Hz), 126.8, 125.0 (q, $J$ = 3.9 Hz), 122.4, 103.2, 97.0, -0.4.

$^{19}F$ NMR: (377 MHz, $CDCl_3$) δ -62.9.

$^{29}Si$ NMR: (79 MHz, $CDCl_3$) δ -17.1.

EI-MS m/z (rel. int.): 242 ($[M]^+$, 15%), 227 (100), 197 (10), 164 (10).

## Trimethyl((3-nitrophenyl)ethynyl)silane (**3o**)

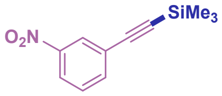


Trimethyl((3-nitrophenyl)ethynyl)silane was obtained in 87% yield as a pale-yellow oil (0.191 g). The title compound was known in the literature, and all spectroscopic data are in agreement.[6]

$^1$H NMR: (400 MHz, $CDCl_3$) δ 8.30 (s, 1H), 8.21 – 8.10 (m, 1H), 7.75 (dt, *J* = 7.8, 1.3 Hz, 1H), 7.48 (t, *J* = 8.0 Hz, 1H), 0.27 (s, 9H).

$^{13}$C{$^1$H} NMR: (101 MHz, $CDCl_3$) δ 148.2, 137.7, 129.4, 126.9, 125.1, 123.3, 102.3, 97.8, -0.1.

$^{29}$Si NMR: (79 MHz, $CDCl_3$) δ -16.8.

EI-MS m/z (rel. int.): 219 ($[M]^+$, 5%), 204 (100), 158 (25), 143 (20).

## Trimethyl((4-nitrophenyl)ethynyl)silane (**3p**)

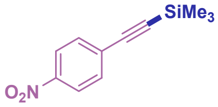


Trimethyl((4-nitrophenyl)ethynyl)silane was obtained in 93% yield as a pale-yellow oil (0.204 g). The title compound was known in the literature, and all spectroscopic data are in agreement.[5]

$^1$H NMR: (400 MHz, $CDCl_3$) δ 8.16 (d, *J* = 9.0 Hz, 2H), 7.59 (d, *J* = 9.0 Hz, 2H), 0.27 (s, 9H).

$^{13}$C{$^1$H} NMR: (101 MHz, $CDCl_3$) δ 147.3, 132.8, 130.1, 123.6, 102.8, 100.7, -0.2.

$^{29}$Si NMR: (79 MHz, $CDCl_3$) δ -16.6.

EI-MS m/z (rel. int.): 219 ($[M]^+$, 15%), 204 (100), 158 (15), 143 (10).

## 4-((Trimethylsilyl)ethynyl)benzonitrile (**3q**)

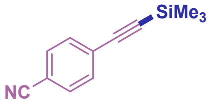


4-((Trimethylsilyl)ethynyl)benzonitrile was obtained in 75% yield as a pale-yellow oil (0.149 g). The title compound was known in the literature, and all spectroscopic data are in agreement.[7]

$^1$H NMR: (400 MHz, $CDCl_3$) δ 7.62 – 7.57 (m, 2H), 7.55 – 7.51 (m, 2H), 0.26 (s, 9H).

$^{13}$C{$^1$H} NMR: (101 MHz, $CDCl_3$) δ 132.3, 131.8, 127.8, 118.3, 111.6, 102.8, 99.4, -0.4.

$^{29}$Si NMR: (79 MHz, $CDCl_3$) δ -16.8.

EI-MS m/z (rel. int.): 199 ($[M]^+$, 20%), 184 (100), 154 (20), 140 (20).

## 2-((Trimethylsilyl)ethynyl)pyridine (**3r**)

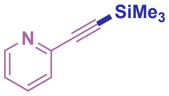


2-((Trimethylsilyl)ethynyl)pyridine was obtained in 91% yield as a pale-yellow oil (0.159 g). The title compound was known in the literature, and all spectroscopic data are in agreement.[1]

$^1$H NMR: (400 MHz, $CDCl_3$) δ 8.62 – 8.47 (m, 1H), 7.67 – 7.56 (m, 1H), 7.51 – 7.35 (m, 1H), 7.22 – 7.14 (m, 1H), 0.25 (s, 9H).

$^{13}$C{$^1$H} NMR: (101 MHz, $CDCl_3$) δ 150.4, 143.6, 136.4, 127.7, 123.4, 104.1, 95.2, 0.1.

$^{29}$Si NMR: (79 MHz, $CDCl_3$) δ -16.6.

EI-MS m/z (rel. int.): 175 ($[M]^+$, 10%), 160 (100), 145 (10), 132 (15).

## Trimethyl(thiophen-3-ylethynyl)silane (**3s**)

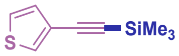


Trimethyl(thiophen-3-ylethynyl)silane was obtained in 92% yield as a pale-yellow oil (0.166 g). The title compound was known in the literature, and all spectroscopic data are in agreement.[1]

$^{1}$H NMR: (400 MHz, $CDCl_3$) δ 7.48 (dd, $J$ = 3.0, 1.2 Hz, 1H), 7.24 (dd, $J$ = 5.0, 3.0 Hz, 1H), 7.13 (dd, $J$ = 5.0, 1.2 Hz, 1H), 0.24 (s, 9H).

$^{13}$C{$^{1}$H} NMR: (101 MHz, $CDCl_3$) δ 130.6, 130.0, 125.6, 122.9, 100.4, 94.3, 0.4.

$^{29}$Si NMR: (79 MHz, $CDCl_3$) δ -17.7.

EI-MS m/z (rel. int.): 180 ($[M]^+$, 20%), 165 (100), 135 (5), 121 (5).

## Trimethyl((phenylthio)ethynyl)silane (**3t**)

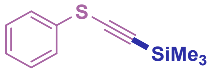


Trimethyl((phenylthio)ethynyl)silane was obtained in 86% yield as a pale-yellow oil (0.177 g). The title compound was known in the literature, and all spectroscopic data are in agreement.[8]

$^{1}$H NMR: (400 MHz, $CDCl_3$) δ 7.47 – 7.41 (m, 2H), 7.39 – 7.33 (m, 2H), 7.26 – 7.20 (m, 1H), 0.27 (s, 9H).

$^{13}$C{$^{1}$H} NMR: (101 MHz, $CDCl_3$) δ 132.5, 129.4, 126.7, 126.3, 106.4, 90.3, 0.0.

$^{29}$Si NMR: (79 MHz, $CDCl_3$) δ -17.0.

EI-MS m/z (rel. int.): 176 (5%), 161 (5), 135 (100), 91 (40).

## Tributyl((trimethylsilyl)ethynyl)silane (**3u**)

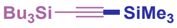


Tributyl((trimethylsilyl)ethynyl)silane was obtained in 84% yield as a pale-yellow oil (0.249 g). The title compound was known in the literature, and all spectroscopic data are in agreement.[9]

$^{1}$H NMR: (400 MHz, $CDCl_3$) δ 1.39 – 1.31 (m, 12H), 0.93 – 0.85 (m, 9H), 0.63 – 0.56 (m, 6H), 0.17 (s, 9H).

$^{13}$C{$^{1}$H} NMR: (101 MHz, $CDCl_3$) δ 115.4, 112.4, 26.5, 26.2, 13.9, 13.1, 0.1.

$^{29}$Si NMR: (79 MHz, $CDCl_3$) δ -13.2, -19.3.

EI-MS m/z (rel. int.): 281 ($[M-CH_3]^+$, 5%), 239 (85), 183 (90), 73 (100).

## Trimethyl((triisopropylsilyl)ethynyl)silane (**3v**)

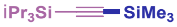


Trimethyl((triisopropylsilyl)ethynyl)silane was obtained in 82% yield as a pale-yellow oil (0.208 g). The title compound was known in the literature, and all spectroscopic data are in agreement.[1]

$^{1}$H NMR: (400 MHz, $CDCl_3$) δ 1.46 – 0.74 (m, 21H), 0.17 (s, 9H).

$^{13}$C{$^{1}$H} NMR: (101 MHz, $CDCl_3$) δ 116.7, 110.7, 19.0, 11.6, 0.5.

$^{29}$Si NMR: (79 MHz, $CDCl_3$) δ -2.8, -19.4.

EI-MS m/z (rel. int.): 239 ($[M- CH_3]^+$, 10%), 211 (100), 155 (40), 141 (55).

## Trimethyl((triphenylgermyl)ethynyl)silane (**3w**)

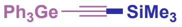


Trimethyl((triphenylgermyl)ethynyl)silane was obtained in 99% yield as a pale-yellow oil (0.398 g). The title compound was known in the literature, and all spectroscopic data are in agreement.[10]

$^{1}$H NMR: (400 MHz, $CDCl_3$) δ 8.21 – 7.54 (m, 6H), 7.51 – 7.16 (m, 9H), 0.28 (s, 9H).

$^{13}$C{$^{1}$H} NMR: (101 MHz, $CDCl_3$) δ 135.3, 134.7, 129.6, 128.5, 117.4, 107.2, 0.2.

$^{29}$Si NMR: (79 MHz, $CDCl_3$) δ -18.4.

EI-MS m/z (rel. int.): 387 ($[M\text{-}CH_3]^+$, 50%), 325 (35), 228 (75), 159 (100).

## (Dimethyl(phenyl)germyl)ethynyl)trimethylsilane (**3x**)

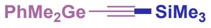


((Dimethyl(phenyl)germyl)ethynyl)trimethylsilane was obtained in 83% yield as a pale-yellow oil (0.231 g). The title compound was known in the literature, and all spectroscopic data are in agreement.

$^{1}$H NMR: (400 MHz, $CDCl_3$) δ 7.71 – 7.55 (m, 2H), 7.48 – 7.35 (m, 3H), 0.59 (s, 6H), 0.22 (s, 9H)

$^{13}$C{$^{1}$H} NMR: (101 MHz, $CDCl_3$) δ 139.2, 133.5, 129.4, 128.6, 114.5, 111.7, 0.5, -0.4.

$^{29}$Si NMR: (79 MHz, $CDCl_3$) δ -19.2.

EI-MS m/z (rel. int.): 278 ($[M]^+$, 15%), 263 (100), 159 (55), 77 (55).

## 1-((Dimethyl((trimethylsilyl)ethynyl)silyl)oxy)-3,5,7,9,11,13,15-heptaisobutyl-2,4,6,8,10,12,14,16,17,18,19,20-dodecaoxa-1,3,5,7,9,11,13,15-octasilapentacyclo[9.5.1.13,9.15,15.17,13]icosane (**3y**)

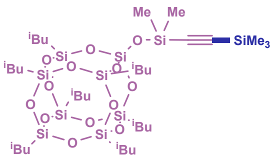

1-((Dimethyl((trimethylsilyl)ethynyl)silyl)oxy)-3,5,7,9,11,13,15-heptaisobutyl-2,4,6,8,10,12,14,16,17,18,19,20-dodecaoxa-1,3,5,7,9,11,13,15-octasilapentacyclo[9.5.1.13,9.15,15.17,13]icosane was obtained in 92% yield as a white solid (0.090 g). The title compound was known in the literature, and all spectroscopic data are in agreement.[11]

$^{1}$H NMR: (400 MHz, $CDCl_3$) δ 2.07 – 1.47 (m, 7H), 1.09 – 0.78 (m, 42H), 0.61 (dd, $J$ = 12.1, 7.0 Hz, 14H), 0.26 (s, 6H), 0.17 (s, 9H).

$^{13}$C{$^{1}$H} NMR: (101 MHz, $CDCl_3$) δ 113.3, 112.1, 26.2, 26.2, 24.3, 24.3, 23.0, 22.8, 2.2, 0.3.

$^{29}$Si NMR: (79 MHz, $CDCl_3$) δ -17.6, -18.7, -67.0, -67.9, -109.9.

## Triethyl(phenylethynyl)silane (**3a’**)

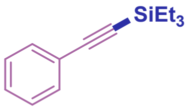


Triethyl(phenylethynyl)silane was obtained in 90% yield as a pale-yellow oil (0.194 g). The title compound was known in the literature, and all spectroscopic data are in agreement.[12]

$^{1}$H NMR: (400 MHz, $CDCl_3$) δ 7.50 – 7.44 (m, 2H), 7.34 – 7.28 (m, 3H), 1.06 (t, $J$ = 7.9 Hz, 9H), 0.68 (qd, $J$ = 7.8, 0.6 Hz, 6H).

$^{13}$C{$^{1}$H} NMR: (101 MHz, $CDCl_3$) δ 132.2, 128.5, 128.3, 123.5, 106.5, 91.7, 7.6, 4.6.

$^{29}Si$ NMR: (79 MHz, $CDCl_3$) δ -7.2.

EI-MS m/z (rel. int.): 216 ($[M]^+$, 5%), 187 (90), 159 (90), 131 (100).

## Dec-1-yn-1-yltrimethylsilane (**3aa**)

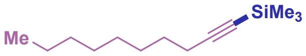


Dec-1-yn-1-yltrimethylsilane was obtained in 88% yield as a pale-yellow oil (0.185 g). The title compound was known in the literature, and all spectroscopic data are in agreement.[1]

$^1H$ NMR: (400 MHz, $CDCl_3$) δ 2.21 (t, $J$ = 7.2 Hz, 2H), 1.59 – 1.47 (m, 2H), 1.42 – 1.34 (m, 2H), 1.31 – 1.18 (m, 8H), 0.97 – 0.83 (m, 3H), 0.15 (s, 9H).

$^{13}C\{^1H\}$ NMR: (101 MHz, $CDCl_3$) δ 108.0, 84.4, 32.0, 29.3, 29.2, 29.0, 28.8, 22.8, 20.0, 14.3, 0.3.

$^{29}Si$ NMR: (79 MHz, $CDCl_3$) δ -19.4.

EI-MS m/z (rel. int.): 195 ($[M - CH_3]^+$, 100%), 109 (80), 73 (60), 59 (40).

## Trimethyl(3-phenylprop-1-yn-1-yl)silane (**3ab**)

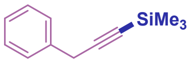


Trimethyl(3-phenylprop-1-yn-1-yl)silane was obtained in 78% yield as a pale-yellow oil (0.147 g). The title compound was known in the literature, and all spectroscopic data are in agreement.[13]

$^1H$ NMR: (400 MHz, $CDCl_3$) δ 7.39 – 7.29 (m, 4H), 7.27 – 7.19 (m, 1H), 3.66 (s, 2H), 0.19 (s, 9H).

$^{13}C\{^1H\}$ NMR: (101 MHz, $CDCl_3$) δ 136.5, 128.6, 128.0, 126.7, 104.4, 87.0, 26.3, 0.2.

$^{29}Si$ NMR: (79 MHz, $CDCl_3$) δ -18.7.

EI-MS m/z (rel. int.): 188 ($[M]^+$, 15%), 173 (100), 145 (20), 115 (45).

## *Tert*-butyldimethyl((3-(trimethylsilyl)prop-2-yn-1-yl)oxy)silane (**3ac**)

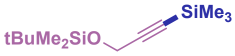


*Tert*-butyldimethyl((3-(trimethylsilyl)prop-2-yn-1-yl)oxy)silane was obtained in 71% yield as a pale-yellow oil (0.172 g). The title compound was known in the literature, and all spectroscopic data are in agreement.[1]

$^1H$ NMR: (400 MHz, $CDCl_3$) δ 4.31 (s, 2H), 0.91 (s, 9H), 0.16 (s, 9H), 0.12 (s, 6H).

$^{13}C\{^1H\}$ NMR: (101 MHz, $CDCl_3$) δ 105.0, 90.1, 52.8, 26.3, 26.3, 18.8, 0.2, -4.6, -4.7.

$^{29}Si$ NMR: (79 MHz, $CDCl_3$) δ 22.9, -18.1.

EI-MS m/z (rel. int.): 185 (45%), 155 (100), 73 (20), 57 (30).

## *N,N*-diethyl-3-(trimethylsilyl)prop-2-yn-1-amine (**3ad**)

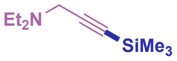


*N,N*-diethyl-3-(trimethylsilyl)prop-2-yn-1-amine was obtained in 77% yield as a pale-yellow oil (0.141 g). The title compound was known in the literature, and all spectroscopic data are in agreement.[5]

$^1H$ NMR: (400 MHz, $CDCl_3$) δ 3.39 (s, 2H), 2.51 (q, $J$ = 7.2 Hz, 4H), 1.04 (t, $J$ = 7.2 Hz, 6H), 0.14 (s, 9H).

$^{13}C\{^1H\}$ NMR: (101 MHz, $CDCl_3$) δ 101.4, 89.8, 47.5, 42.2, 13.0, 0.5.

$^{29}Si$ NMR: (79 MHz, $CDCl_3$) δ -18.7.

EI-MS m/z (rel. int.): 183 ([M]$^+$, 5%), 168 (100), 111 (10), 83 (15).

## (Oxybis(prop-1-yne-3,1-diyl))bis(trimethylsilane) (**3ae**)

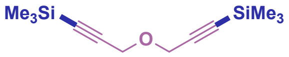


(Oxybis(prop-1-yne-3,1-diyl))bis(trimethylsilane) was obtained in 85% yield as a pale-yellow oil (0.202 g). The title compound was known in the literature, and all spectroscopic data are in agreement.[1]

$^1$H NMR: (400 MHz, $CDCl_3$) δ 4.23 (s, 4H), 0.17 (s, 18H).

$^{13}$C{$^1$H} NMR: (101 MHz, $CDCl_3$) δ 100.8, 92.1, 57.5, -0.1.

$^{29}$Si NMR: (79 MHz, $CDCl_3$) δ -17.7.

EI-MS m/z (rel. int.): 223 ([M - $CH_3$]$^+$, 10%), 193 (55), 83 (70), 73 (100).

## Trimethyl((4-((trimethylsilyl)ethynyl)benzyl)oxy)silane (**3af**)

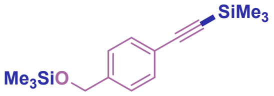


Trimethyl((4-((trimethylsilyl)ethynyl)benzyl)oxy)silane was obtained in 96% yield as a pale-yellow oil (0.265 g). The title compound was known in the literature, and all spectroscopic data are in agreement.

$^1$H NMR: (400 MHz, $CDCl_3$) δ 7.46 – 7.41 (m, 2H), 7.30 – 7.22 (m, 2H), 4.68 (s, 2H), 0.25 (s, 9H), 0.15 (s, 9H).

$^{13}$C{$^1$H} NMR: (101 MHz, $CDCl_3$) δ 141.7, 132.1, 126.3, 121.9, 105.3, 93.8, 64.4, 0.1, -0.3.

$^{29}$Si NMR: (79 MHz, $CDCl_3$) δ 19.6, -17.9.

EI-MS m/z (rel. int.): 276 ([M]$^+$, 40%), 261 (40), 187 (100), 73 (40).

## Trimethyl(3-phenyl-3-((trimethylsilyl)oxy)prop-1-yn-1-yl)silane (**3ag**)

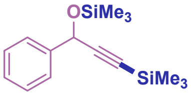


Trimethyl(3-phenyl-3-((trimethylsilyl)oxy)prop-1-yn-1-yl)silane was obtained in 78% yield as a pale-yellow oil (0.215 g). The title compound was known in the literature, and all spectroscopic data are in agreement.[14]

$^1$H NMR: (400 MHz, $CDCl_3$) δ 7.54 – 7.46 (m, 2H), 7.41 – 7.32 (m, 2H), 7.31 – 7.29 (m, 1H), 5.49 (s, 1H), 0.21 (s, 9H), 0.19 (s, 9H).

$^{13}$C{$^1$H} NMR: (101 MHz, $CDCl_3$) δ 141.3, 128.5, 127.9, 126.7, 106.1, 91.0, 65.2, 0.5, -0.1.

$^{29}$Si NMR: (79 MHz, $CDCl_3$) δ 20.4, -17.8.

EI-MS m/z (rel. int.): 276 ([M]$^+$, 25%), 155 (60), 114 (50), 73 (100).

## Trimethyl(3-(oxiran-2-ylmethoxy)prop-1-yn-1-yl)silane (**3ah**)

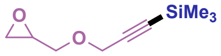


Trimethyl(3-(oxiran-2-ylmethoxy)prop-1-yn-1-yl)silane was obtained in 82% yield as a pale-yellow oil (0.151 g). The title compound was known in the literature, and all spectroscopic data are in agreement.[15]

$^1$H NMR: (400 MHz, $CDCl_3$) δ 4.33 – 4.08 (m, 2H), 3.90 – 3.70 (m, 1H), 3.56 – 3.28 (m, 1H), 3.26 – 3.07 (m, 1H), 2.86 – 2.72 (m, 1H), 2.68 – 2.55 (m, 1H), 0.16 (s, 9H).

$^{13}$C{$^1$H} NMR: (101 MHz, $CDCl_3$) δ 101.1, 91.9, 70.4, 59.4, 50.6, 44.5, 44.5, -0.1.

$^{29}Si$ NMR: (79 MHz, $CDCl_3$) δ -17.7.

EI-MS m/z (rel. int.): 169 ($[M-CH_3]^+$, 5%), 109 (60), 83 (100), 73 (85).

## Methyl 4-((trimethylsilyl)ethynyl)benzoate (**3ai**)

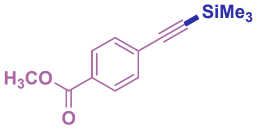


Methyl 4-((trimethylsilyl)ethynyl)benzoate was obtained in 88% yield as a pale-yellow oil (0.204 g). The title compound was known in the literature, and all spectroscopic data are in agreement.[2]

$^1H$ NMR: (400 MHz, $CDCl_3$) δ 8.14 – 7.74 (m, 2H), 7.68 – 7.37 (m, 2H), 3.91 (s, 3H), 0.26 (s, 9H).

$^{13}C\{^1H\}$ NMR: (101 MHz, $CDCl_3$) δ 167.0, 132.3, 130.2, 129.8, 128.2, 104.5, 98.2, 52.7, 0.3.

$^{29}Si$ NMR: (79 MHz, $CDCl_3$) δ -17.2.

EI-MS m/z (rel. int.): 232 ($[M]^+$, 20%), 217 (100), 201 (5), 143 (10).

## (1-(4-Ethynylphenyl)-2,2,2-trifluoroethoxy)trimethylsilane (**3aj**)

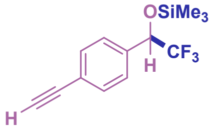


(1-(4-Ethynylphenyl)-2,2,2-trifluoroethoxy)trimethylsilane was obtained in 80% yield as a pale-yellow oil (0.218 g). The title compound was known in the literature, and all spectroscopic data are in agreement.[16]

$^1H$ NMR: (400 MHz, $CDCl_3$) δ 7.53 – 7.48 (m, 2H), 7.45 – 7.38 (m, 2H), 3.10 (s, 1H), 2.00 (s, 1H), 0.12 (s, 9H).

$^{13}C\{^1H\}$ NMR: (101 MHz, $CDCl_3$) δ 136.4, 132.4, 127.8, 123.3, 83.4, 78.2, 73.23 (q, $J$ = 32.3 Hz), -0.0.

$^{29}Si$ NMR: (79 MHz, $CDCl_3$) δ 23.7.

EI-MS m/z (rel. int.): 272 ($[M]^+$, 5%), 203 (60), 133 (100), 77 (40).

## *N*-benzyl-N-methyl-3-(trimethylsilyl)prop-2-yn-1-amine (**3ak**)

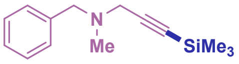


*N*-benzyl-N-methyl-3-(trimethylsilyl)prop-2-yn-1-amine was obtained in 85% yield as a pale-yellow oil (0.196 g). The title compound was known in the literature, and all spectroscopic data are in agreement.[1]

$^1H$ NMR: (400 MHz, $CDCl_3$) δ 7.35 – 7.31 (m, 4H), 7.30 – 7.26 (m, 1H), 3.58 (s, 2H), 3.32 (s, 2H), 2.35 (s, 3H), 0.23 (s, 9H).

$^{13}C\{^1H\}$ NMR: (101 MHz, $CDCl_3$) δ 138.5, 129.4, 128.4, 127.3, 101.1, 90.3, 60.2, 46.1, 42.0, 0.3.

$^{29}Si$ NMR: (79 MHz, $CDCl_3$) δ -18.5.

EI-MS m/z (rel. int.): 231 ($[M]^+$, 30%), 158 (30), 110 (25), 91 (100).

## *(R)*-*N*-(3-(trimethylsilyl)prop-2-yn-1-yl)-2,3-dihydro-1H-inden-1-amine (**3al**)

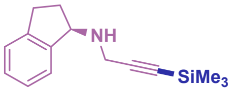


*(R)*-*N*-(3-(trimethylsilyl)prop-2-yn-1-yl)-2,3-dihydro-1H-inden-1-amine was obtained in 83% yield as a pale-yellow oil (0.202 g). The title compound was known in the literature, and all spectroscopic data are in agreement.[1]

$^1H$ NMR: (400 MHz, $CDCl_3$) δ 7.48 – 7.33 (m, 1H), 7.29 – 7.11 (m, 3H), 4.62 – 4.26 (m, 1H), 3.54 (s, 2H), 3.22 – 2.65 (m, 2H), 2.56 – 2.33 (m, 1H), 1.97 – 1.78 (m, 1H), 1.51 – 1.37 (m, 1H), 0.20 (s, 9H).

$^{13}C\{^1H\}$ NMR: (101 MHz, $CDCl_3$) δ 144.8, 144.0, 127.7, 126.4, 125.0, 124.4, 105.0, 88.0, 62.2, 37.5, 33.5, 30.6, 0.1.

$^{29}Si$ NMR: (79 MHz, $CDCl_3$) δ –18.4.

EI-MS m/z (rel. int.): 243 ($[M]^+$, 60%), 170 (100), 132 (60), 117 (95).

## Trimethyl(1-(*o*-tolyl)ethoxy)silane (**5a**)

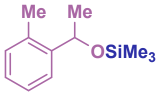


Trimethyl(1-(*o*-tolyl)ethoxy)silane was obtained in 78% yield as a pale-yellow oil (0.162 g). The title compound was known in the literature, and all spectroscopic data are in agreement.[17]

$^1H$ NMR: (400 MHz, $CDCl_3$) δ 7.54 (d, *J* = 7.7 Hz, 1H), 7.25 – 7.20 (m, 1H), 7.19 – 7.09 (m, 2H), 5.08 (qd, *J* = 6.4, 2.3 Hz, 1H), 2.35 (s, 3H), 1.42 (dd, *J* = 6.3, 2.1 Hz, 3H), 0.10 (s, 9H).

$^{13}C\{^1H\}$ NMR: (101 MHz, $CDCl_3$) δ 144.8, 133.2, 130.2, 126.7, 126.2, 125.6, 67.6, 25.8, 19.1, 0.2.

$^{29}Si$ NMR: (79 MHz, $CDCl_3$) δ 16.2.

EI-MS m/z (rel. int.): 193 ($[M-CH_3]^+$, 100%), 117 (30), 91 (30), 75 (55).

## (1-(3-Methoxyphenyl)ethoxy)trimethylsilane (**5b**)

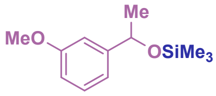


(1-(3-Methoxyphenyl)ethoxy)trimethylsilane was obtained in 84% yield as a pale-yellow oil (0.188 g). The title compound was known in the literature, and all spectroscopic data are in agreement.[17]

$^1H$ NMR: (400 MHz, $CDCl_3$) δ 7.24 (td, *J* = 7.9, 1.5 Hz, 1H), 6.99 – 6.88 (m, 2H), 6.79 (dd, *J* = 8.2, 2.4 Hz, 1H), 4.85 (dd, *J* = 6.4, 2.3 Hz, 1H), 3.82 (s, 3H), 1.45 (d, *J* = 6.5 Hz, 3H), 0.11 (s, 9H).

$^{13}C\{^1H\}$ NMR: (101 MHz, $CDCl_3$) δ 159.7, 148.4, 129.2, 117.9, 112.3, 111.1, 70.6, 55.3, 27.0, 0.2.

$^{29}Si$ NMR: (79 MHz, $CDCl_3$) δ 16.8.

EI-MS m/z (rel. int.): 209 ($[M-CH_3]^+$, 5%), 193 (100), 91 (40), 75 (50).

## (1-(4-Chlorophenyl)ethoxy)trimethylsilane (**5c**)

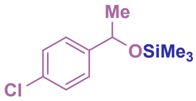


(1-(4-Chlorophenyl)ethoxy)trimethylsilane was obtained in 84% yield as a pale-yellow oil (0.192 g). The title compound was known in the literature, and all spectroscopic data are in agreement.[17]

$^1H$ NMR: (400 MHz, $CDCl_3$) δ 7.32 – 7.26 (m, 4H), 4.83 (q, *J* = 6.4 Hz, 1H), 1.41 (dd, *J* = 6.4, 0.8 Hz, 3H), 0.08 (s, 9H).

$^{13}C\{^1H\}$ NMR: (101 MHz, $CDCl_3$) δ 145.2, 132.6, 128.4, 126.9, 70.1, 27.0, 0.2.

$^{29}Si$ NMR: (79 MHz, $CDCl_3$) δ 17.1.

EI-MS m/z (rel. int.): 213 ($[M-CH_3]^+$, 95%), 139 (35), 103 (40), 75 (100).

## 1,1,1-Trimethyl-3,3,3-tripropyldisiloxane (**5d**)

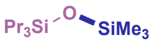

1,1,1-Trimethyl-3,3,3-tripropyldisiloxane was obtained in 79% yield as a pale-yellow oil (0.194 g). The title compound was known in the literature, and all spectroscopic data are in agreement.[9]

$^1$H NMR: (400 MHz, $CDCl_3$) δ 1.40 – 1.26 (m, 6H), 0.95 (t, *J* = 7.3 Hz, 9H), 0.57 – 0.44 (m, 6H), 0.06 (s, 9H).

$^{13}$C{$^1$H} NMR: (101 MHz, $CDCl_3$) δ 18.6, 18.5, 17.0, 2.1.

$^{29}$Si NMR: (79 MHz, $CDCl_3$) δ 6.3, 6.1.

EI-MS m/z (rel. int.): 203 (15%), 161 (65), 191 (100), 73 (25).

## 1,1,1-Trimethyl-3,3,3-triisopropyldisiloxane (**5e**)

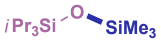


1,1,1-Trimethyl-3,3,3-triisopropyldisiloxane was obtained in 81% yield as a pale-yellow oil (0.199 g). The title compound was known in the literature, and all spectroscopic data are in agreement.[18]

$^1$H NMR: (400 MHz, $CDCl_3$) δ 1.20 – 1.09 (m, 21H), 0.13 (s, 9H).

$^{13}$C{$^1$H} NMR: (101 MHz, $CDCl_3$) δ 17.7, 12.5, 1.9.

$^{29}$Si NMR: (79 MHz, $CDCl_3$) δ 6.5, 5.1.

EI-MS m/z (rel. int.): 246 ($[M]^+$, 5%), 232 (100), 205 (20), 149 (5).

## 1-(*Tert*-butyl)-3,3,3-trimethyl-1,1-diphenyldisiloxane (**5f**)

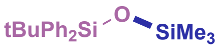


1-(*Tert*-butyl)-3,3,3-trimethyl-1,1-diphenyldisiloxane was obtained in 85% yield as a pale-yellow oil (0.279 g). The title compound was known in the literature, and all spectroscopic data are in agreement.[19]

$^1$H NMR: (400 MHz, $CDCl_3$) δ 7.74 – 7.62 (m, 4H), 7.53 – 7.34 (m, 6H), 1.09 (d, *J* = 0.9 Hz, 9H), 0.19 (s, 9H).

$^{13}$C{$^1$H} NMR: (101 MHz, $CDCl_3$) δ 135.9, 134.8, 129.1, 127.3, 26.5, 19.0, 2.0.

$^{29}$Si NMR: (79 MHz, $CDCl_3$) δ 8.8, -12.6.

EI-MS m/z (rel. int.): 313 ($[M\text{-}CH_3]^+$, 5%), 271 (95), 193 (85), 57 (100).

## (Cyclopropyl(phenyl)methoxy)trimethylsilane (**5g**)

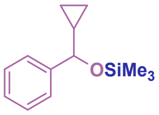


(Cyclopropyl(phenyl)methoxy)trimethylsilane was obtained in 91% yield as a pale-yellow oil (0.200 g). The title compound was known in the literature, and all spectroscopic data are in agreement.[20]

$^1$H NMR: (400 MHz, $CDCl_3$) δ 7.24 – 7.16 (m, 4H), 7.15 – 7.08 (m, 1H), 4.34 – 3.84 (m, 1H), 1.12 – 0.95 (m, 1H), 0.54 – 0.15 (m, 4H), -0.07 (s, 9H).

$^{13}$C{$^1$H} NMR: (101 MHz, $CDCl_3$) δ 145.3, 128.2, 127.1, 126.1, 78.3, 20.1, 3.7, 2.9, 0.4.

$^{29}$Si NMR: (79 MHz, $CDCl_3$) δ 16.7.

EI-MS m/z (rel. int.): 205 ($[M\text{-}CH_3]^+$, 5%), 192 (100), 91 (50), 73 (75).

SPECTRA FOR ALL PRODUCTS

Trimethyl(phenylethynyl)silane (**3a**)

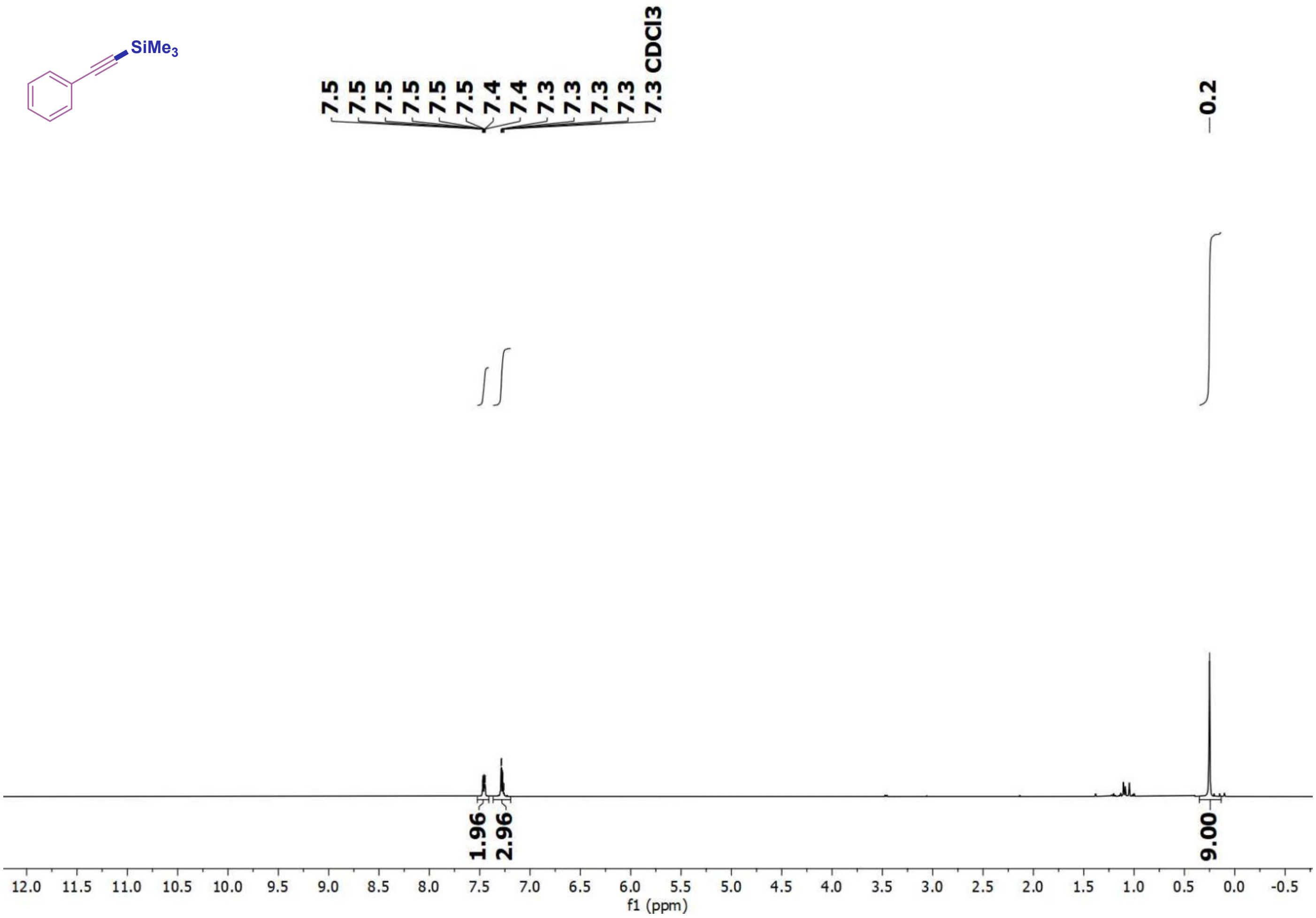


Figure S1. $^{1}H$ NMR (400 MHz, Chloroform-d, 25°C) of trimethyl(phenylethynyl)silane (**3a**).

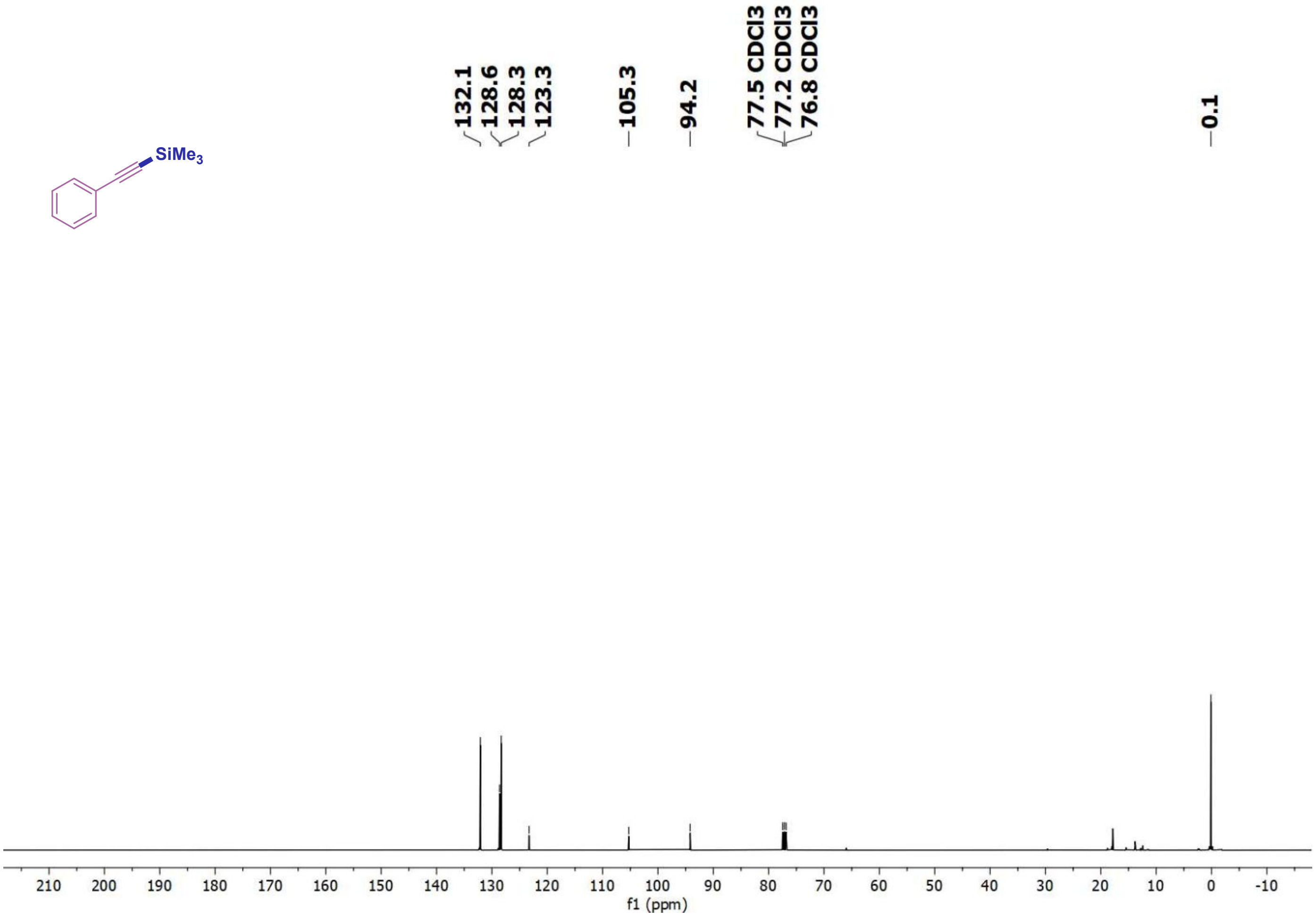


Figure S2. $^{13}C\{^{1}H\}$ NMR (101 MHz, Chloroform-d, 25°C) of trimethyl(phenylethynyl)silane (**3a**).

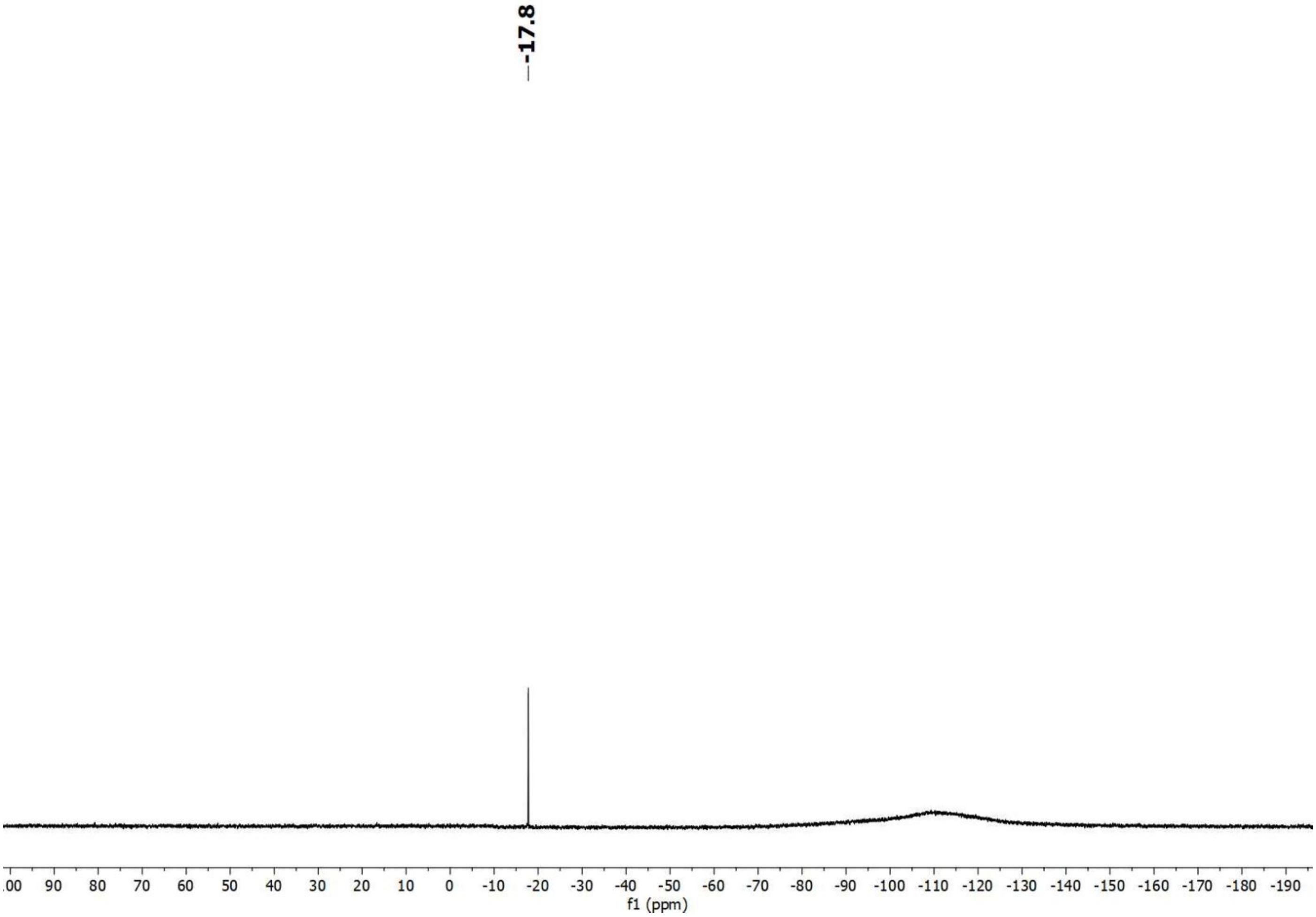


Figure S3. $^{29}$Si NMR (79 MHz, Chloroform-d, 25°C) of trimethyl(phenylethynyl)silane (**3a**).

Trimethyl((4-propylphenyl)ethynyl)silane (**3b**)

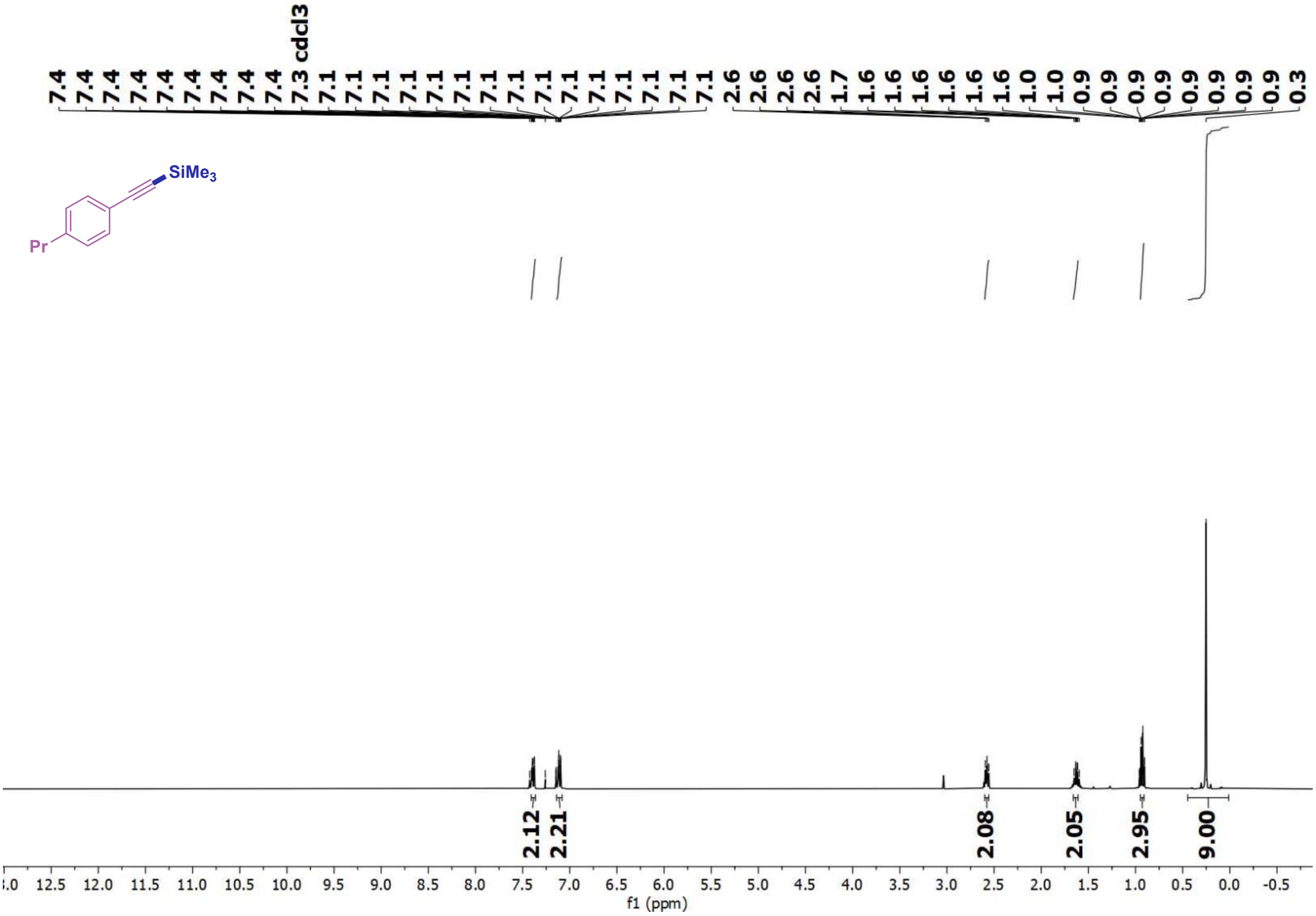

Figure S4. $^{1}$H NMR (400 MHz, Chloroform-d, 25°C) of trimethyl((4-propylphenyl)ethynyl)silane (**3b**).

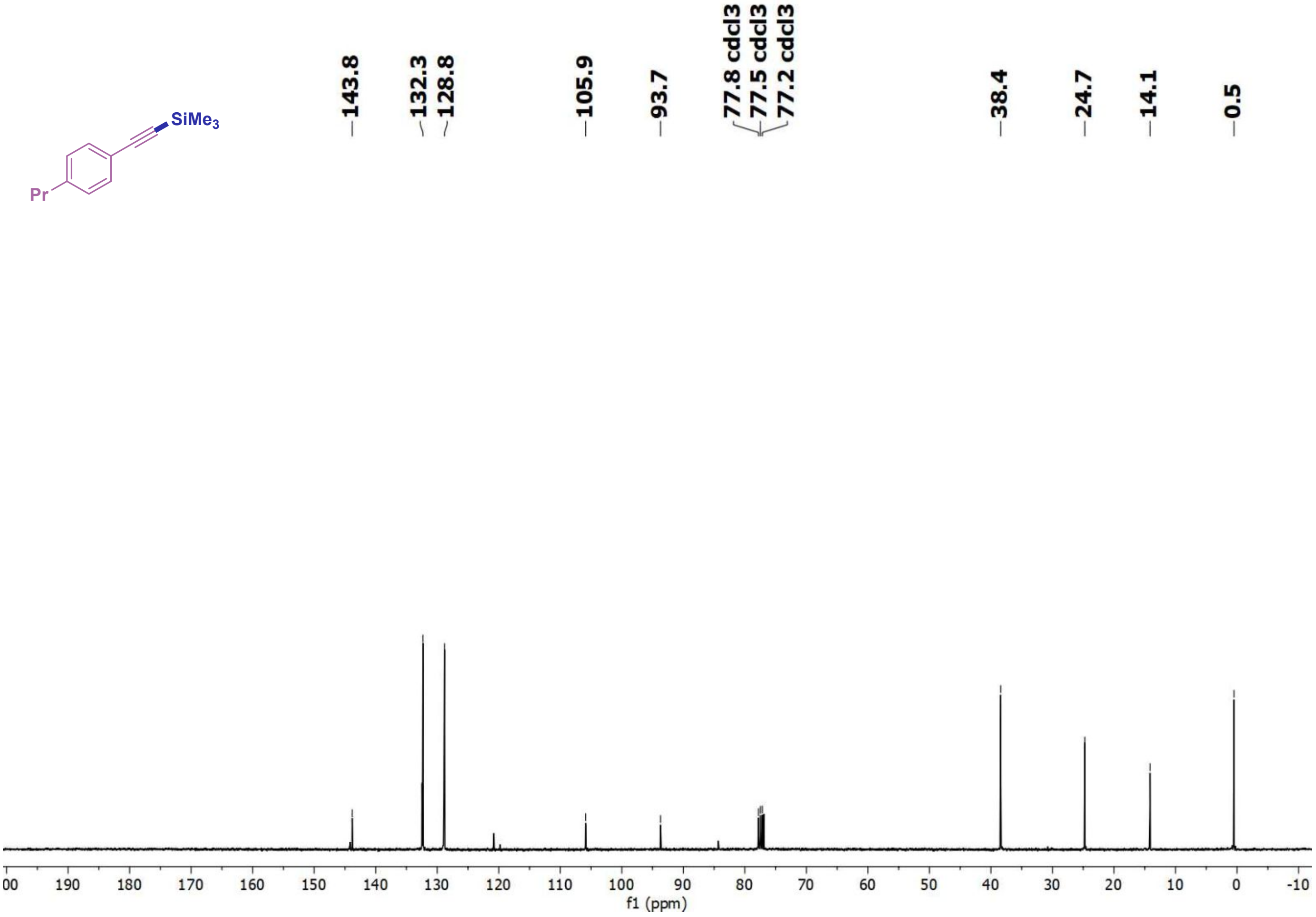

Figure S5. $^{13}$C{$^{1}$H} NMR (101 MHz, Chloroform-d, 25°C) of trimethyl((4-propylphenyl)ethynyl)silane (**3b**).

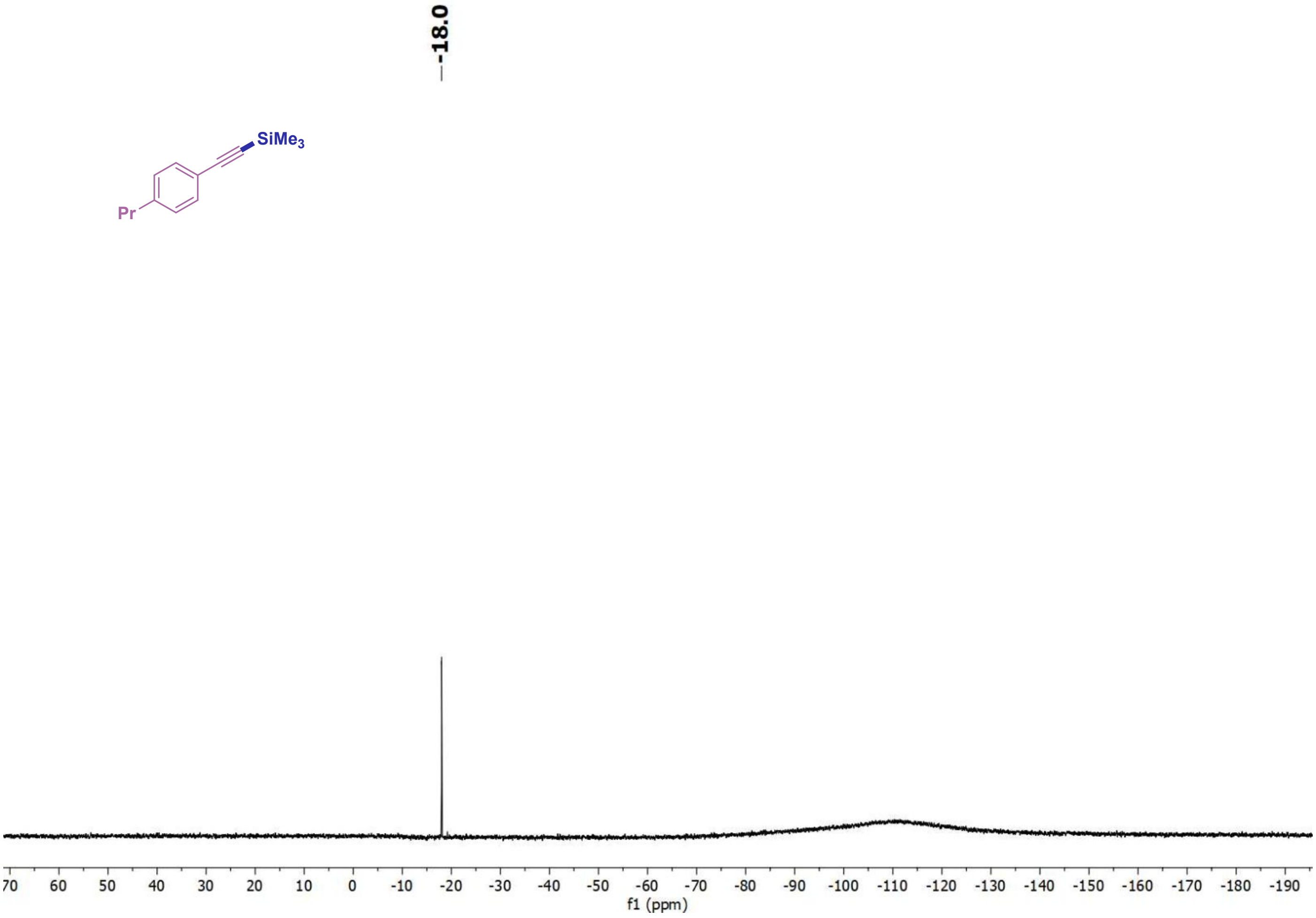


Figure S6. $^{29}Si$ NMR (79 MHz, Chloroform-d, 25°C) of trimethyl((4-propylphenyl)ethynyl)silane (**3b**).

([1,1'-Biphenyl]-4-ylethynyl)trimethylsilane (**3c**)

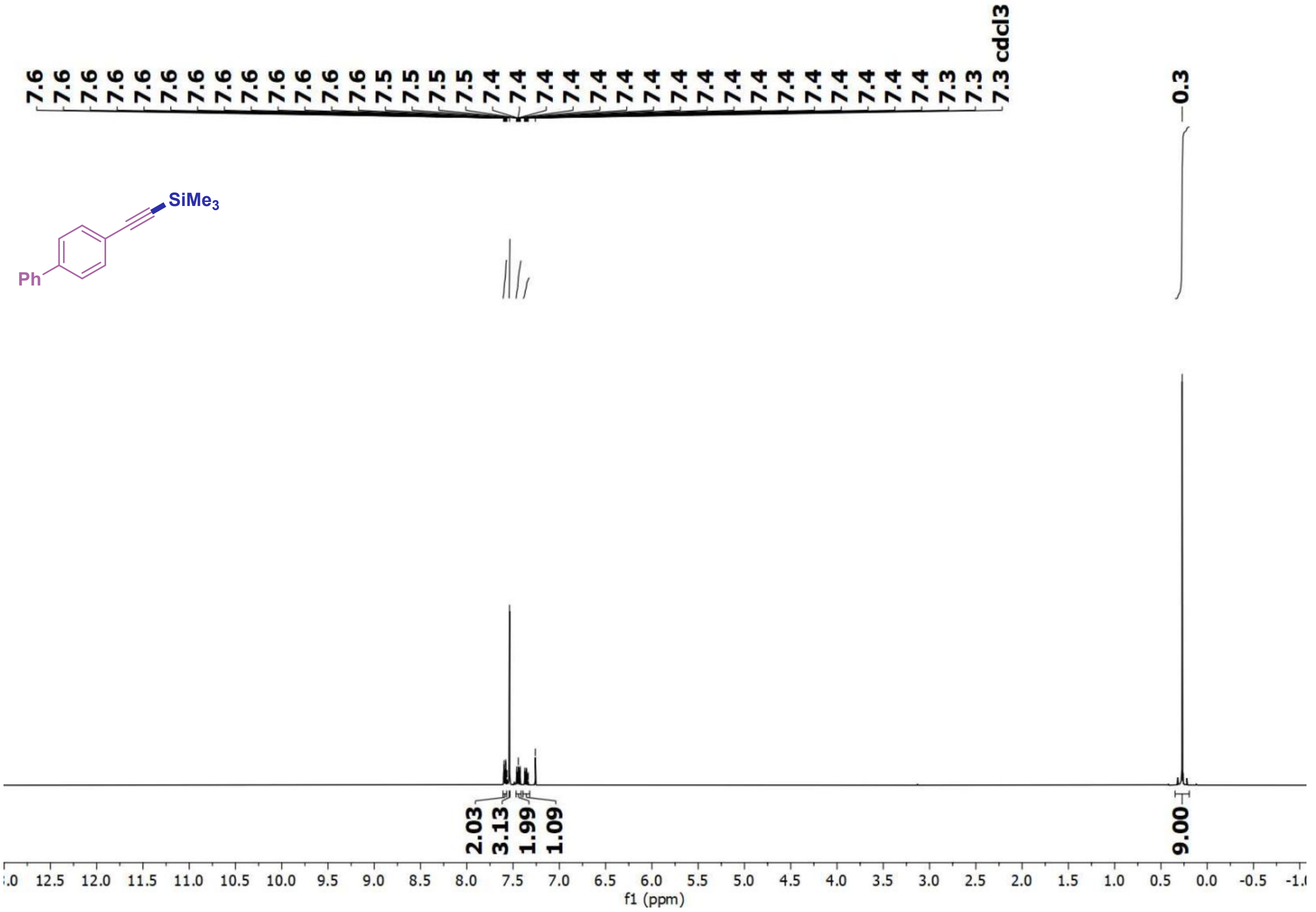

Figure S7. $^{1}H$ NMR (400 MHz, Chloroform-d, 25°C) of ([1,1'-biphenyl]-4-ylethynyl)trimethylsilane (**3c**).

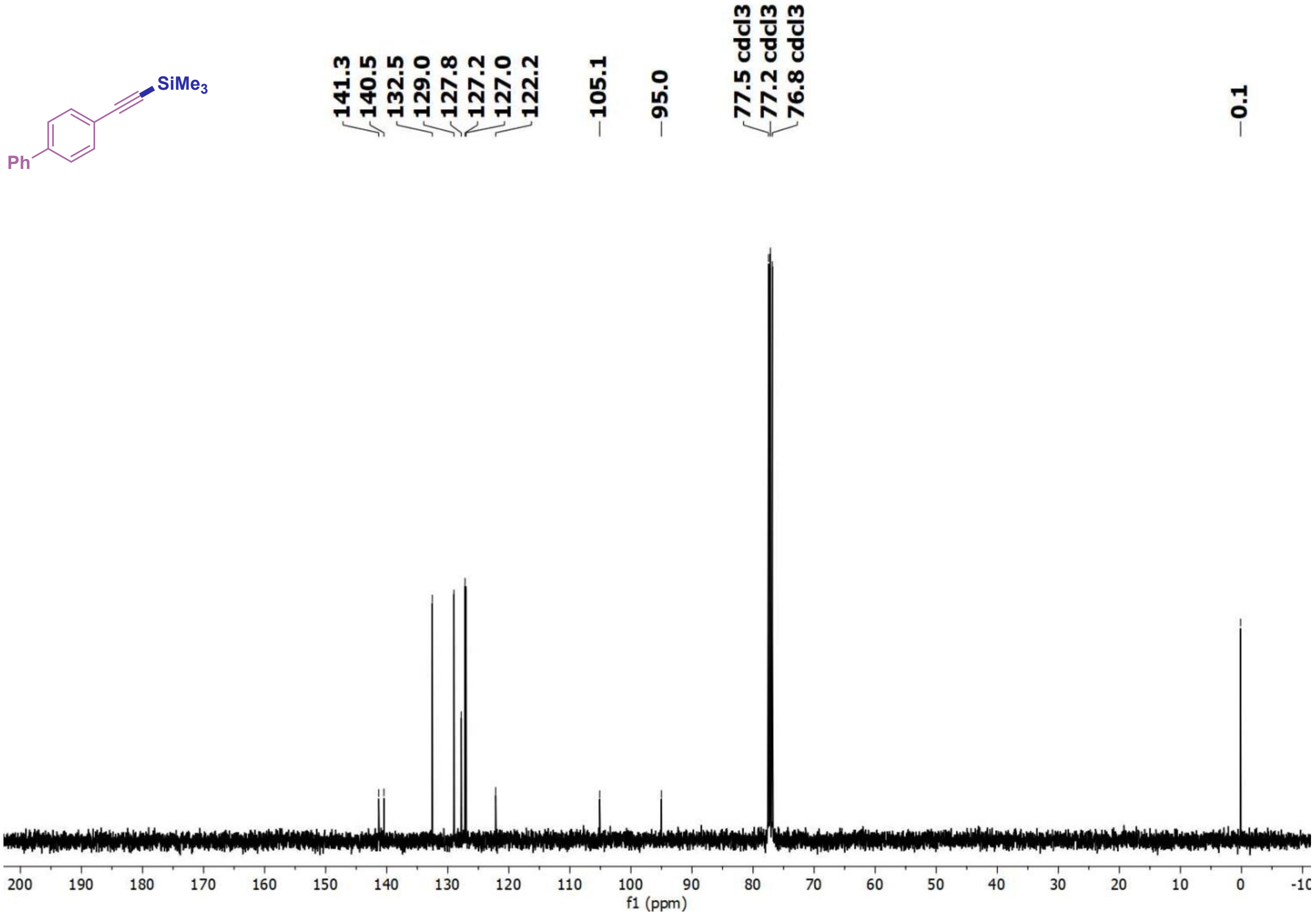

Figure S8. $^{13}C\{^{1}H\}$ NMR (101 MHz, Chloroform-d, 25°C) of ([1,1'-biphenyl]-4-ylethynyl)trimethylsilane (**3c**).

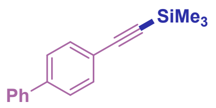


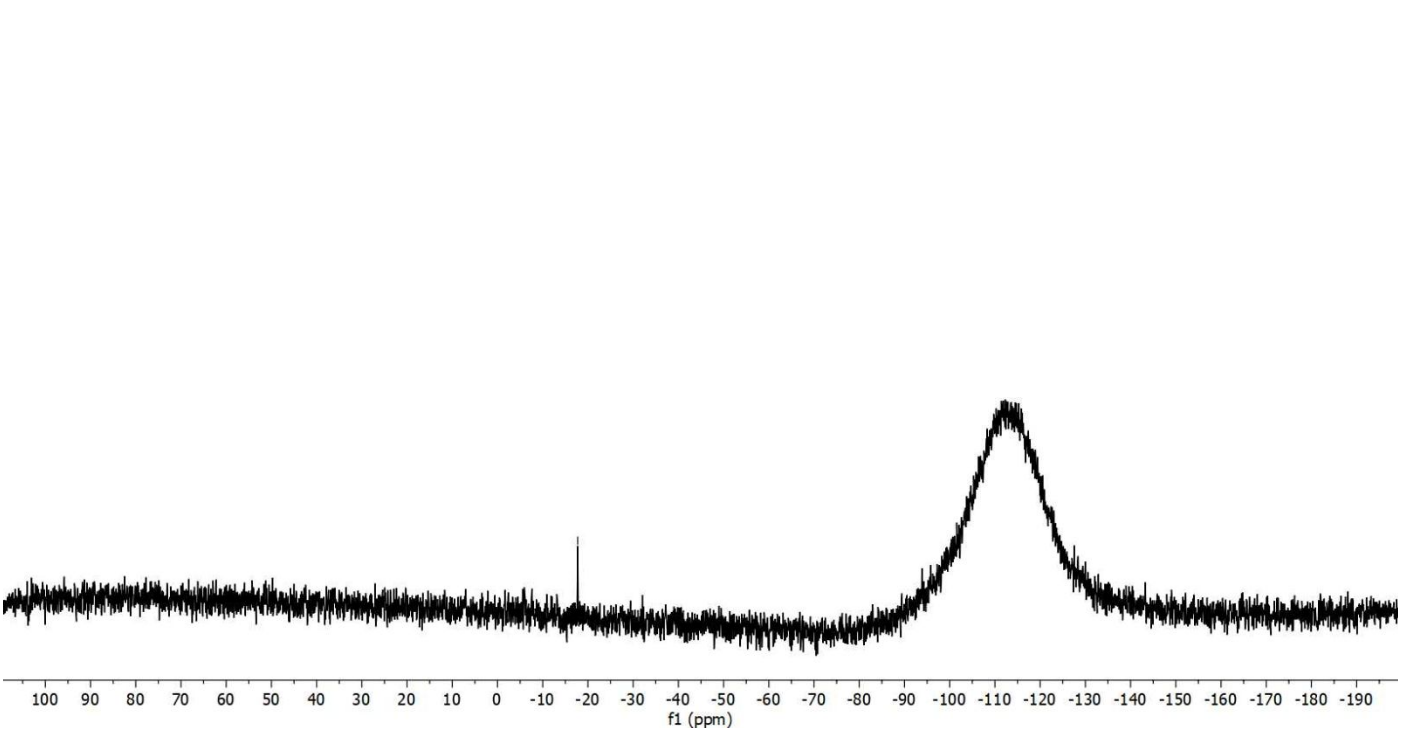


Figure S9. $^{29}Si$ NMR (79 MHz, Chloroform-d, 25°C) of ([1,1'-biphenyl]-4-ylethynyl)trimethylsilane (**3c**).

((4-Ethoxyphenyl)ethynyl)trimethylsilane (**3d**)

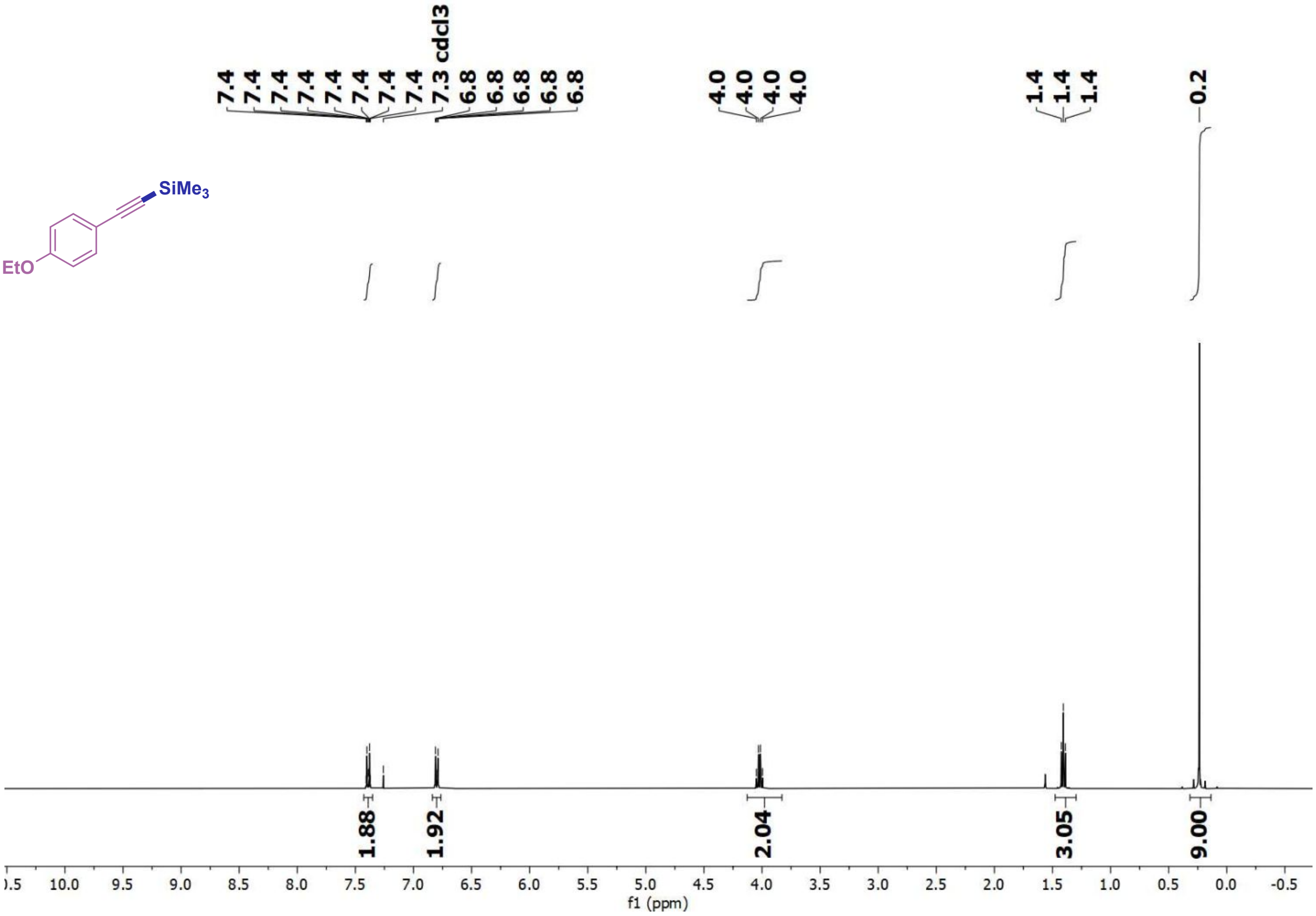


Figure S10. $^{1}H$ NMR (400 MHz, Chloroform-d, 25°C) of ((4-ethoxyphenyl)ethynyl)trimethylsilane (**3d**).

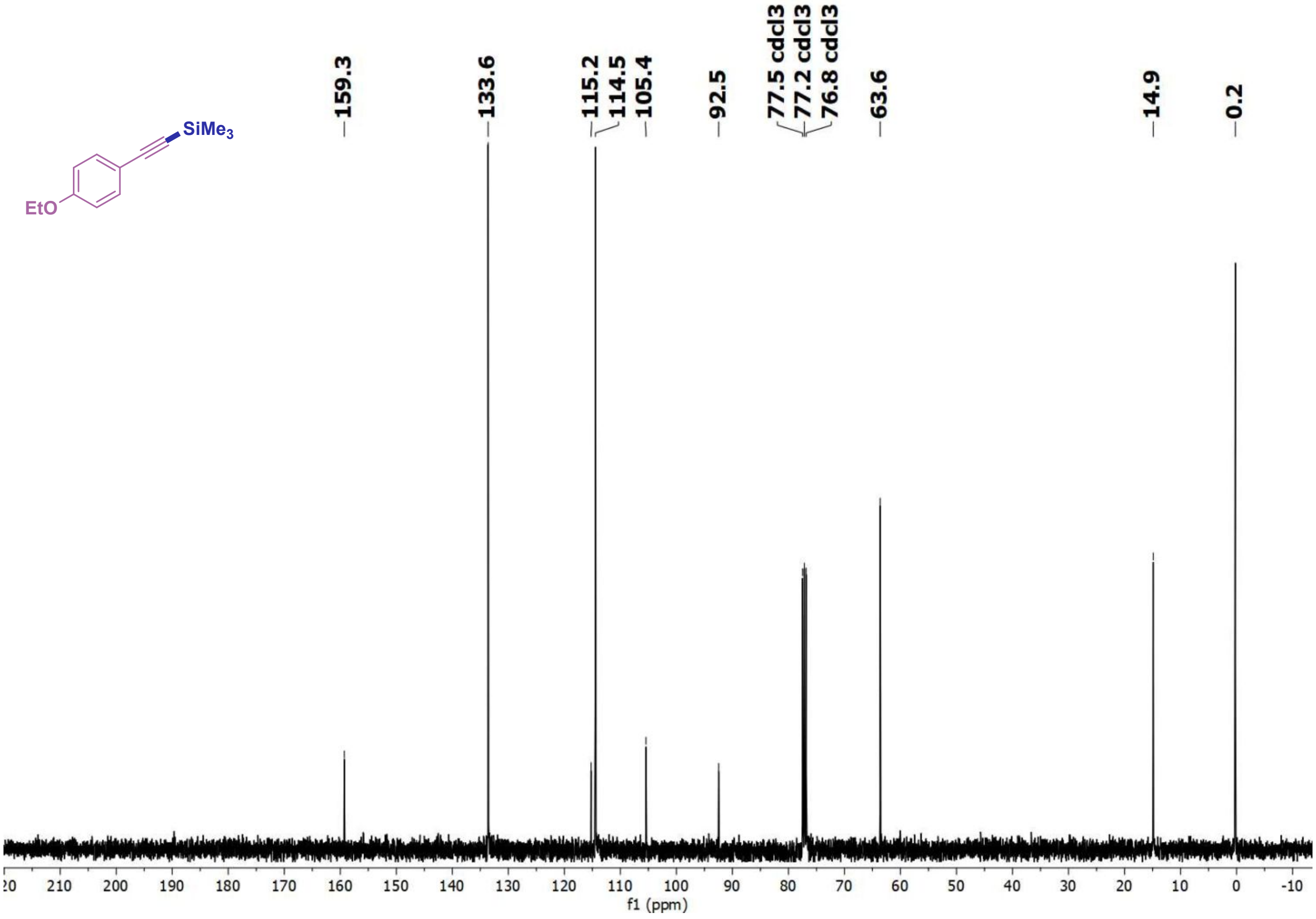


Figure S11. $^{13}C\{^{1}H\}$ NMR (101 MHz, Chloroform-d, 25°C) of ((4-ethoxyphenyl)ethynyl)trimethylsilane (**3d**).

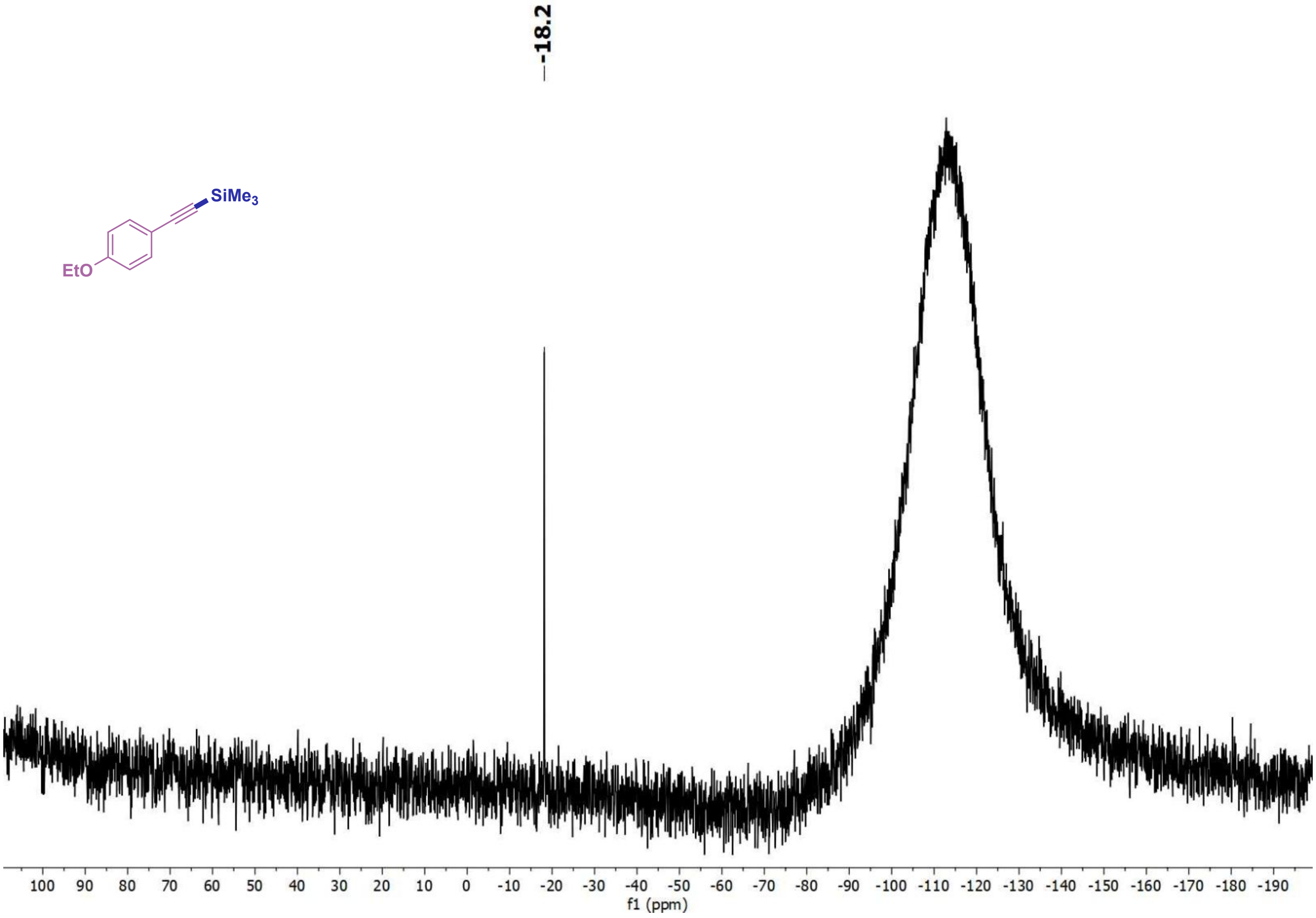


Figure S12. $^{29}Si$ NMR (79 MHz, Chloroform-d, 25°C) of ((4-ethoxyphenyl)ethynyl)trimethylsilane (**3d**).

# ((2-Methoxyphenyl)ethynyl)trimethylsilane (**3e**)

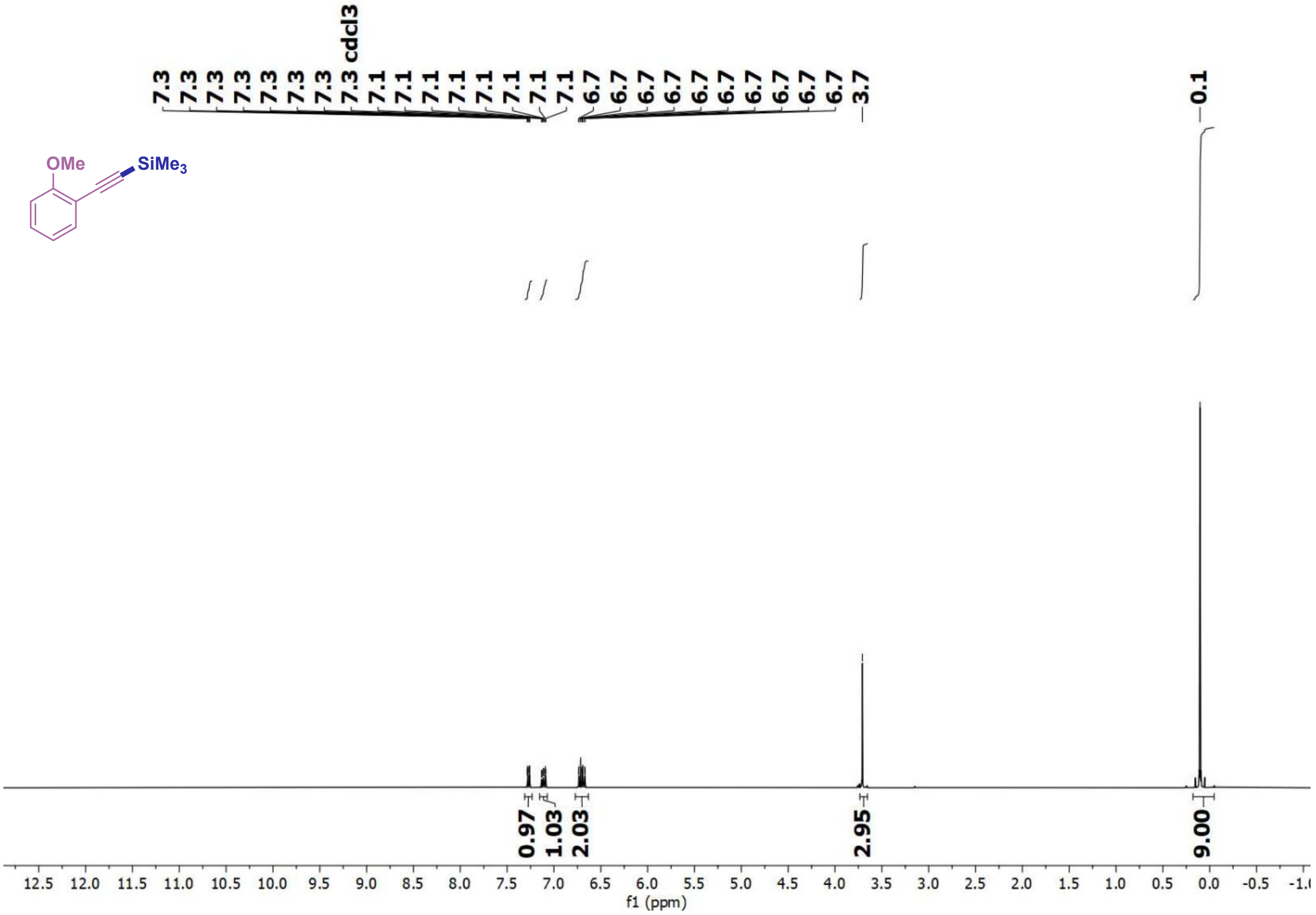


Figure S13. $^{1}H$ NMR (400 MHz, Chloroform-d, 25°C) of ((2-methoxyphenyl)ethynyl)trimethylsilane (**3e**).

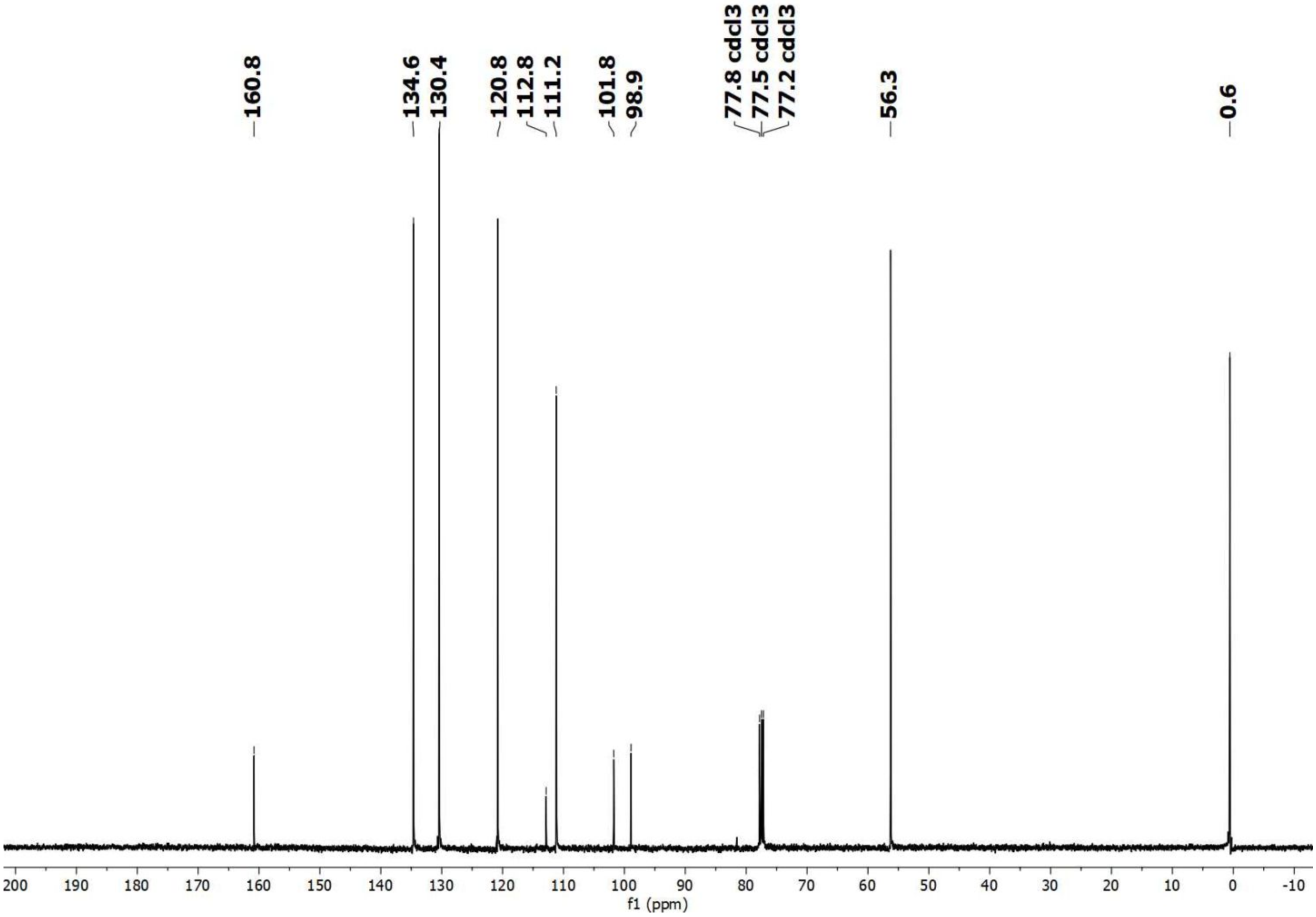


Figure S14. $^{13}C\{^{1}H\}$ NMR (101 MHz, Chloroform-d, 25°C) of ((2-methoxyphenyl)ethynyl)trimethylsilane (**3e**).

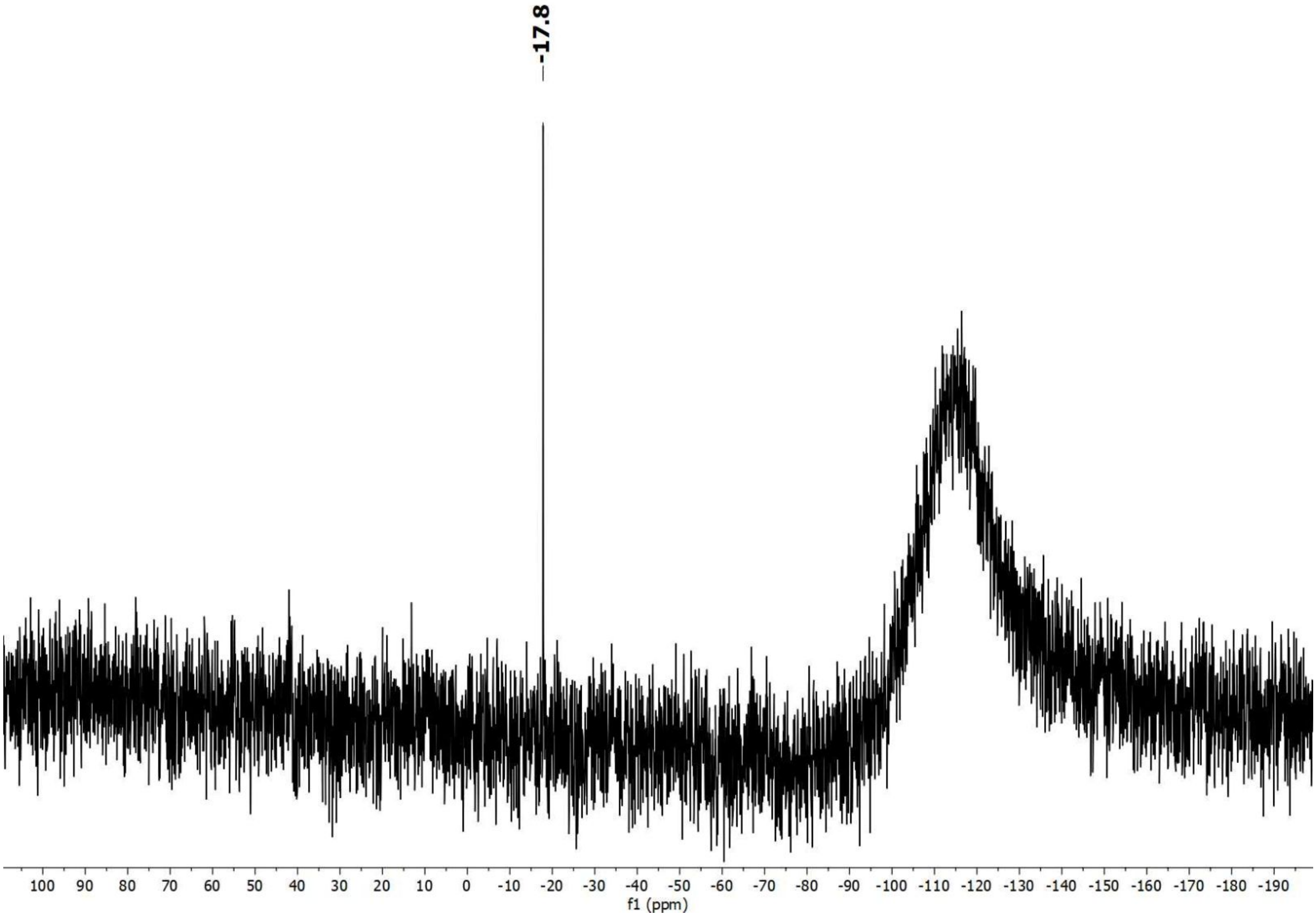


Figure S15. $^{29}Si$ NMR (79 MHz, Chloroform-d, 25°C) of ((2-methoxyphenyl)ethynyl)trimethylsilane (**3e**).

# ((3-Methoxyphenyl)ethynyl)trimethylsilane (**3f**)

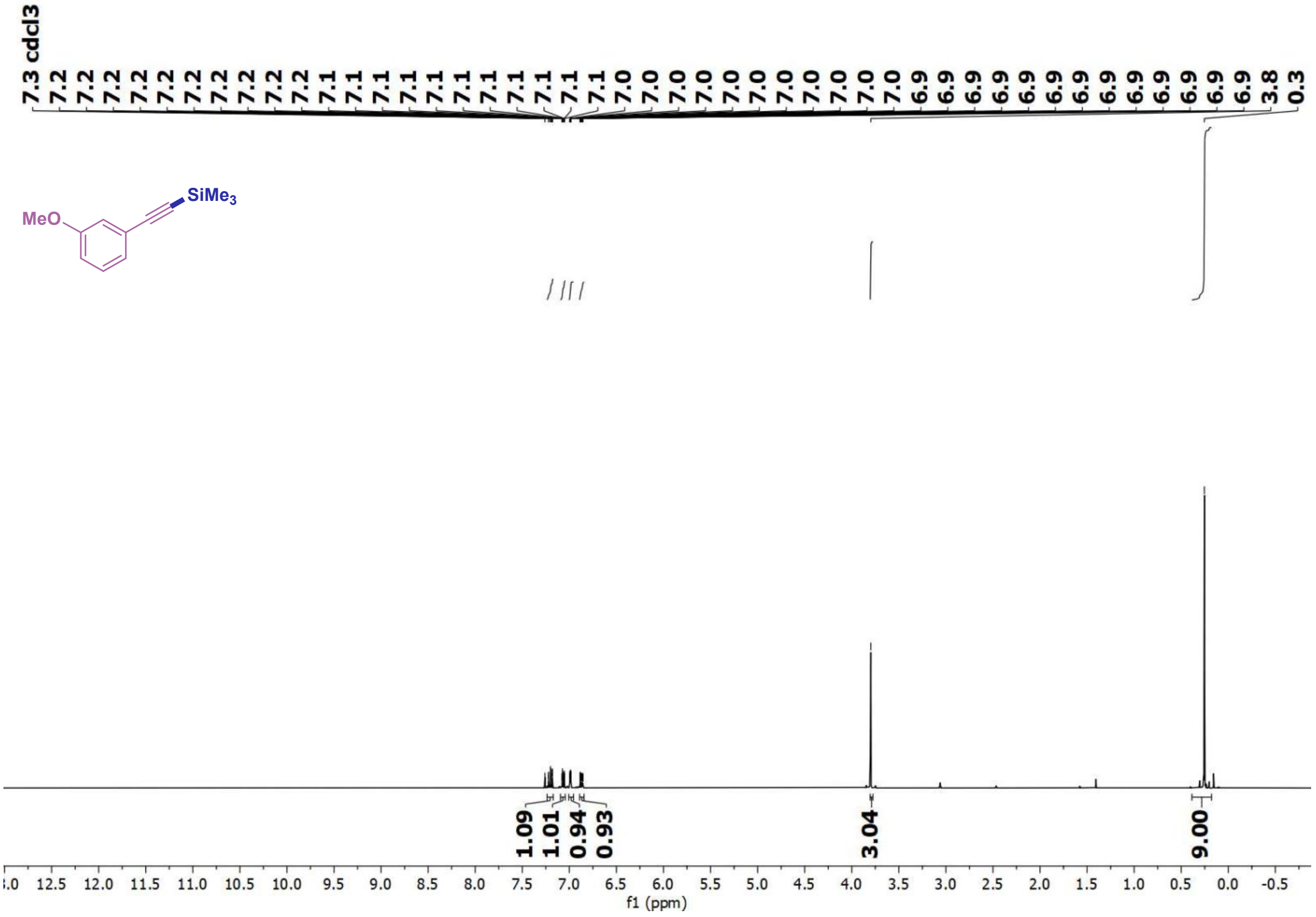


Figure S16. $^{1}$H NMR (400 MHz, Chloroform-d, 25°C) of ((3-methoxyphenyl)ethynyl)trimethylsilane (**3f**)

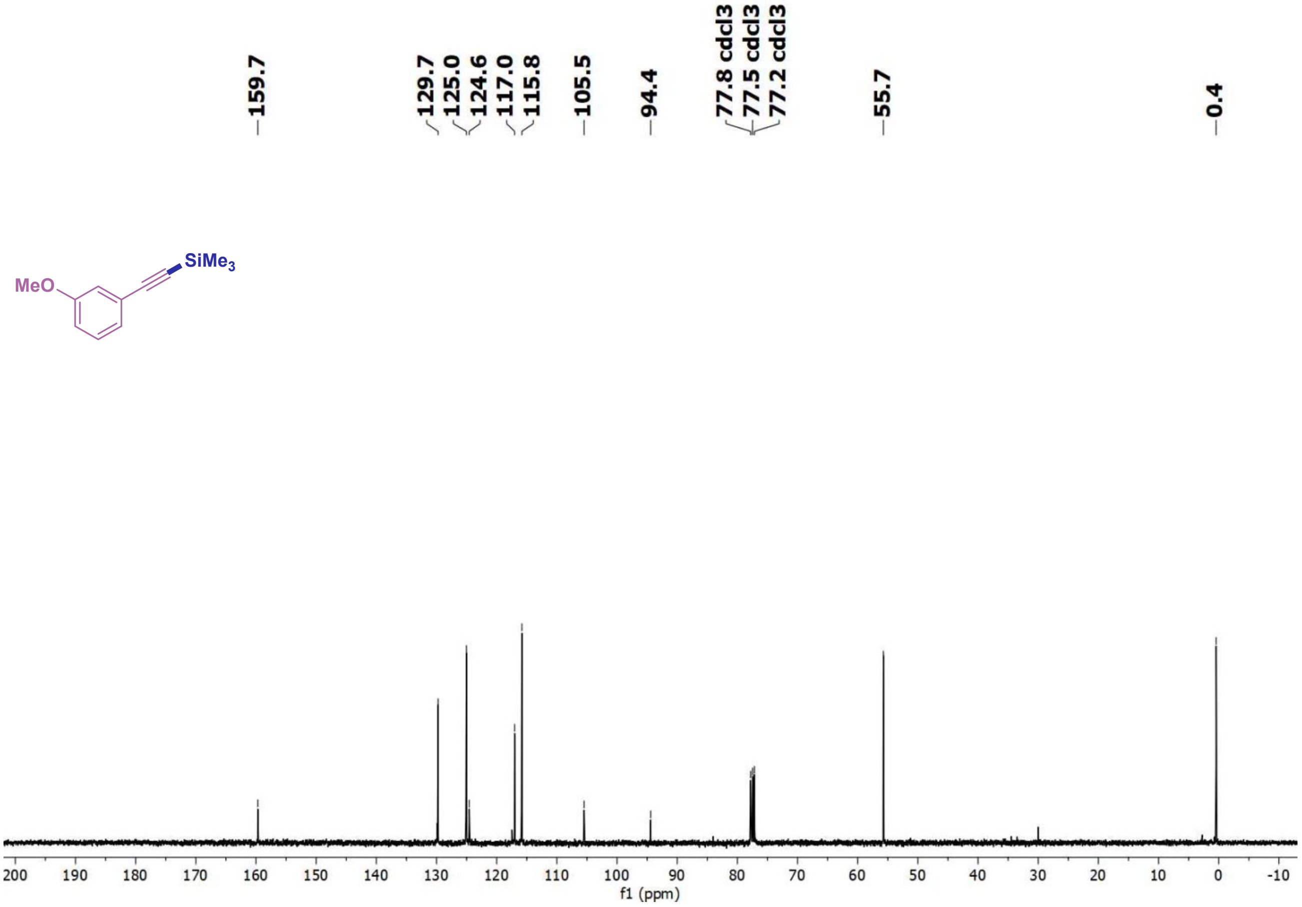


Figure S17. $^{13}$C{$^{1}$H} NMR (101 MHz, Chloroform-d, 25°C) of ((3-methoxyphenyl)ethynyl)trimethylsilane (**3f**)

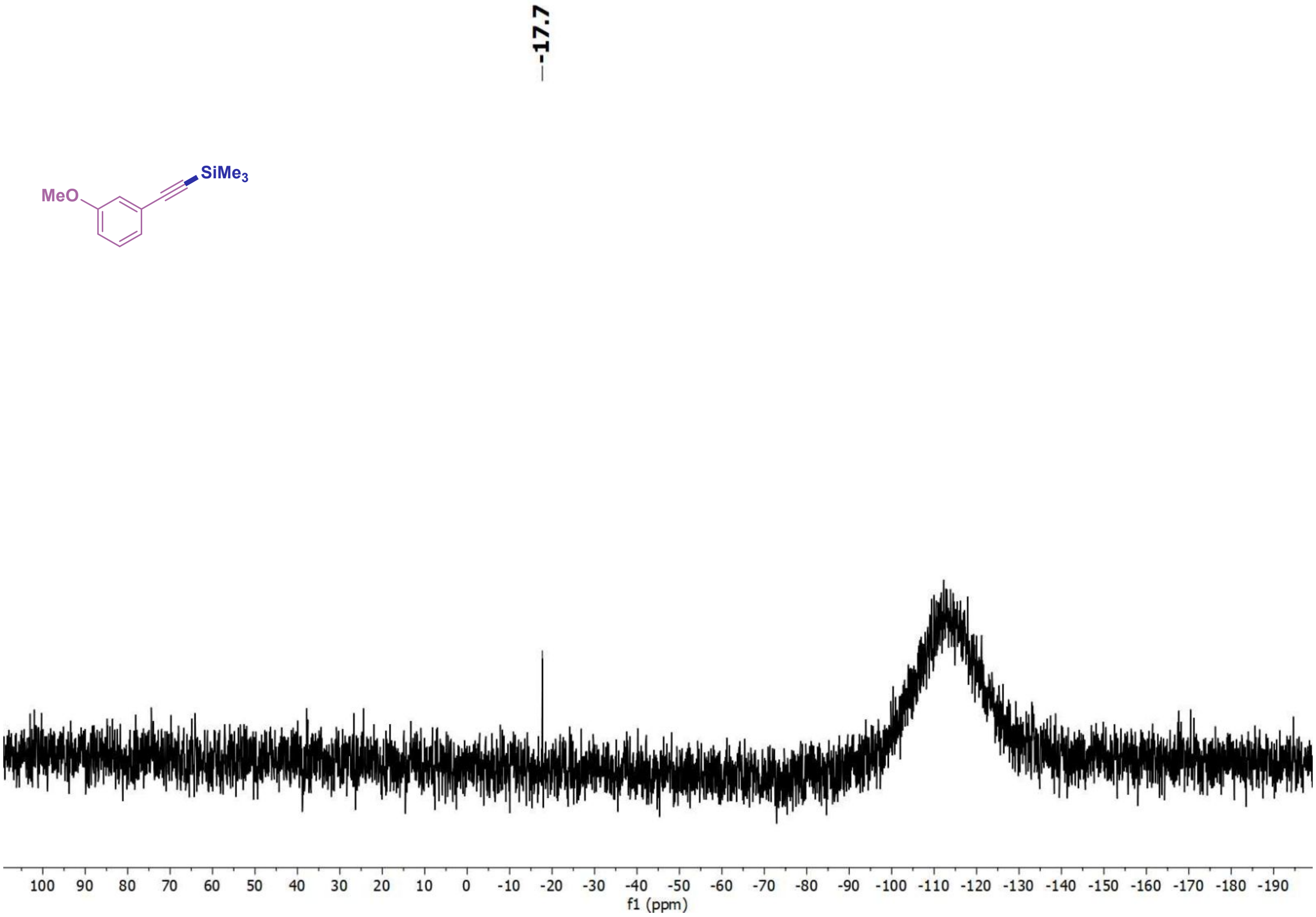


Figure S18. $^{29}Si$ NMR (79 MHz, Chloroform-d, 25°C) of ((3-methoxyphenyl)ethynyl)trimethylsilane (**3f**)

## ((4-Methoxyphenyl)ethynyl)trimethylsilane (**3g**)

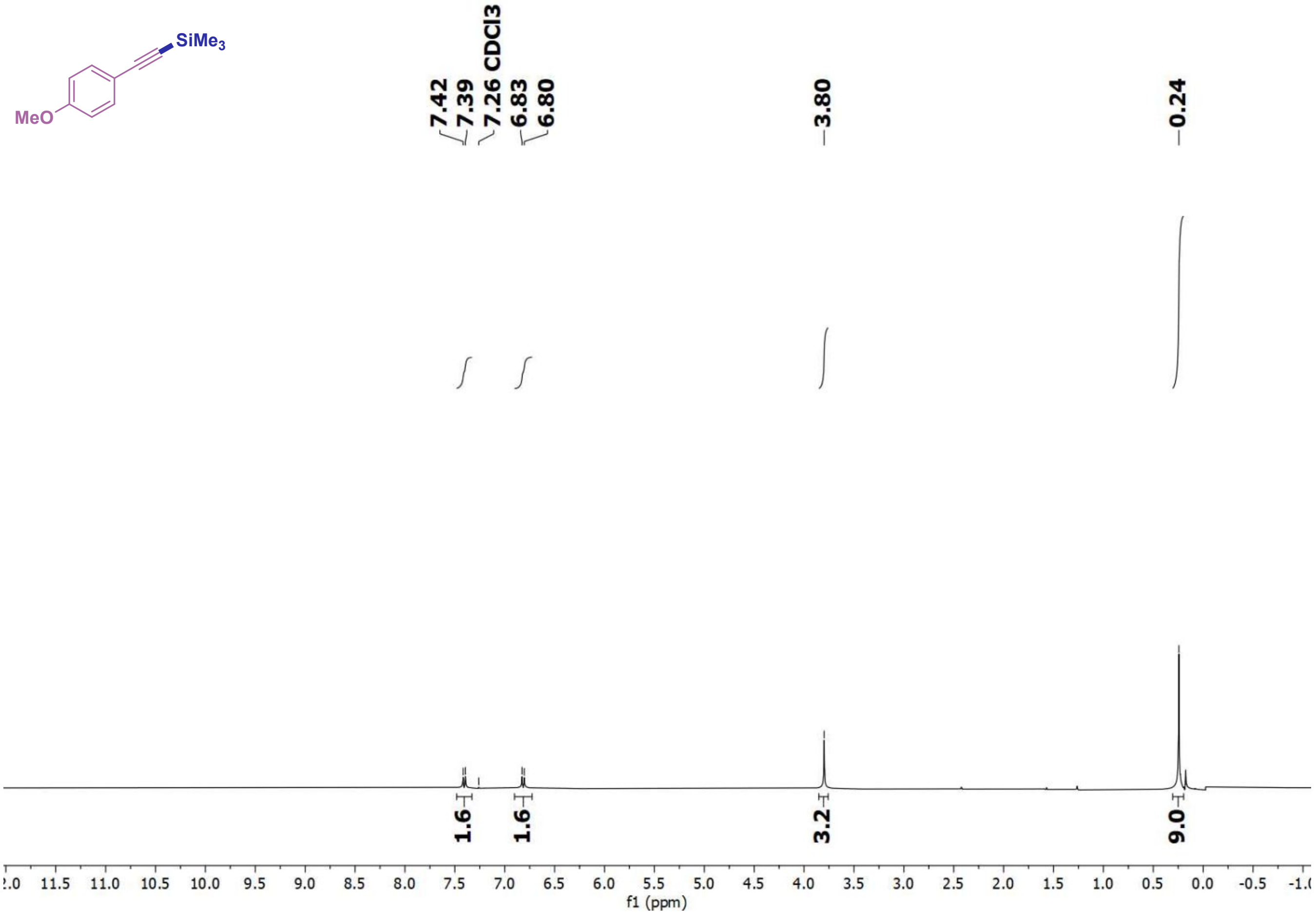


Figure S19. $^{1}$H NMR (400 MHz, Chloroform-d, 25°C) of ((4-methoxyphenyl)ethynyl)trimethylsilane (**3g**)

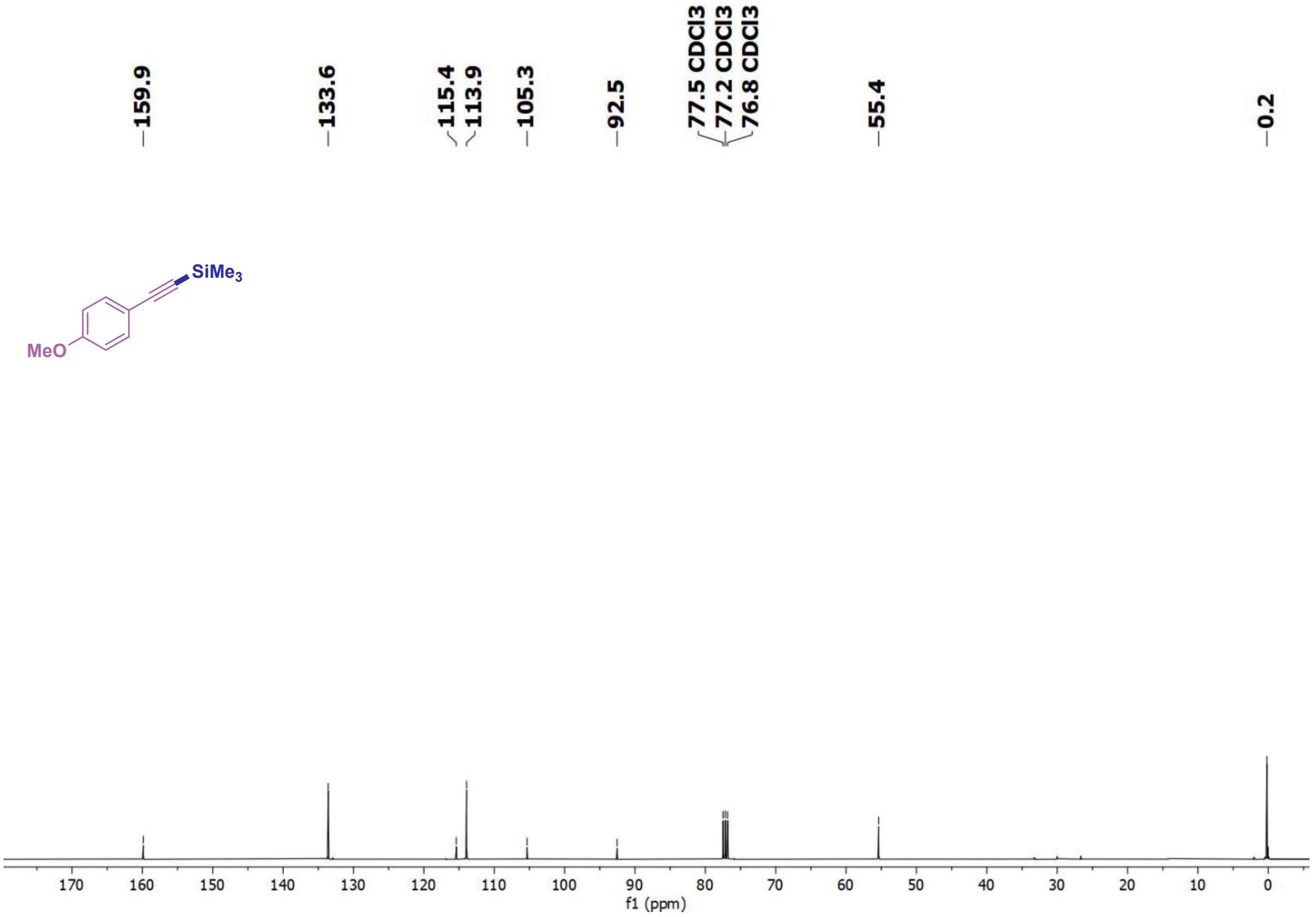


Figure S20. $^{13}C\{^{1}H\}$ NMR (101 MHz, Chloroform-d, 25°C) of ((4-methoxyphenyl)ethynyl)trimethylsilane (**3g**)

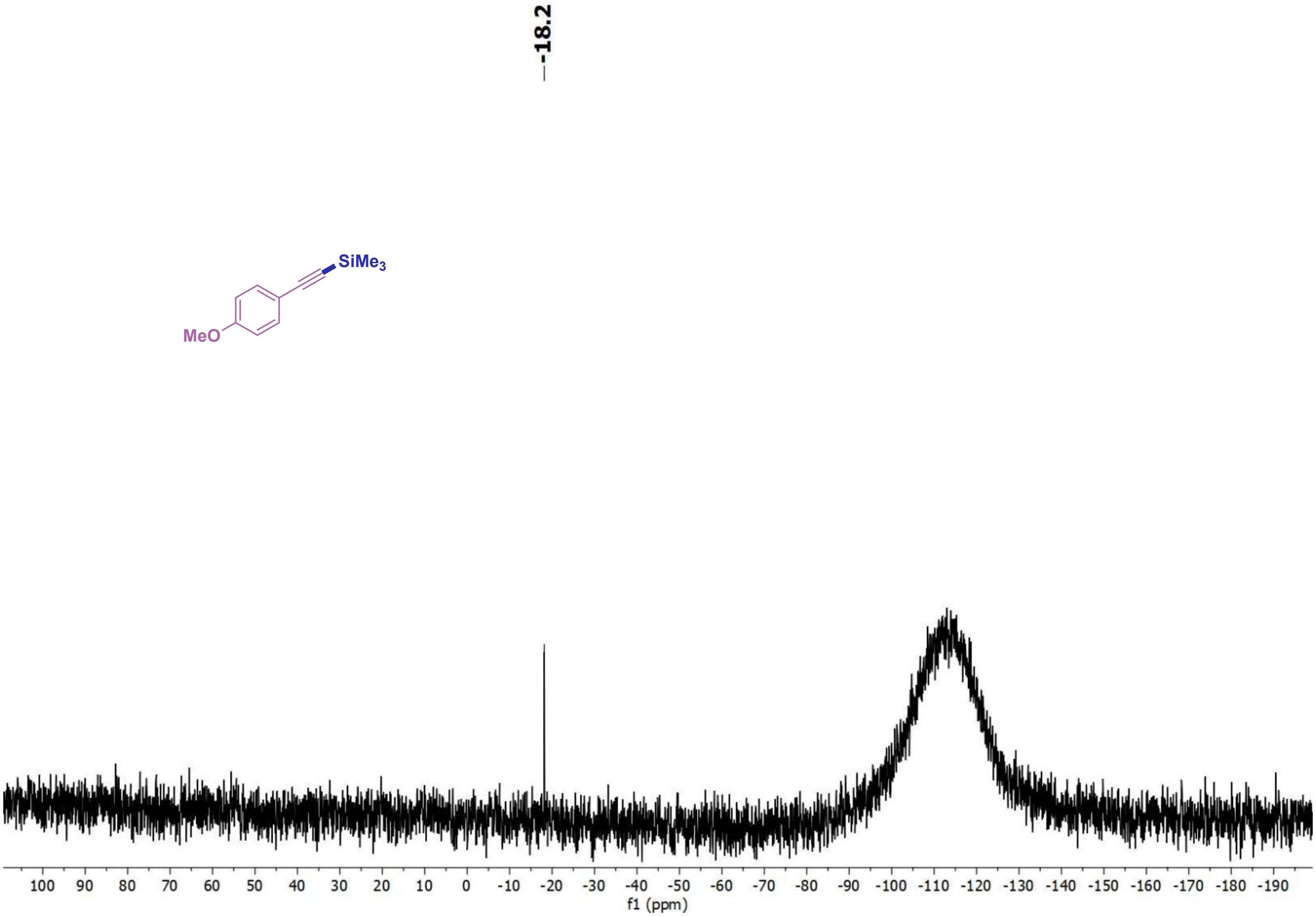


Figure S21. $^{29}Si$ NMR (79 MHz, Chloroform-d, 25°C) of ((4-methoxyphenyl)ethynyl)trimethylsilane (**3g**).

Trimethyl(naphthalen-1-ylethynyl)silane (**3h**)

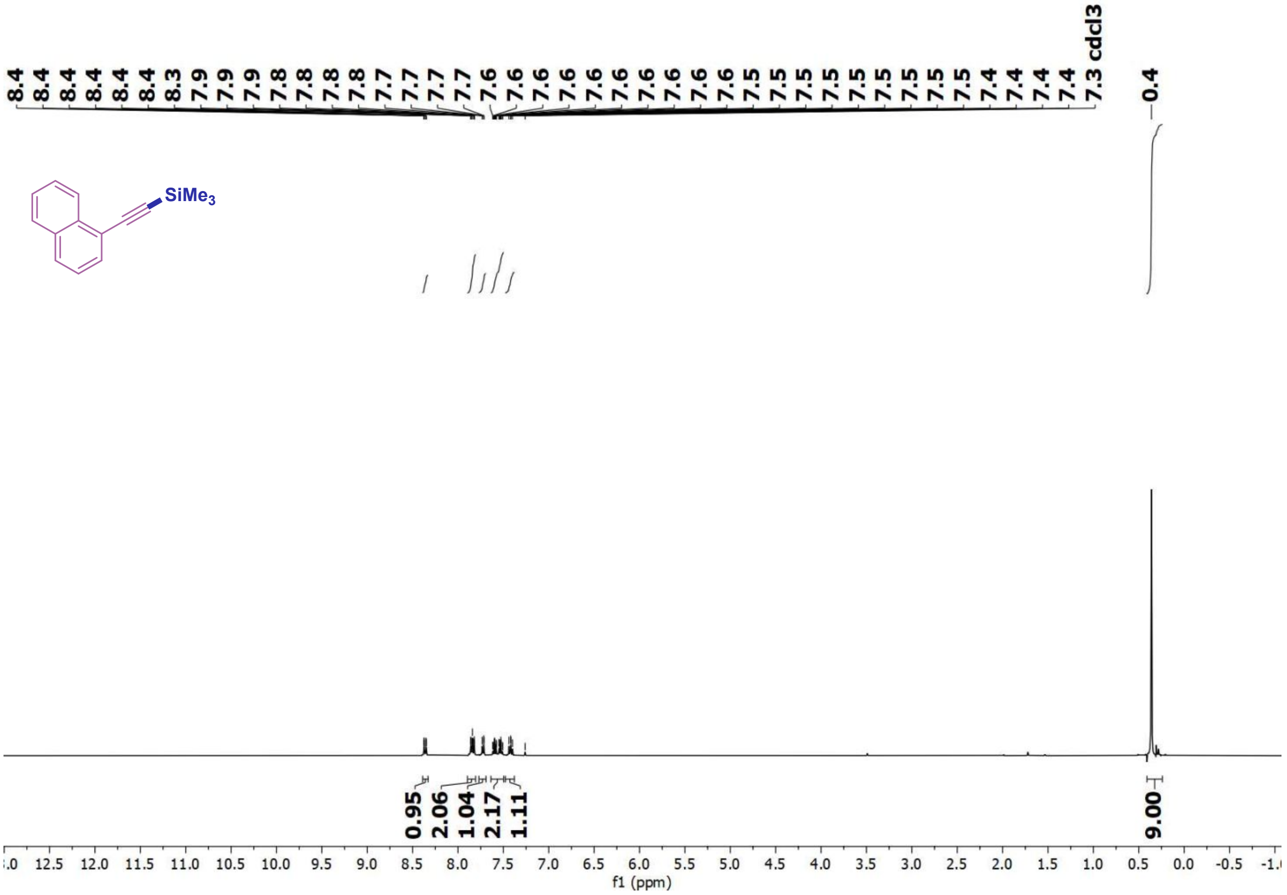


Figure S22. $^{1}H$ NMR (400 MHz, Chloroform-d, 25°C) of trimethyl(naphthalen-1-ylethynyl)silane (**3h**).

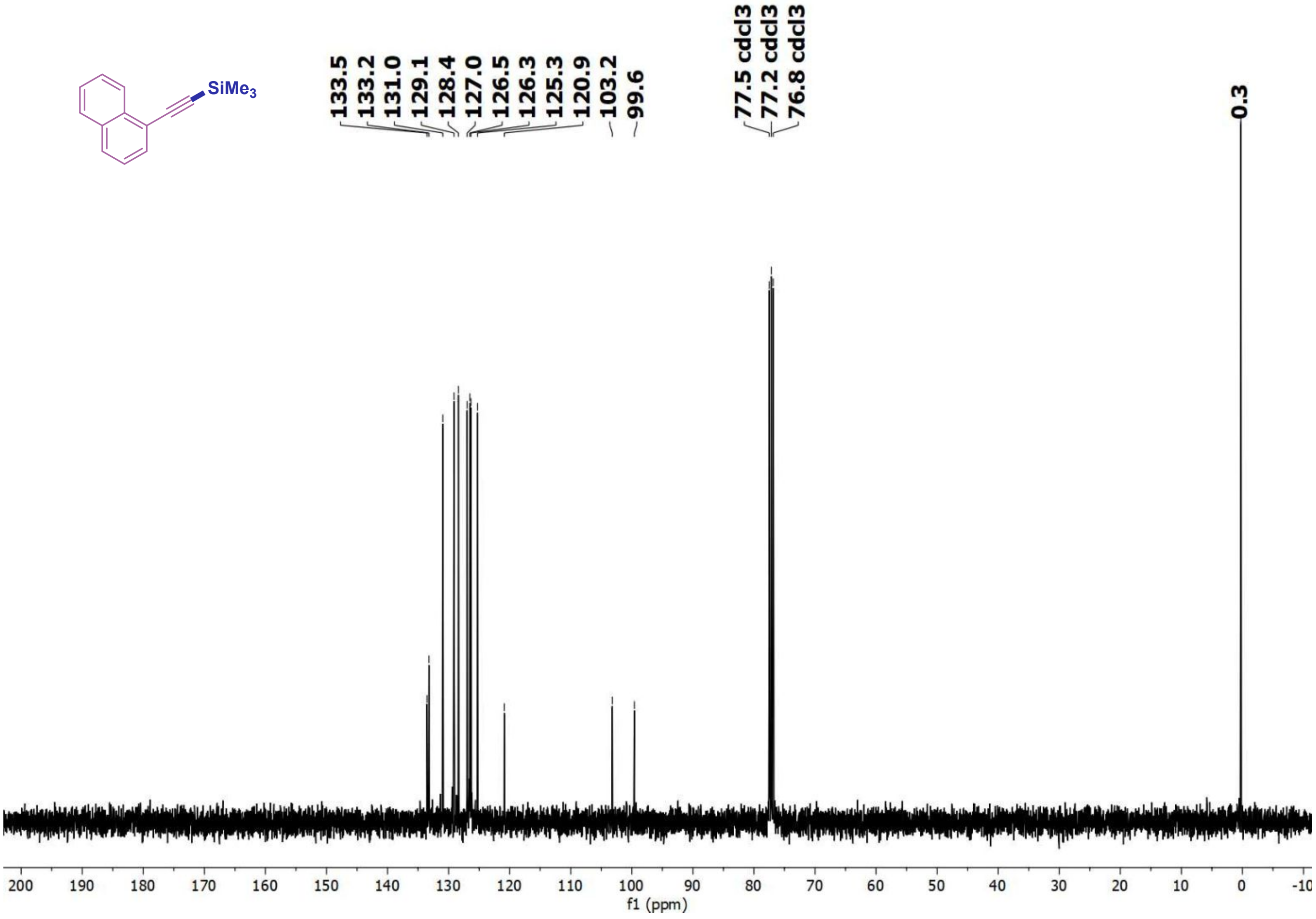


Figure S23. $^{13}C\{^{1}H\}$ NMR (101 MHz, Chloroform-d, 25°C) of trimethyl(naphthalen-1-ylethynyl)silane (**3h**).

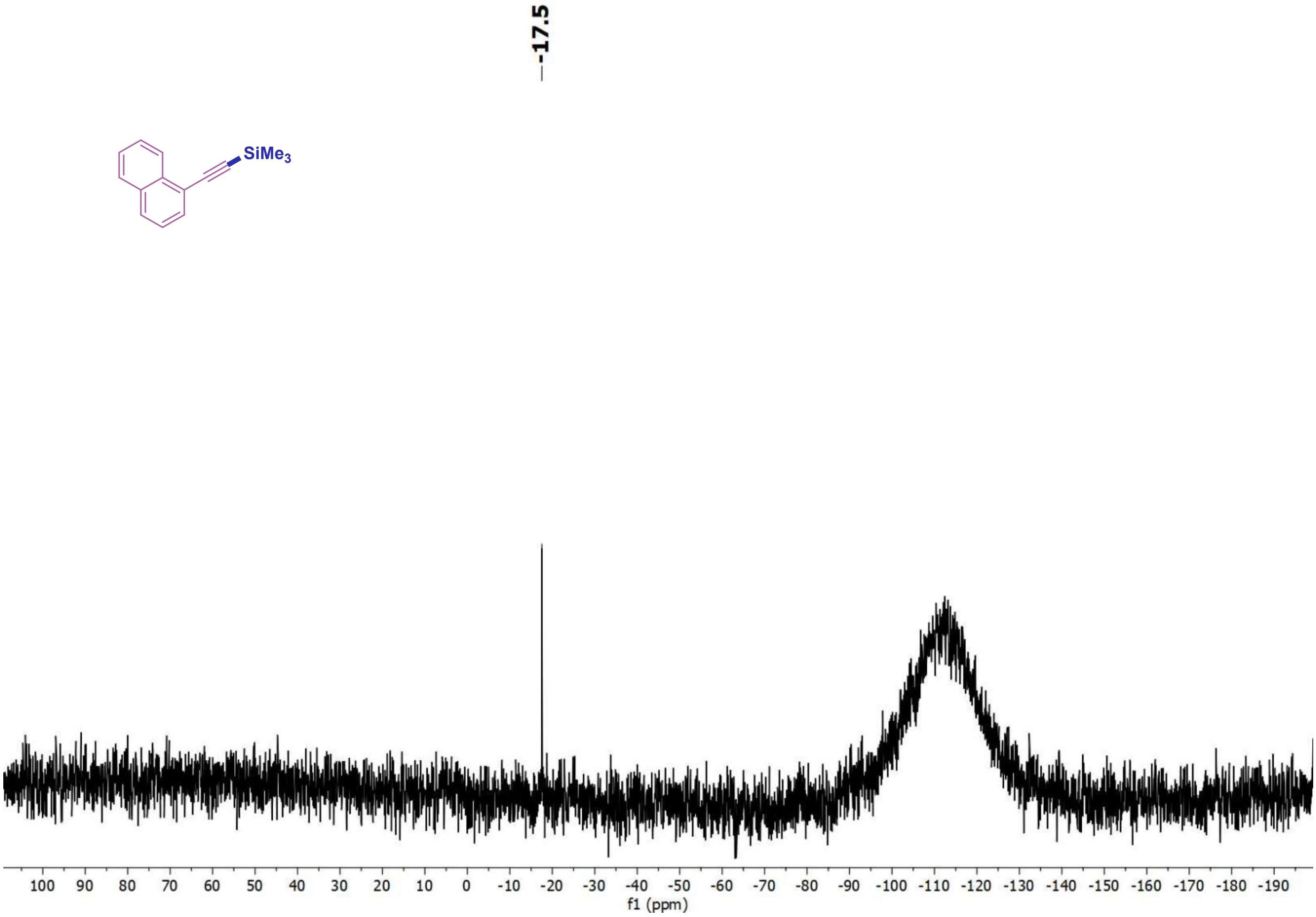


Figure S24. $^{29}$Si NMR (79 MHz, Chloroform-d, 25°C) of trimethyl(naphthalen-1-ylethynyl)silane (**3h**).

# ((2-Fluorophenyl)ethynyl)trimethylsilane (**3i**)

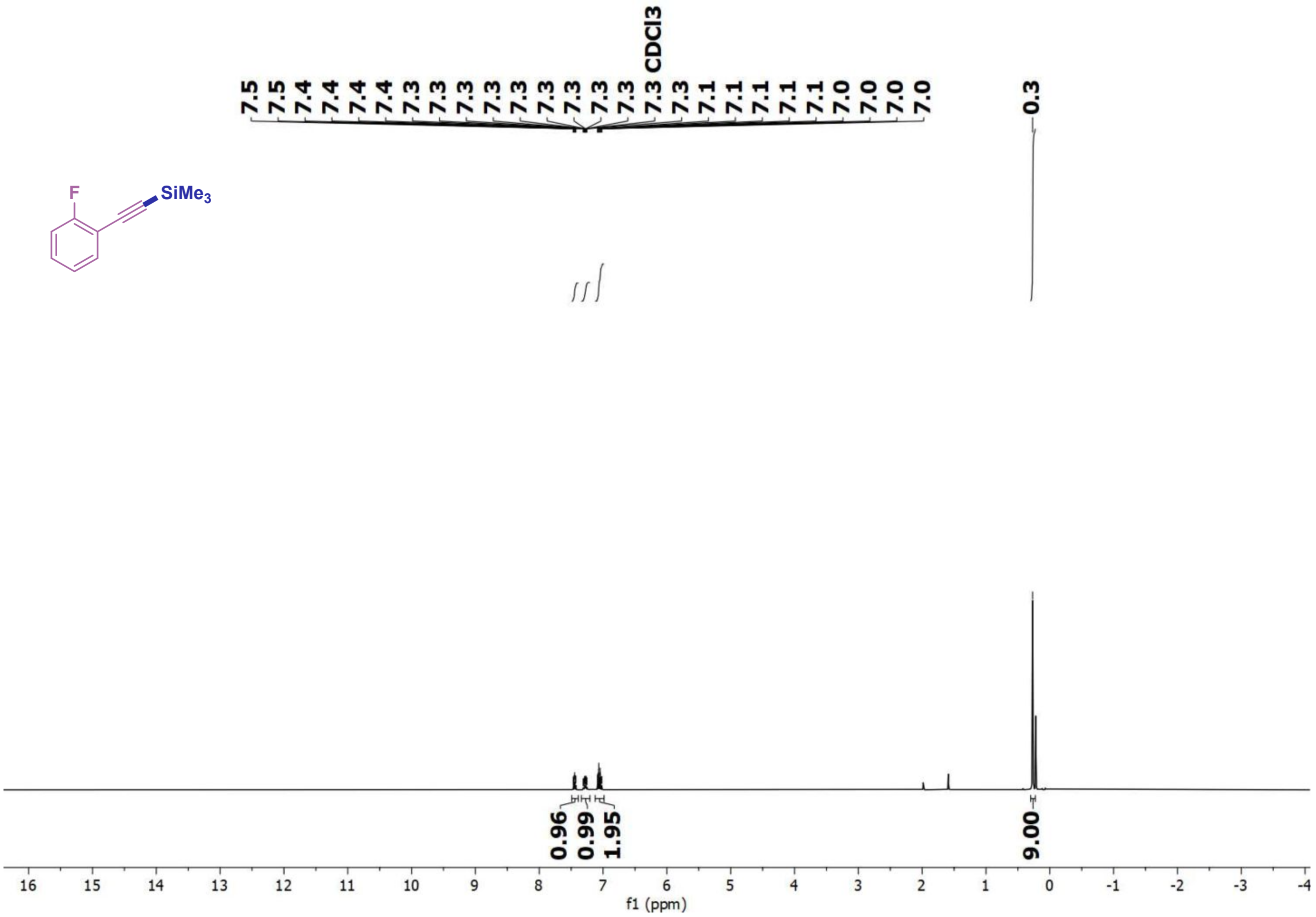


Figure S24. $^{1}H$ NMR (400 MHz, Chloroform-d, 25°C) of ((2-fluorophenyl)ethynyl)trimethylsilane (**3i**).

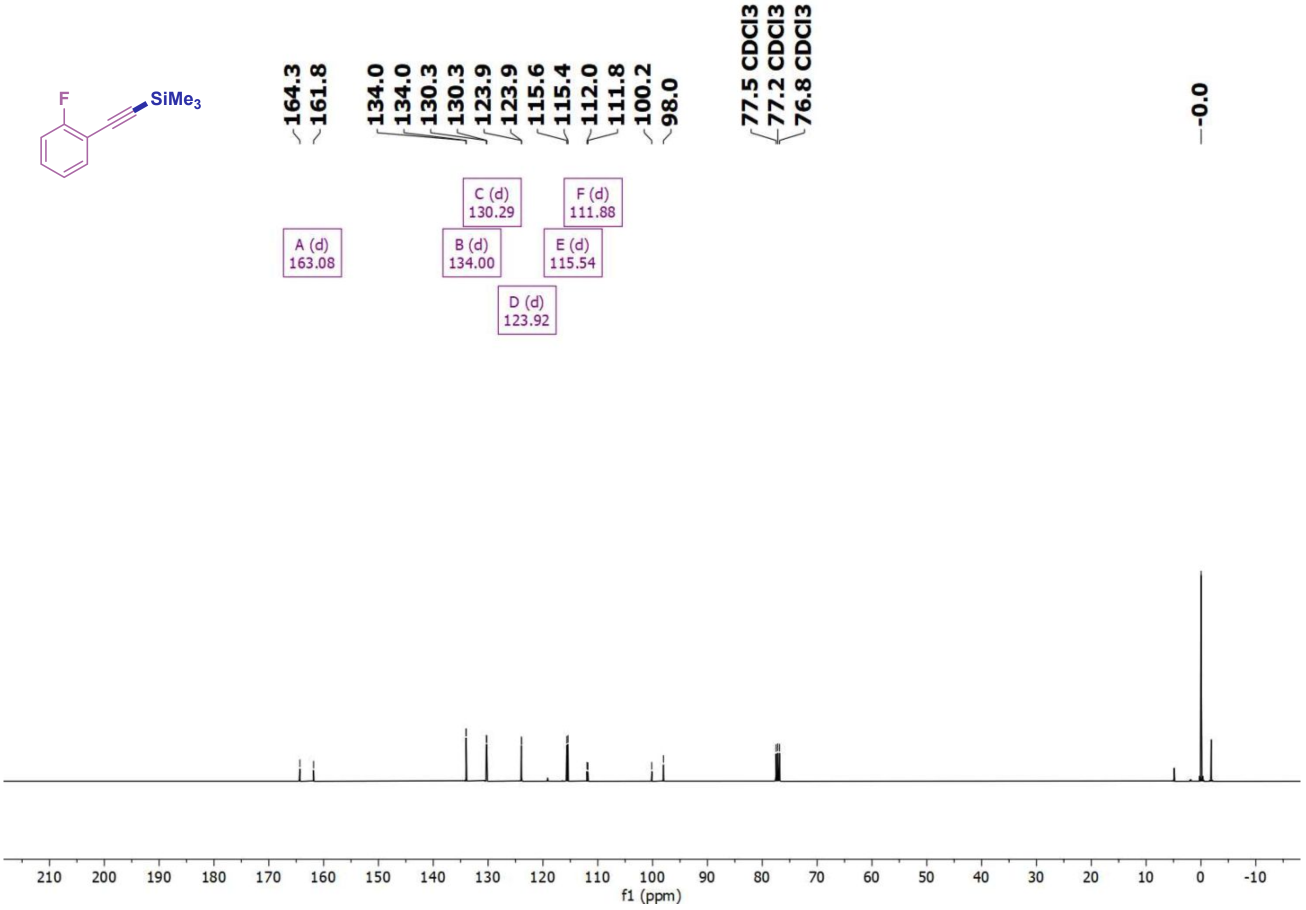


Figure S25. $^{13}C\{^{1}H\}$ NMR (101 MHz, Chloroform-d, 25°C) of ((2-fluorophenyl)ethynyl)trimethylsilane (**3i**).

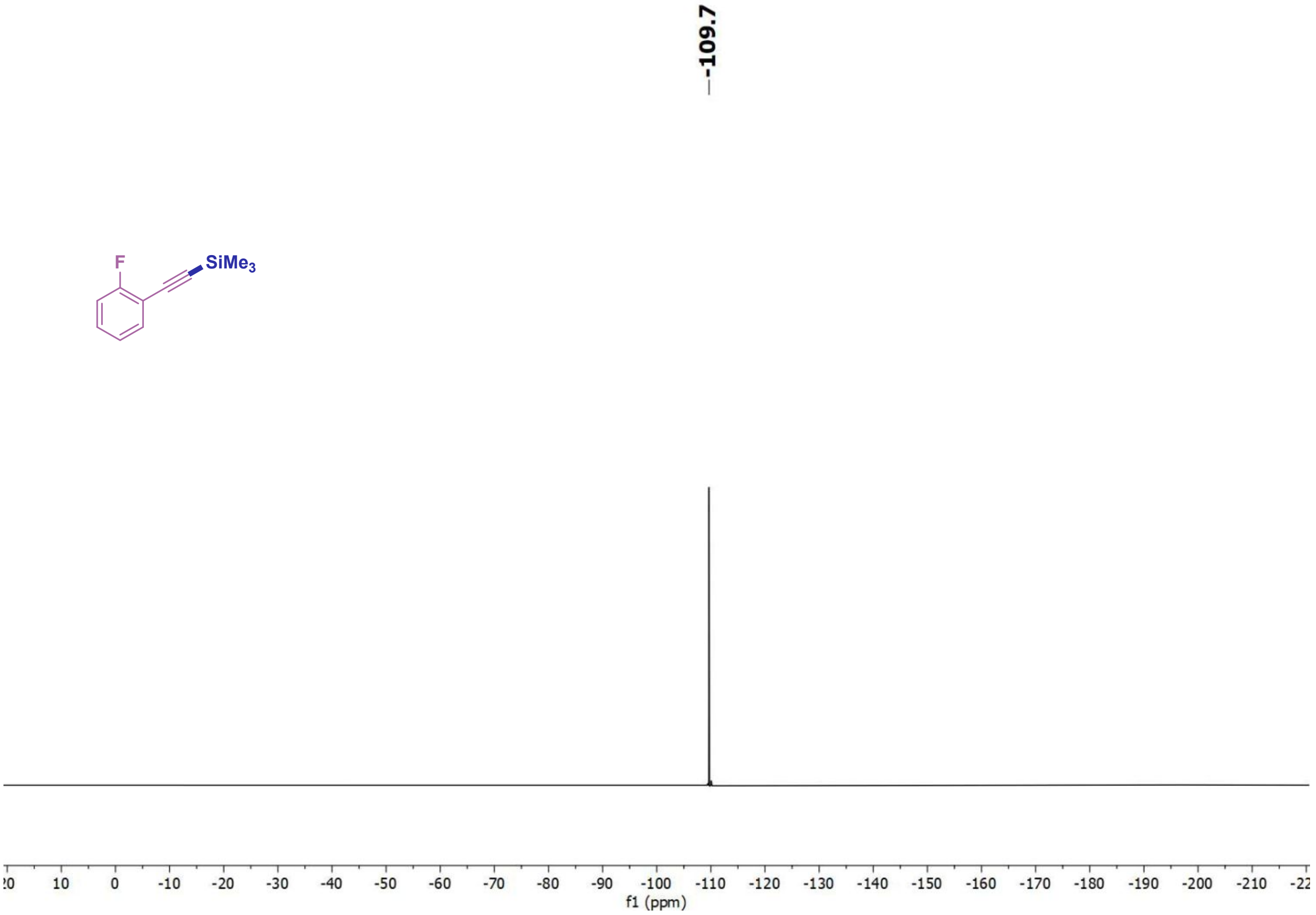


Figure S26. $^{19}$F NMR (377 MHz, Chloroform-d, 25°C) of ((2-fluorophenyl)ethynyl)trimethylsilane (**3i**).

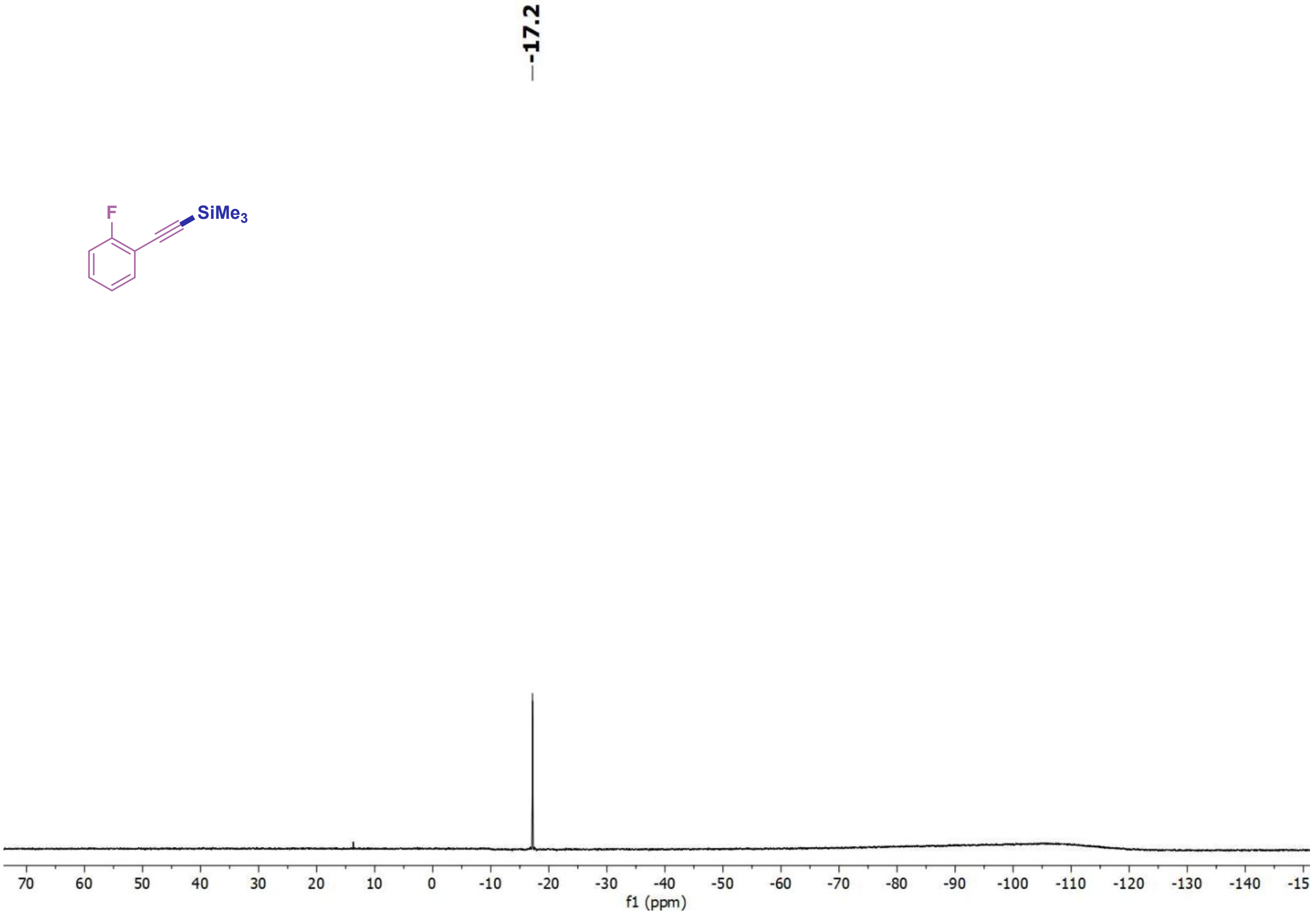


Figure S27. $^{29}$Si NMR (79 MHz, Chloroform-d, 25°C) of ((2-fluorophenyl)ethynyl)trimethylsilane (**3i**).

## ((3-Fluorophenyl)ethynyl)trimethylsilane (**3j**)

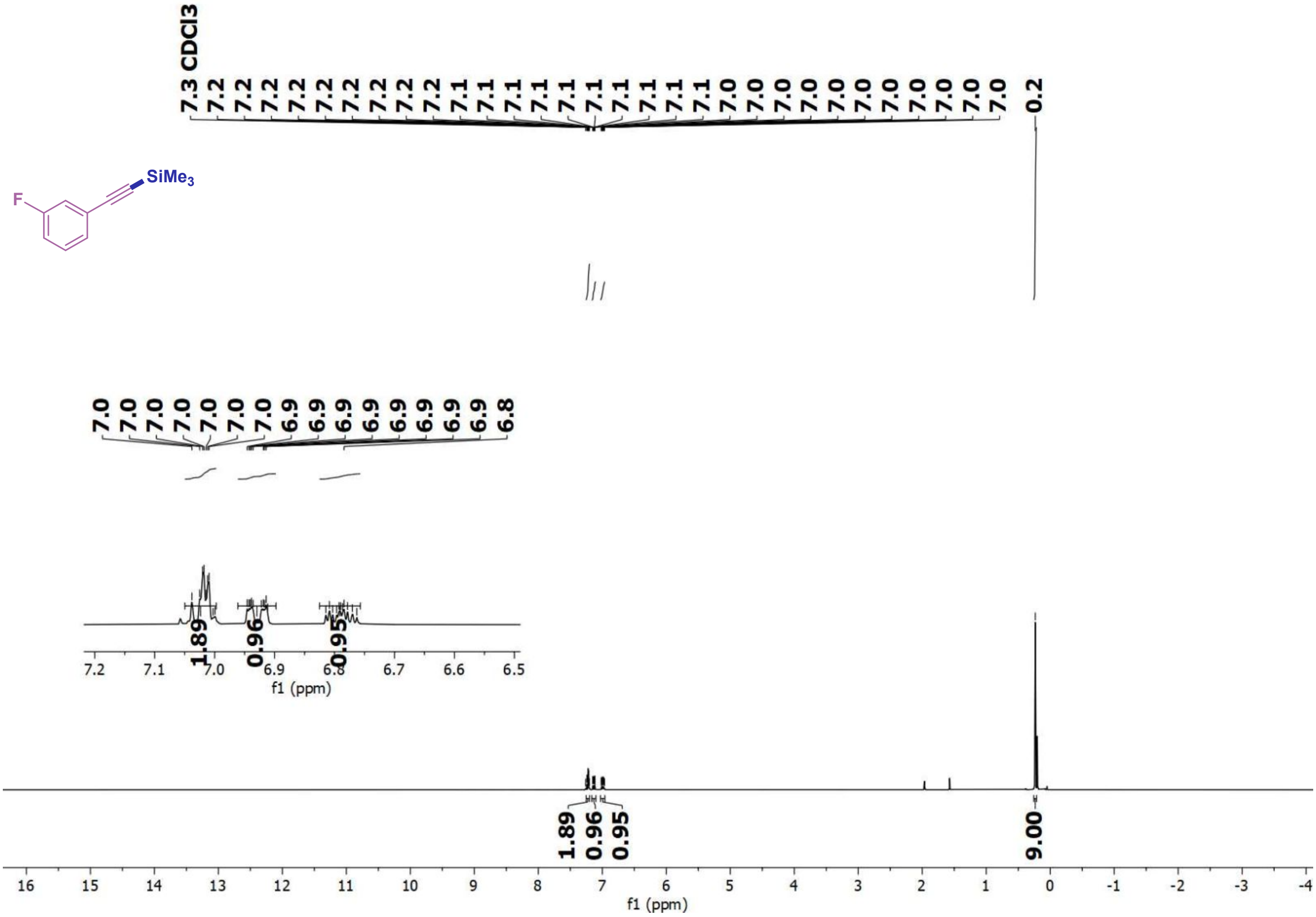


Figure S28. $^{1}$H NMR (400 MHz, Chloroform-d, 25°C) of ((3-fluorophenyl)ethynyl)trimethylsilane (**3j**).

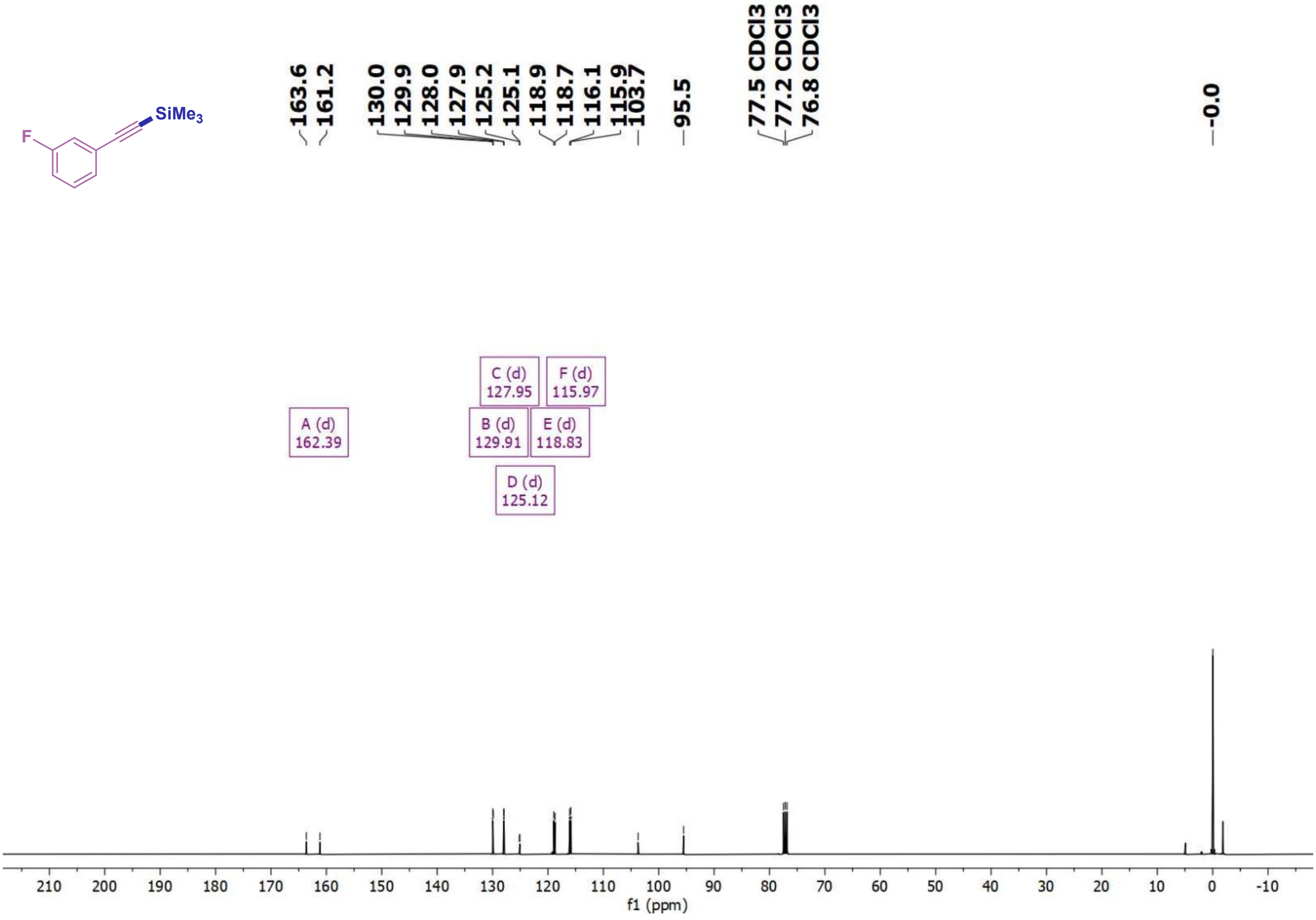


Figure S29. $^{13}C\{^{1}H\}$ NMR (101 MHz, Chloroform-d, 25°C) of ((3-fluorophenyl)ethynyl)trimethylsilane (**3j**).

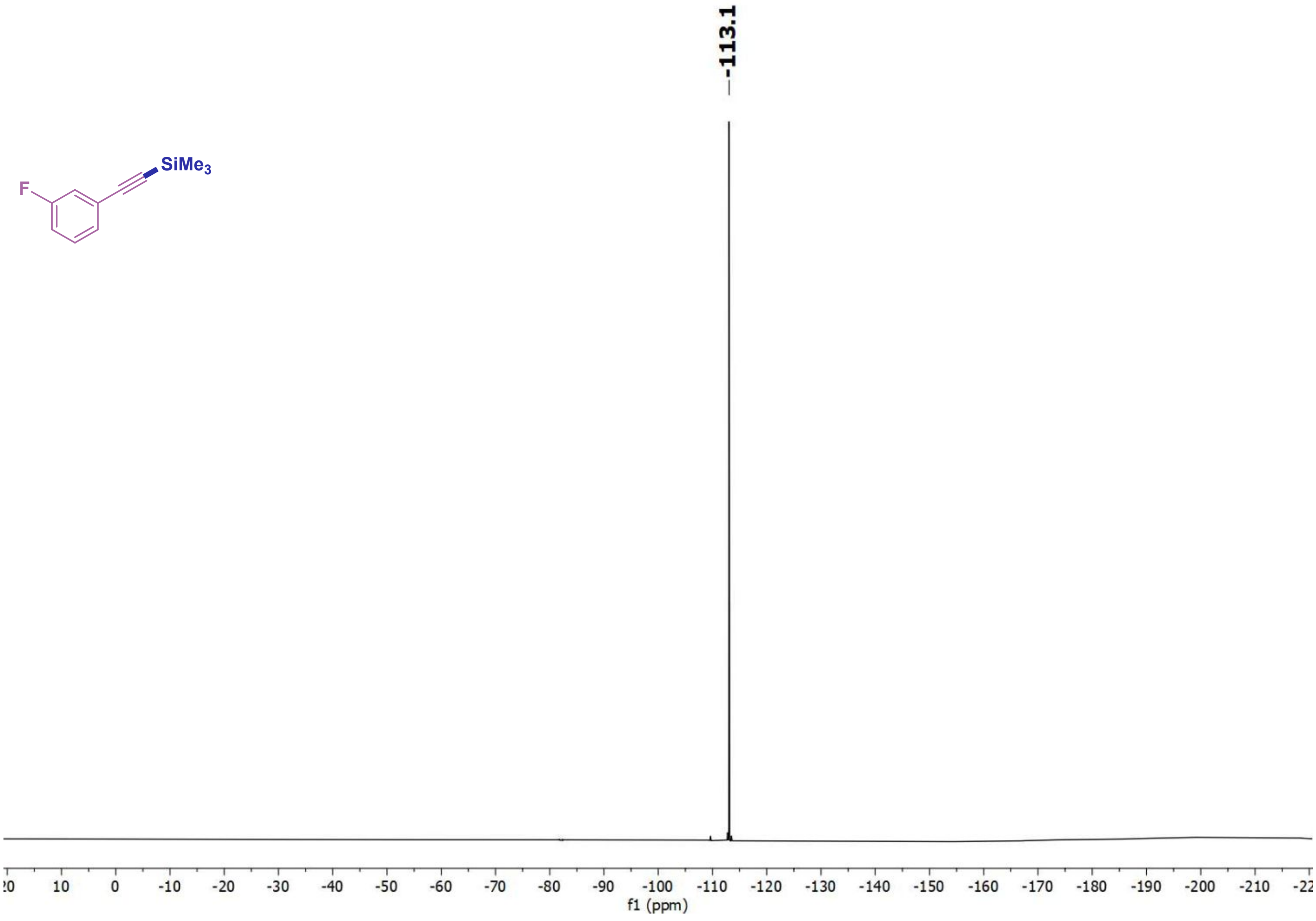


Figure S30. $^{19}F$ NMR (377 MHz, Chloroform-d, 25°C) of ((3-fluorophenyl)ethynyl)trimethylsilane (**3j**).

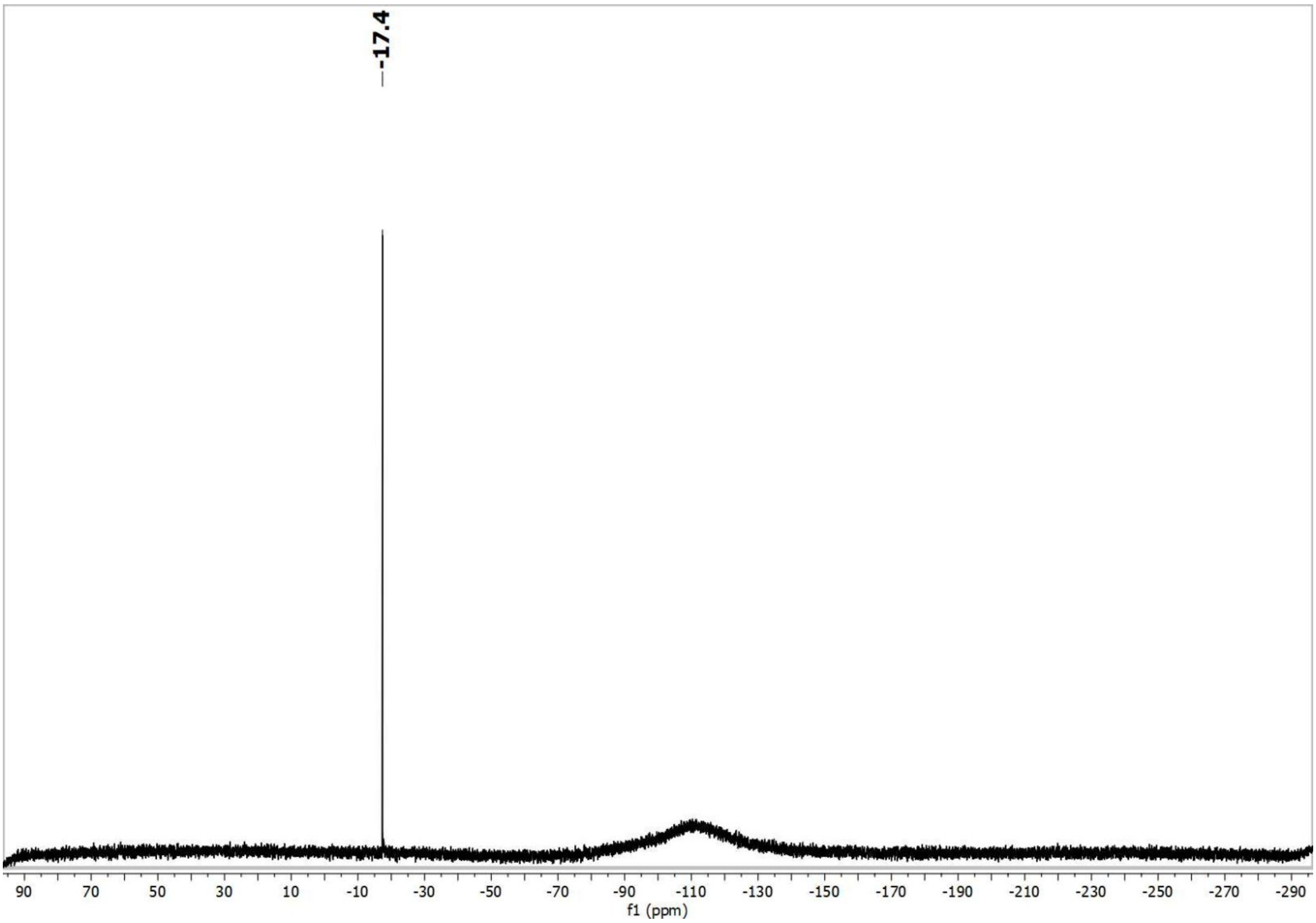


Figure S31. $^{29}Si$ NMR (79 MHz, Chloroform-d, 25°C) of ((3-fluorophenyl)ethynyl)trimethylsilane (**3j**).

((4-Fluorophenyl)ethynyl)trimethylsilane (**3k**)

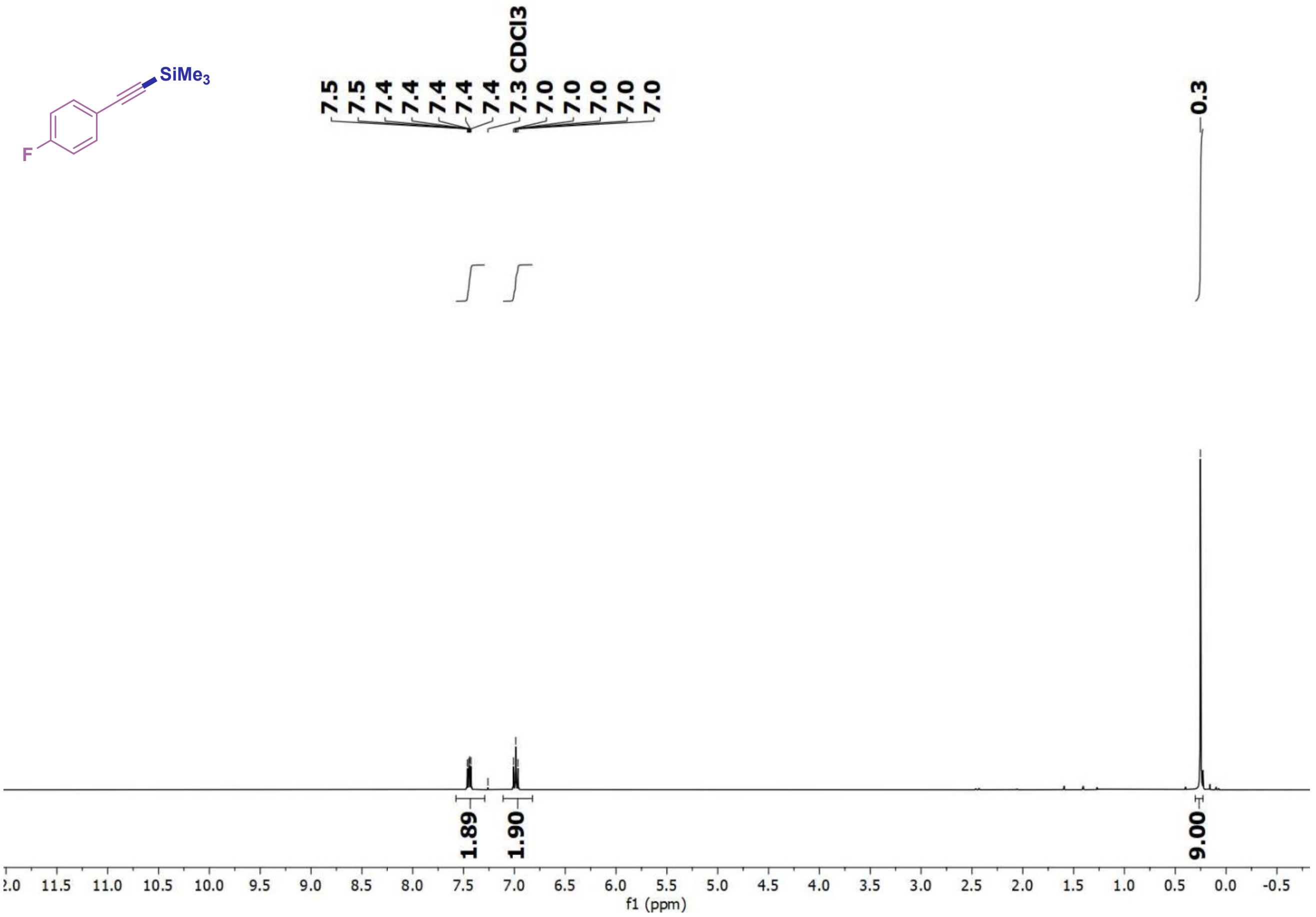

Figure S32. $^{1}H$ NMR (400 MHz, Chloroform-d, 25°C) of ((4-fluorophenyl)ethynyl)trimethylsilane (**3k**).

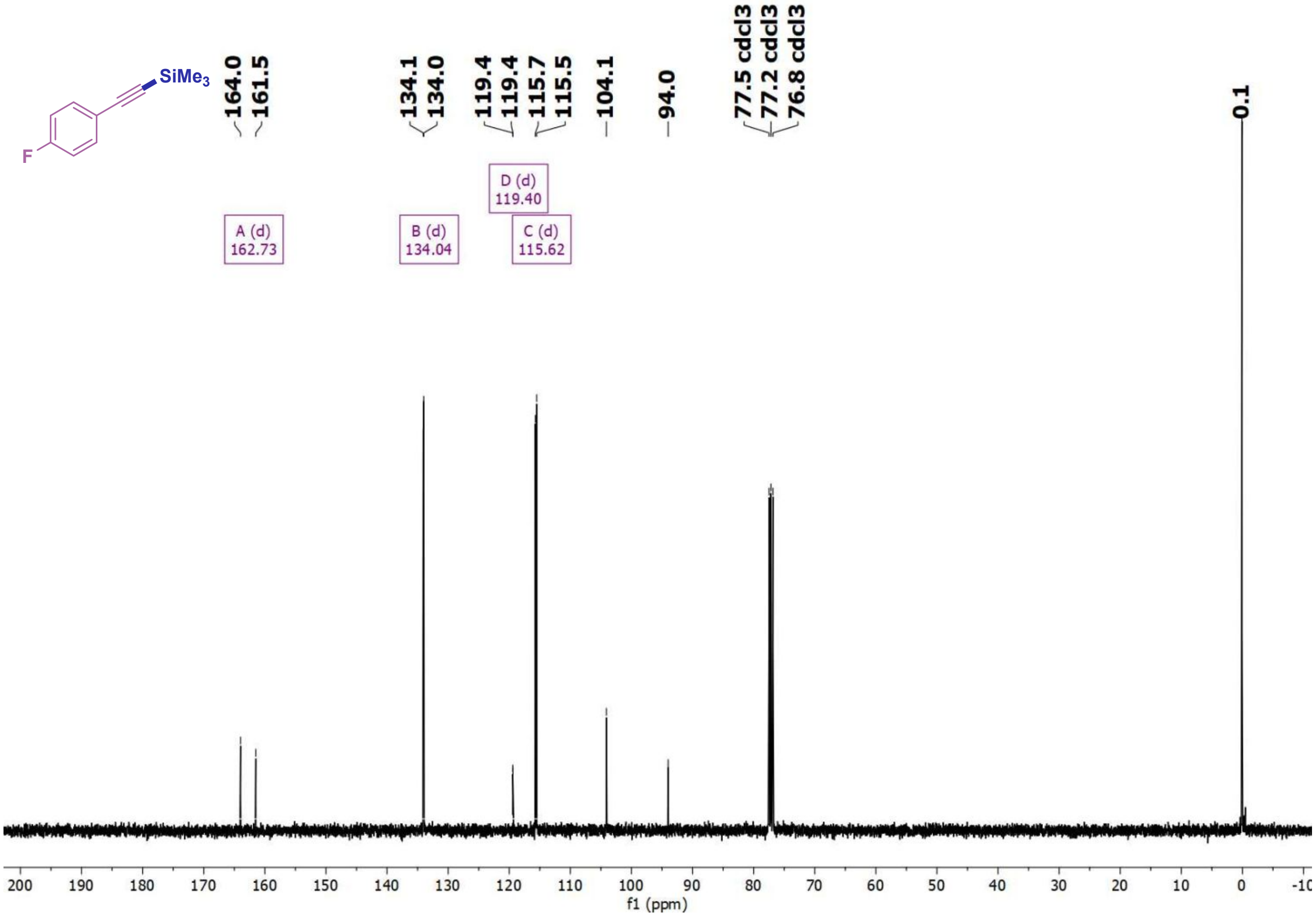

Figure S33. $^{13}C\{^{1}H\}$ NMR (101 MHz, Chloroform-d, 25°C) of ((4-fluorophenyl)ethynyl)trimethylsilane (**3k**).

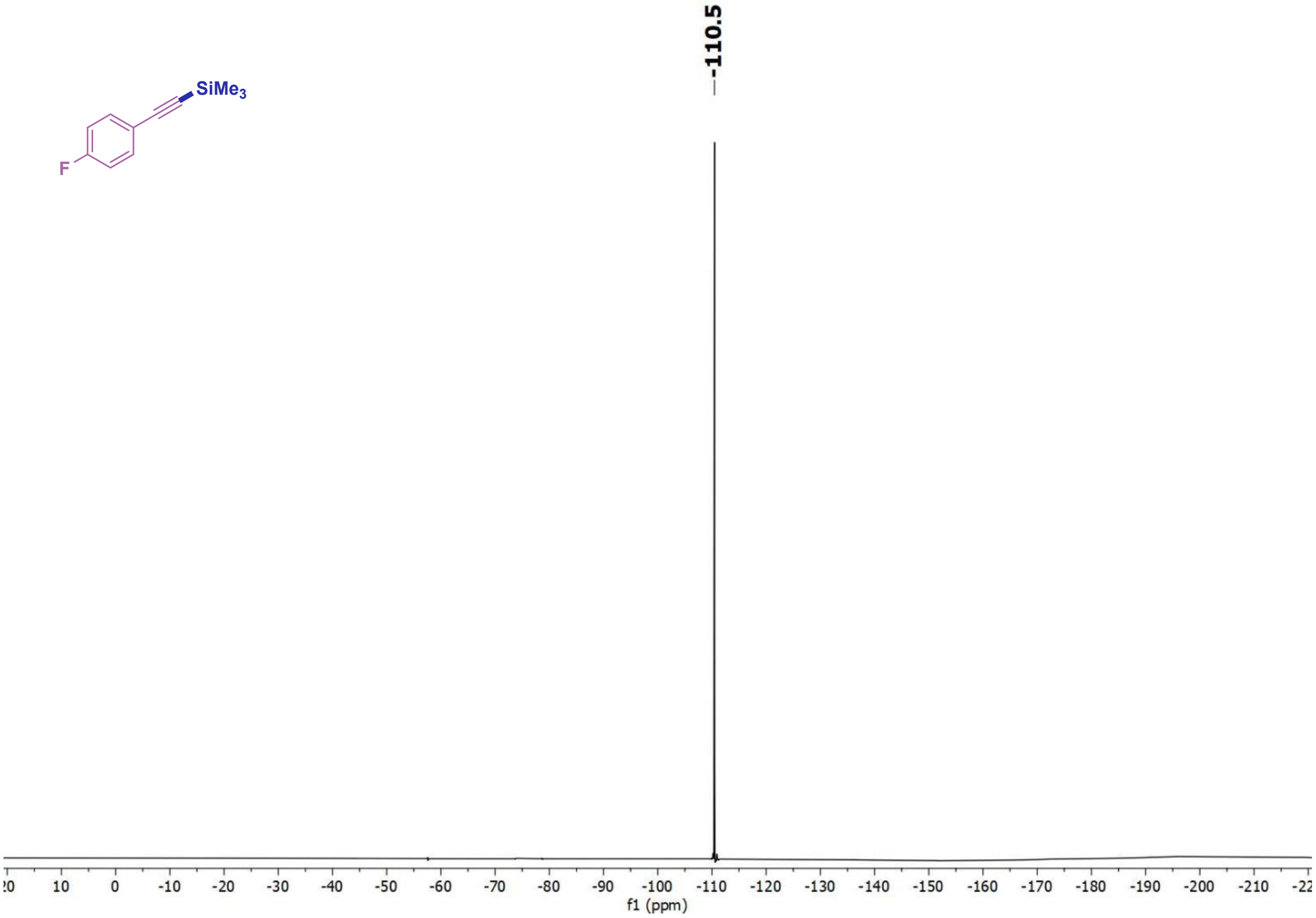


Figure S34. $^{19}F$ NMR (377 MHz, Chloroform-d, 25°C) of ((4-fluorophenyl)ethynyl)trimethylsilane (**3k**).

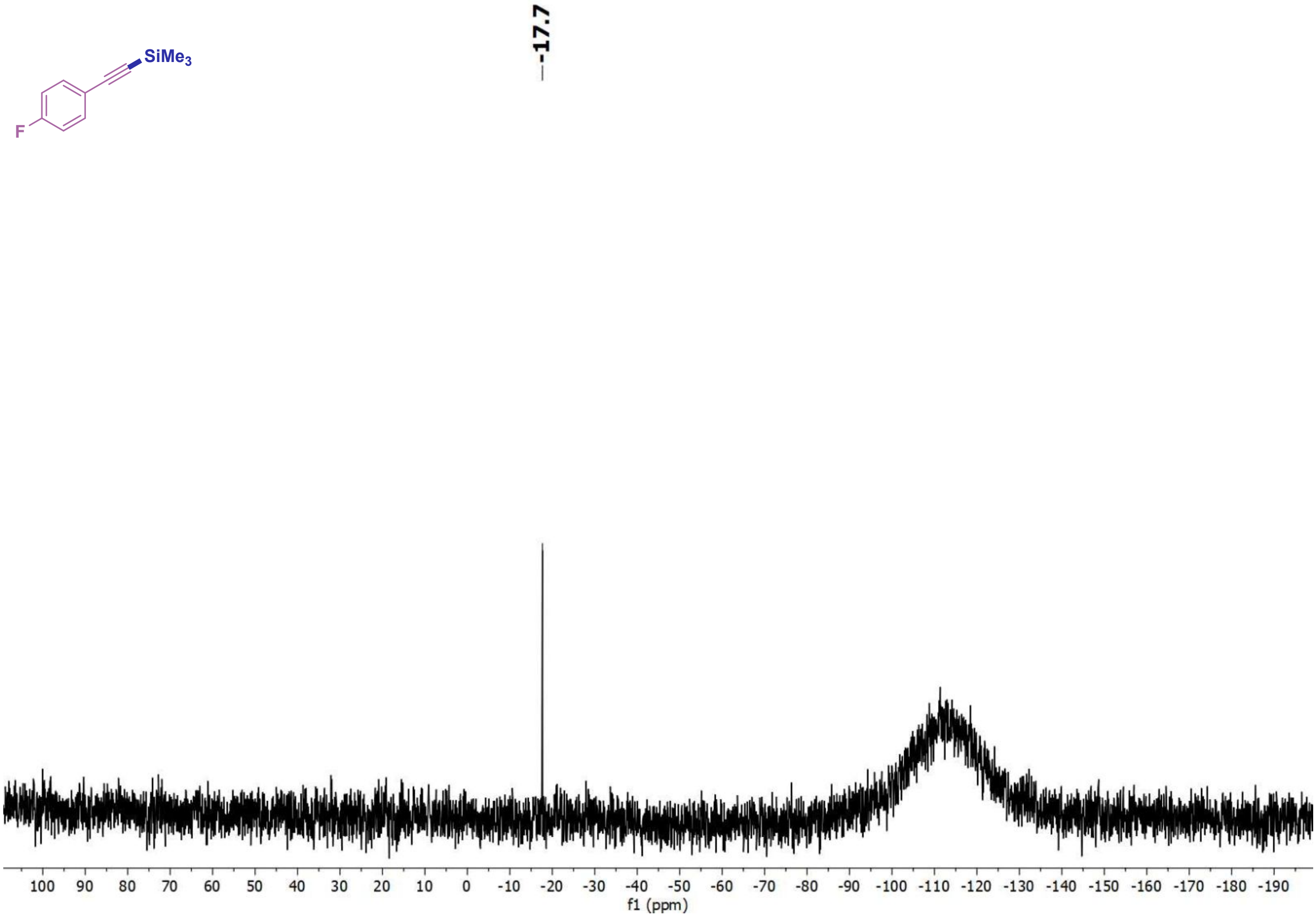


Figure S35. $^{29}Si$ NMR (79 MHz, Chloroform-d, 25°C) of ((4-fluorophenyl)ethynyl)trimethylsilane (**3k**).

((4-Chlorophenyl)ethynyl)trimethylsilane (**3l**)

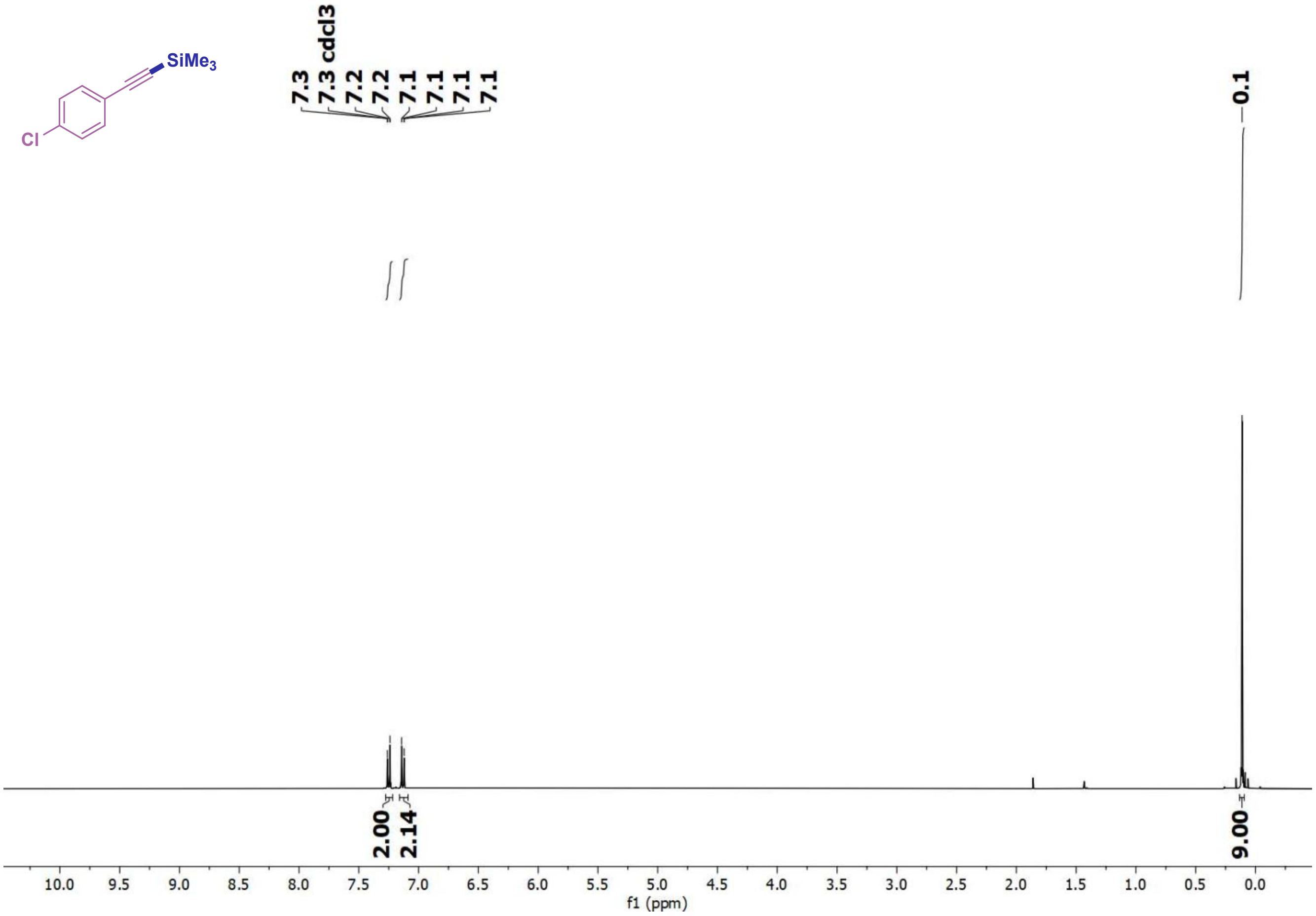


Figure S36. $^{1}$H NMR (400 MHz, Chloroform-d, 25°C) of ((4-chlorophenyl)ethynyl)trimethylsilane (**3l**).

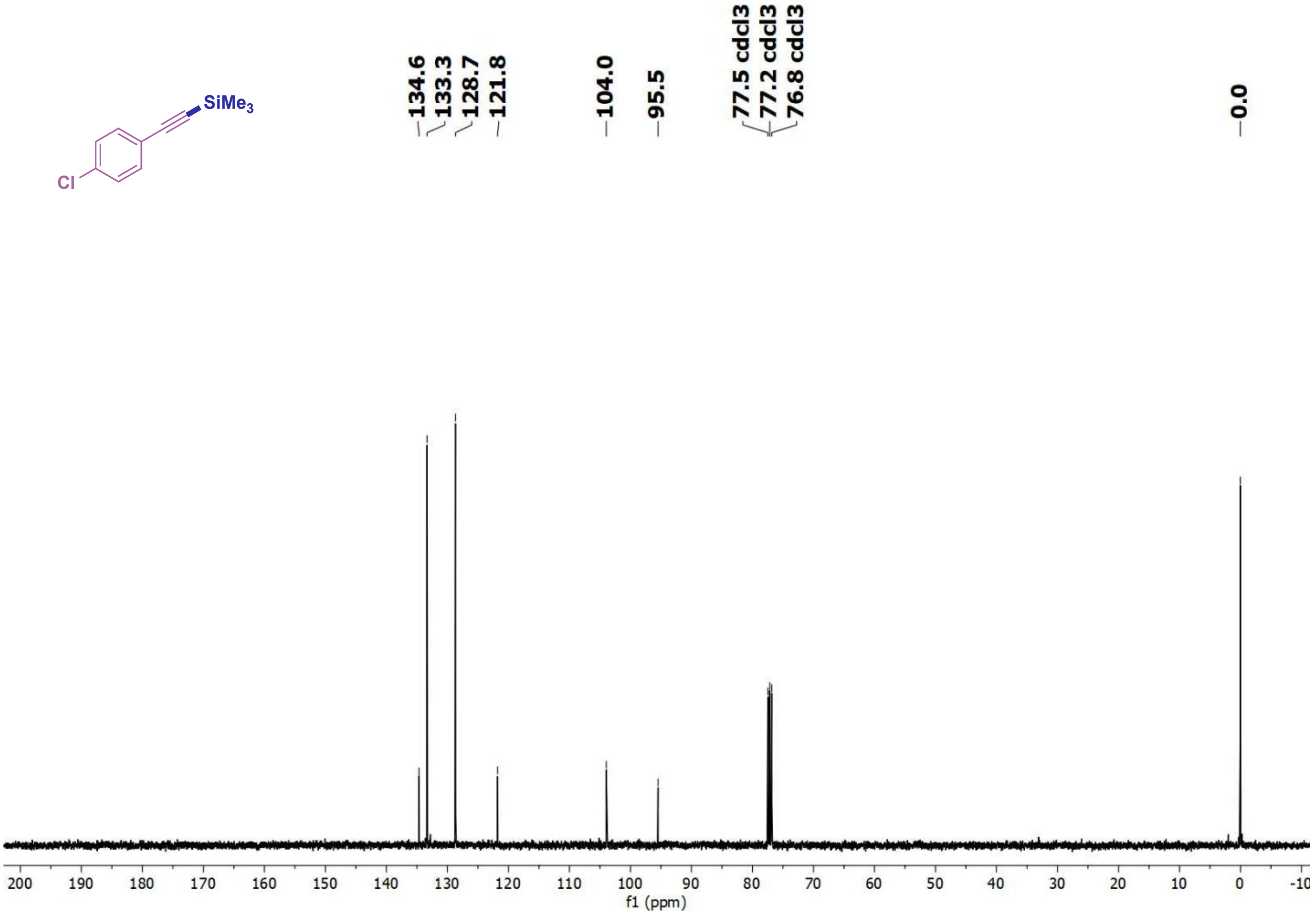


Figure S37. $^{13}$C{$^{1}$H} NMR (101 MHz, Chloroform-d, 25°C) of ((4-chlorophenyl)ethynyl)trimethylsilane (**3l**).

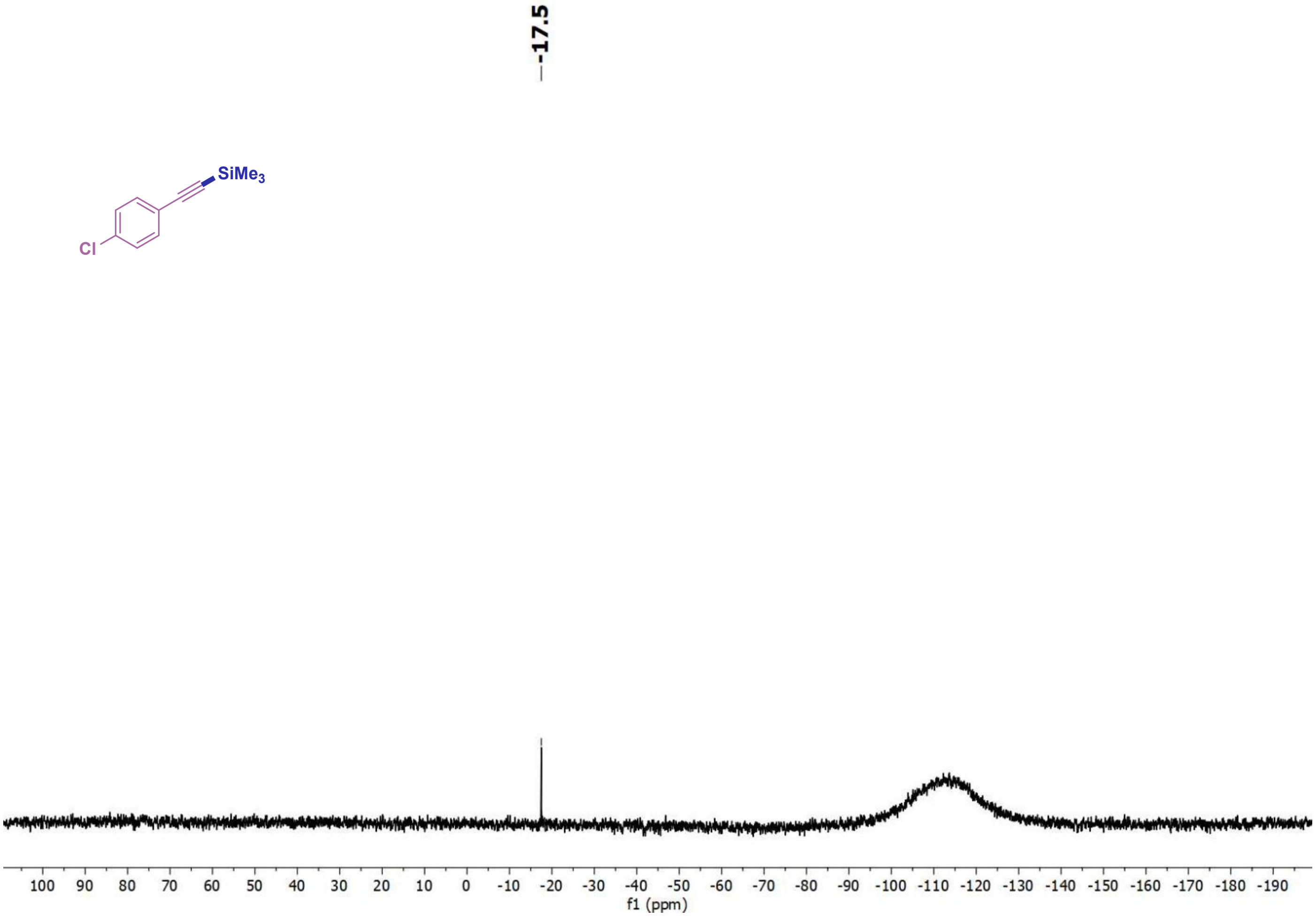


Figure S38. $^{29}$Si NMR (79 MHz, Chloroform-d, 25°C) of ((4-chlorophenyl)ethynyl)trimethylsilane (**3l**).

# Trimethyl((2-(trifluoromethoxy)phenyl)ethynyl)silane (**3m**)

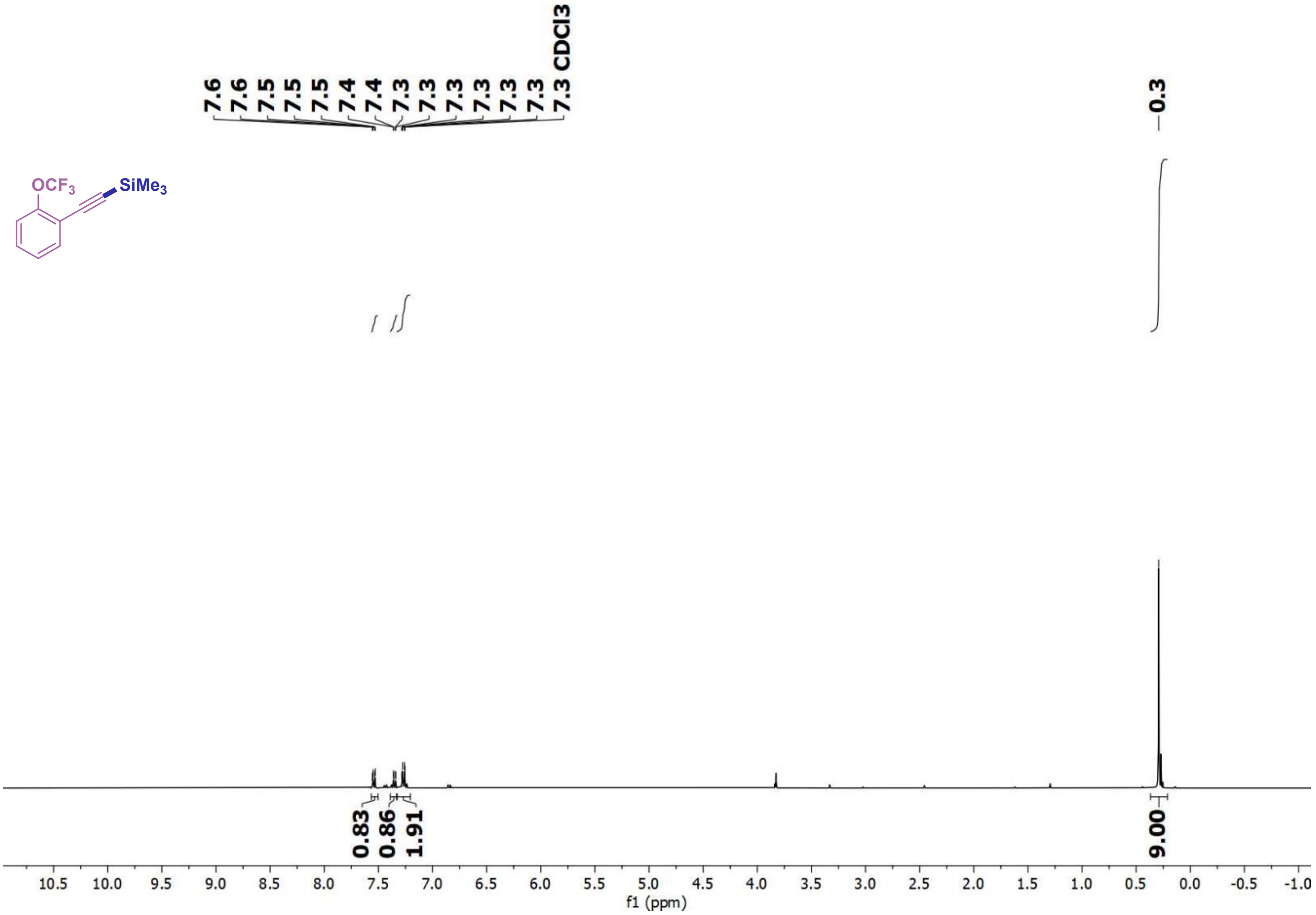


Figure S39. $^{1}H$ NMR (400 MHz, Chloroform-d, 25°C) of trimethyl((2-(trifluoromethoxy)phenyl)ethynyl)silane (**3m**).

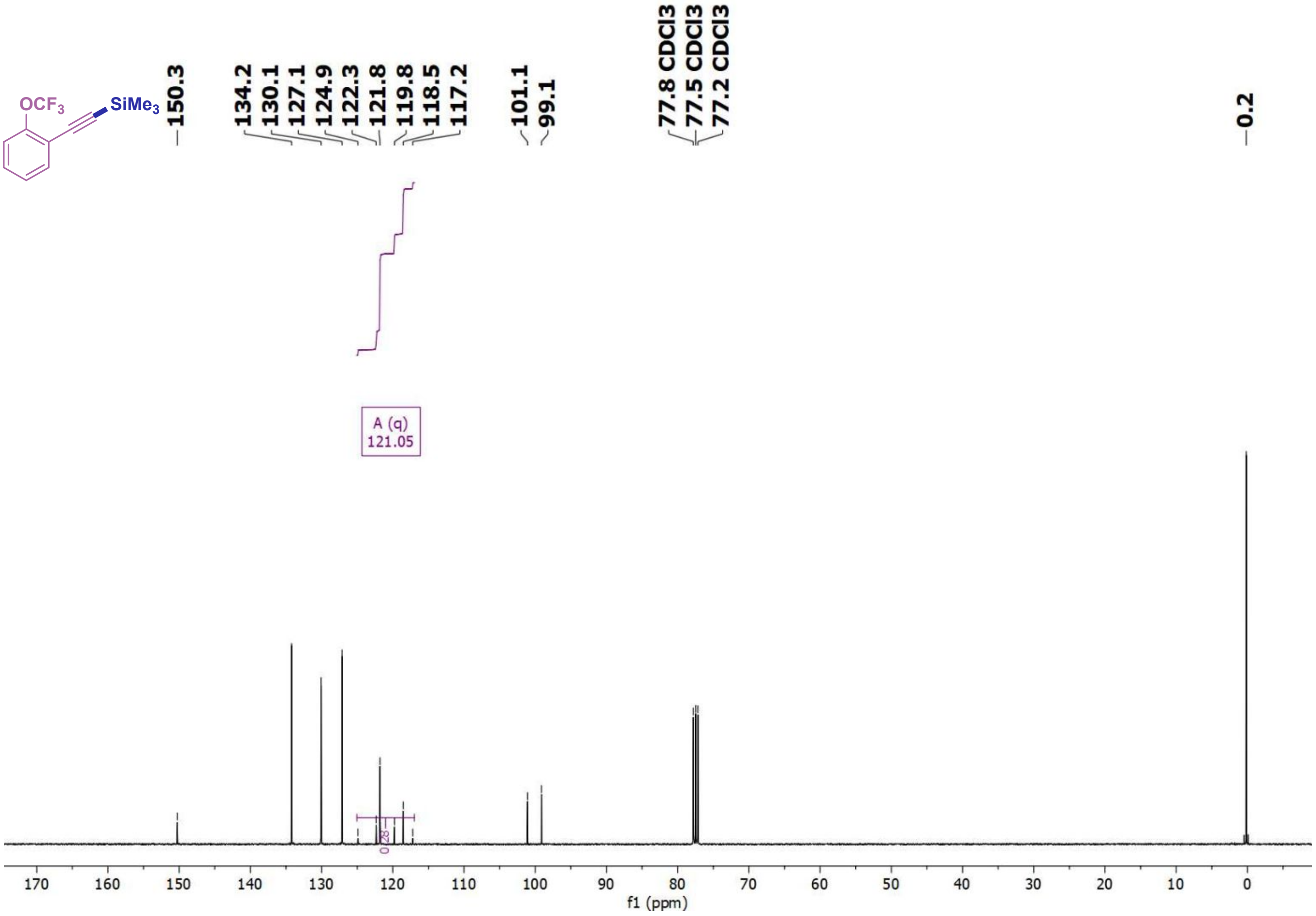


Figure S40. $^{13}C\{^{1}H\}$ NMR (101 MHz, Chloroform-d, 25°C) of trimethyl((2-(trifluoromethoxy)phenyl)ethynyl)silane (**3m**).

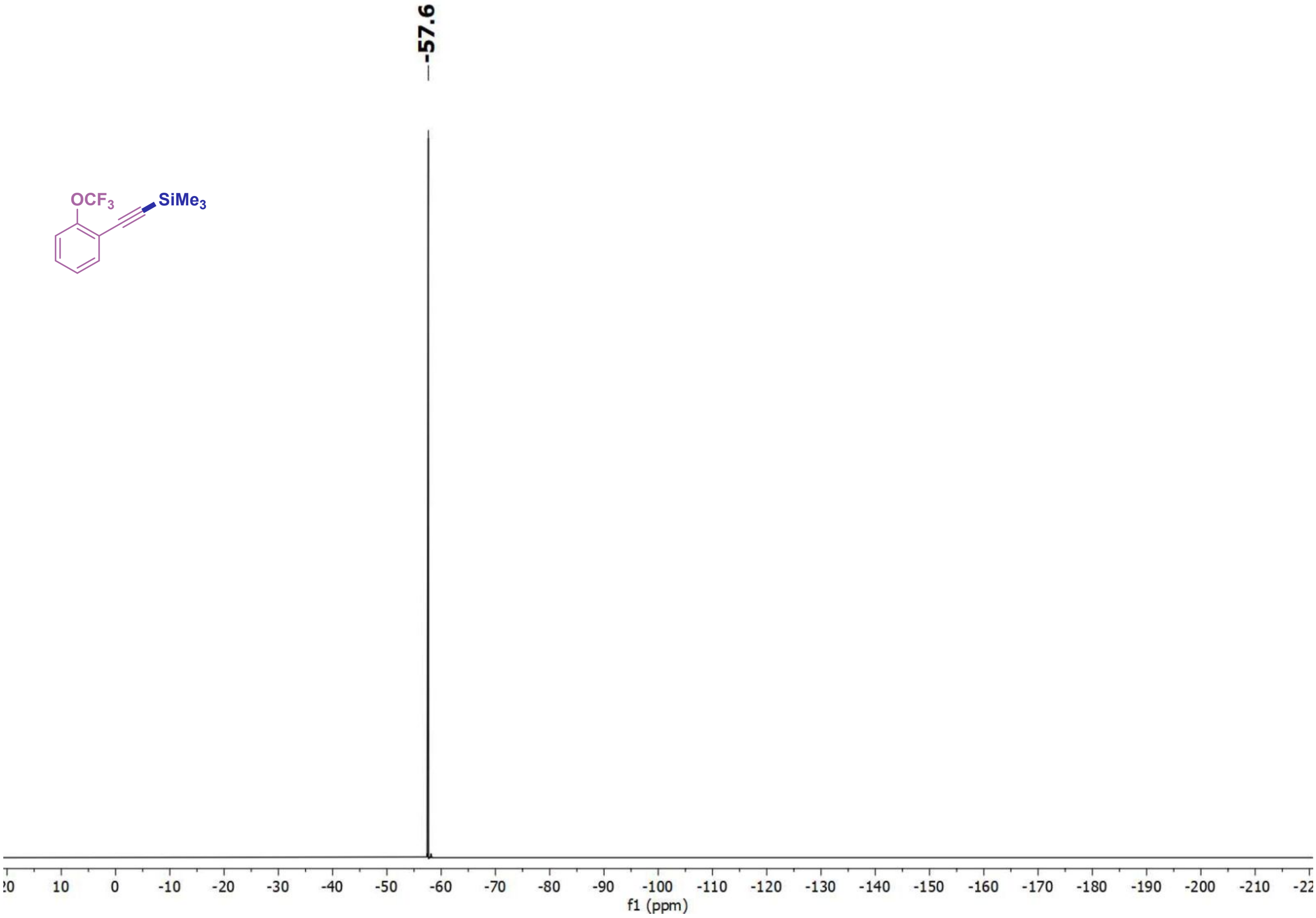


Figure S41. $^{19}$F NMR (377 MHz, Chloroform-d, 25°C) of trimethyl((2-(trifluoromethoxy)phenyl)ethynyl)silane (**3m**).

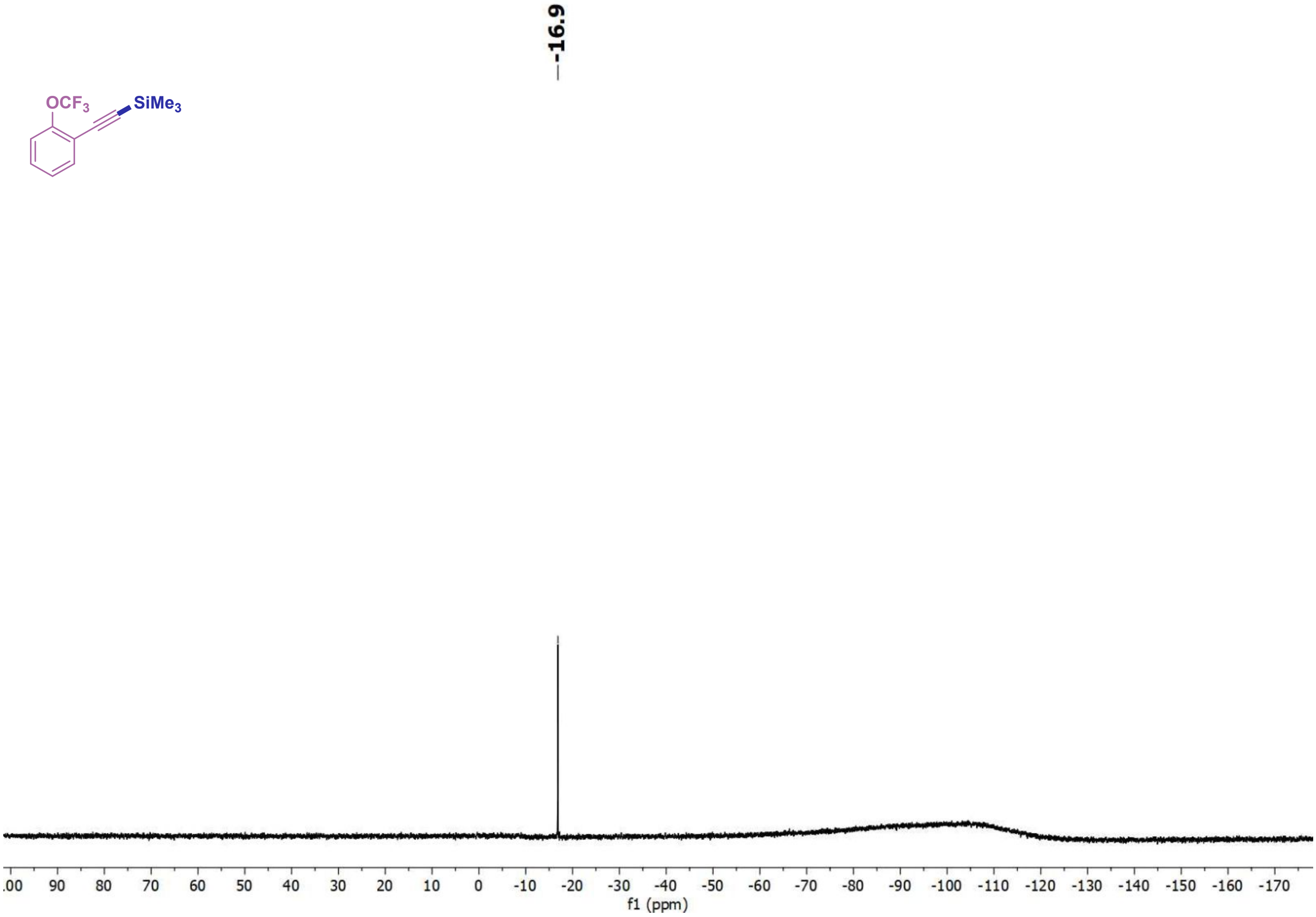


Figure S42. $^{29}$Si NMR (79 MHz, Chloroform-d, 25°C) of trimethyl((2-(trifluoromethoxy)phenyl)ethynyl)silane (**3m**).

# Trimethyl((4-(trifluoromethyl)phenyl)ethynyl)silane (**3n**)

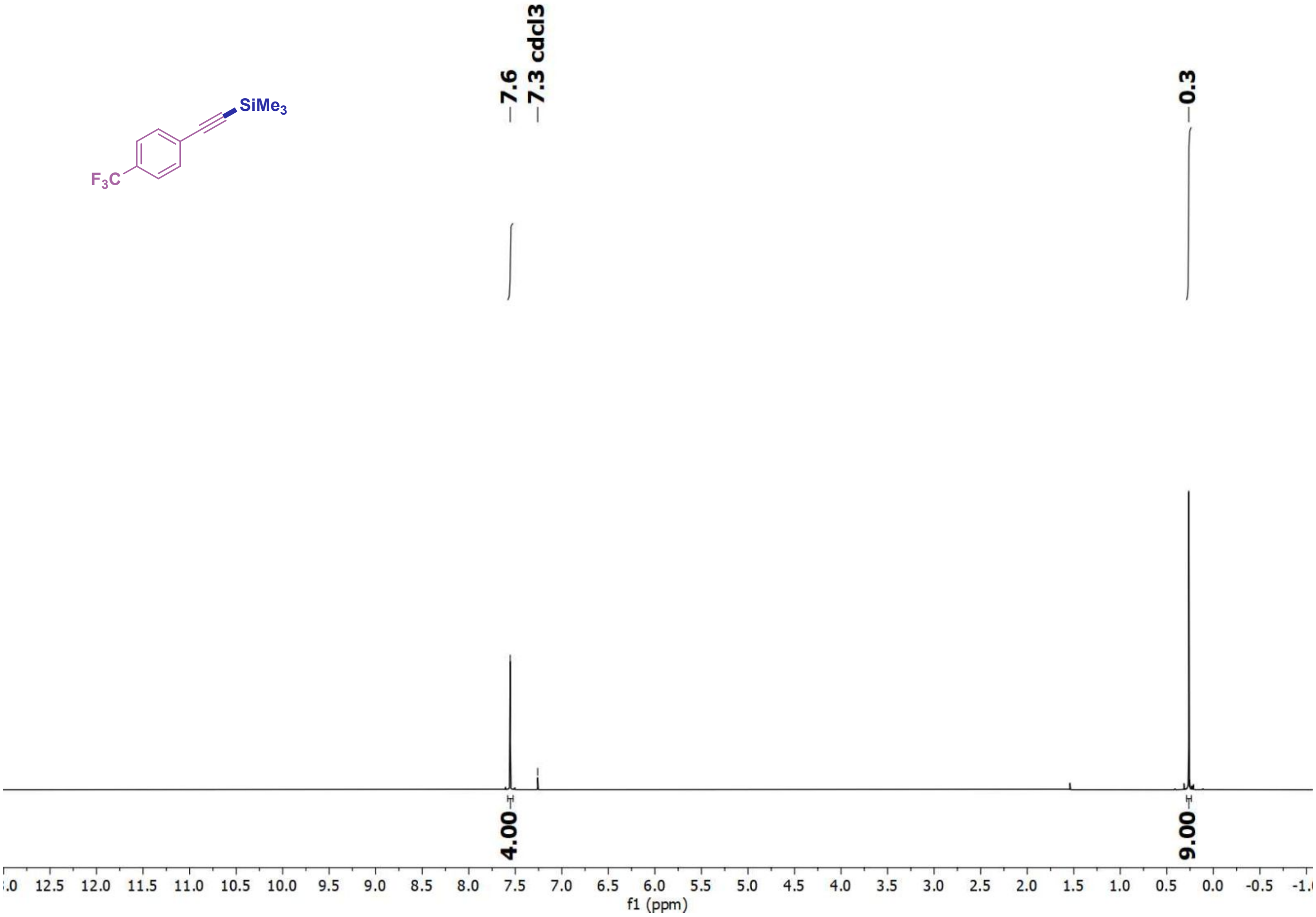


Figure S43. $^{1}H$ NMR (400 MHz, Chloroform-d, 25°C) of trimethyl((4-(trifluoromethyl)phenyl)ethynyl)silane (**3n**).

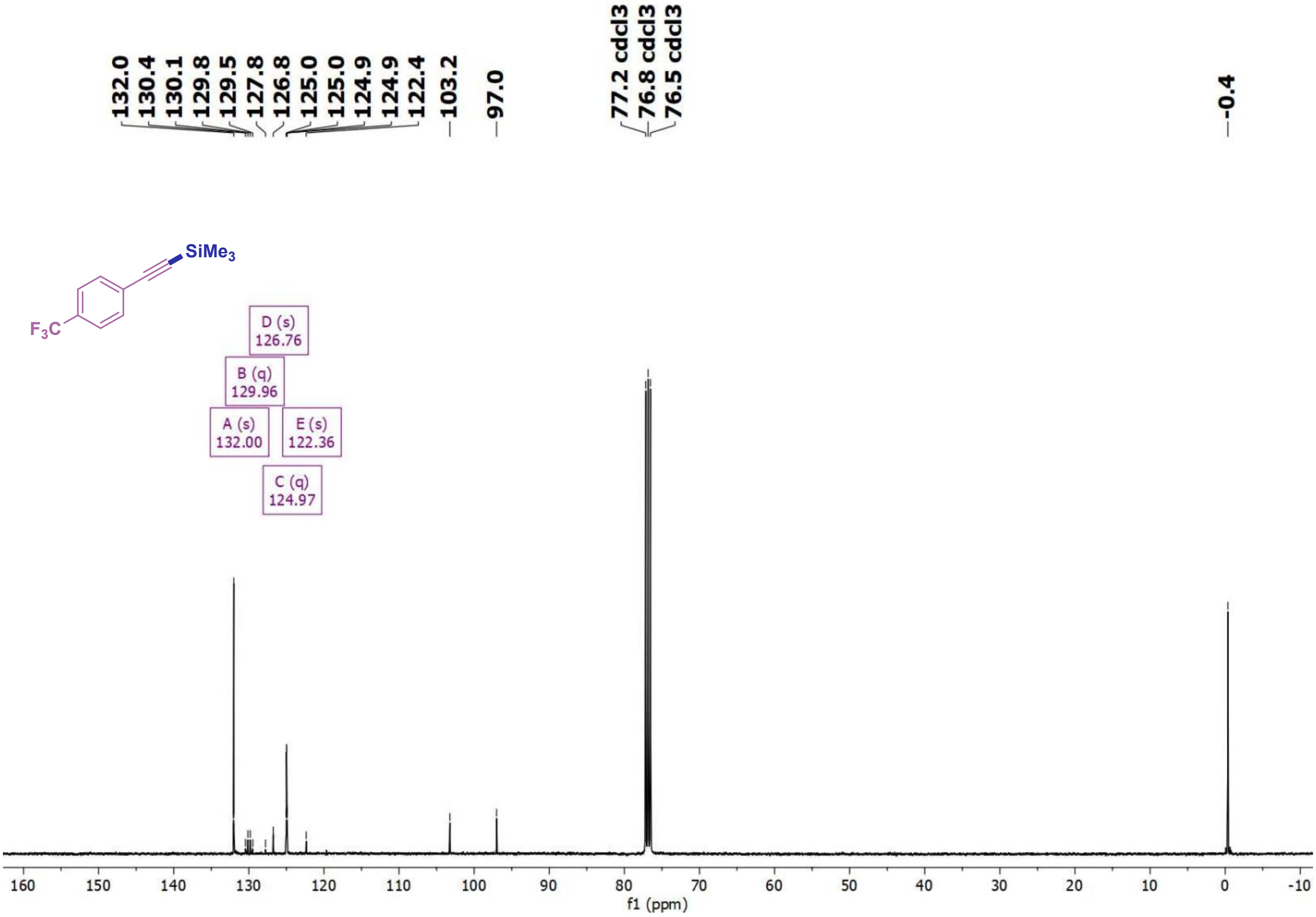


Figure S44. $^{13}C\{^{1}H\}$ NMR (101 MHz, Chloroform-d, 25°C) of trimethyl((4-(trifluoromethyl)phenyl)ethynyl)silane (**3n**).

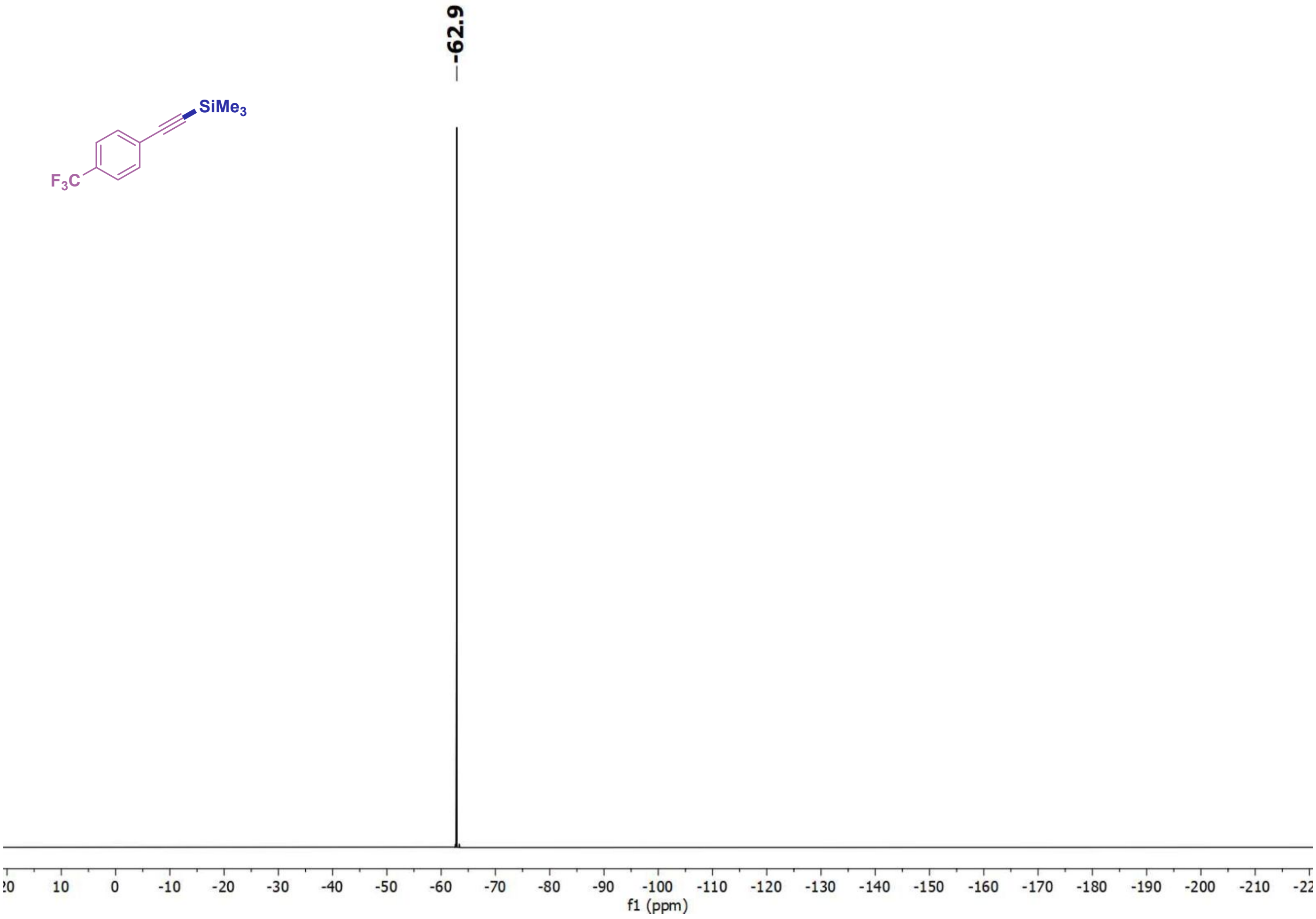


Figure S45. $^{19}$F NMR (377 MHz, Chloroform-d, 25°C) of trimethyl((4-(trifluoromethyl)phenyl)ethynyl)silane (**3n**).

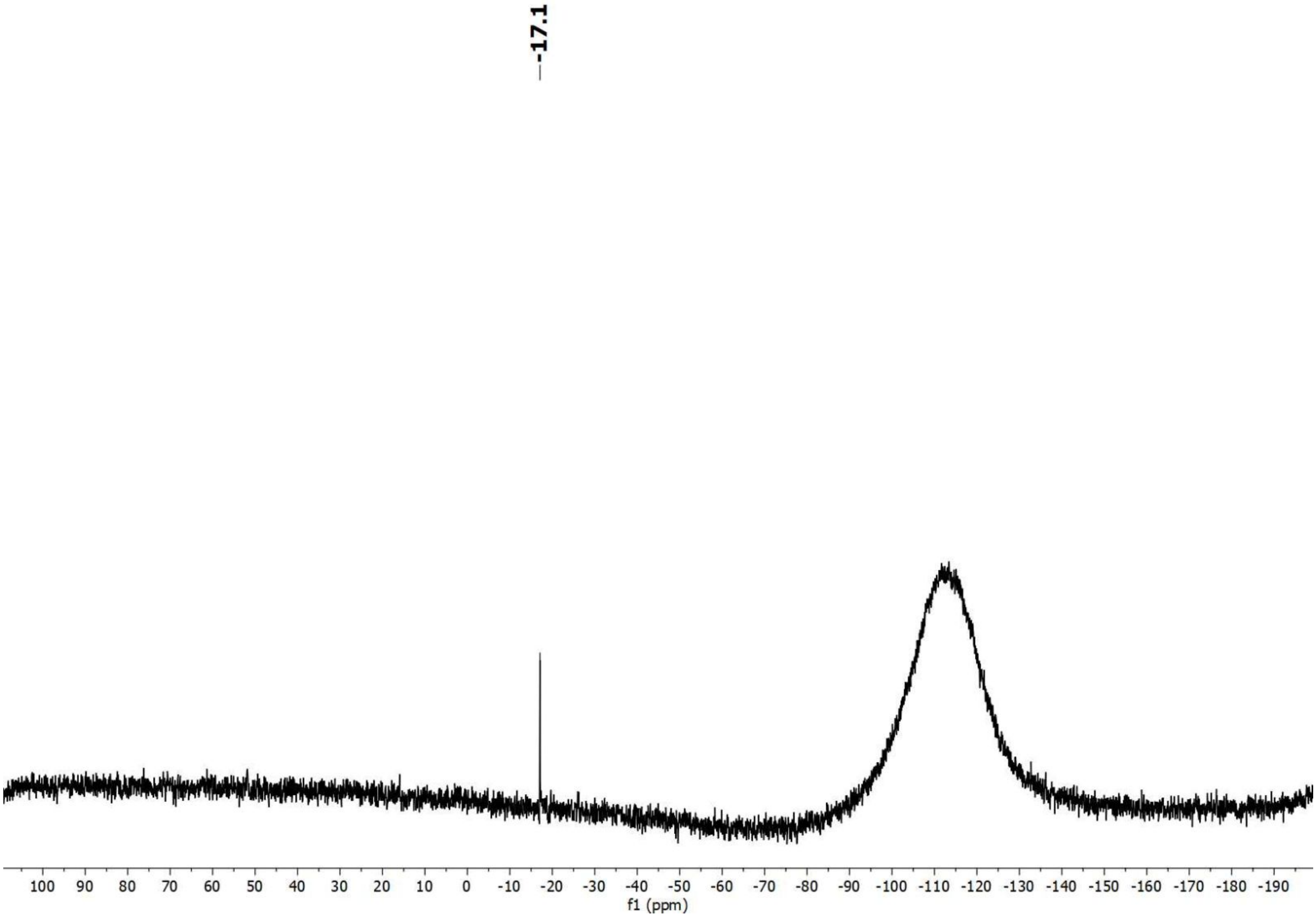


Figure S46. $^{29}$Si NMR (79 MHz, Chloroform-d, 25°C) of trimethyl((4-(trifluoromethyl)phenyl)ethynyl)silane (**3n**).

Trimethyl((3-nitrophenyl)ethynyl)silane (**3o**)

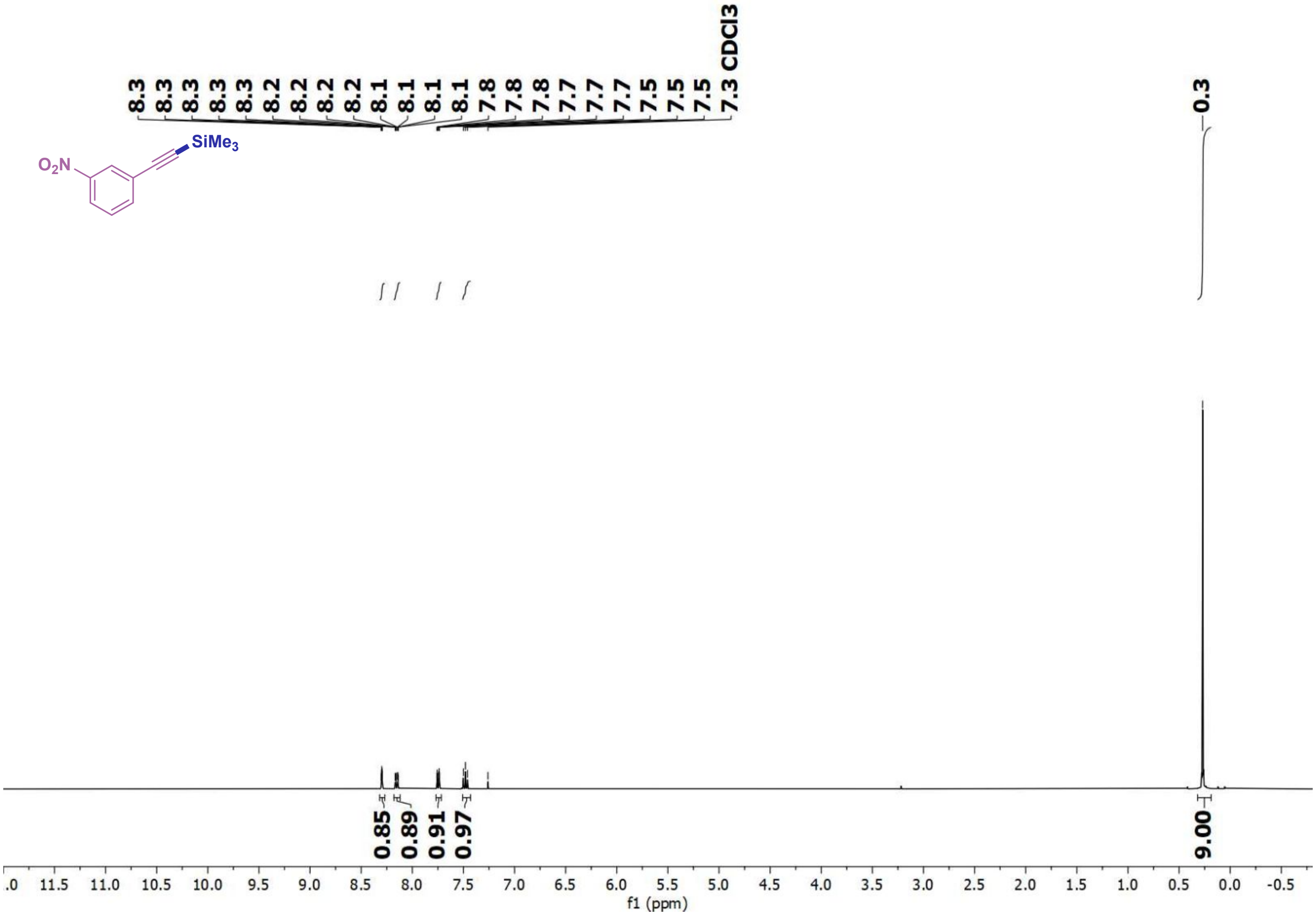


Figure S47. $^{1}$H NMR (400 MHz, Chloroform-d, 25°C) of trimethyl((3-nitrophenyl)ethynyl)silane (**3o**).

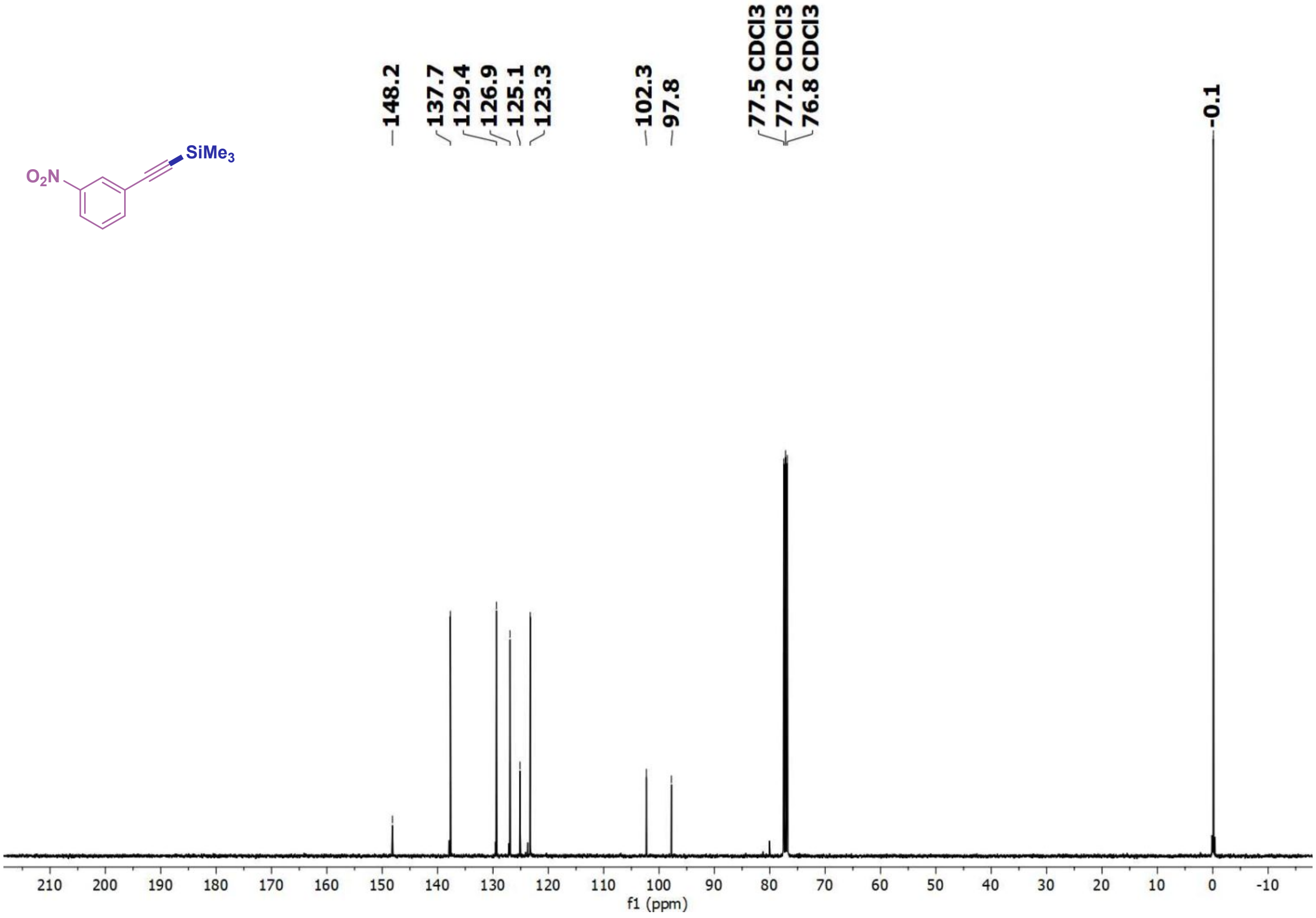


Figure S48. $^{13}$C{$^{1}$H} NMR (101 MHz, Chloroform-d, 25°C) of trimethyl((3-nitrophenyl)ethynyl)silane (**3o**).

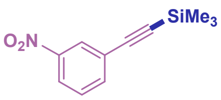

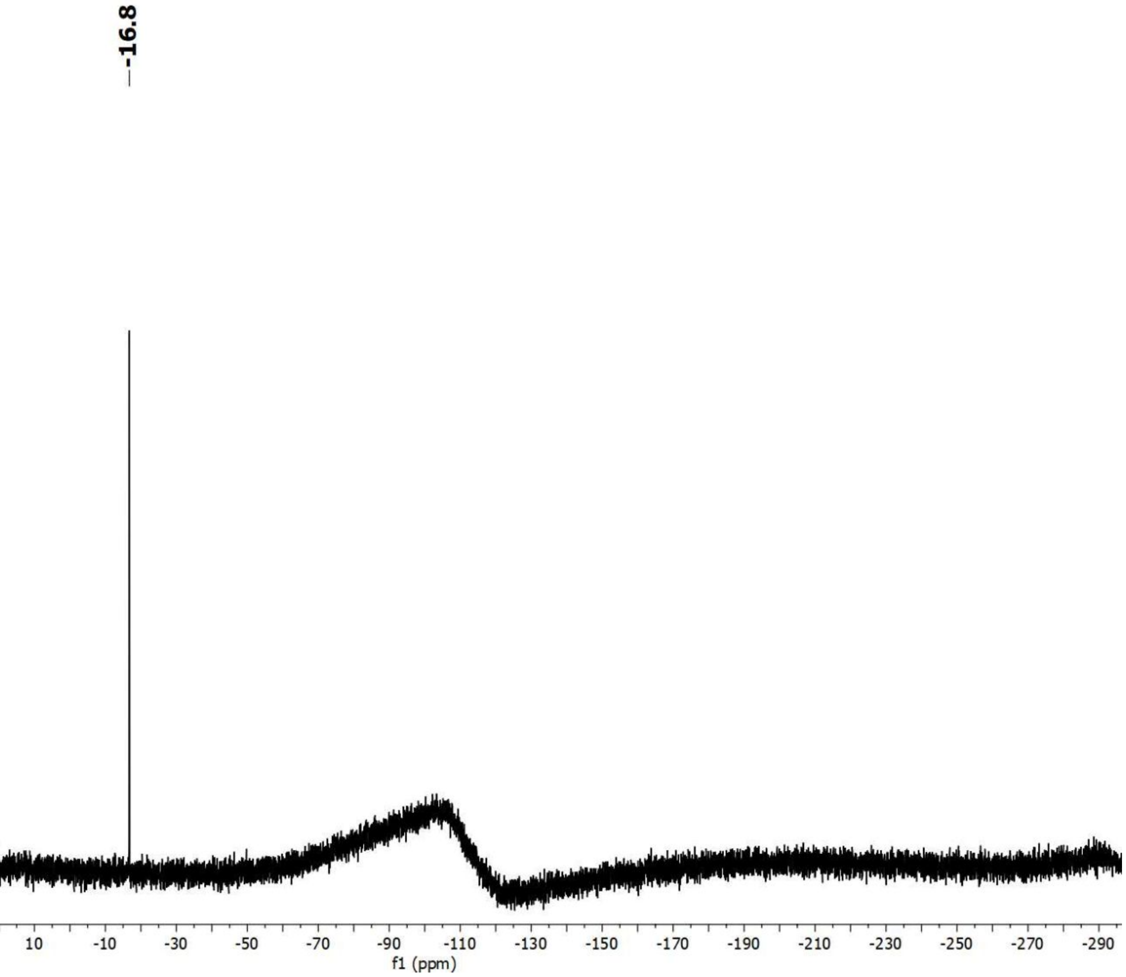


Figure S49. $^{29}$Si NMR (79 MHz, Chloroform-d, 25°C) of trimethyl((3-nitrophenyl)ethynyl)silane (**3o**).

# Trimethyl((4-nitrophenyl)ethynyl)silane (**3p**)

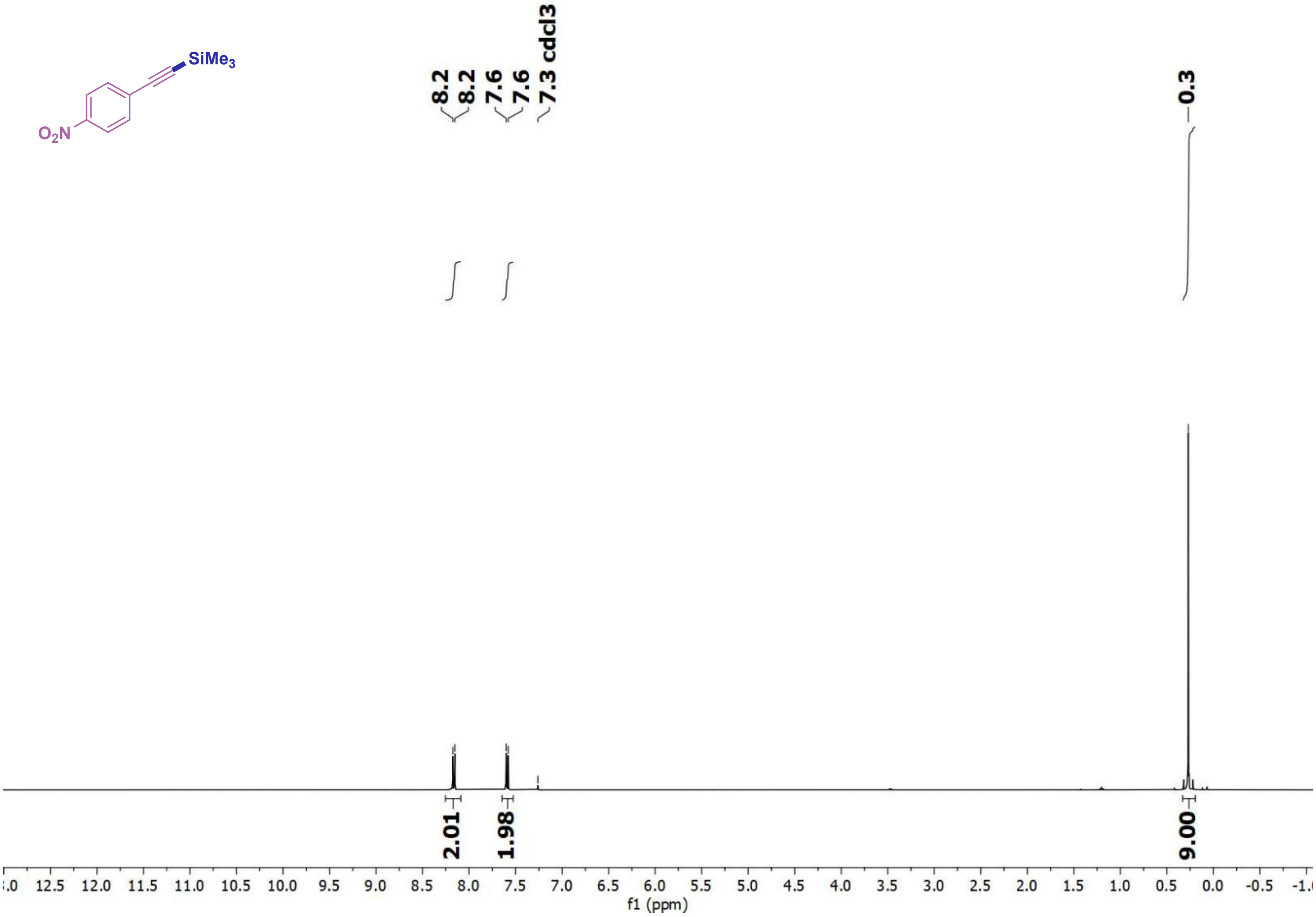


Figure S50. $^{1}$H NMR (400 MHz, Chloroform-d, 25°C) of trimethyl((4-nitrophenyl)ethynyl)silane (**3p**).

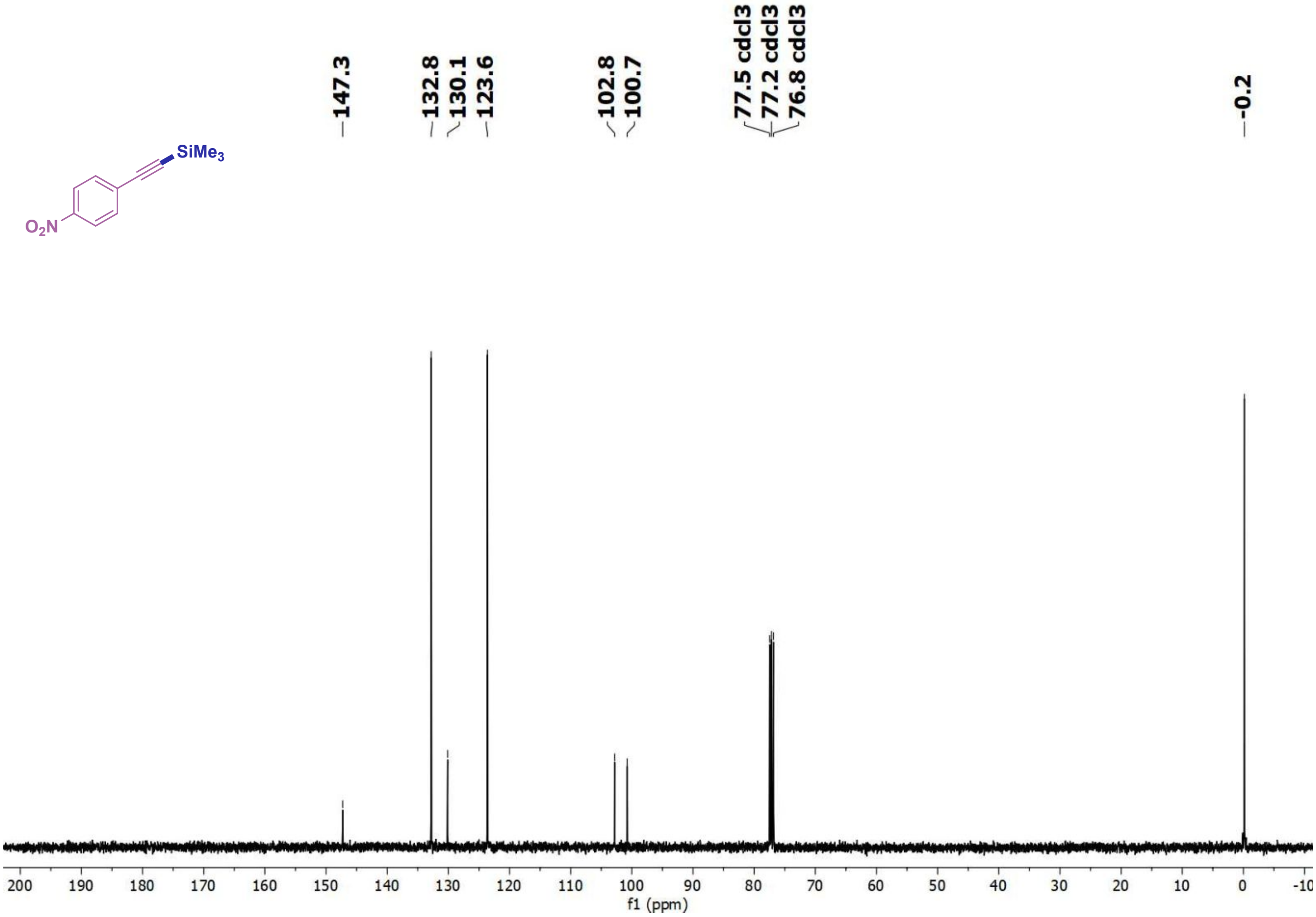


Figure S51. $^{13}$C{$^{1}$H} NMR (101 MHz, Chloroform-d, 25°C) of trimethyl((4-nitrophenyl)ethynyl)silane (**3p**).

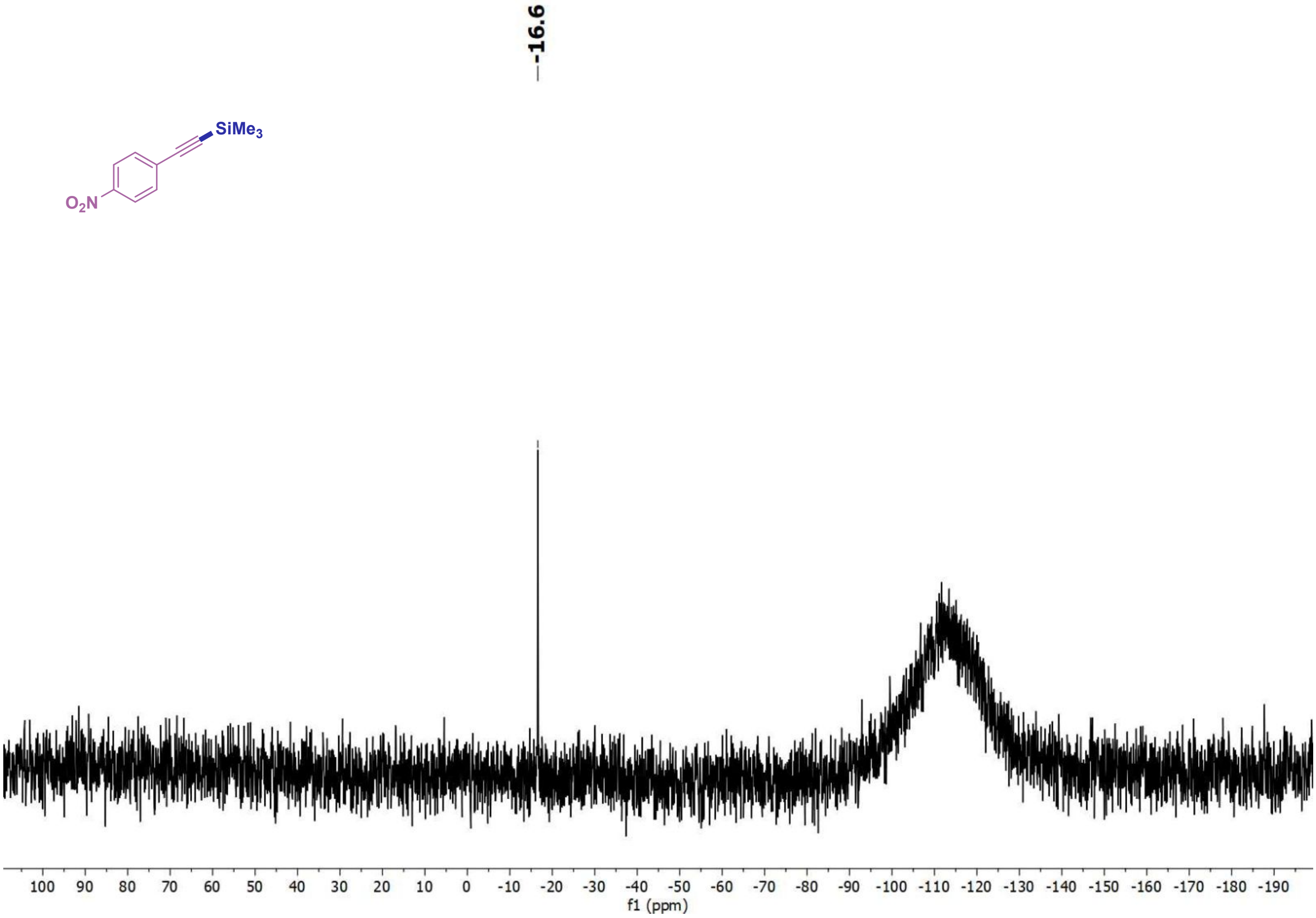


Figure S52. $^{29}$Si NMR (79 MHz, Chloroform-d, 25°C) of trimethyl((4-nitrophenyl)ethynyl)silane (**3p**).

4-((Trimethylsilyl)ethynyl)benzonitrile (**3q**)

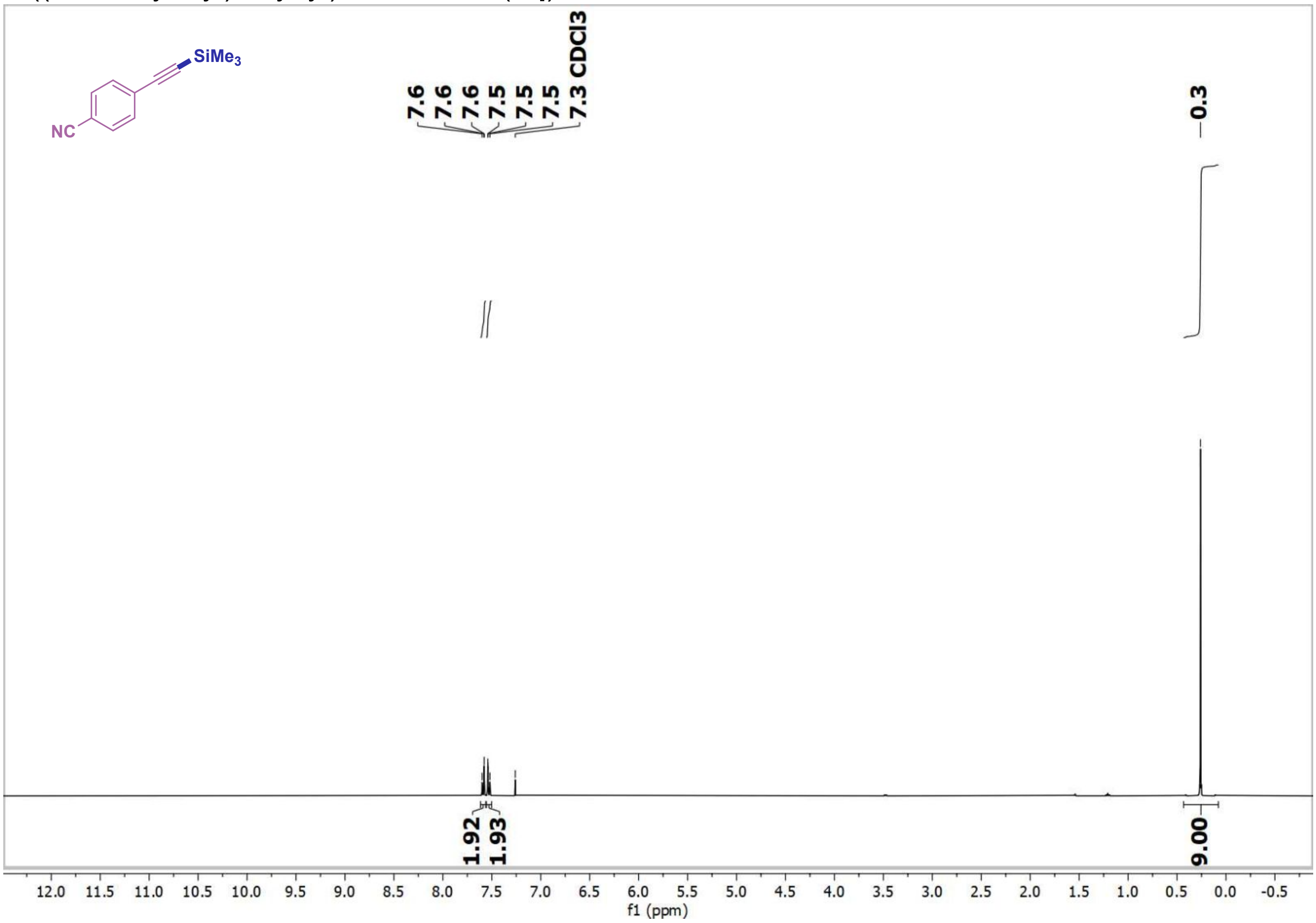


Figure S53. ${}^{1}H$ NMR (400 MHz, Chloroform-d, 25°C) of 4-((trimethylsilyl)ethynyl)benzonitrile (**3q**).

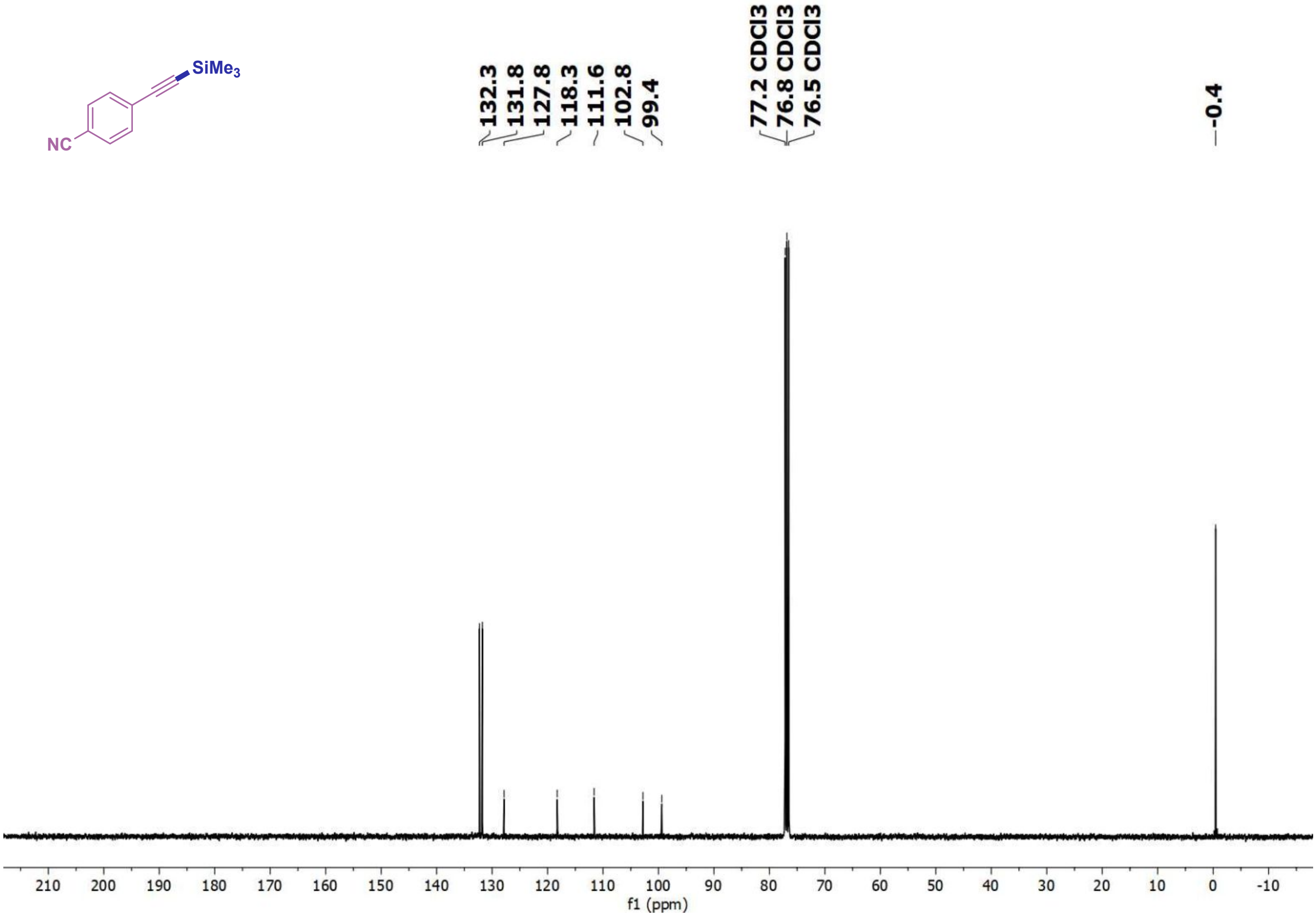


Figure S54. ${}^{13}C\{{}^{1}H\}$ NMR (101 MHz, Chloroform-d, 25°C) of 4-((trimethylsilyl)ethynyl)benzonitrile (**3q**).

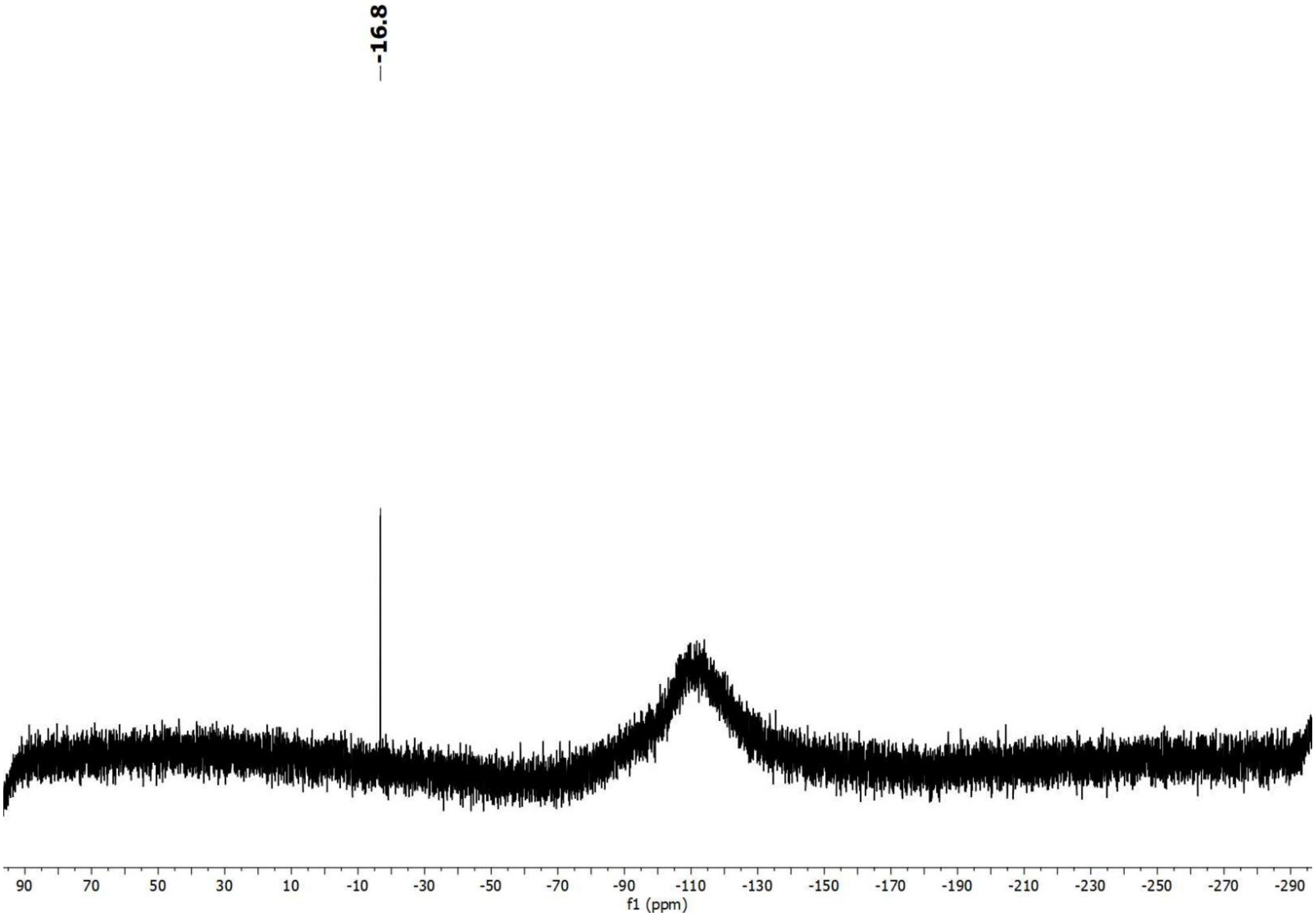


Figure S55. $^{29}Si$ NMR (79 MHz, Chloroform-d, 25°C) of 4-((trimethylsilyl)ethynyl)benzonitrile (**3q**).

2-((Trimethylsilyl)ethynyl)pyridine (**3r**)

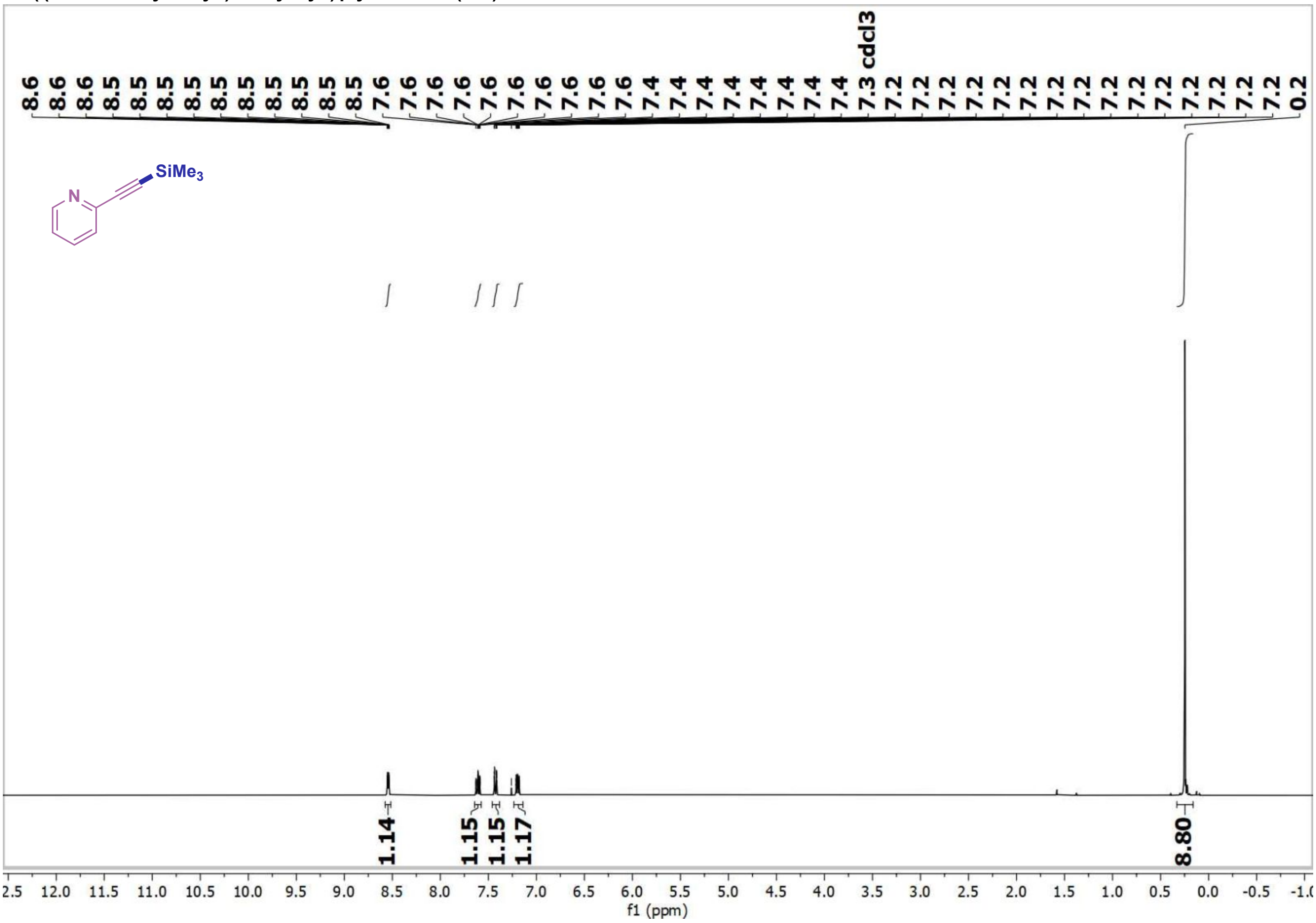


Figure S56. ¹H NMR (400 MHz, Chloroform-d, 25°C) of 2-((trimethylsilyl)ethynyl)pyridine (**3r**).

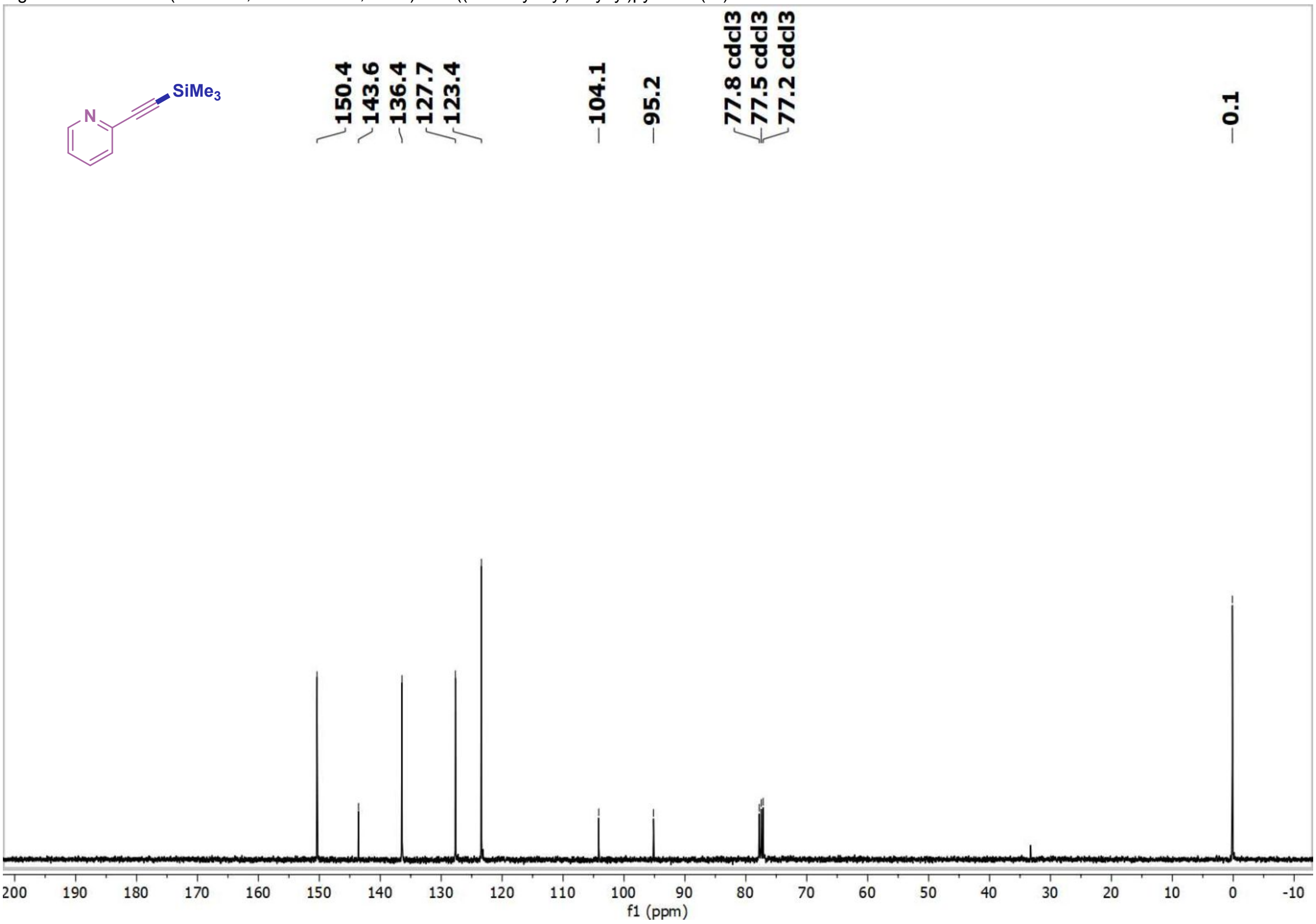


Figure S57. $^{13}C\{^1H\}$ NMR (101 MHz, Chloroform-d, 25°C) of 2-((trimethylsilyl)ethynyl)pyridine (**3r**).

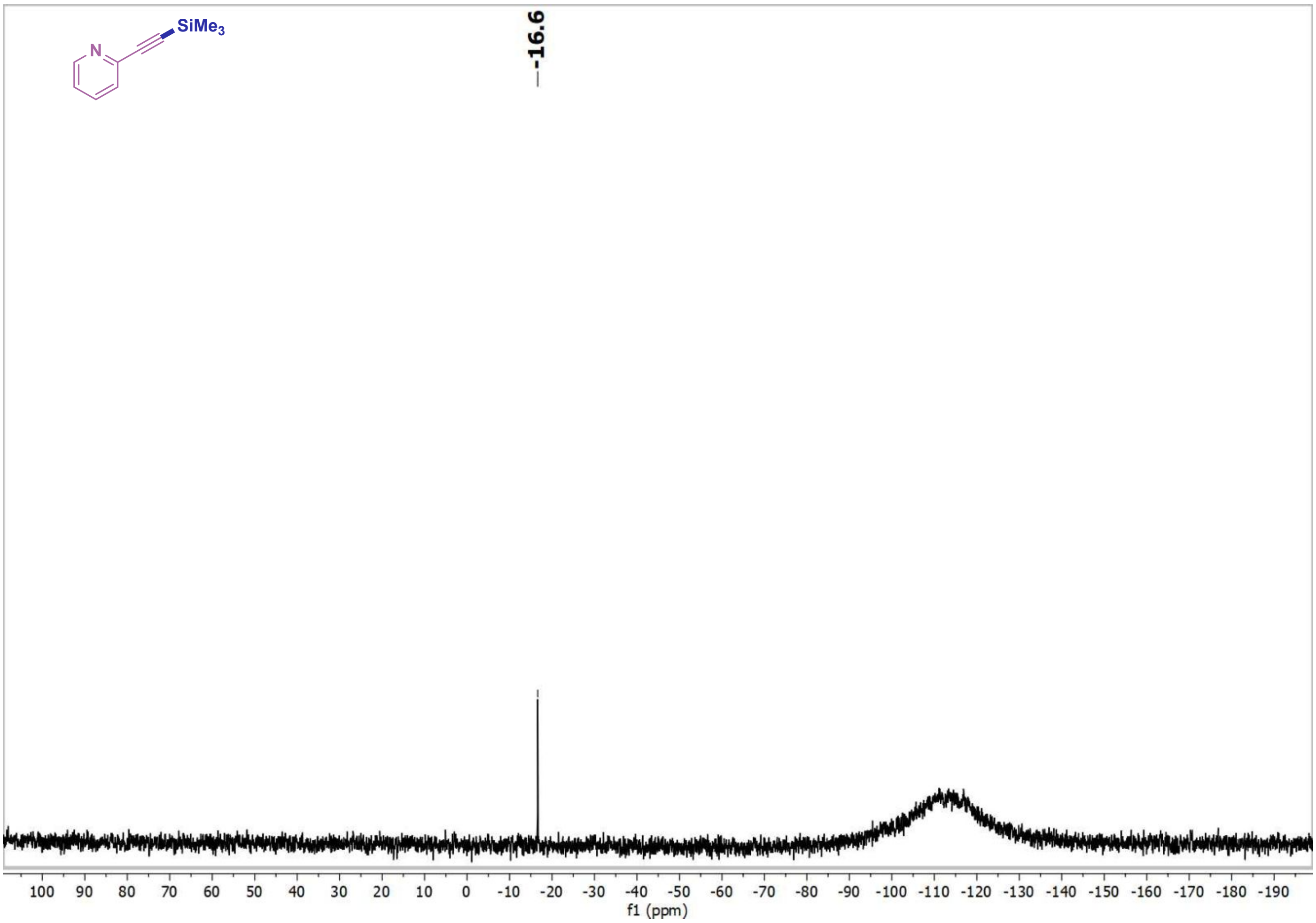


Figure S58. $^{29}Si$ NMR (79 MHz, Chloroform-d, 25°C) of 2-((trimethylsilyl)ethynyl)pyridine (**3r**).

# Trimethyl(thiophen-3-ylethynyl)silane (**3s**)

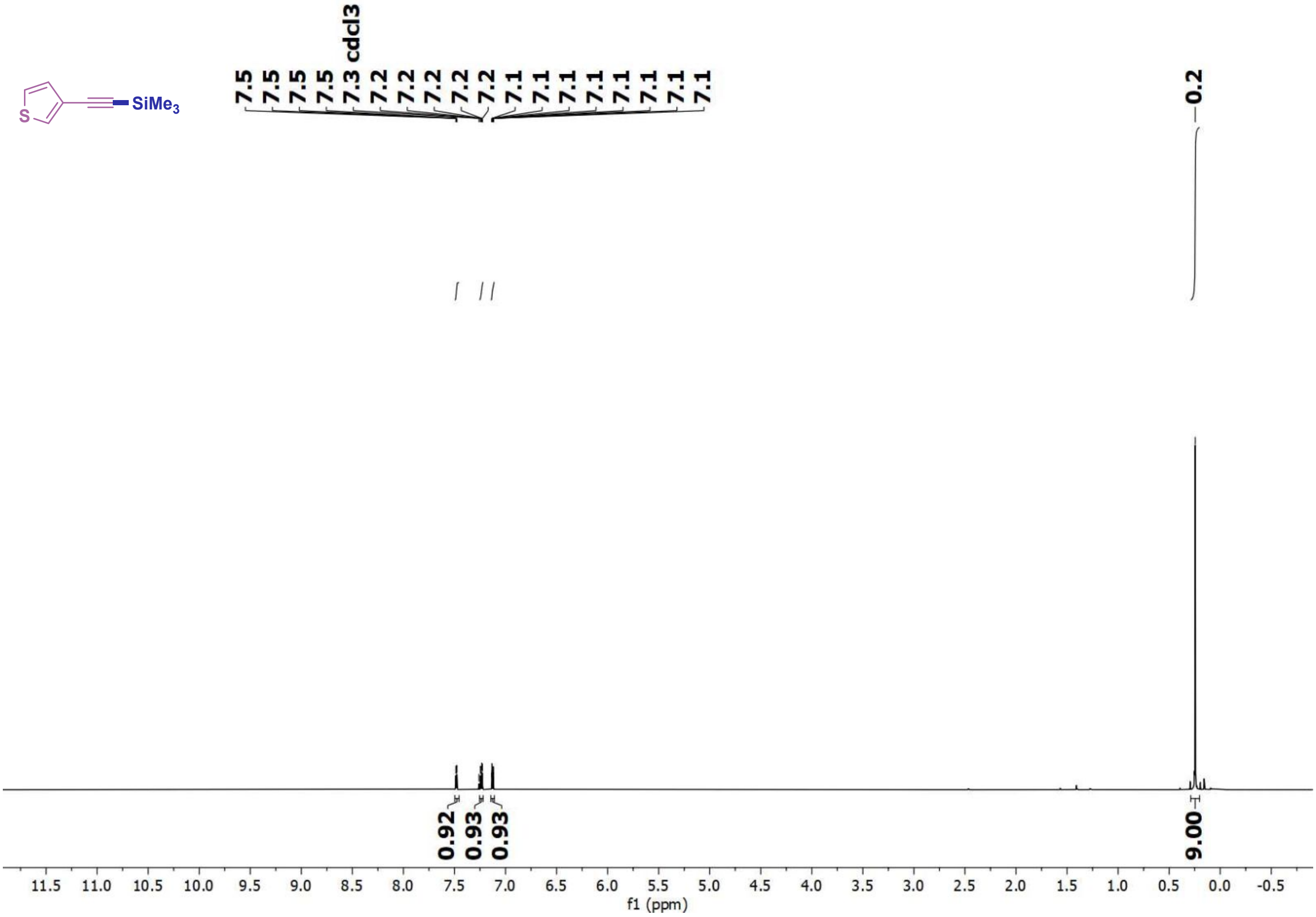


Figure S59. $^{1}H$ NMR (400 MHz, Chloroform-d, 25°C) of trimethyl(thiophen-3-ylethynyl)silane (**3s**).

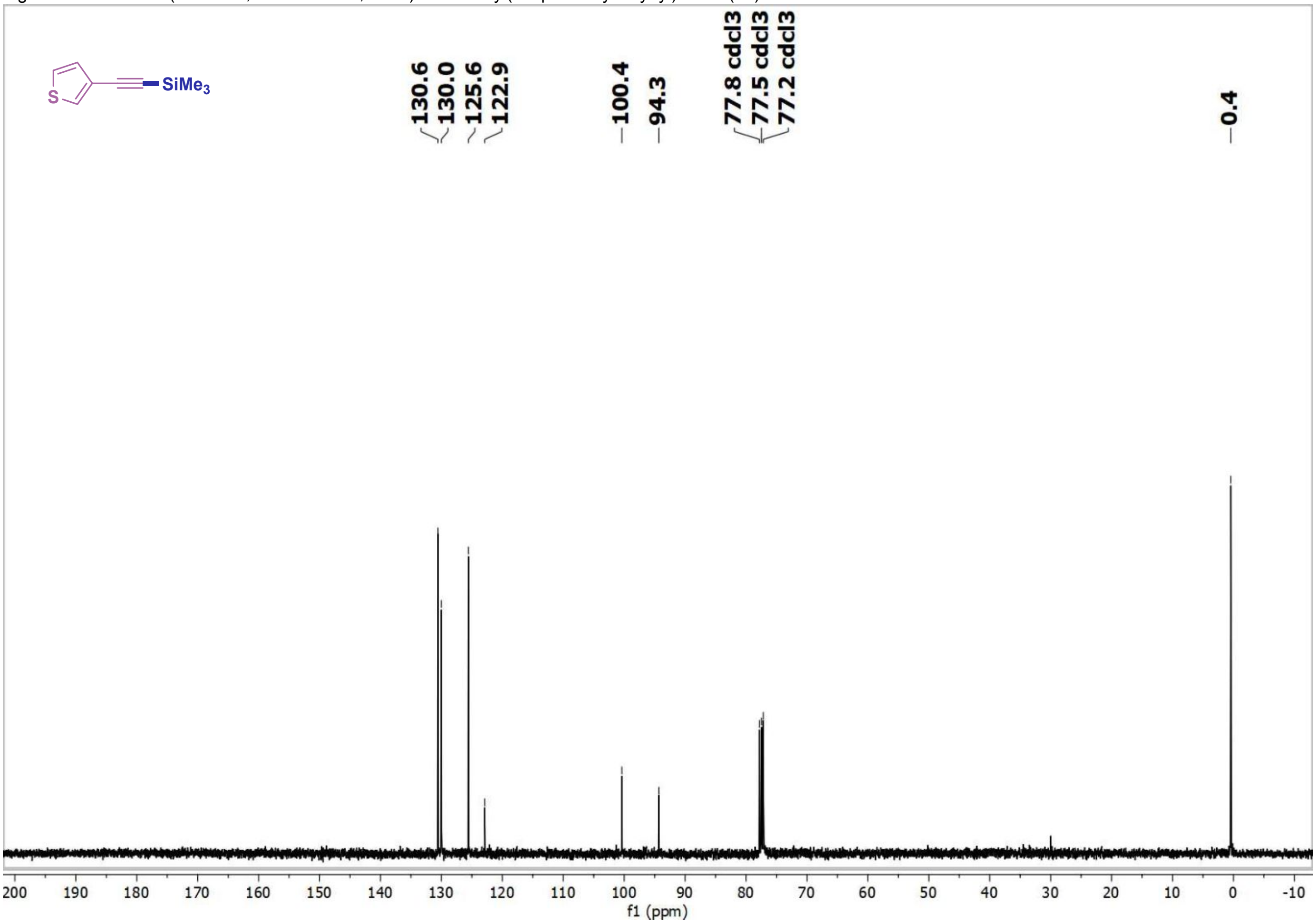


Figure S60. $^{13}C\{^{1}H\}$ NMR (101 MHz, Chloroform-d, 25°C) of trimethyl(thiophen-3-ylethynyl)silane (**3s**).

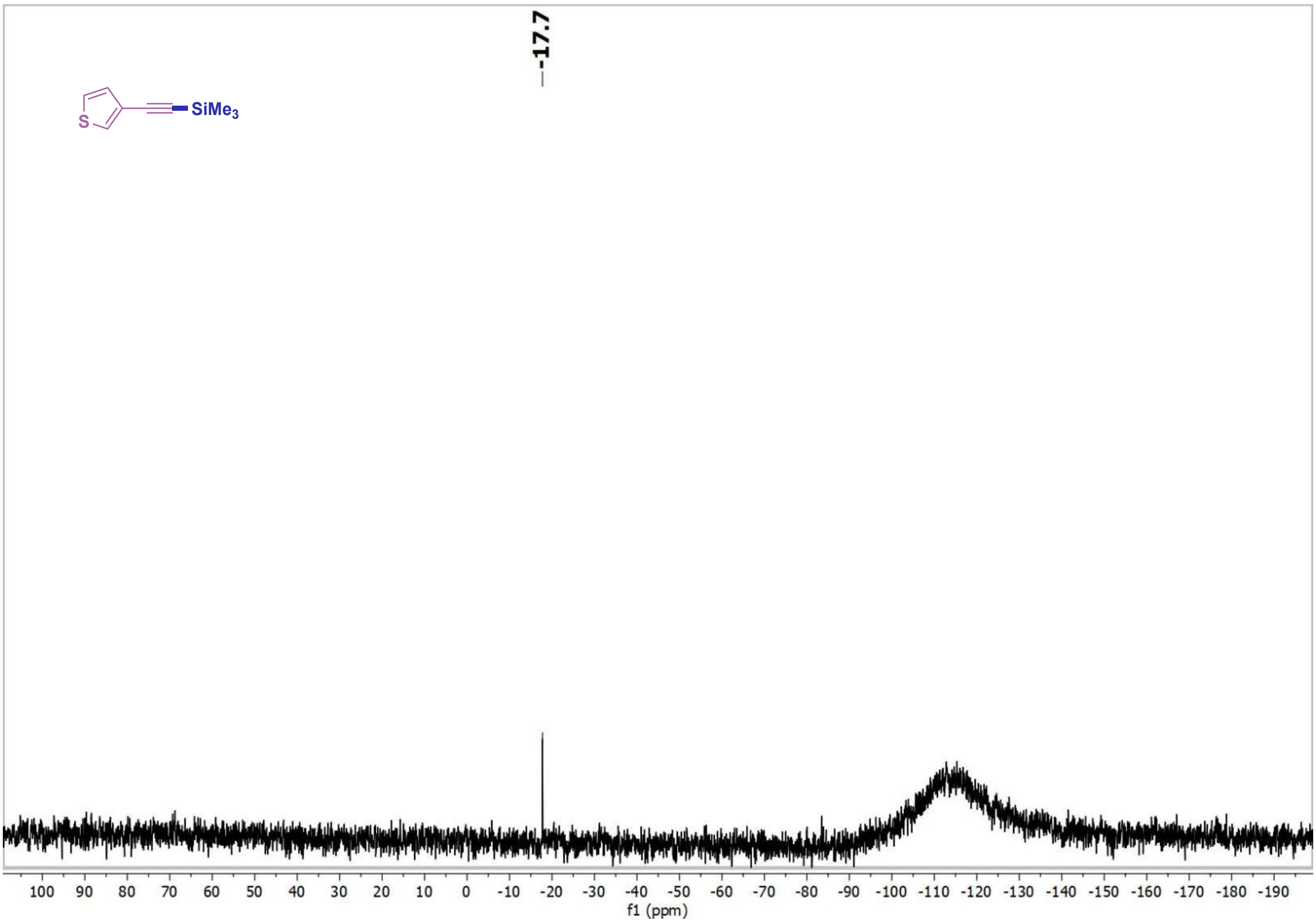


Figure S61. $^{29}Si$ NMR (79 MHz, Chloroform-d, 25°C) of trimethyl(thiophen-3-ylethynyl)silane (**3s**).

Trimethyl((phenylthio)ethynyl)silane (**3t**)

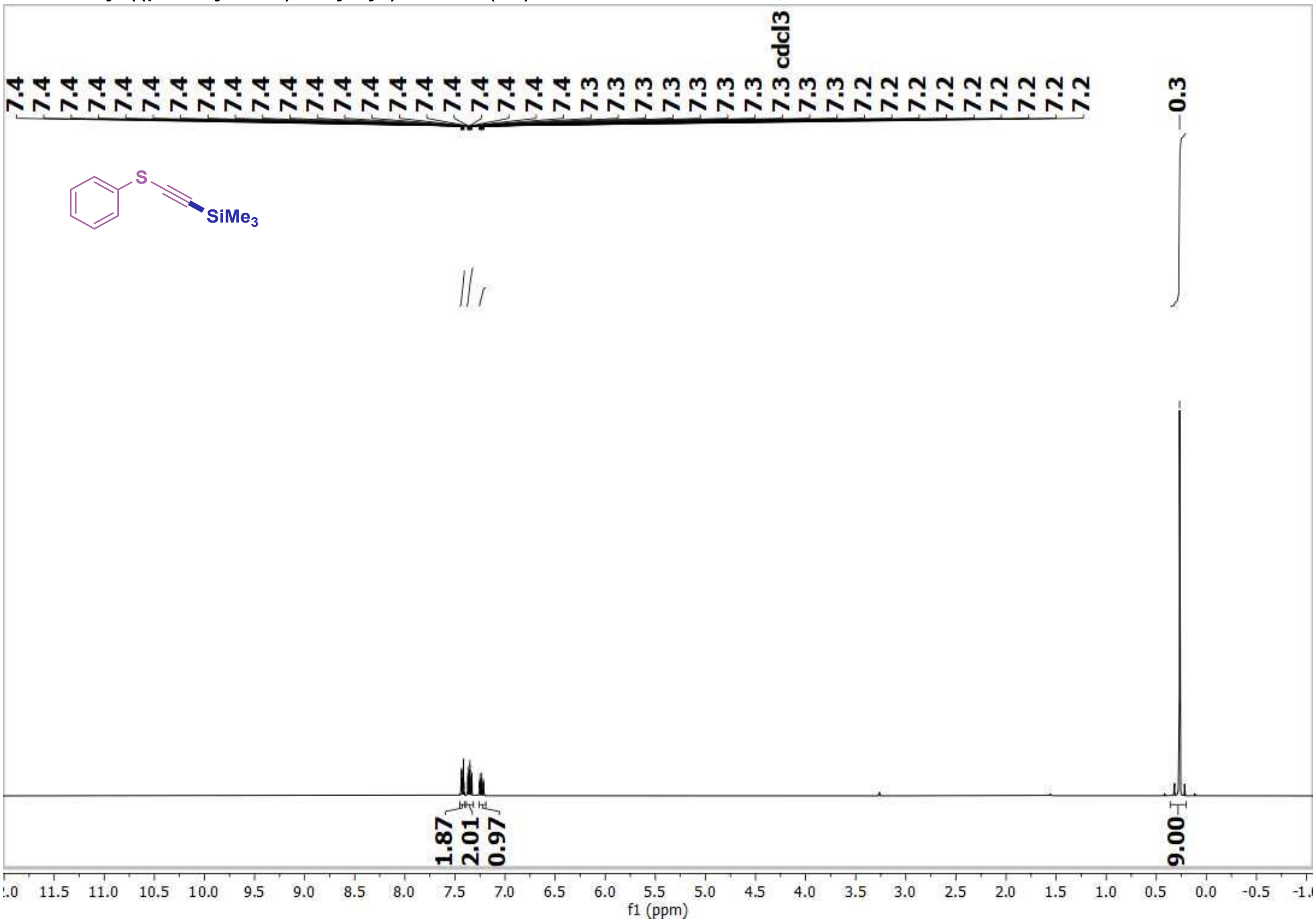

Figure S62. $^{1}$H NMR (400 MHz, Chloroform-d, 25°C) of trimethyl((phenylthio)ethynyl)silane (**3t**).

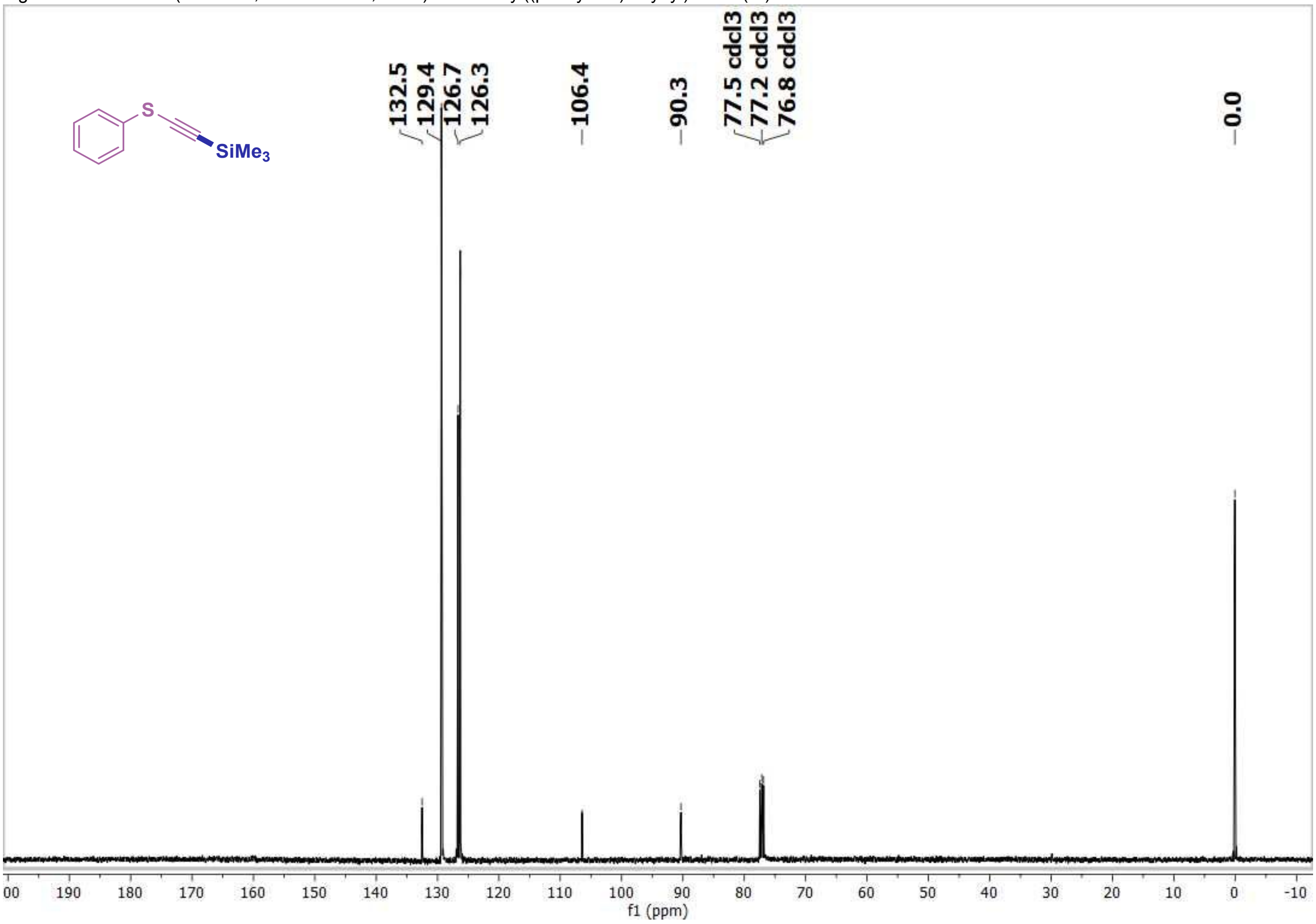

Figure S63. $^{13}$C{$^{1}$H} NMR (101 MHz, Chloroform-d, 25°C) of trimethyl((phenylthio)ethynyl)silane (**3t**).

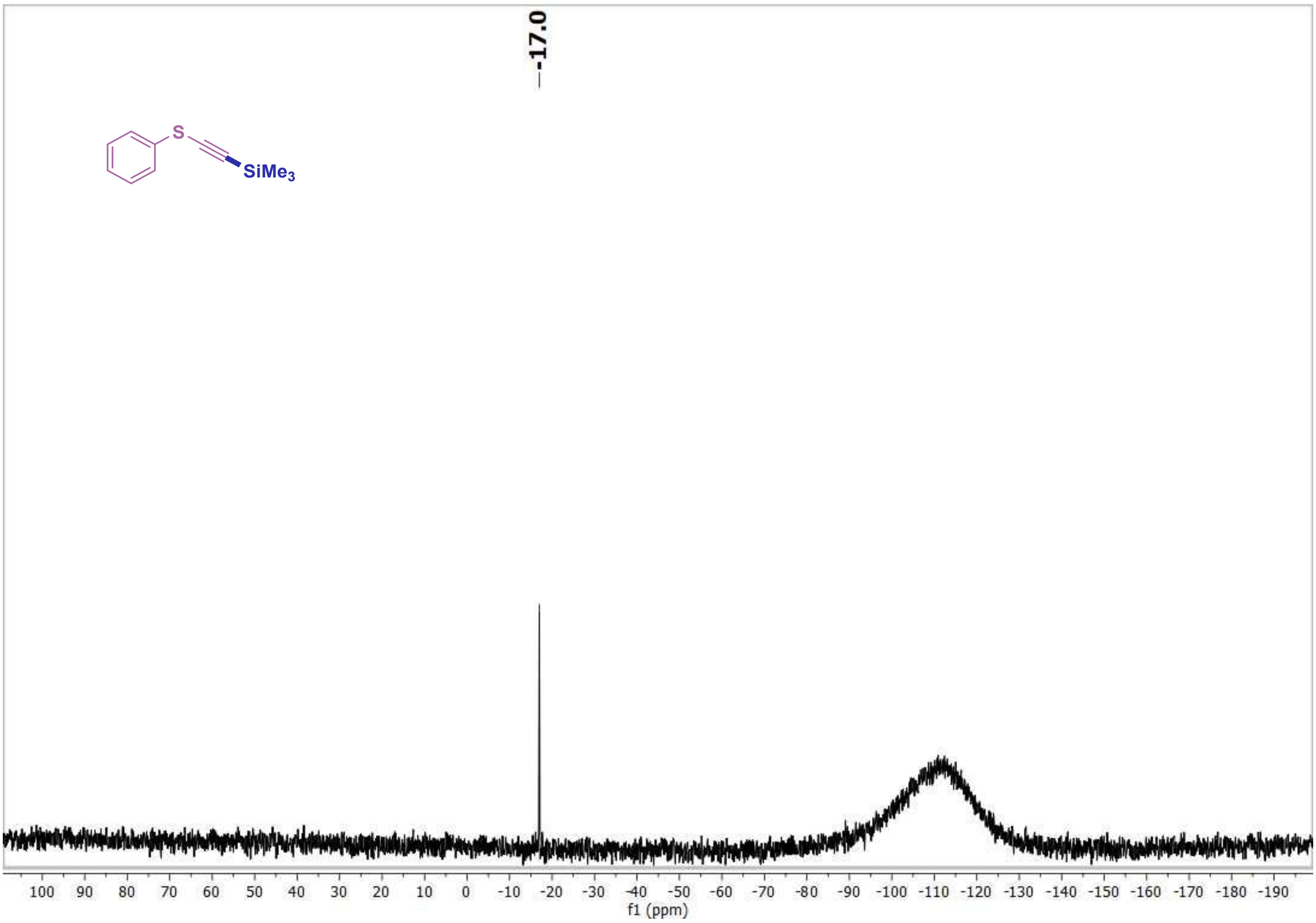


Figure S64. $^{29}Si$ NMR (79 MHz, Chloroform-d, 25°C) of trimethyl((phenylthio)ethynyl)silane (**3t**).

Tributyl((trimethylsilyl)ethynyl)silane (**3u**)

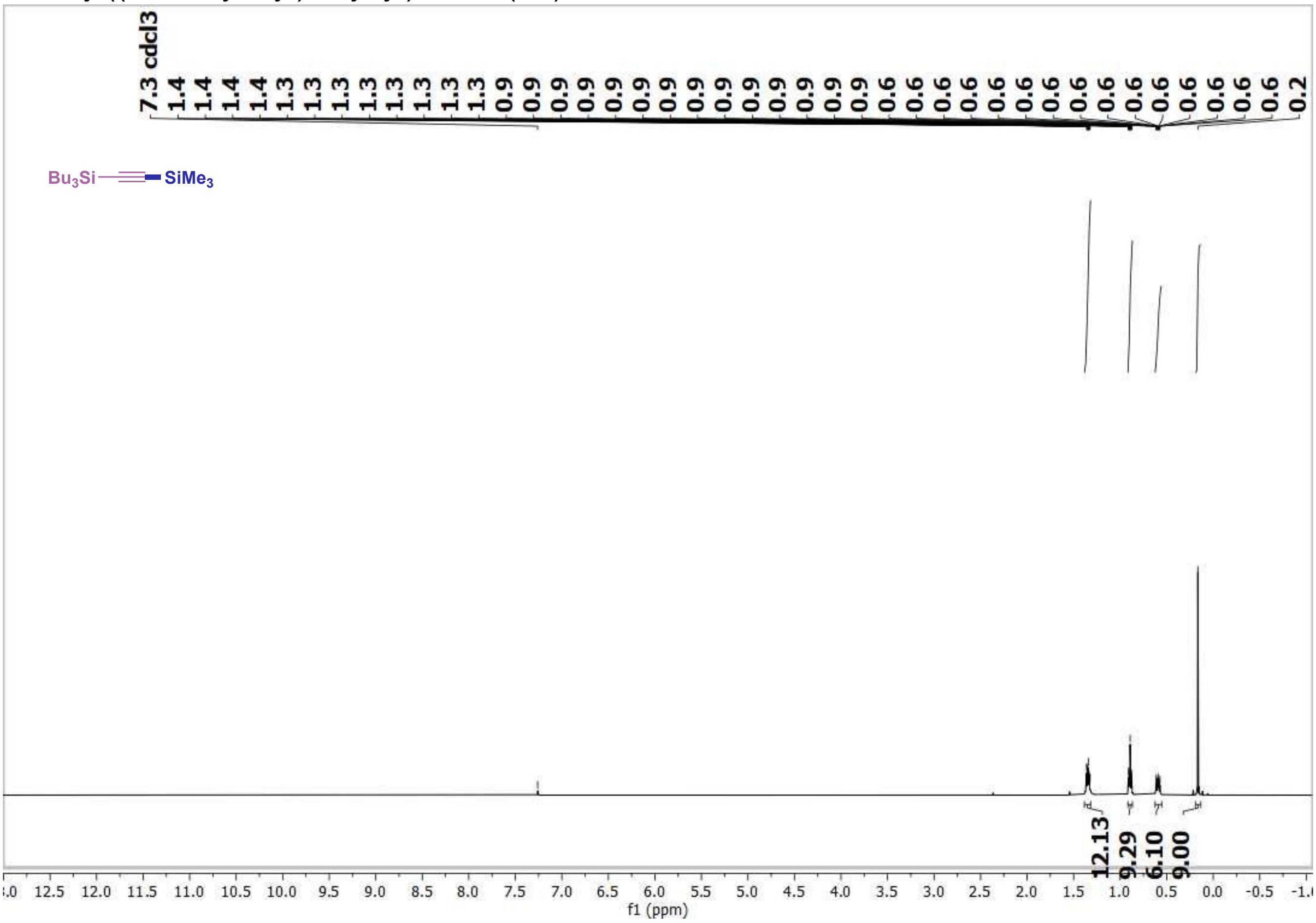


Figure S65. $^{1}$H NMR (400 MHz, Chloroform-d, 25°C) of tributyl((trimethylsilyl)ethynyl)silane (**3u**).

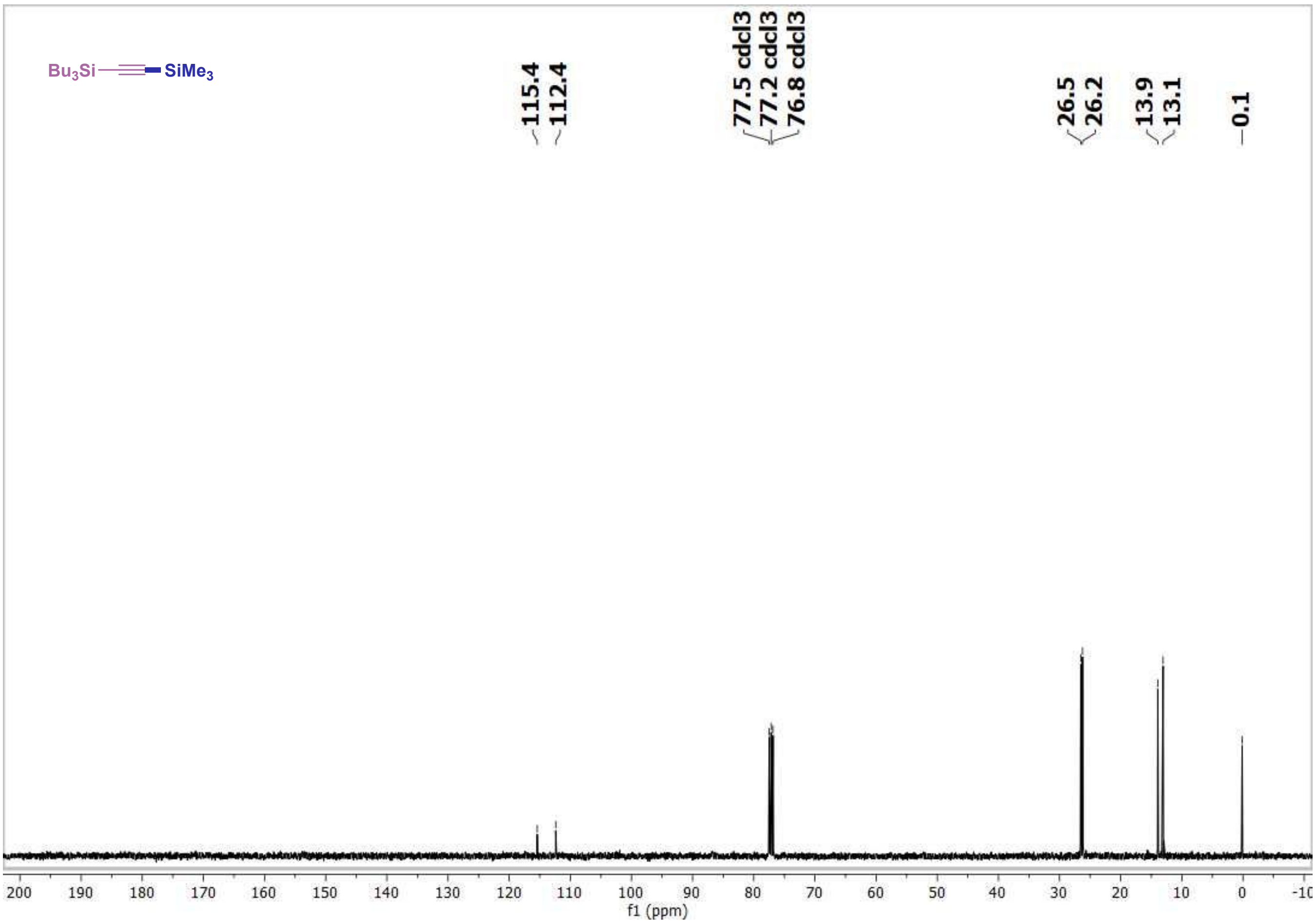


Figure S66. $^{13}C\{^{1}H\}$ NMR (101 MHz, Chloroform-d, 25°C) of tributyl((trimethylsilyl)ethynyl)silane (**3u**).

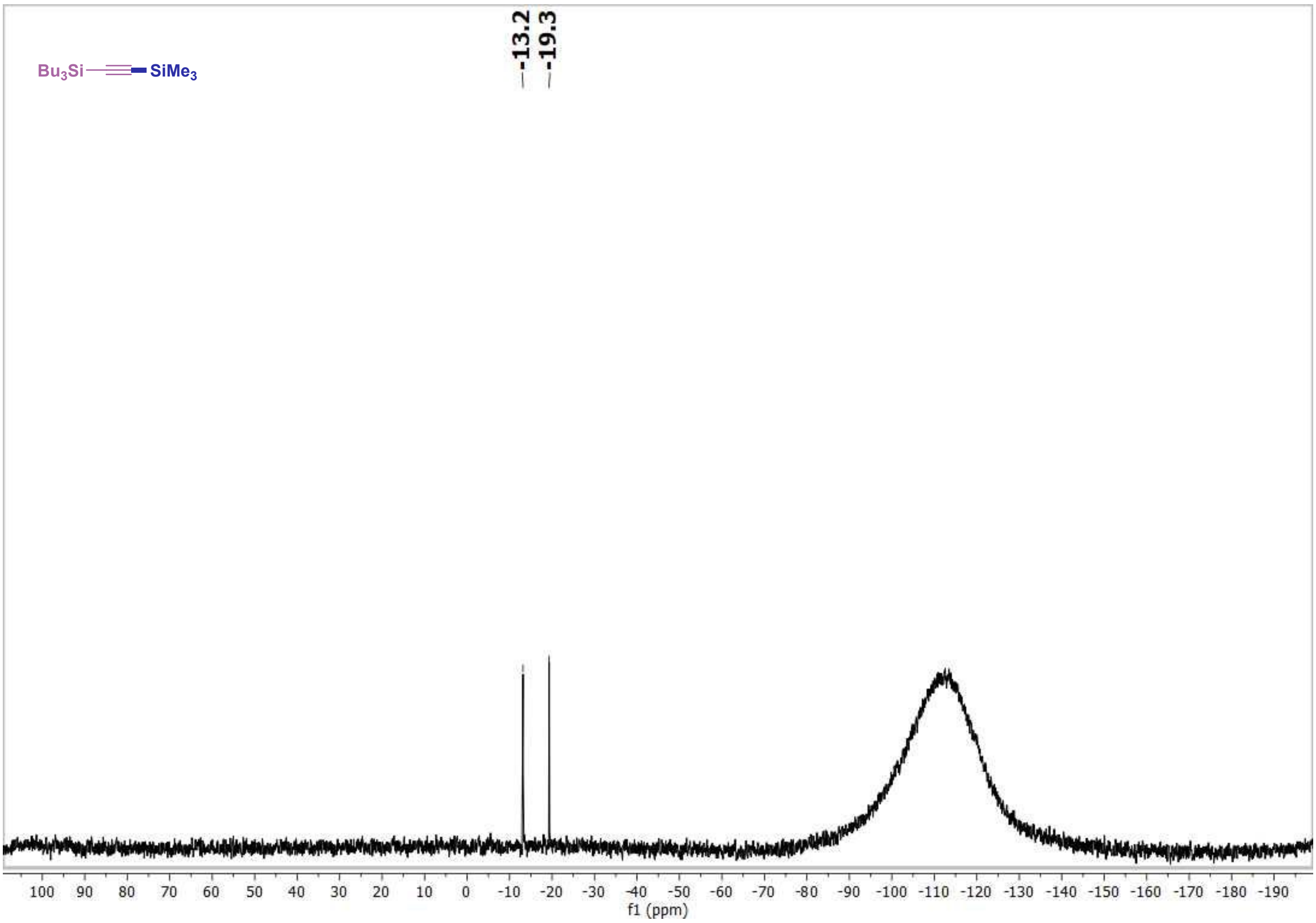


Figure S67. $^{29}Si$ NMR (79 MHz, Chloroform-d, 25°C) of tributyl((trimethylsilyl)ethynyl)silane (**3u**).

Trimethyl((triisopropylsilyl)ethynyl)silane (**3v**)

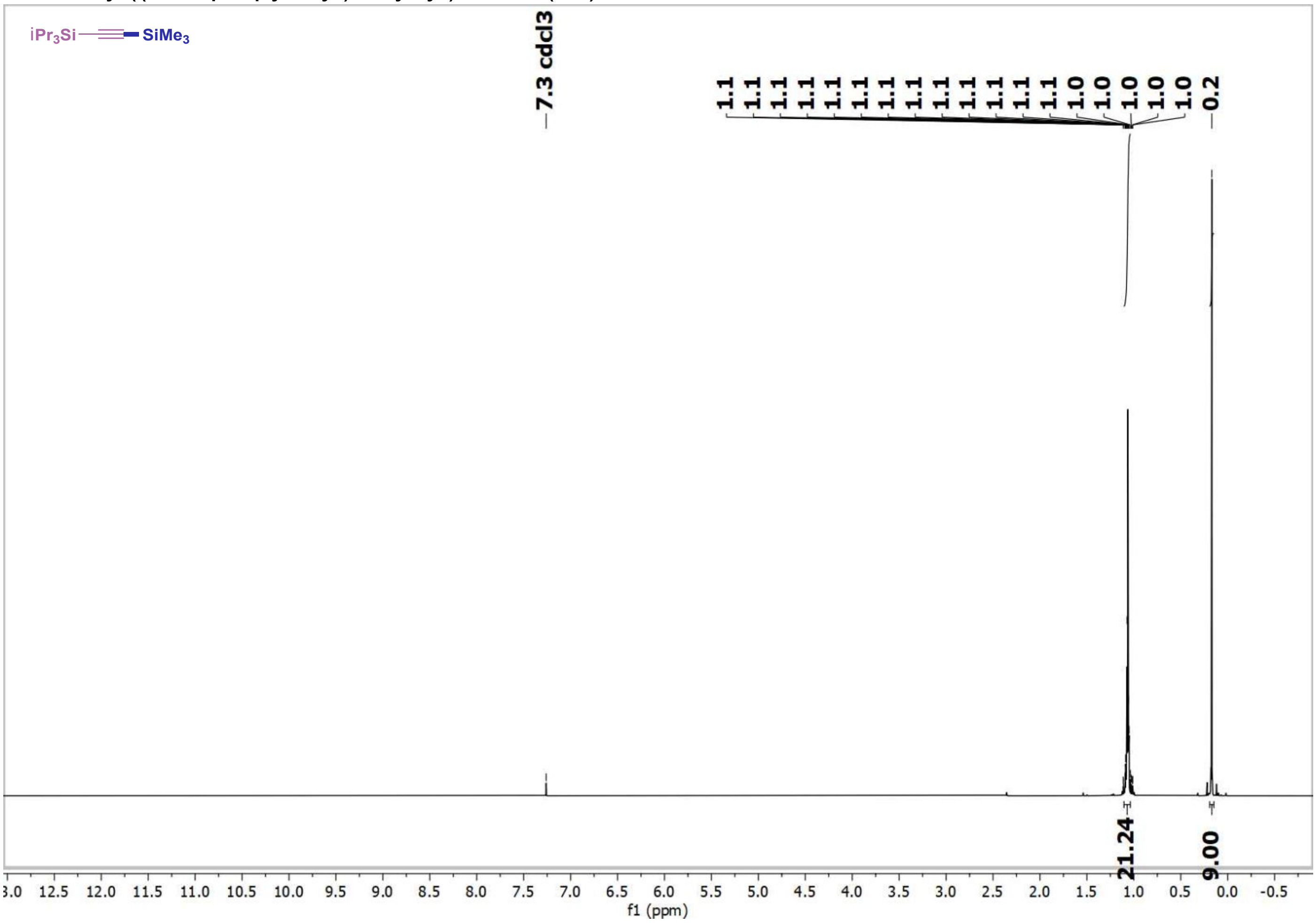


Figure S68. $^{1}H$ NMR (400 MHz, Chloroform-d, 25°C) of trimethyl((triisopropylsilyl)ethynyl)silane (**3v**).

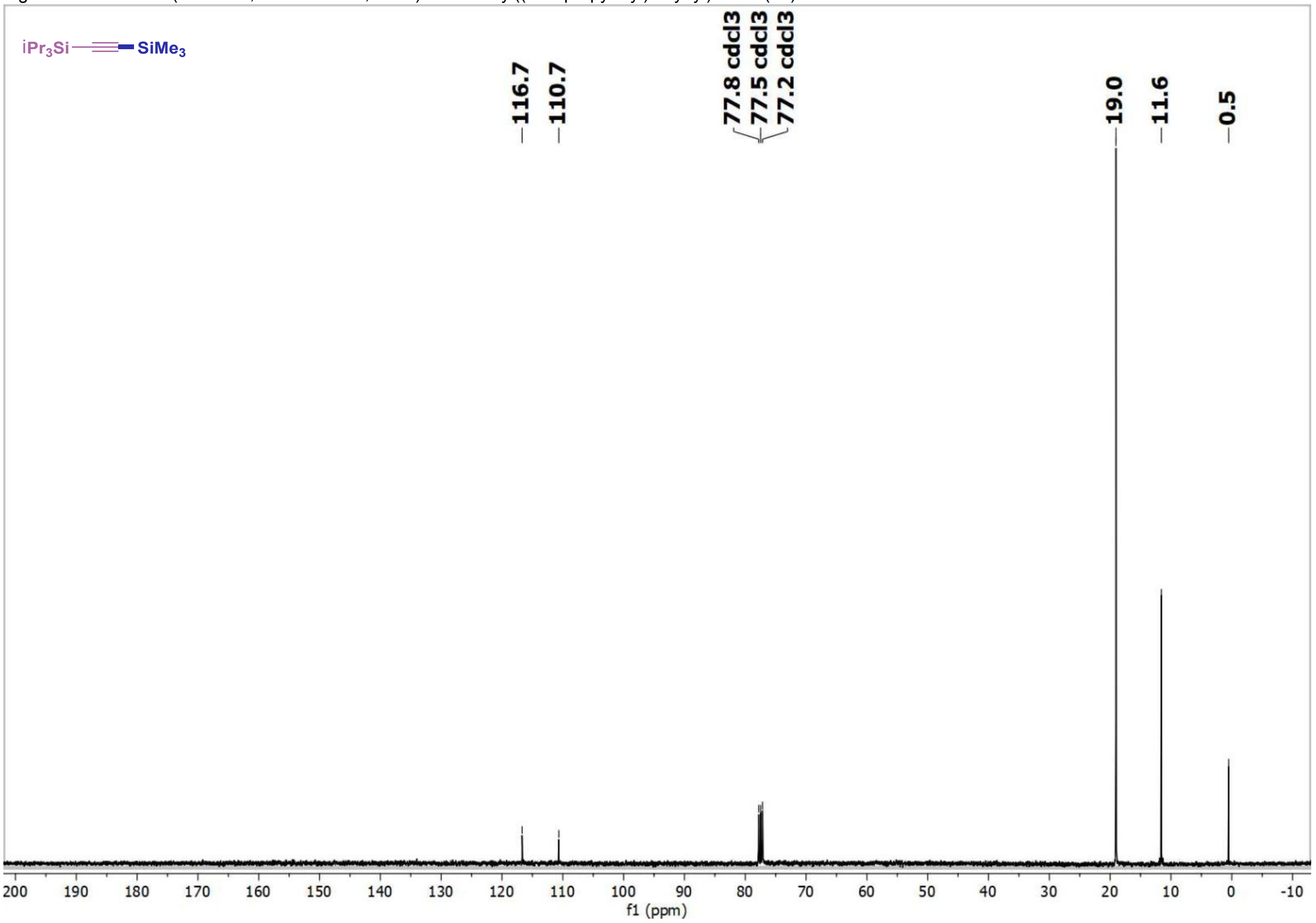


Figure S69. $^{13}C\{^{1}H\}$ NMR (101 MHz, Chloroform-d, 25°C) of trimethyl((triisopropylsilyl)ethynyl)silane (**3v**).

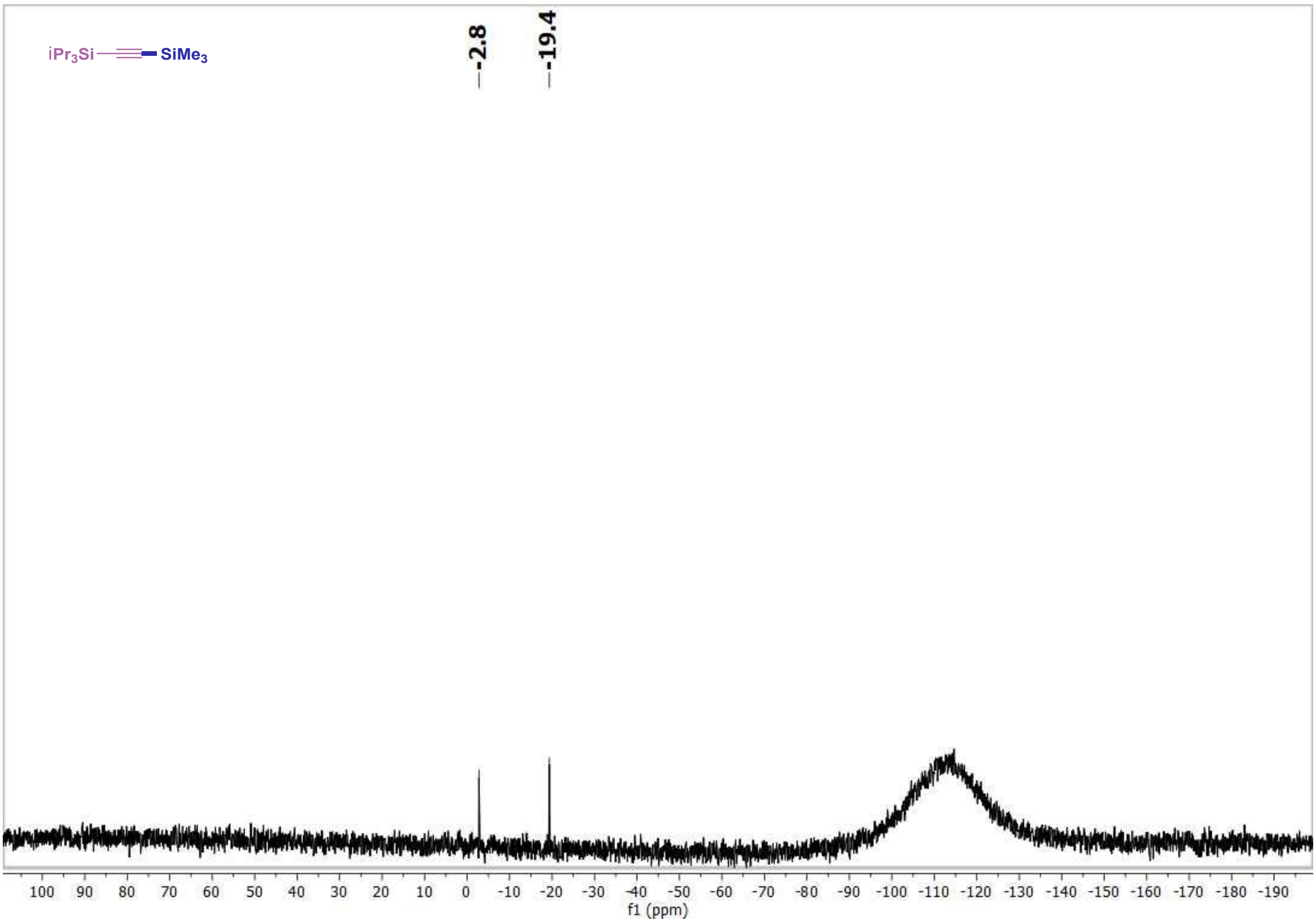


Figure S70. $^{29}$Si NMR (79 MHz, Chloroform-d, 25°C) of trimethyl((triisopropylsilyl)ethynyl)silane (**3v**).

Trimethyl((triphenylgermyl)ethynyl)silane (**3w**)

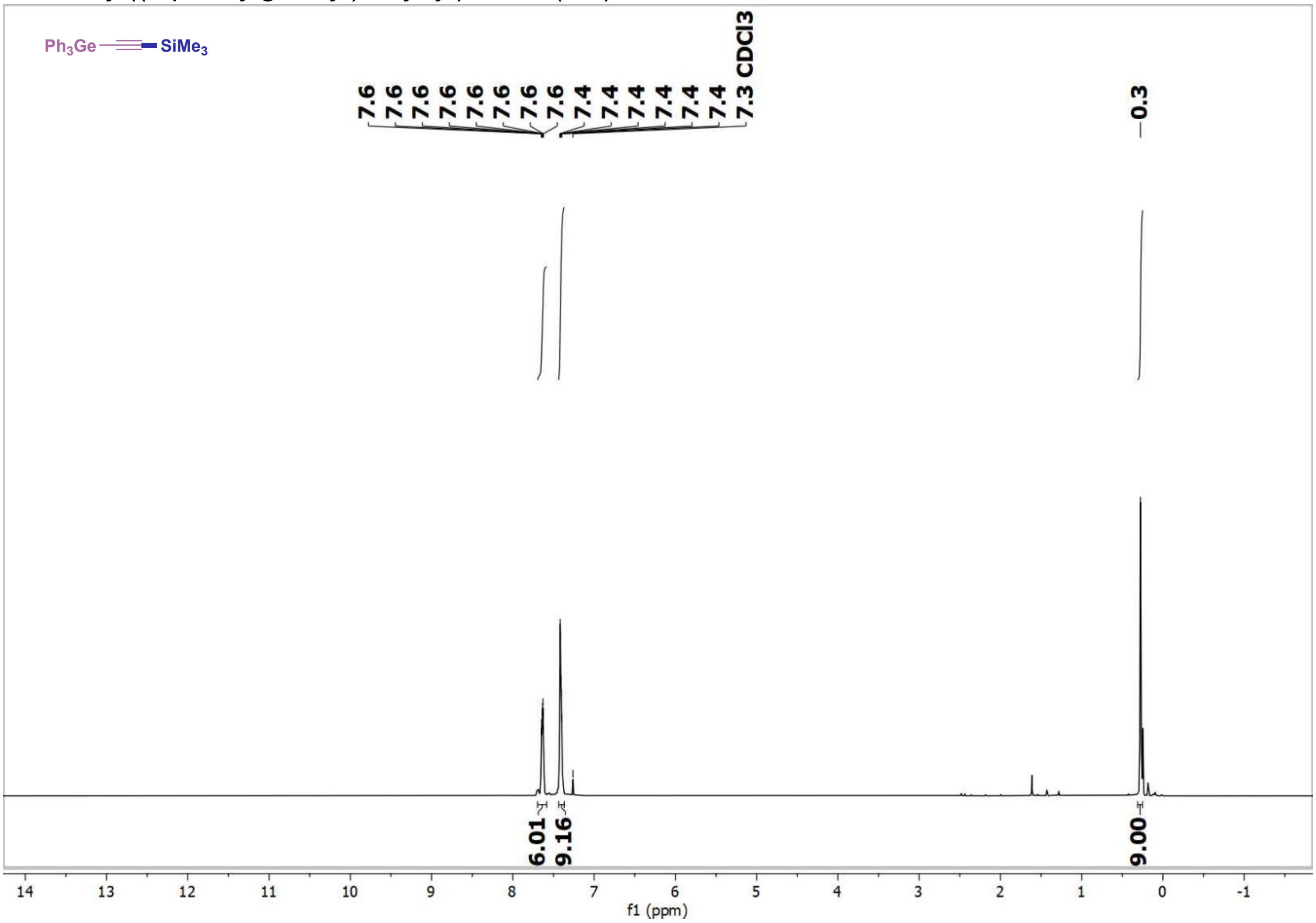


Figure S71. $^{1}H$ NMR (400 MHz, Chloroform-d, 25°C) of trimethyl((triphenylgermyl)ethynyl)silane (**3w**).

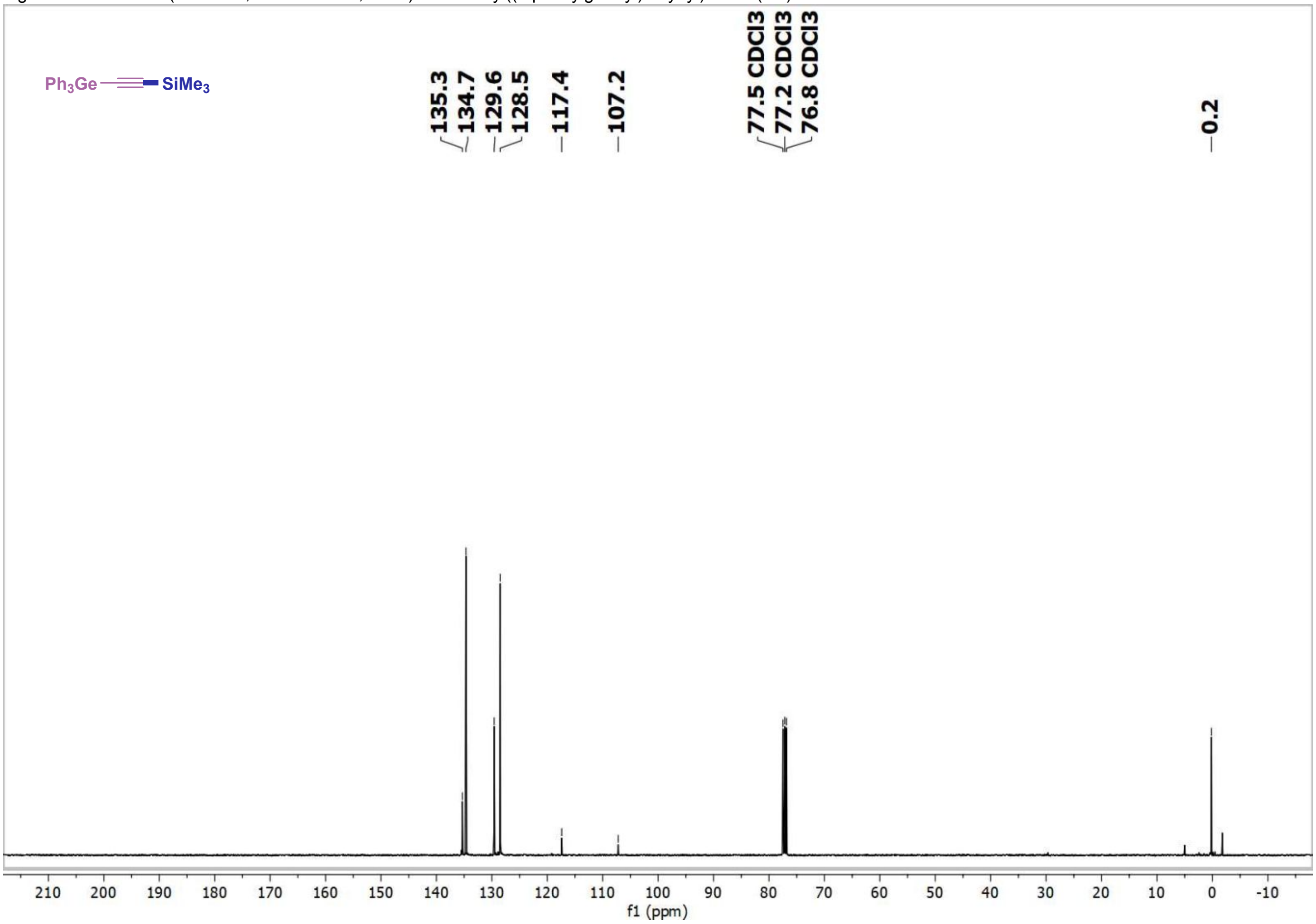


Figure S72. $^{13}C\{^{1}H\}$ NMR (101 MHz, Chloroform-d, 25°C) of trimethyl((triphenylgermyl)ethynyl)silane (**3w**).

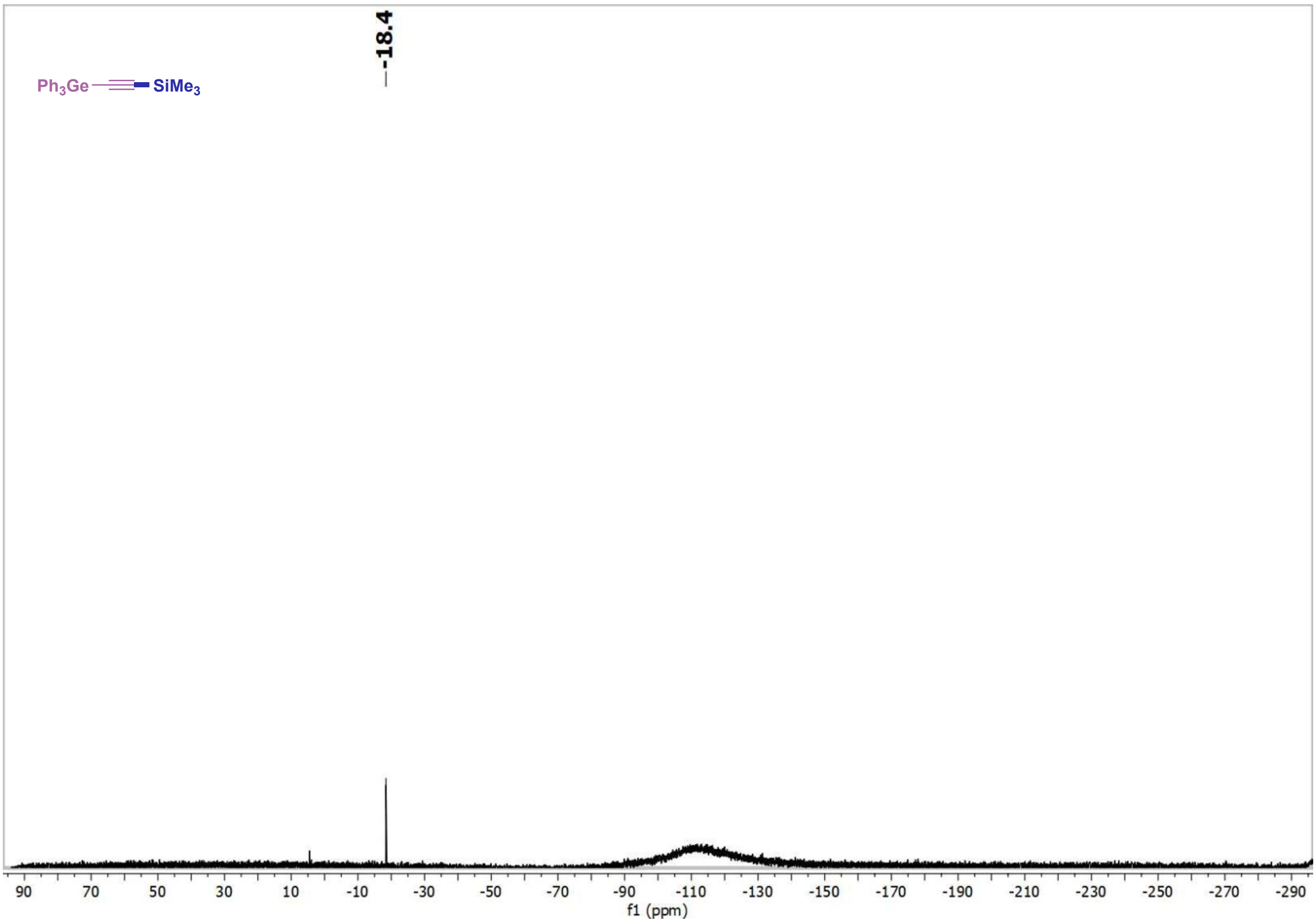


Figure S73. $^{29}$Si NMR (79 MHz, Chloroform-d, 25°C) of trimethyl((triphenylgermyl)ethynyl)silane (**3w**).

(Dimethyl(phenyl)germyl)ethynyl)trimethylsilane (**3x**)

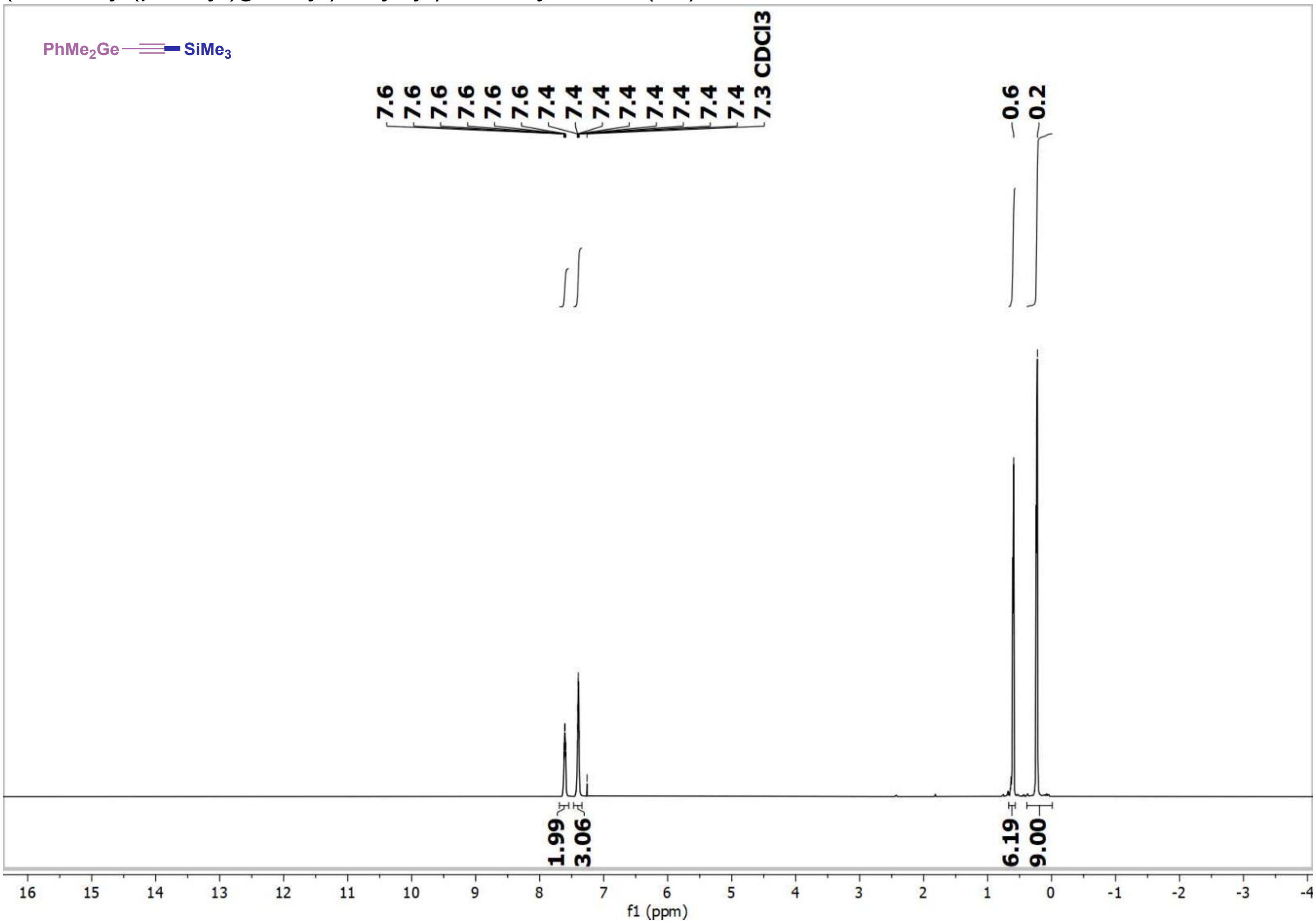


Figure S74. $^{1}H$ NMR (400 MHz, Chloroform-d, 25°C) of (dimethyl(phenyl)germyl)ethynyl)trimethylsilane (**3x**).

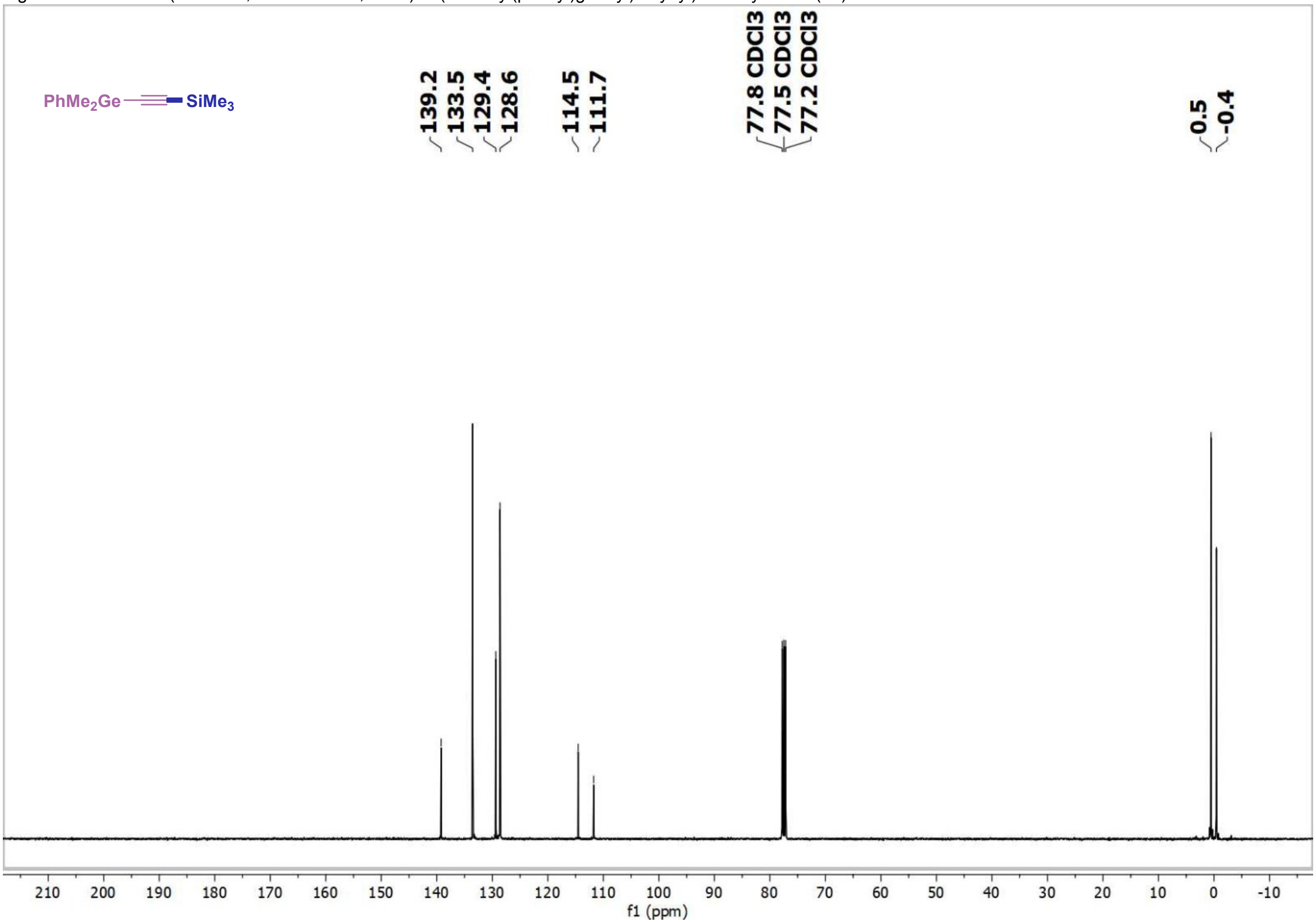


Figure S75. $^{13}C\{^{1}H\}$ NMR (101 MHz, Chloroform-d, 25°C) of (dimethyl(phenyl)germyl)ethynyl)trimethylsilane (**3x**).

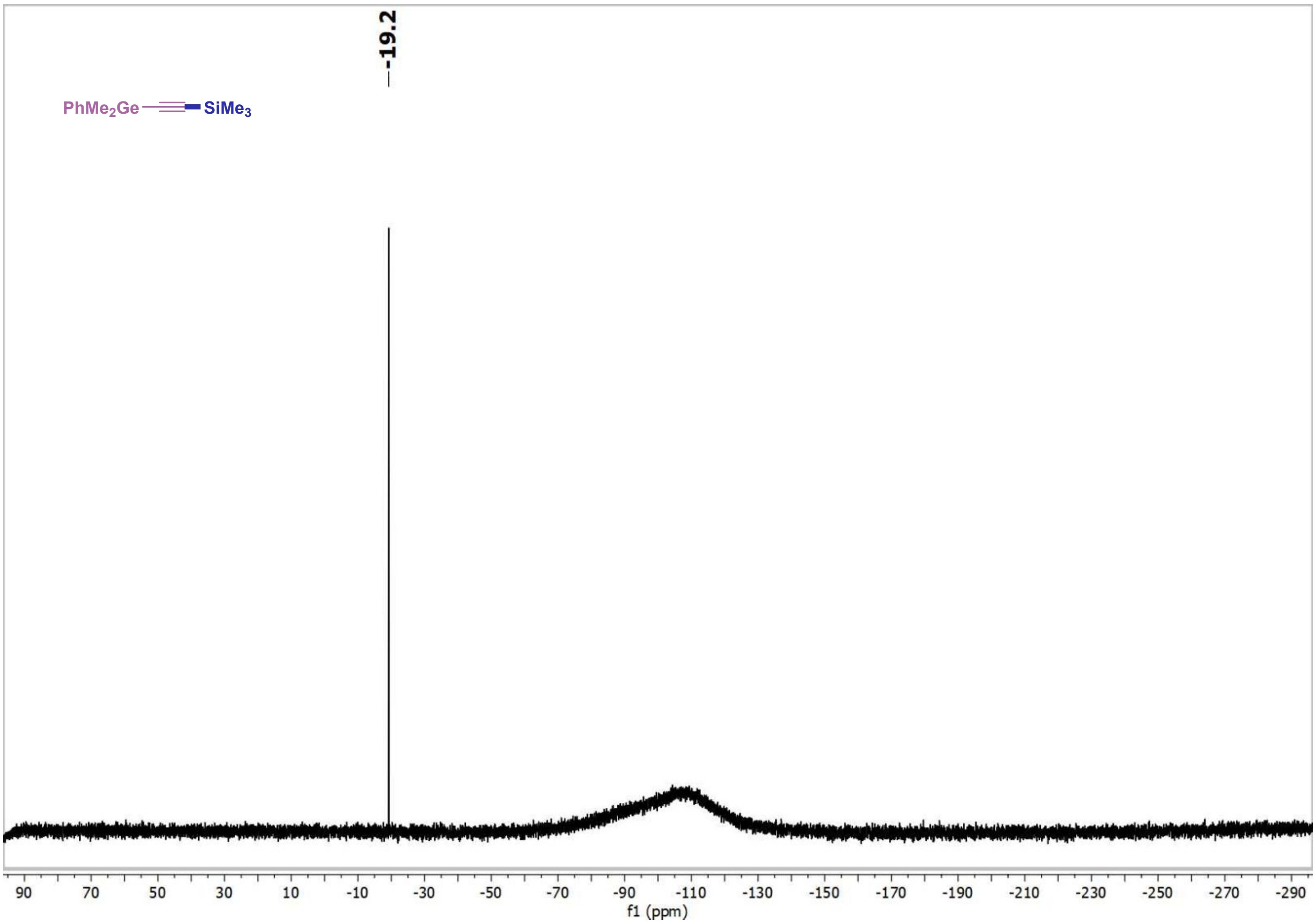


Figure S76. $^{29}$Si NMR (79 MHz, Chloroform-d, 25°C) of (dimethyl(phenyl)germyl)ethynyl)trimethylsilane (**3x**).

1-((Dimethyl((trimethylsilyl)ethynyl)silyl)oxy)-3,5,7,9,11,13,15-heptaisobutyl-2,4,6,8,10,12,14,16,17,18,19,20-dodecaoxa-1,3,5,7,9,11,13,15-octasilapentacyclo[9.5.1.13,9.15,15.17,13]icosane (**3y**)

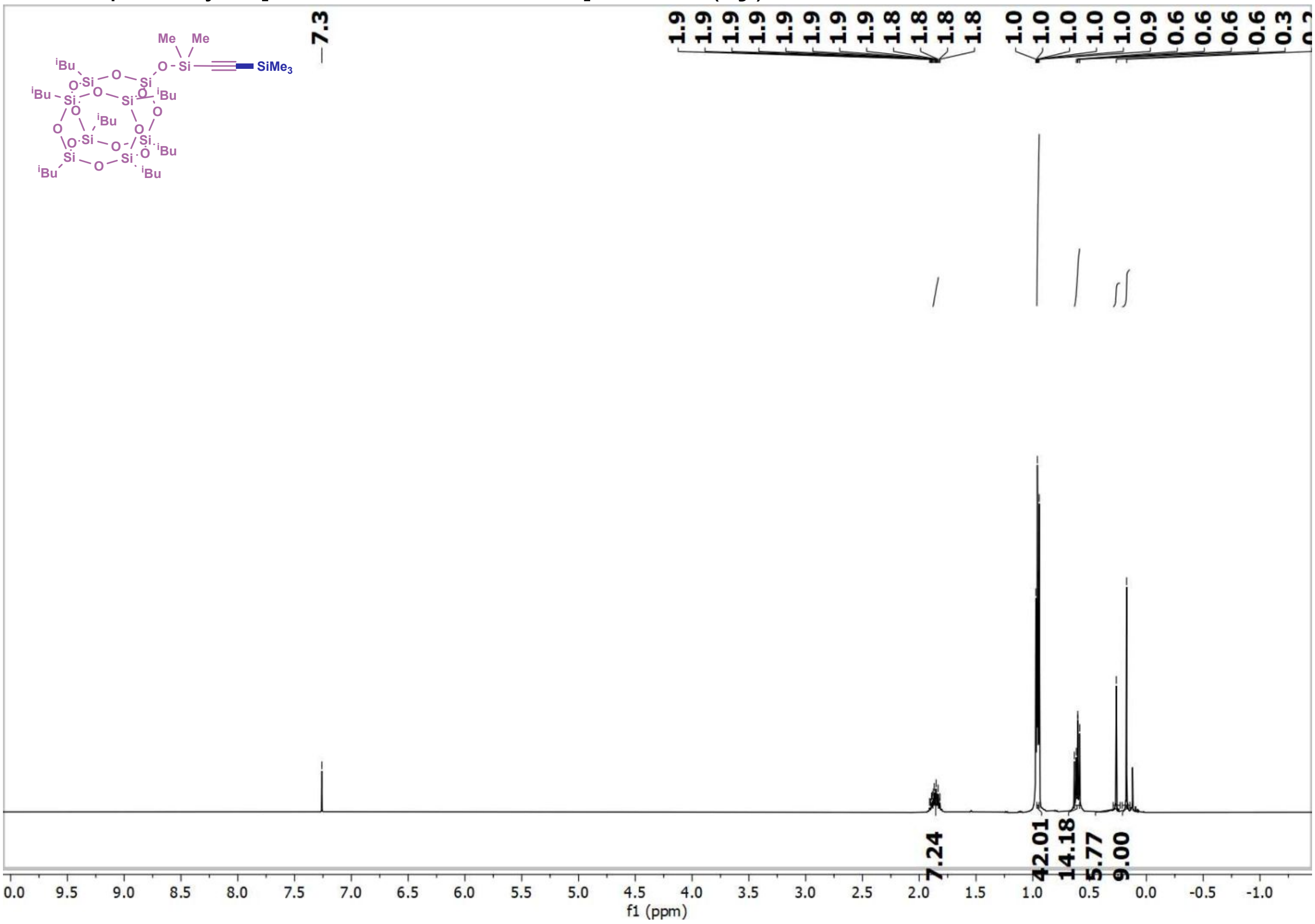


Figure S77. $^{1}H$ NMR (400 MHz, Chloroform-d, 25°C) of **3y**.

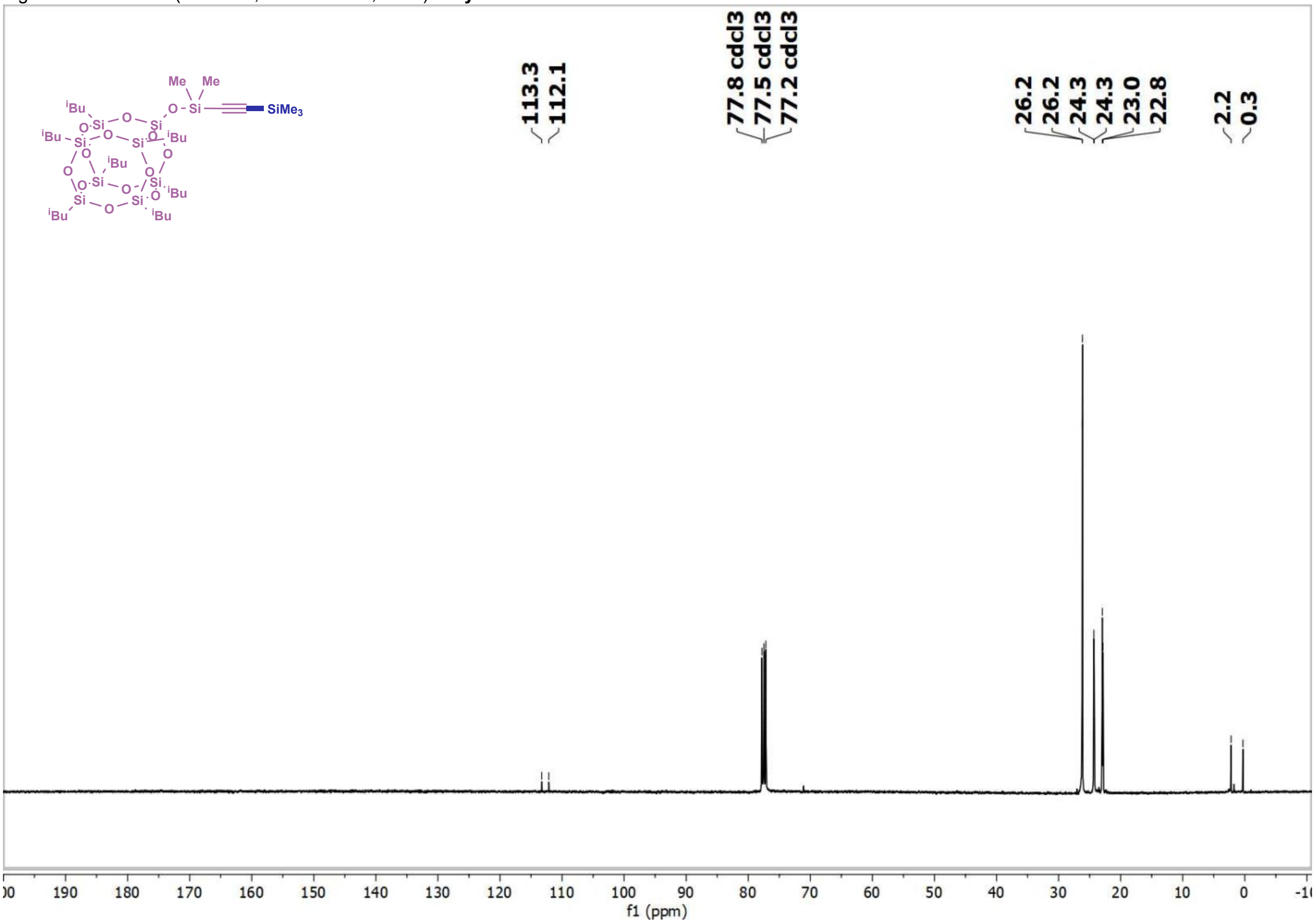


Figure S78. $^{13}C\{^{1}H\}$ NMR (101 MHz, Chloroform-d, 25°C) of **3y**.

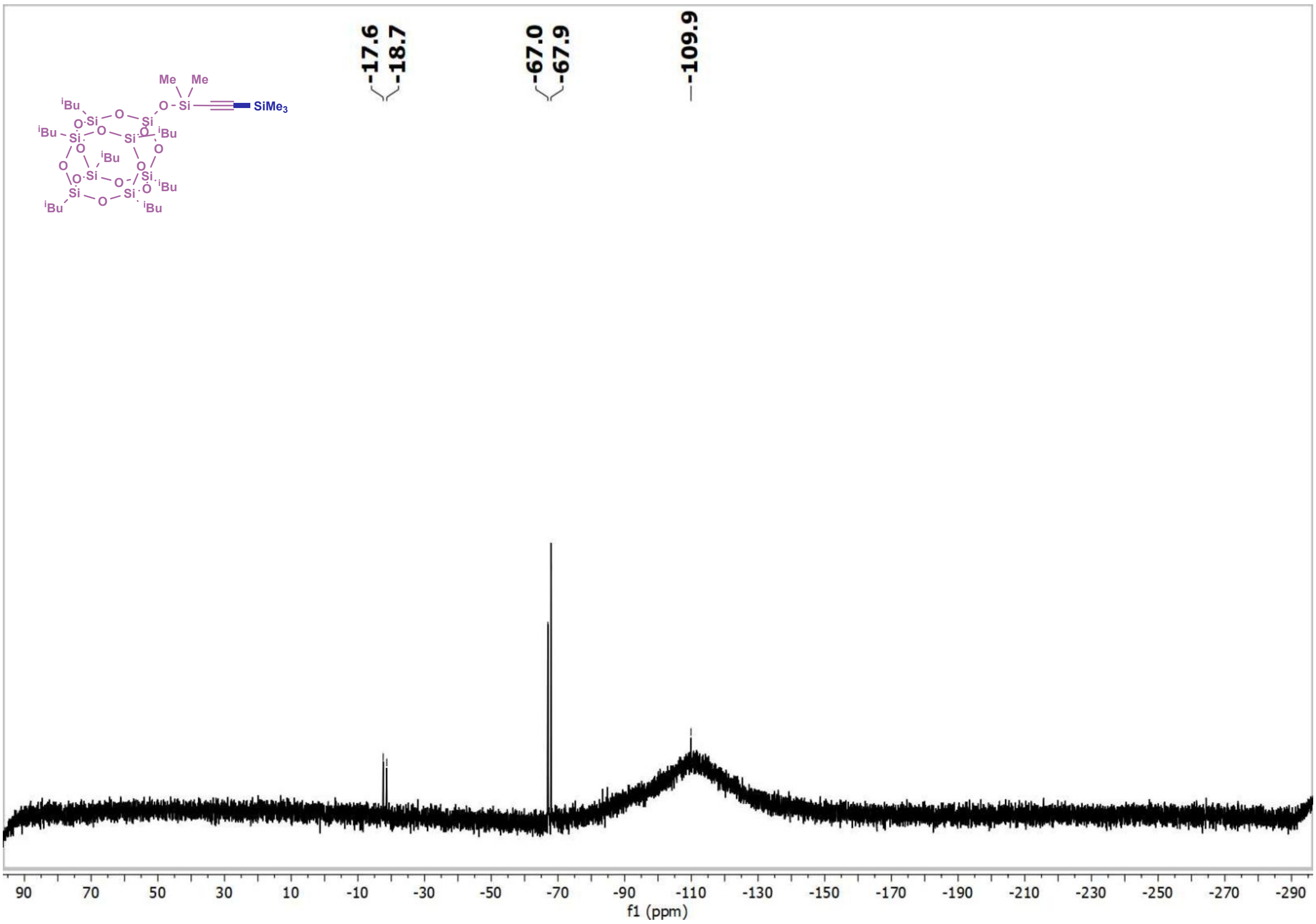


Figure S79. $^{29}Si$ NMR (79 MHz, Chloroform-d, 25°C) of **3y**.

Triethyl(phenylethynyl)silane (**3a’**)

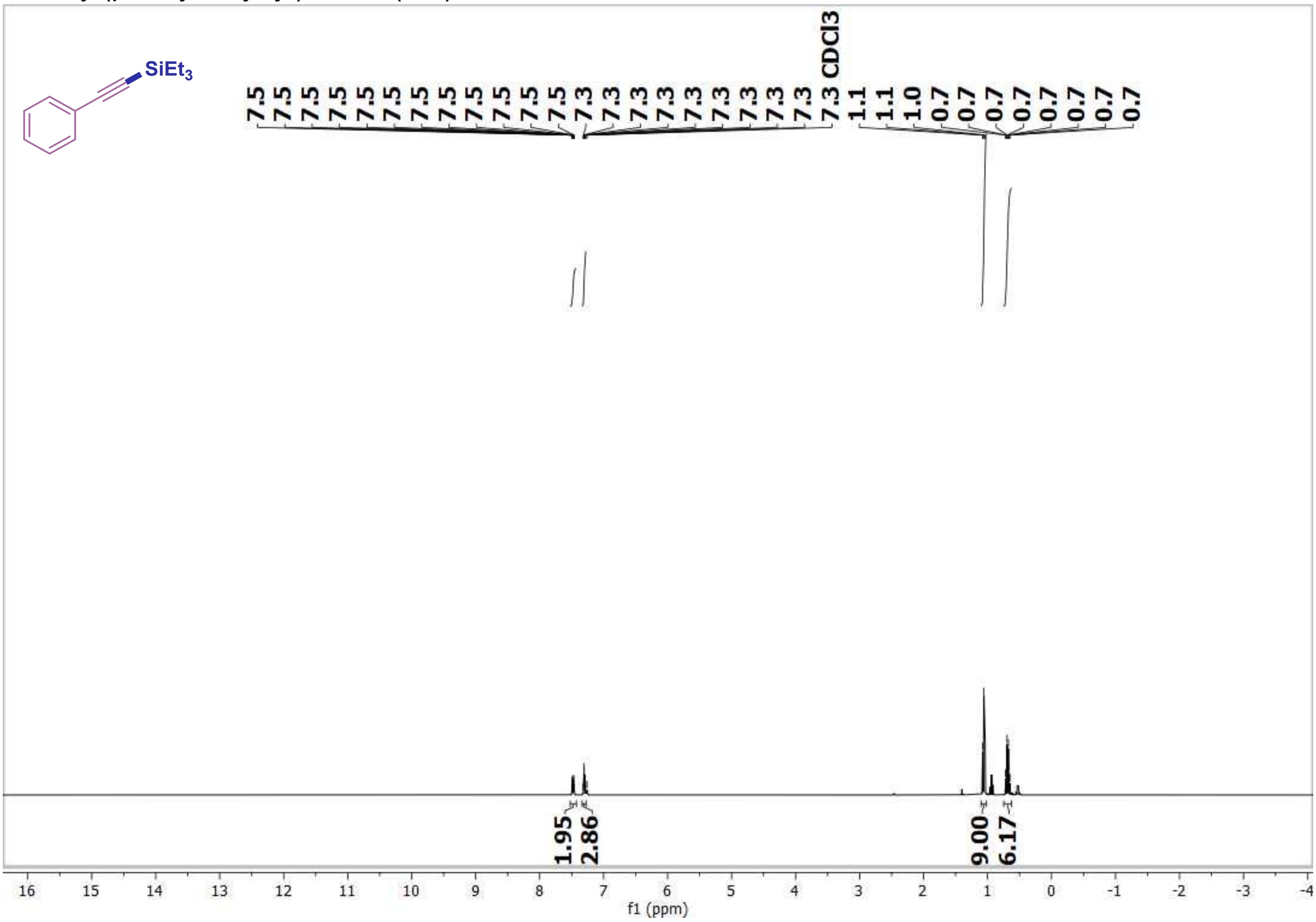


Figure S80. $^{1}H$ NMR (400 MHz, Chloroform-d, 25°C) of triethyl(phenylethynyl)silane (**3a’**).

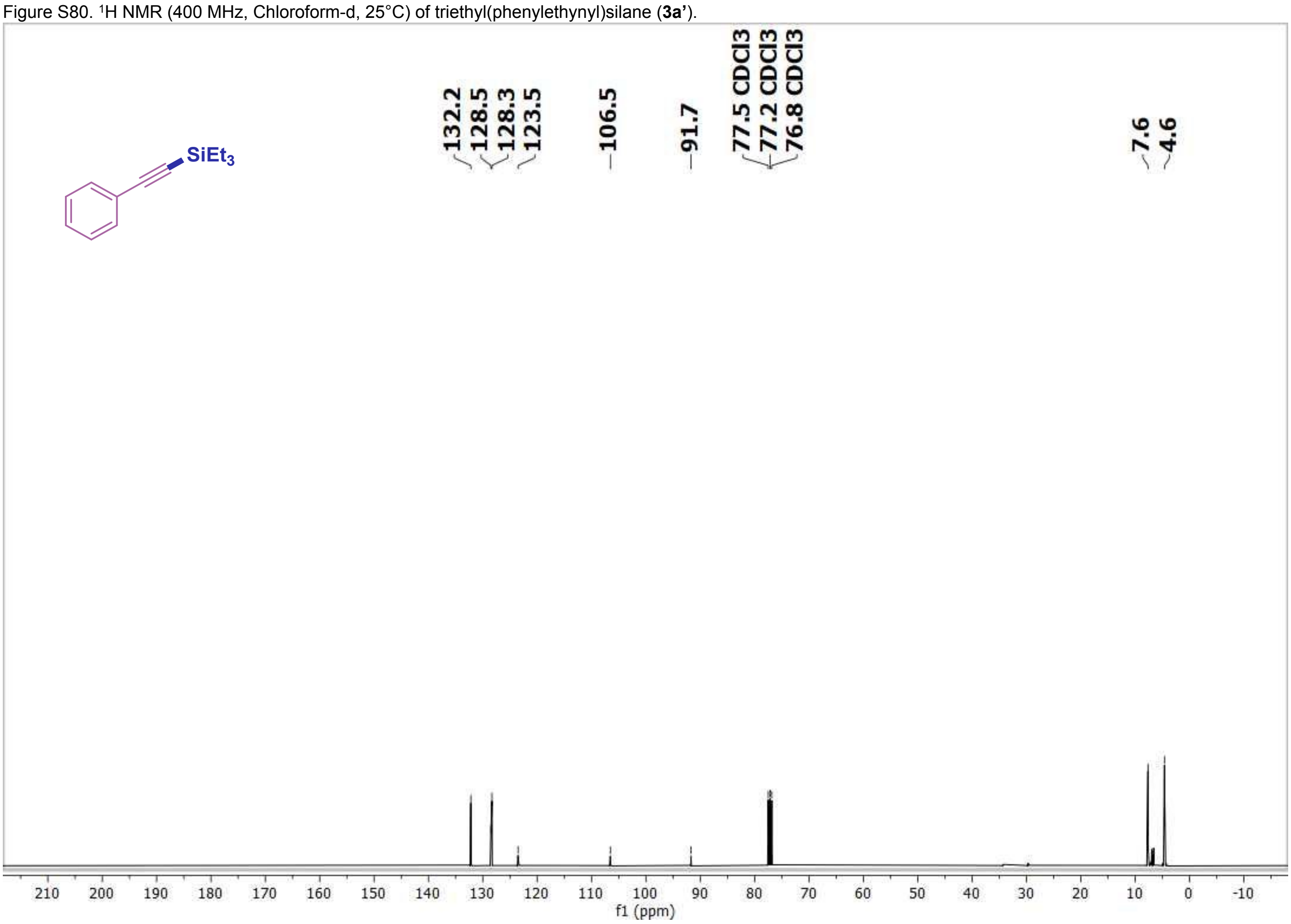


Figure S81. $^{13}C\{^{1}H\}$ NMR (101 MHz, Chloroform-d, 25°C) of triethyl(phenylethynyl)silane (**3a’**).

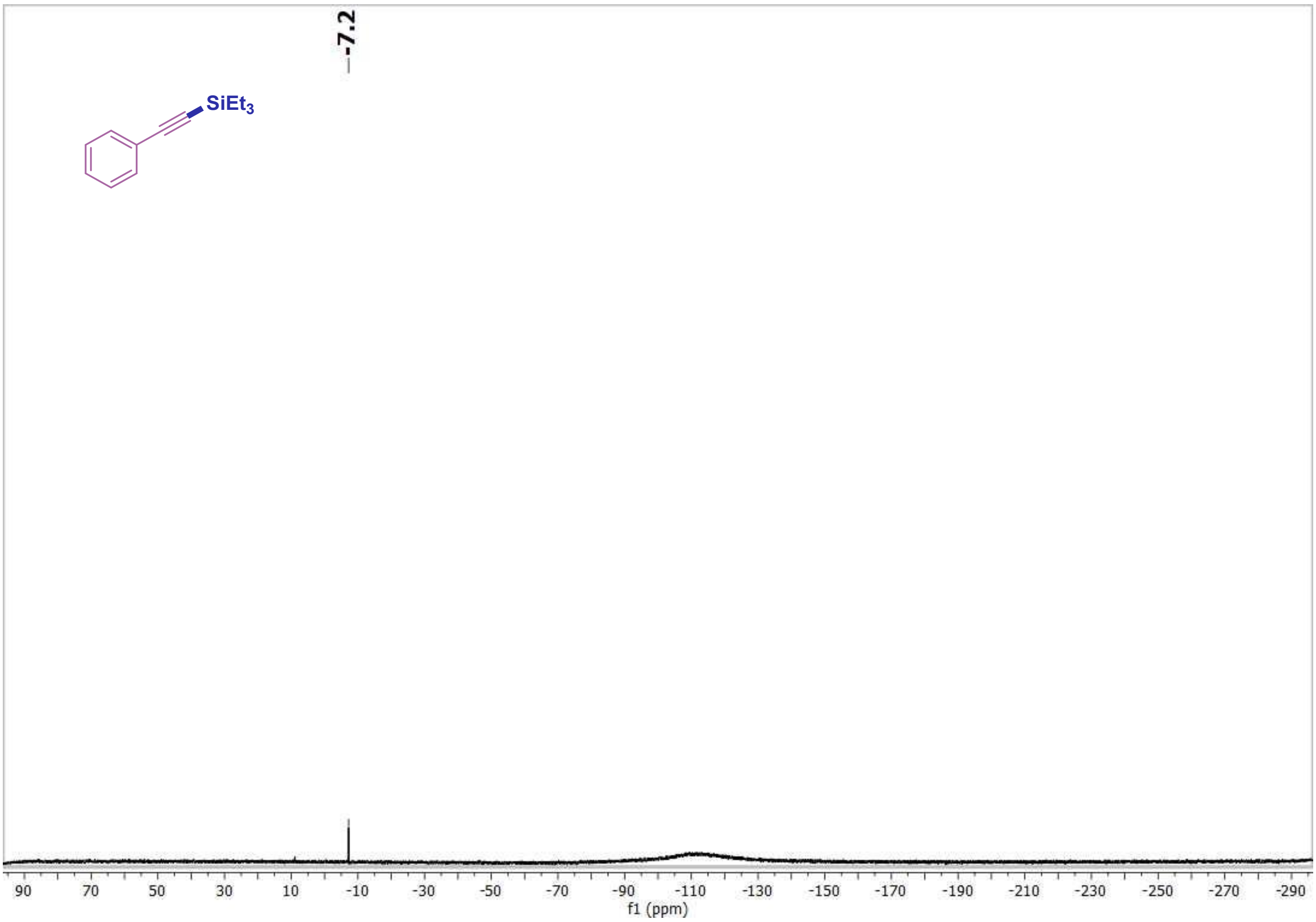


Figure S82. $^{29}Si$ NMR (79 MHz, Chloroform-d, 25°C) of triethyl(phenylethynyl)silane (**3a’**).

Dec-1-yn-1-yltrimethylsilane (**3aa**)

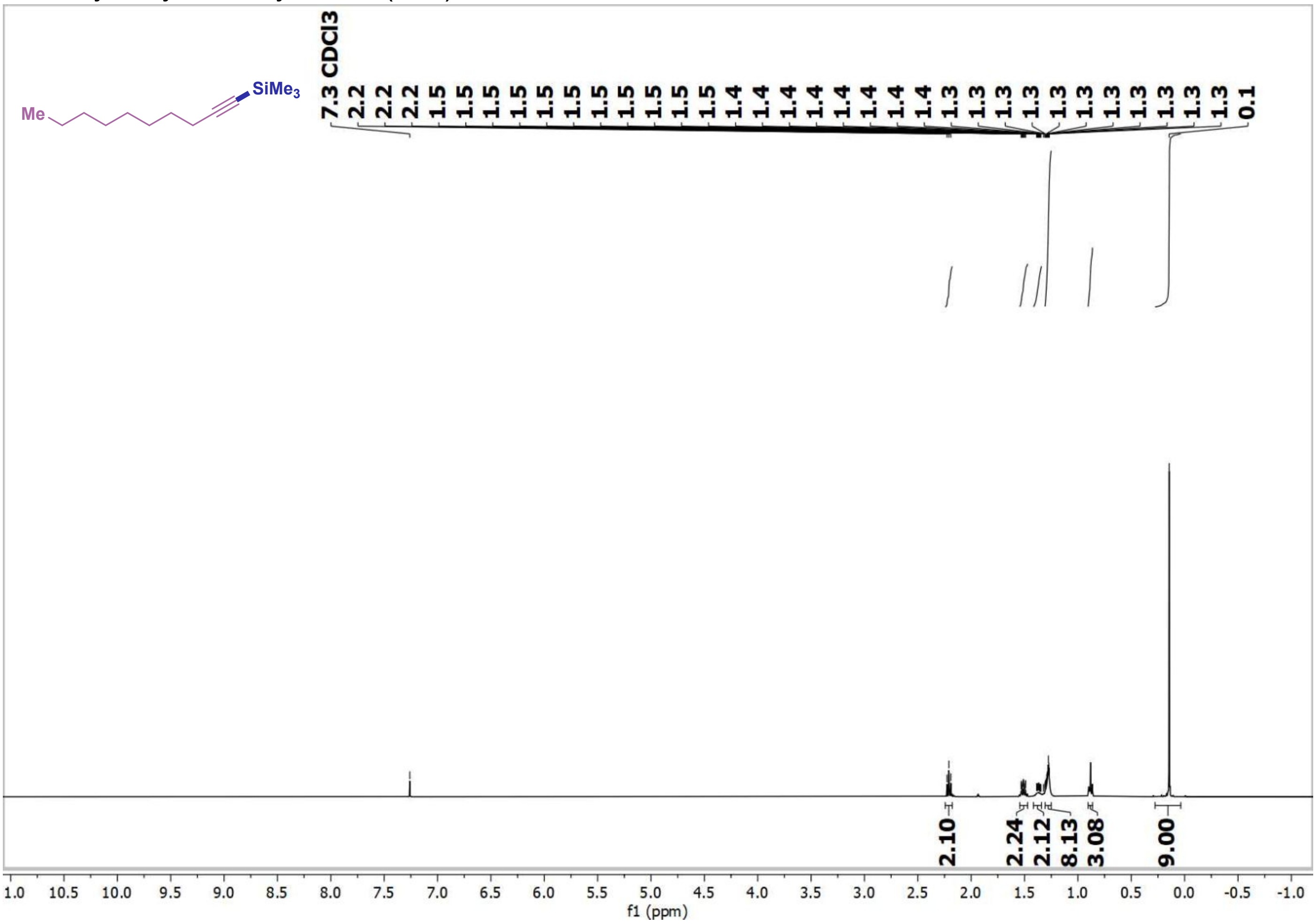


Figure S83. $^{1}H$ NMR (400 MHz, Chloroform-d, 25°C) of dec-1-yn-1-yltrimethylsilane (**3aa**).

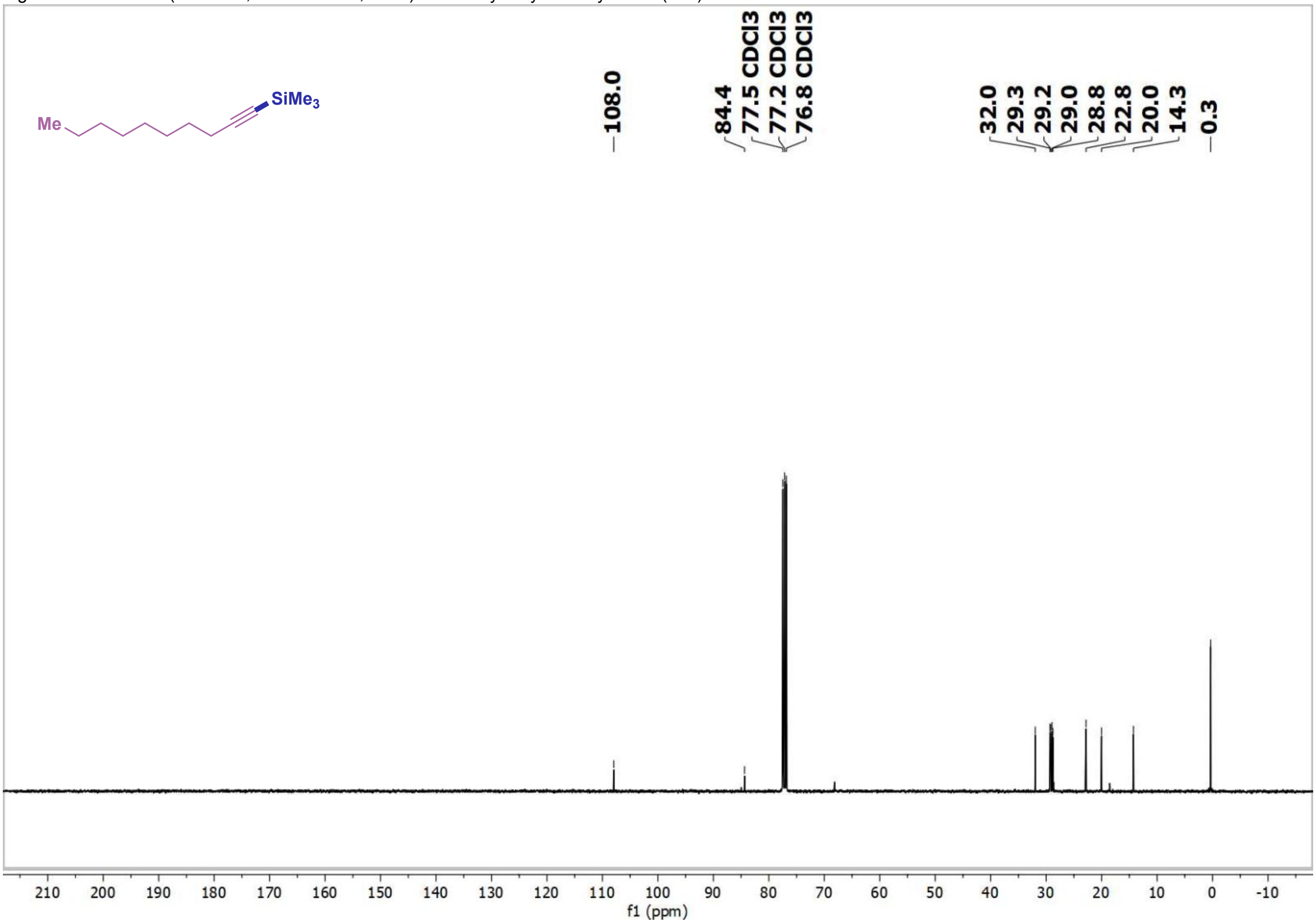


Figure S84. $^{13}C\{^{1}H\}$ NMR (101 MHz, Chloroform-d, 25°C) of dec-1-yn-1-yltrimethylsilane (**3aa**).

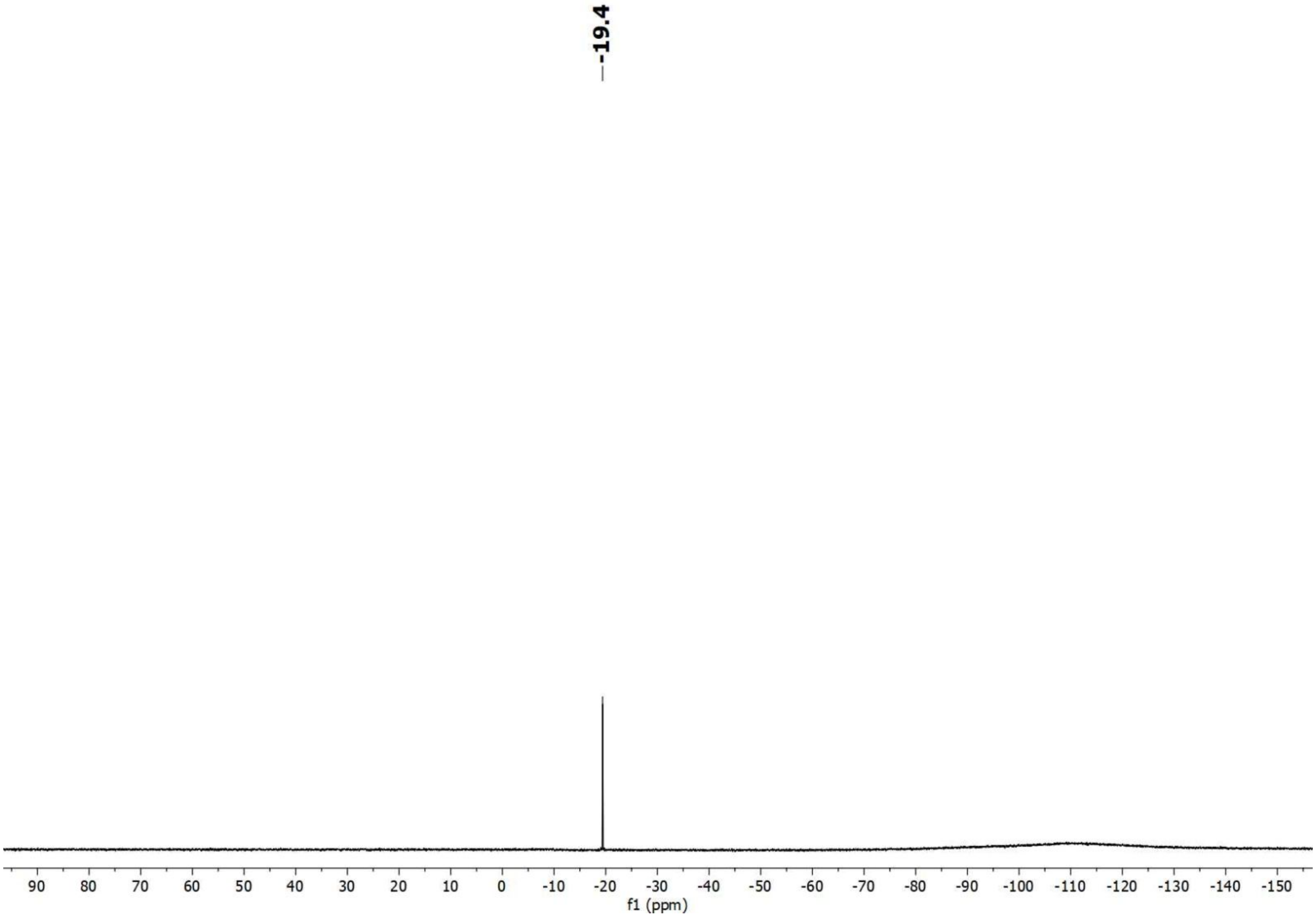


Figure S85. $^{29}Si$ NMR (79 MHz, Chloroform-d, 25°C) of dec-1-yn-1-yltrimethylsilane (**3aa**).

Trimethyl(3-phenylprop-1-yn-1-yl)silane (**3ab**)

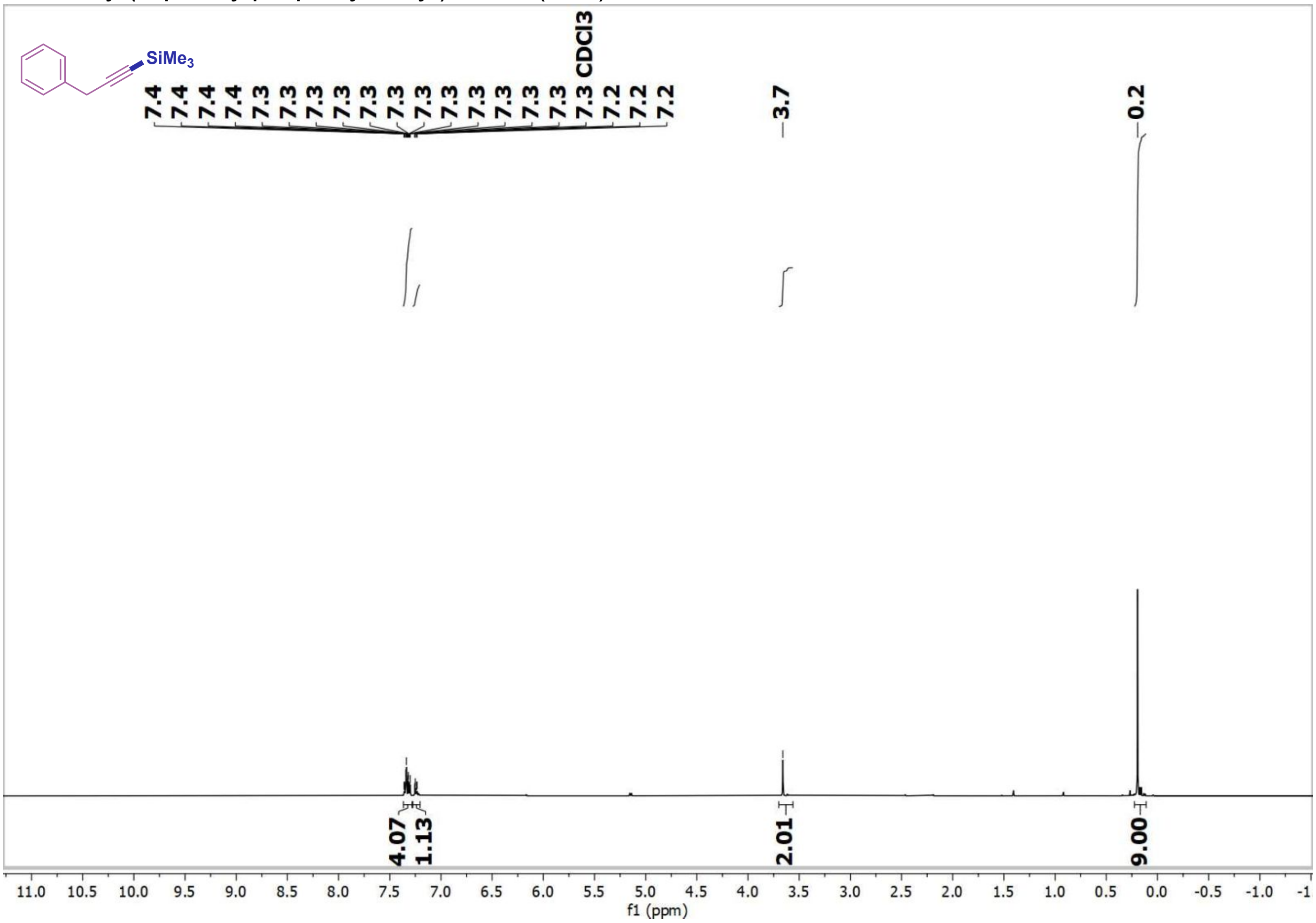


Figure S86. $^{1}H$ NMR (400 MHz, Chloroform-d, 25°C) of trimethyl(3-phenylprop-1-yn-1-yl)silane (**3ab**).

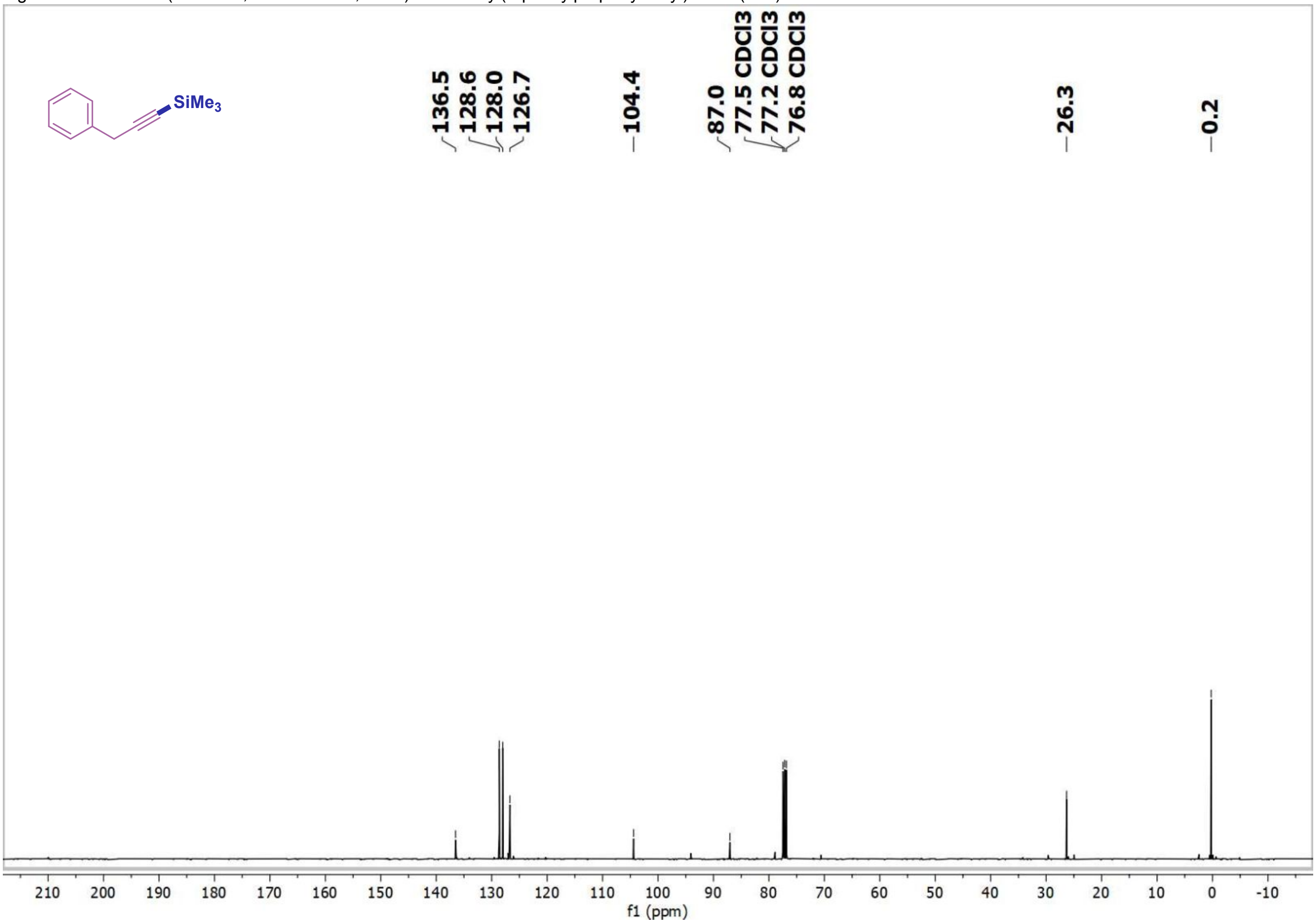


Figure S87. $^{13}C\{^{1}H\}$ NMR (101 MHz, Chloroform-d, 25°C) of trimethyl(3-phenylprop-1-yn-1-yl)silane (**3ab**).

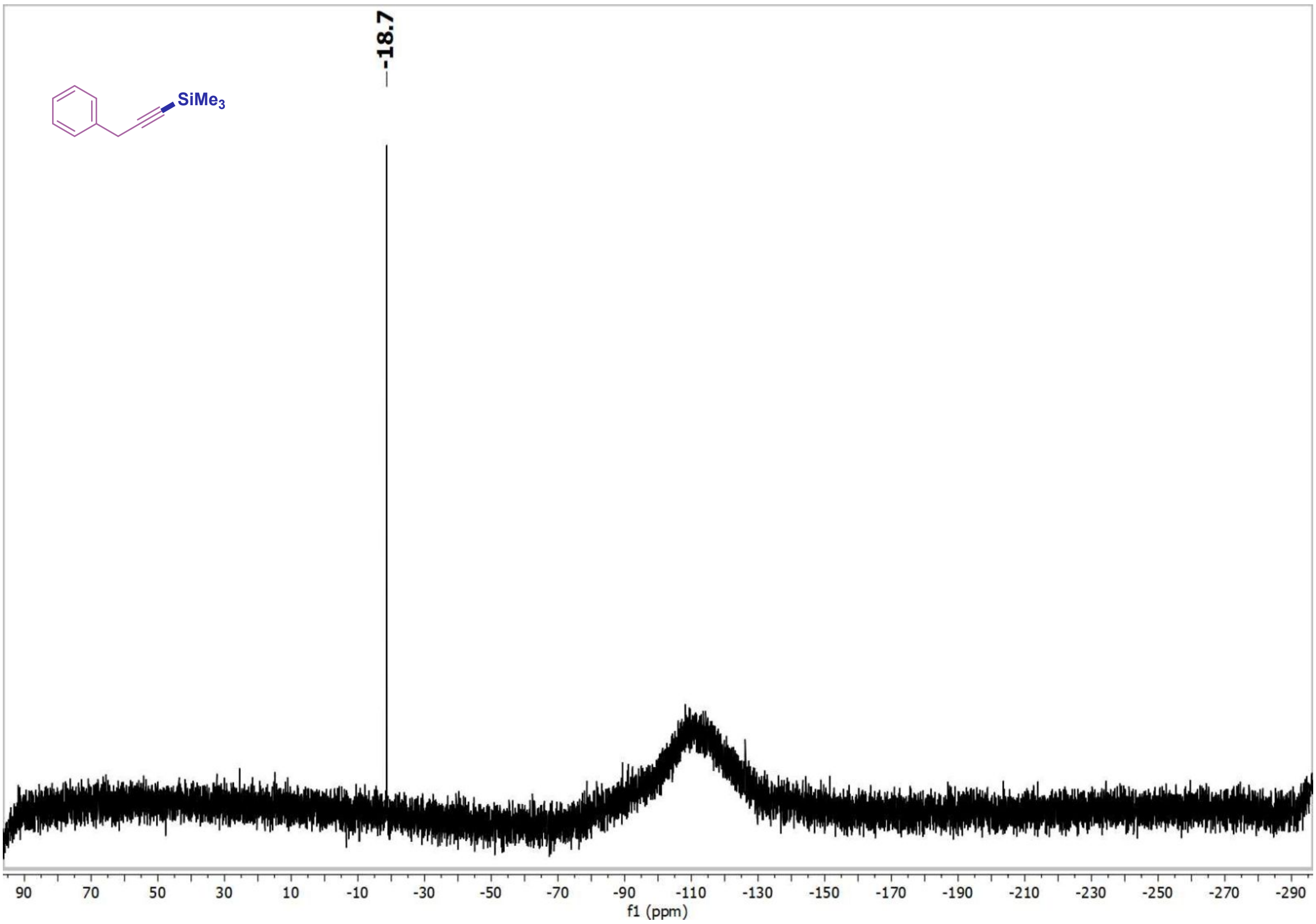


Figure S88. $^{29}$Si NMR (79 MHz, Chloroform-d, 25°C) of trimethyl(3-phenylprop-1-yn-1-yl)silane (**3ab**).

# *Tert*-butyldimethyl((3-(trimethylsilyl)prop-2-yn-1-yl)oxy)silane (**3ac**)

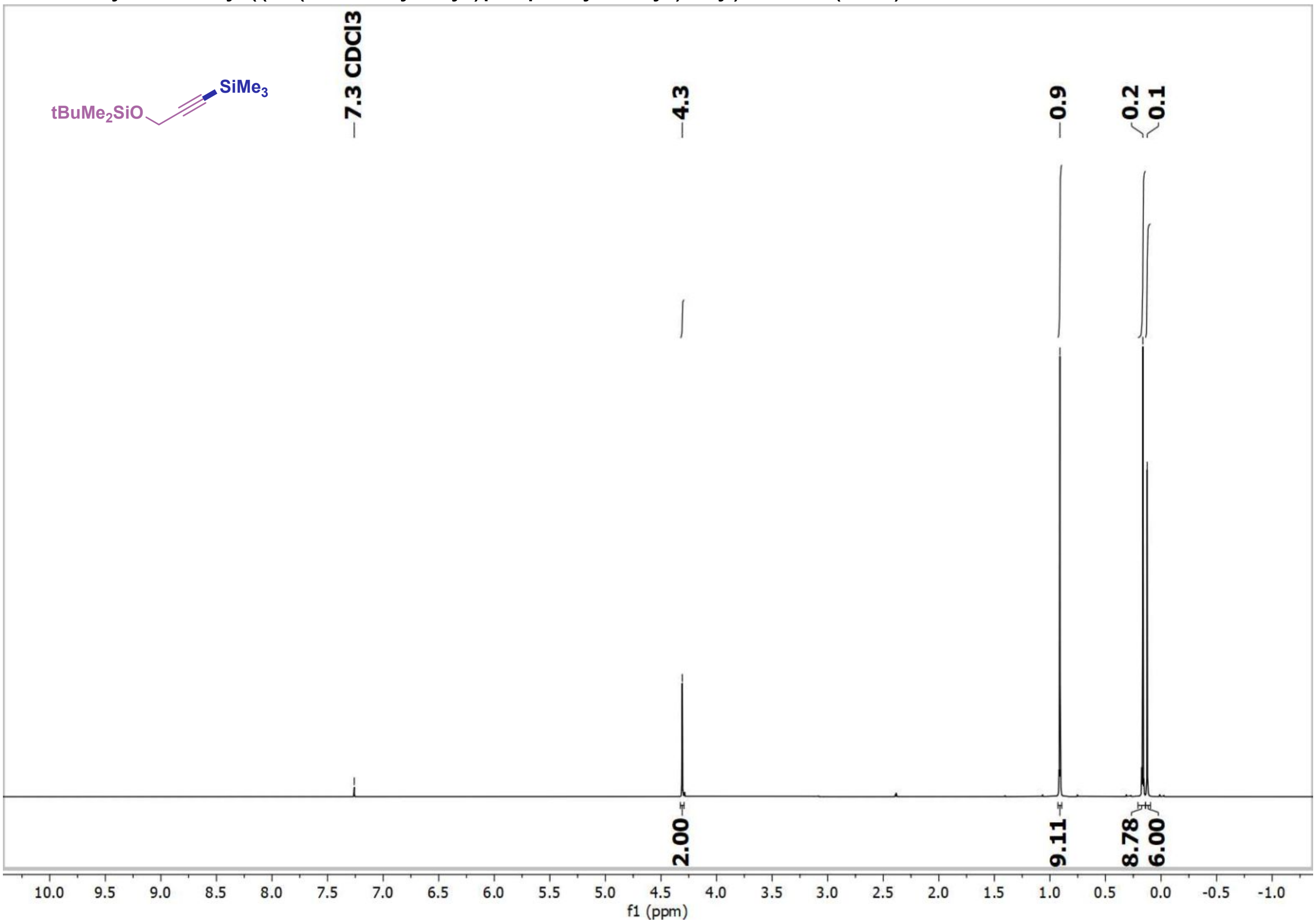


Figure S89. $^{1}H$ NMR (400 MHz, Chloroform-d, 25°C) of *tert*-butyldimethyl((3-(trimethylsilyl)prop-2-yn-1-yl)oxy)silane (**3ac**).

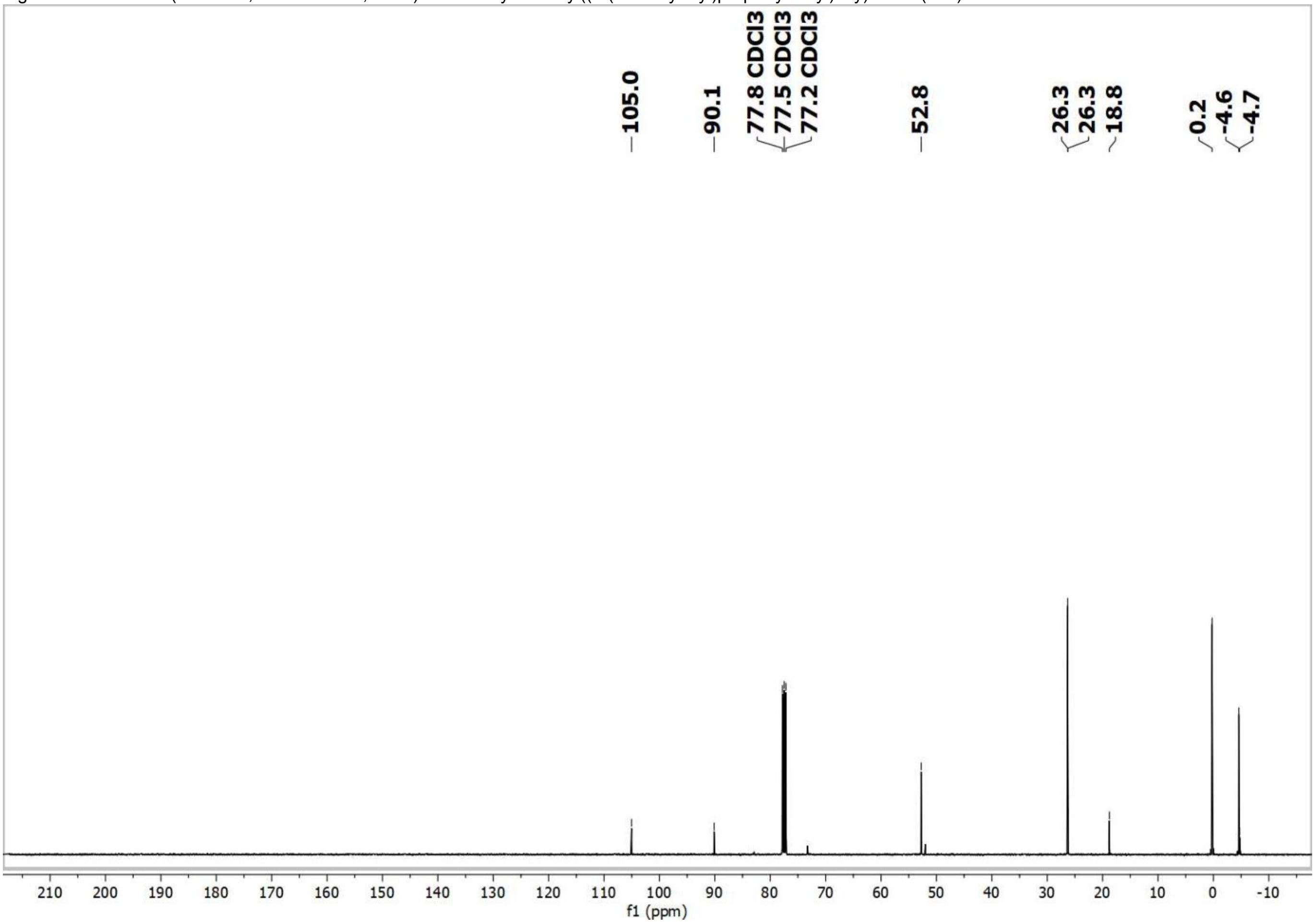


Figure S90. $^{13}C\{^{1}H\}$ NMR (101 MHz, Chloroform-d, 25°C) of *tert*-butyldimethyl((3-(trimethylsilyl)prop-2-yn-1-yl)oxy)silane (**3ac**).

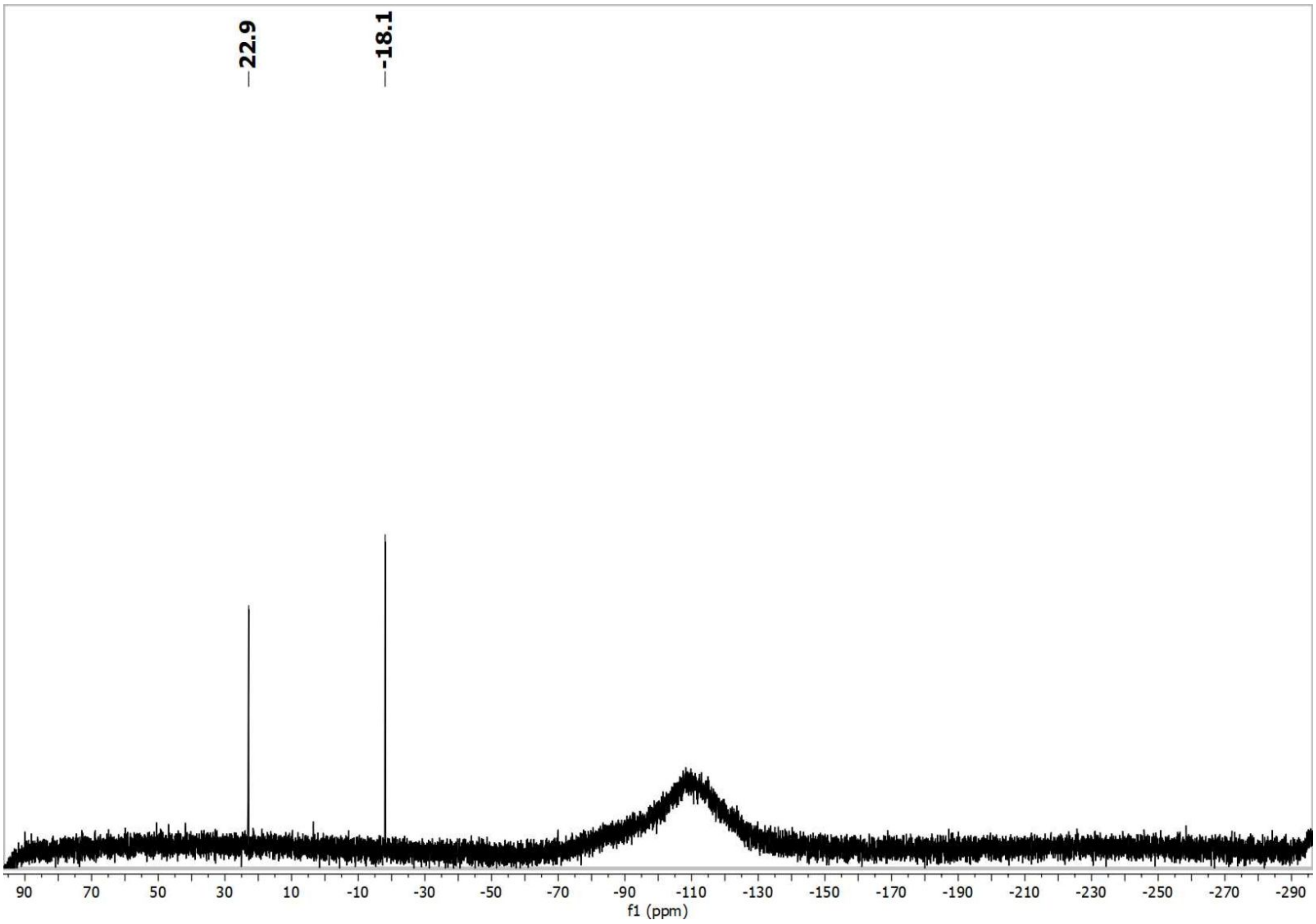


Figure S91. $^{29}Si$ NMR (79 MHz, Chloroform-d, 25°C) of *tert*-butyldimethyl((3-(trimethylsilyl)prop-2-yn-1-yl)oxy)silane (**3ac**).

## *N,N*-diethyl-3-(trimethylsilyl)prop-2-yn-1-amine (**3ad**)

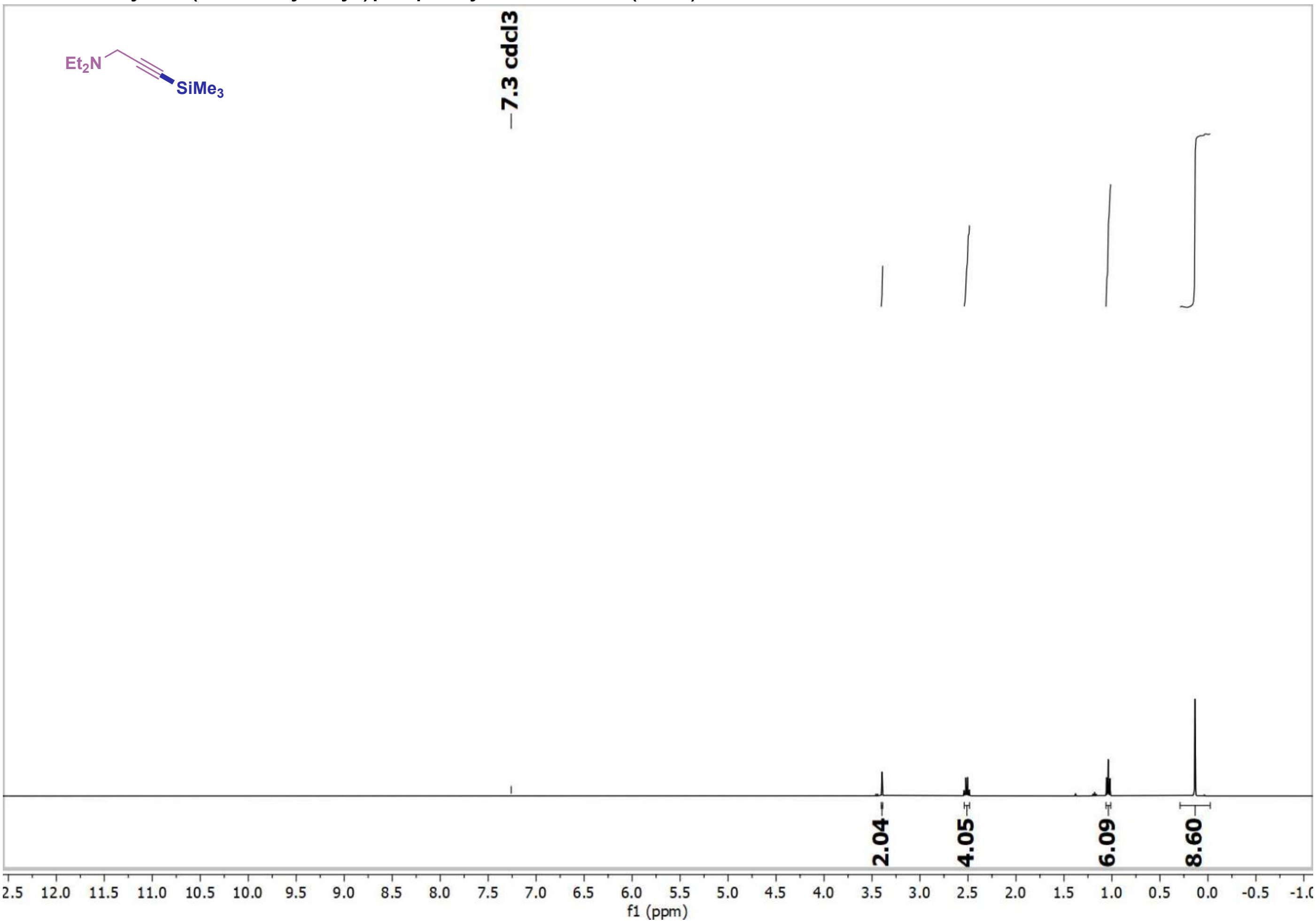


Figure S92. $^{1}$H NMR (400 MHz, Chloroform-d, 25°C) of *N,N*-diethyl-3-(trimethylsilyl)prop-2-yn-1-amine (**3ad**).

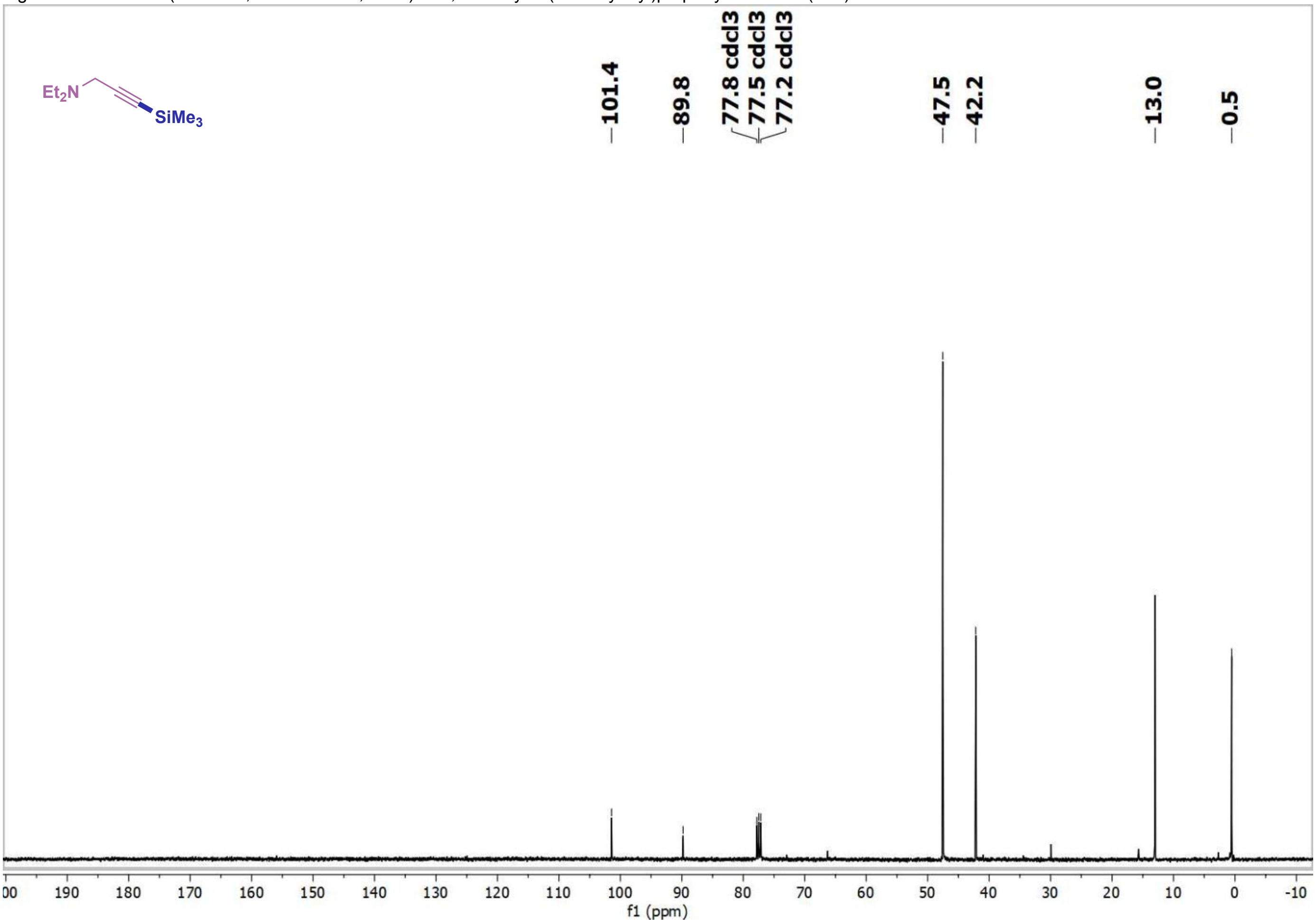


Figure S93. $^{13}$C{$^{1}$H} NMR (101 MHz, Chloroform-d, 25°C) of *N,N*-diethyl-3-(trimethylsilyl)prop-2-yn-1-amine (**3ad**).

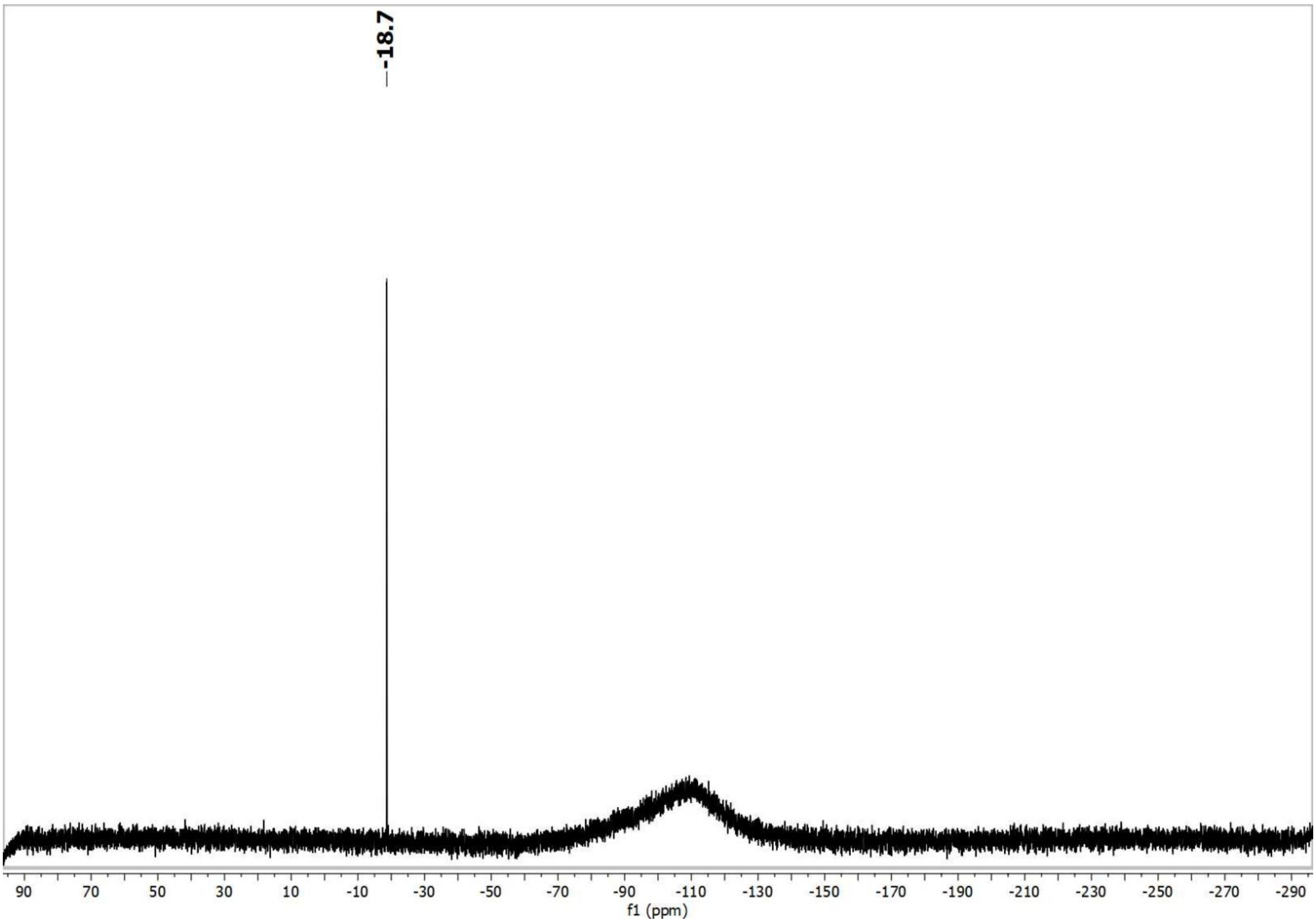


Figure S94. $^{29}Si$ NMR (79 MHz, Chloroform-d, 25°C) of *N,N*-diethyl-3-(trimethylsilyl)prop-2-yn-1-amine (**3ad**).

(Oxybis(prop-1-yne-3,1-diyl))bis(trimethylsilane) (**3ae**)

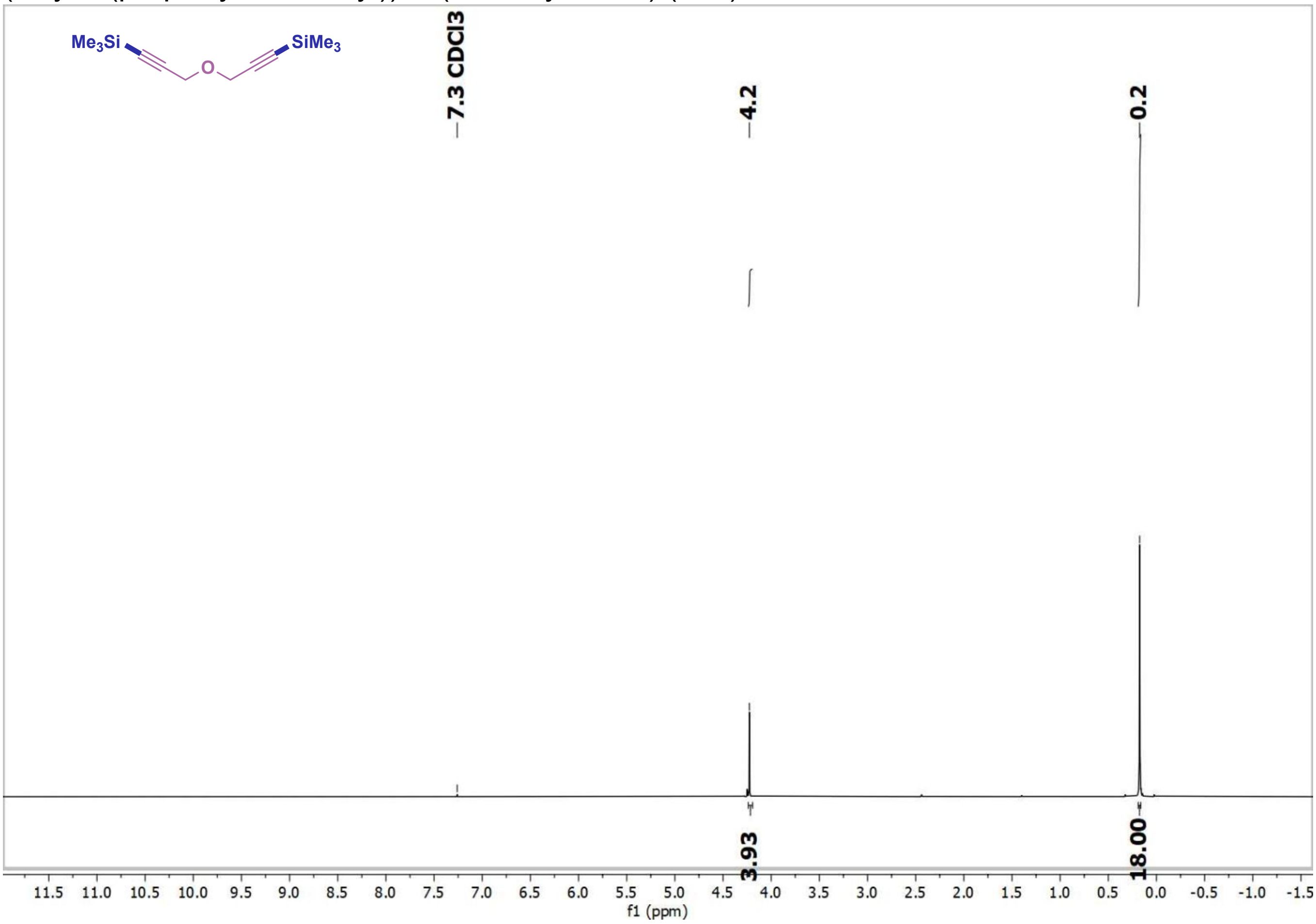


Figure S95. $^{1}H$ NMR (400 MHz, Chloroform-d, 25°C) of (oxybis(prop-1-yne-3,1-diyl))bis(trimethylsilane) (**3ae**).

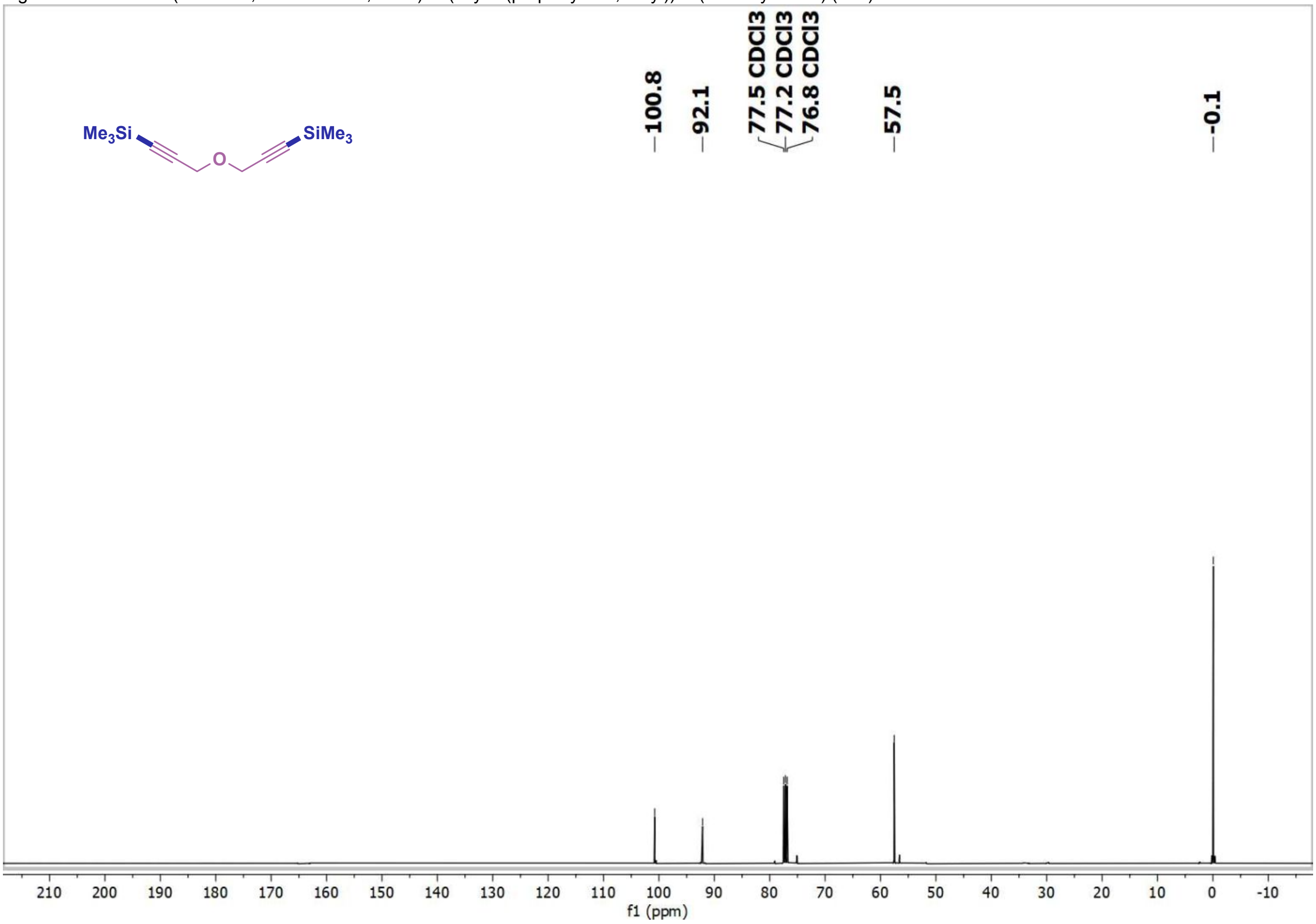


Figure S96. $^{13}C\{^{1}H\}$ NMR (101 MHz, Chloroform-d, 25°C) of (oxybis(prop-1-yne-3,1-diyl))bis(trimethylsilane) (**3ae**).

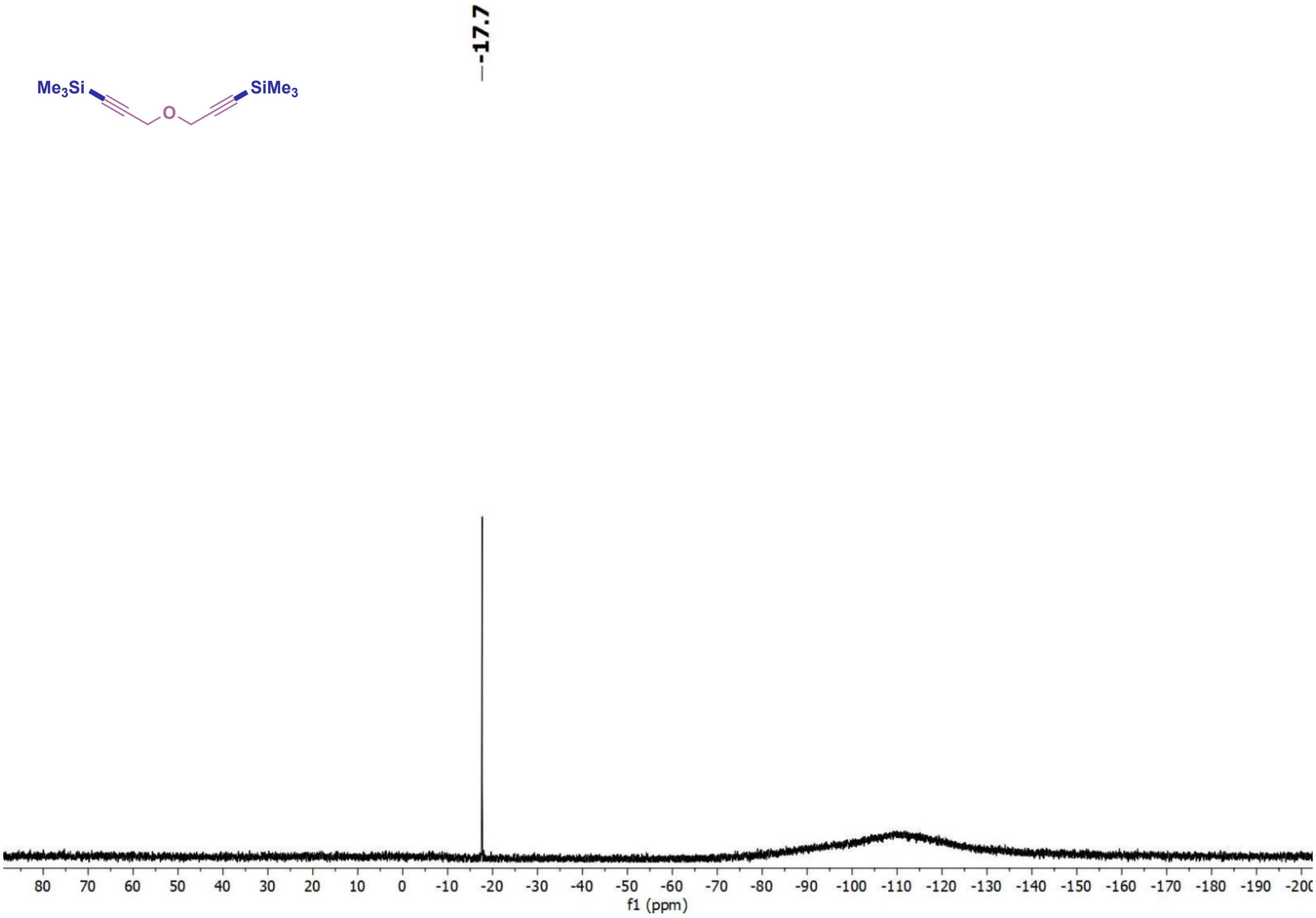


Figure S97. $^{29}Si$ NMR (79 MHz, Chloroform-d, 25°C) of (oxybis(prop-1-yne-3,1-diyl))bis(trimethylsilane) (**3ae**).

Trimethyl((4-((trimethylsilyl)ethynyl)benzyl)oxy)silane (**3af**)

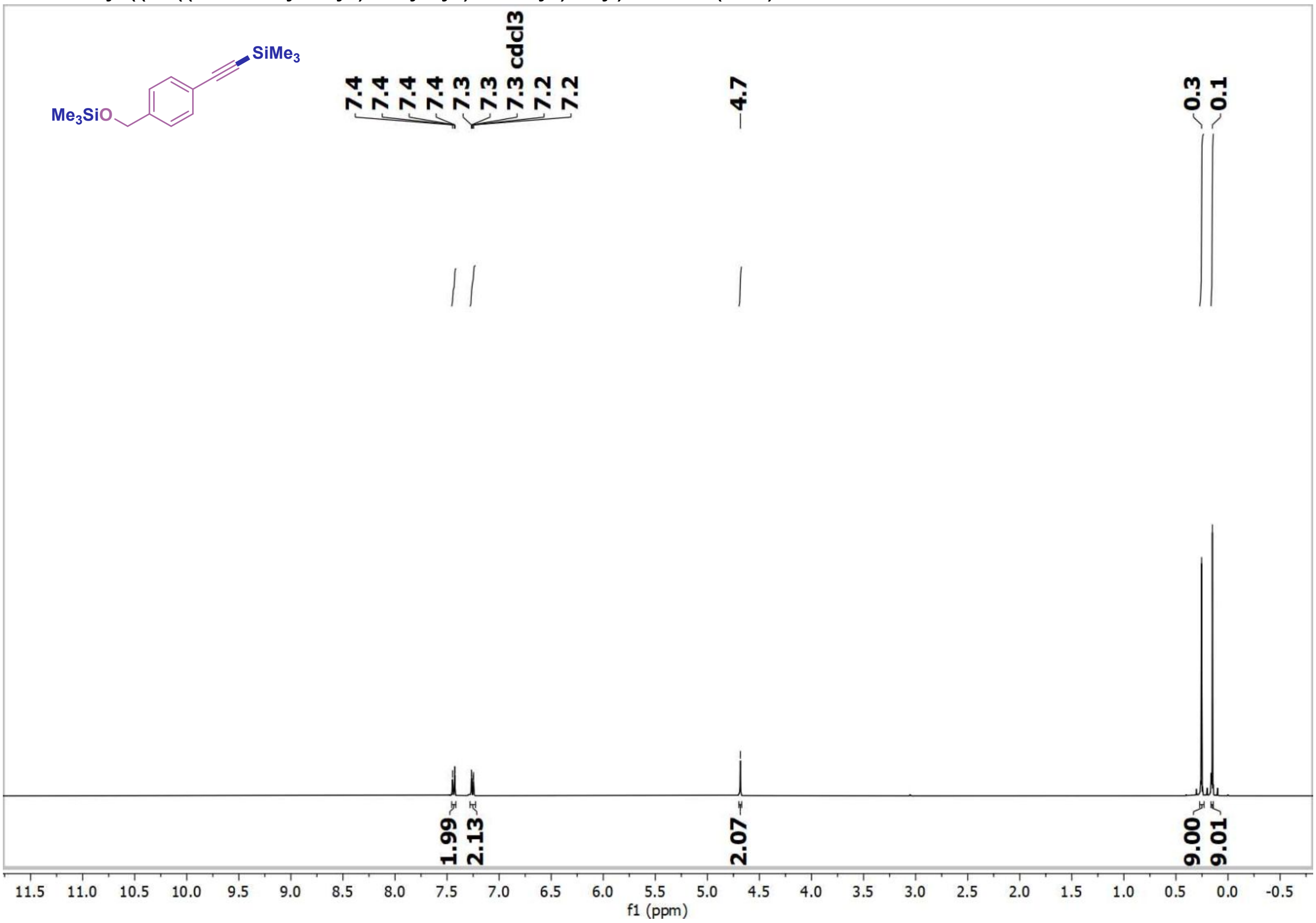


Figure S98. $^{1}$H NMR (400 MHz, Chloroform-d, 25°C) of trimethyl((4-((trimethylsilyl)ethynyl)benzyl)oxy)silane (**3af**).

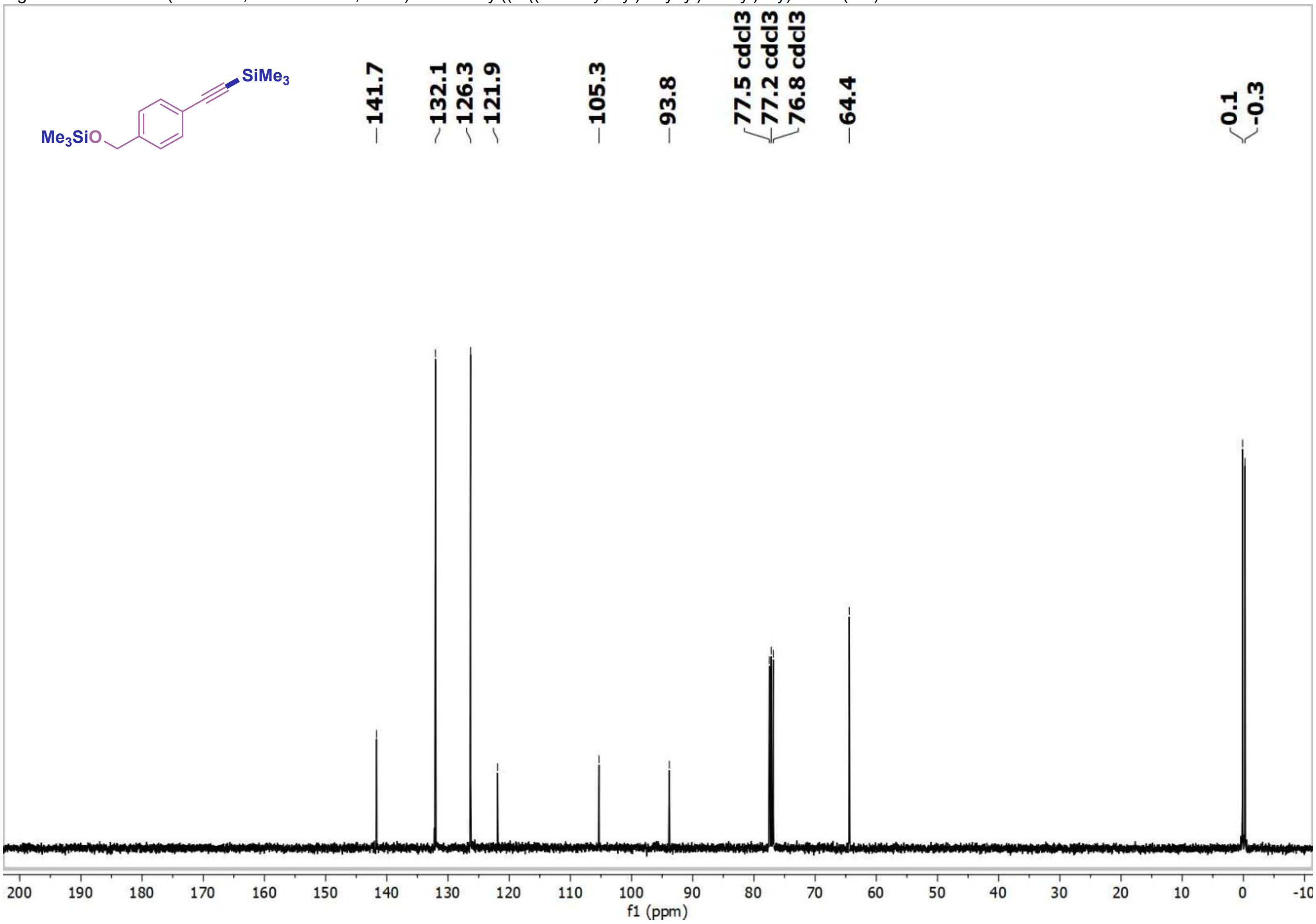


Figure S99. $^{13}$C{$^{1}$H} NMR (101 MHz, Chloroform-d, 25°C) of trimethyl((4-((trimethylsilyl)ethynyl)benzyl)oxy)silane (**3af**).

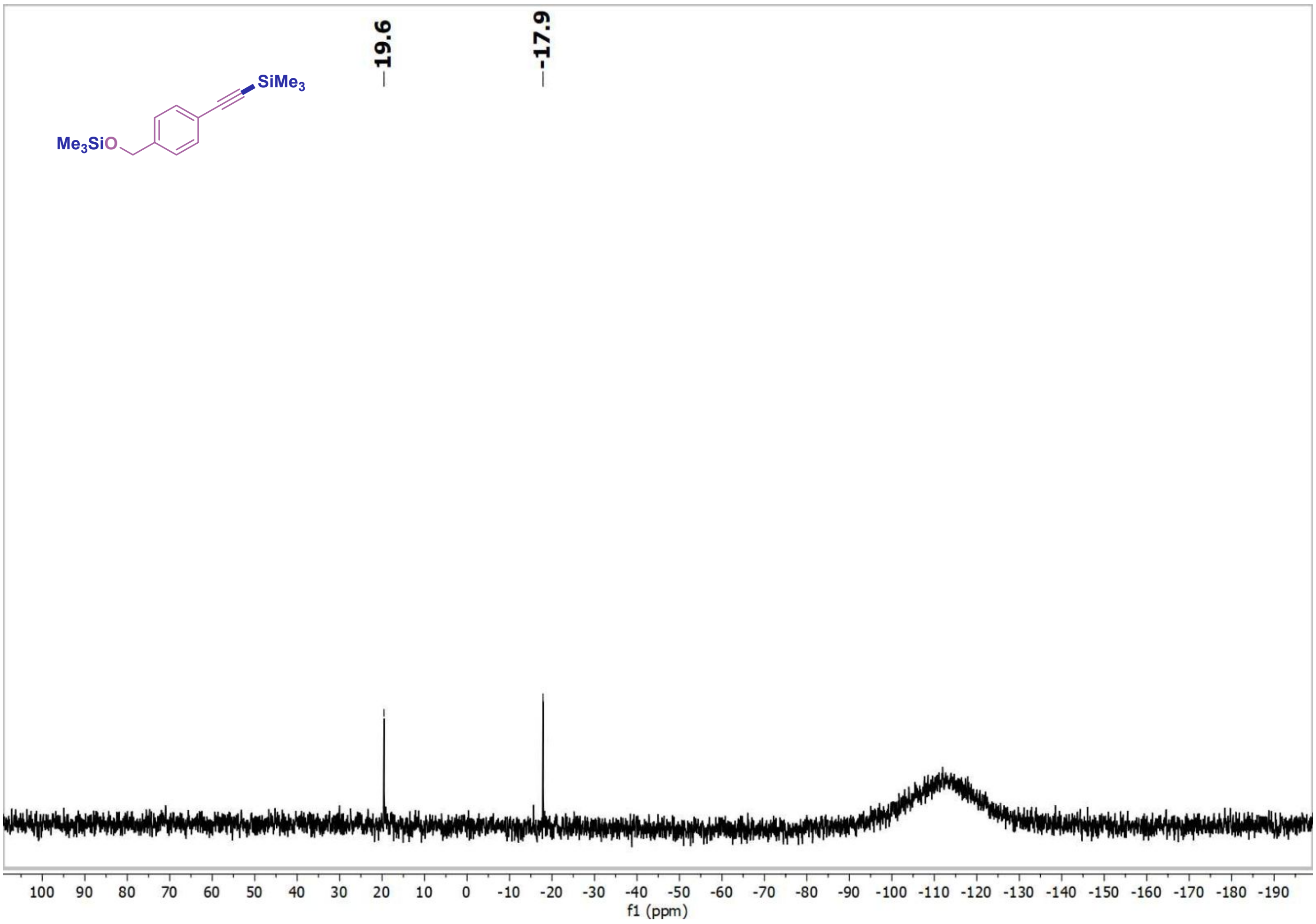


Figure S100. $^{29}$Si NMR (79 MHz, Chloroform-d, 25°C) of trimethyl((4-((trimethylsilyl)ethynyl)benzyl)oxy)silane (**3af**).

Trimethyl(3-phenyl-3-((trimethylsilyl)oxy)prop-1-yn-1-yl)silane (**3ag**)

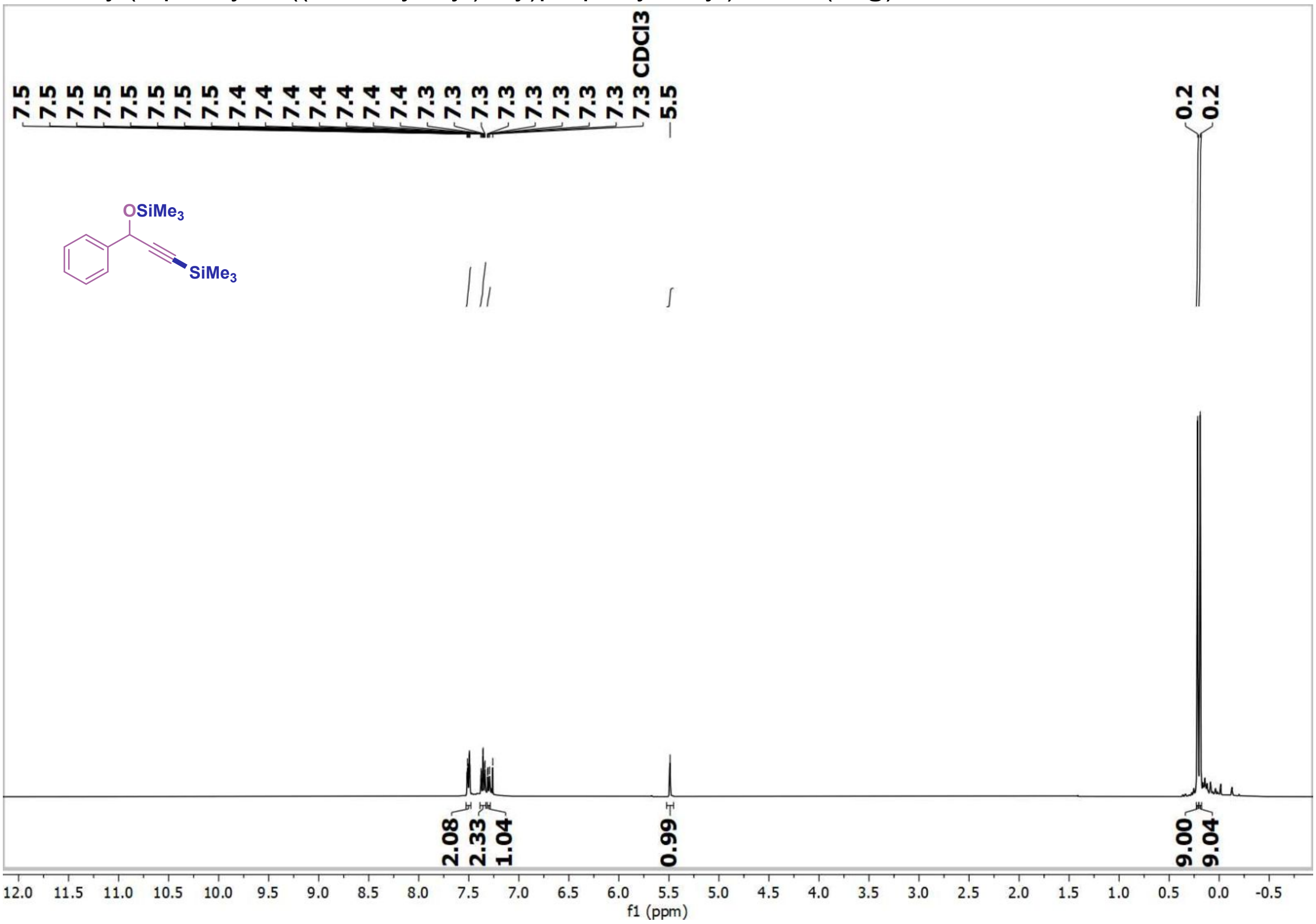


Figure S101. $^{1}H$ NMR (400 MHz, Chloroform-d, 25°C) of trimethyl(3-phenyl-3-((trimethylsilyl)oxy)prop-1-yn-1-yl)silane (**3ag**).

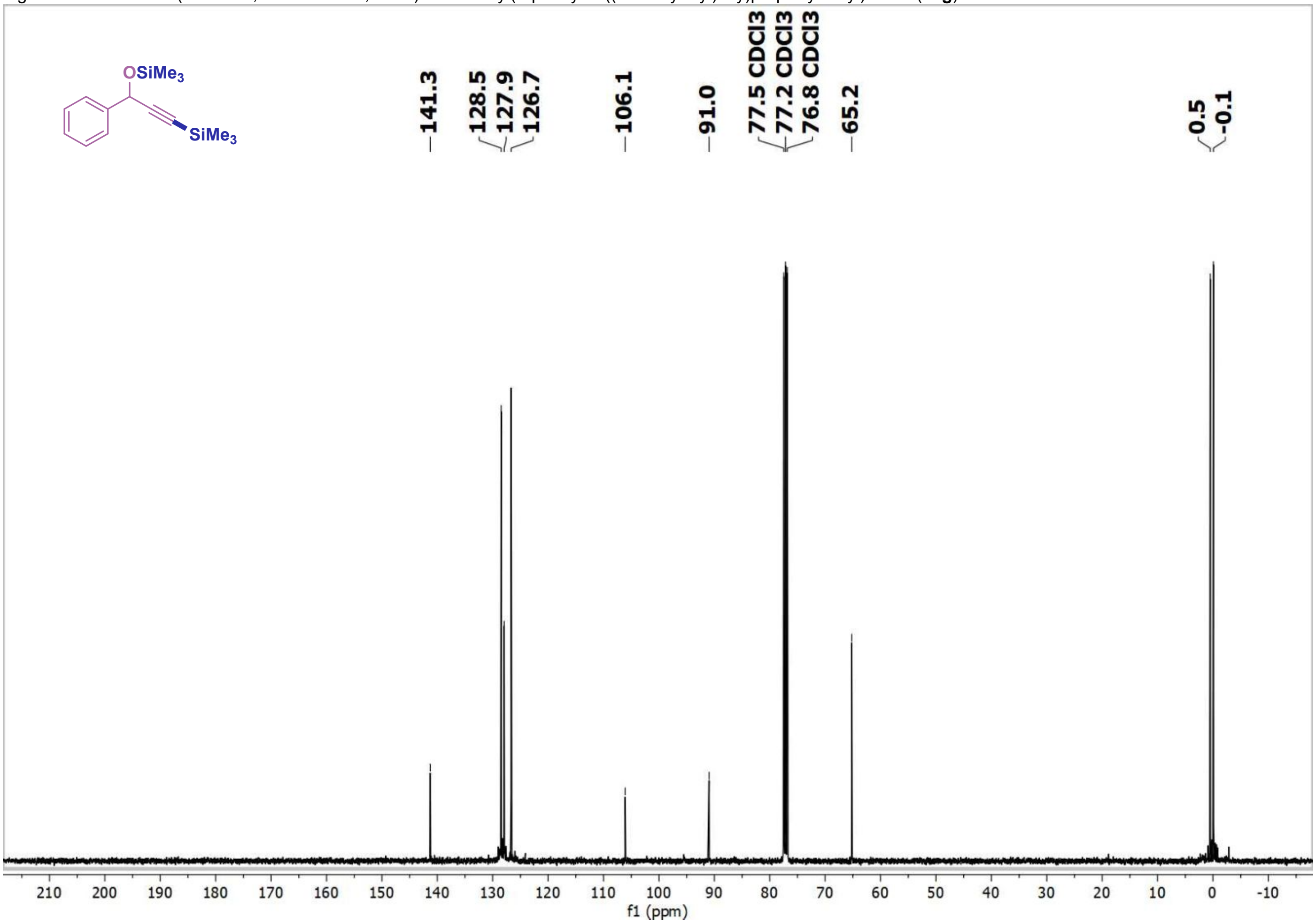


Figure S102. $^{13}C\{^{1}H\}$ NMR (101 MHz, Chloroform-d, 25°C) of trimethyl(3-phenyl-3-((trimethylsilyl)oxy)prop-1-yn-1-yl)silane (**3ag**).

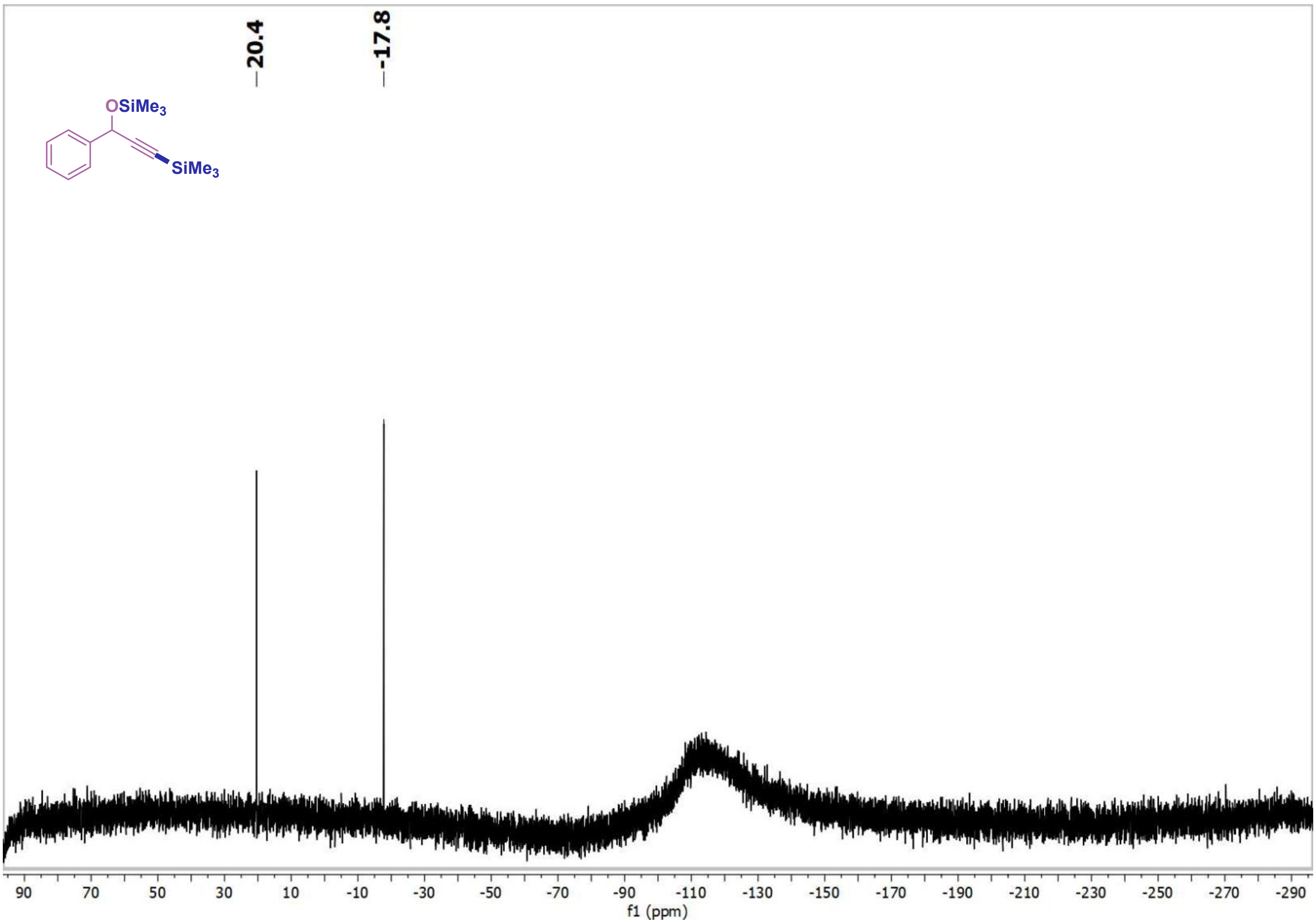


Figure S103. $^{29}Si$ NMR (79 MHz, Chloroform-d, 25°C) of trimethyl(3-phenyl-3-((trimethylsilyl)oxy)prop-1-yn-1-yl)silane (**3ag**).

Trimethyl(3-(oxiran-2-ylmethoxy)prop-1-yn-1-yl)silane (**3ah**)

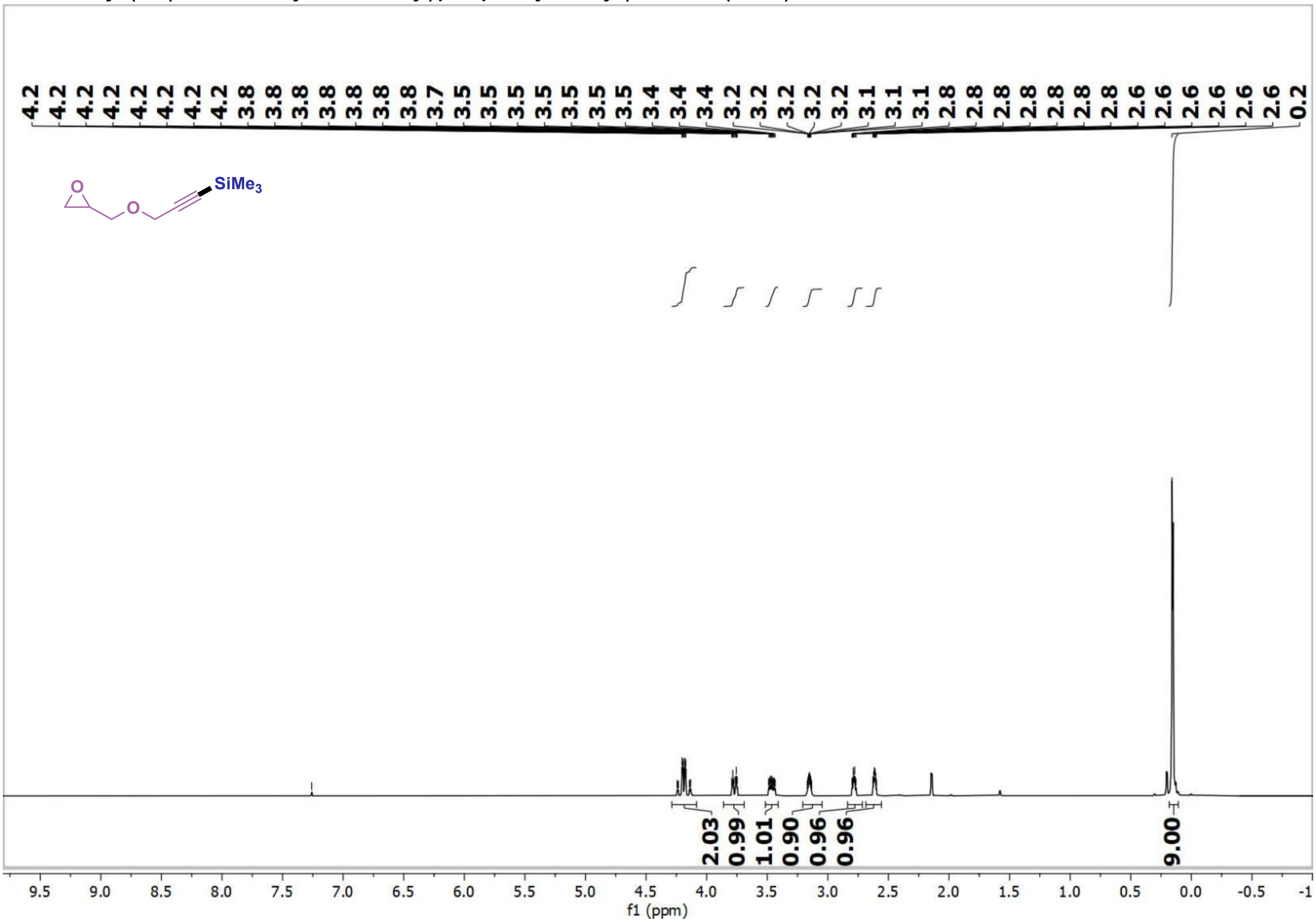


Figure S104. $^{1}$H NMR (400 MHz, Chloroform-d, 25°C) of trimethyl(3-(oxiran-2-ylmethoxy)prop-1-yn-1-yl)silane (**3ah**).

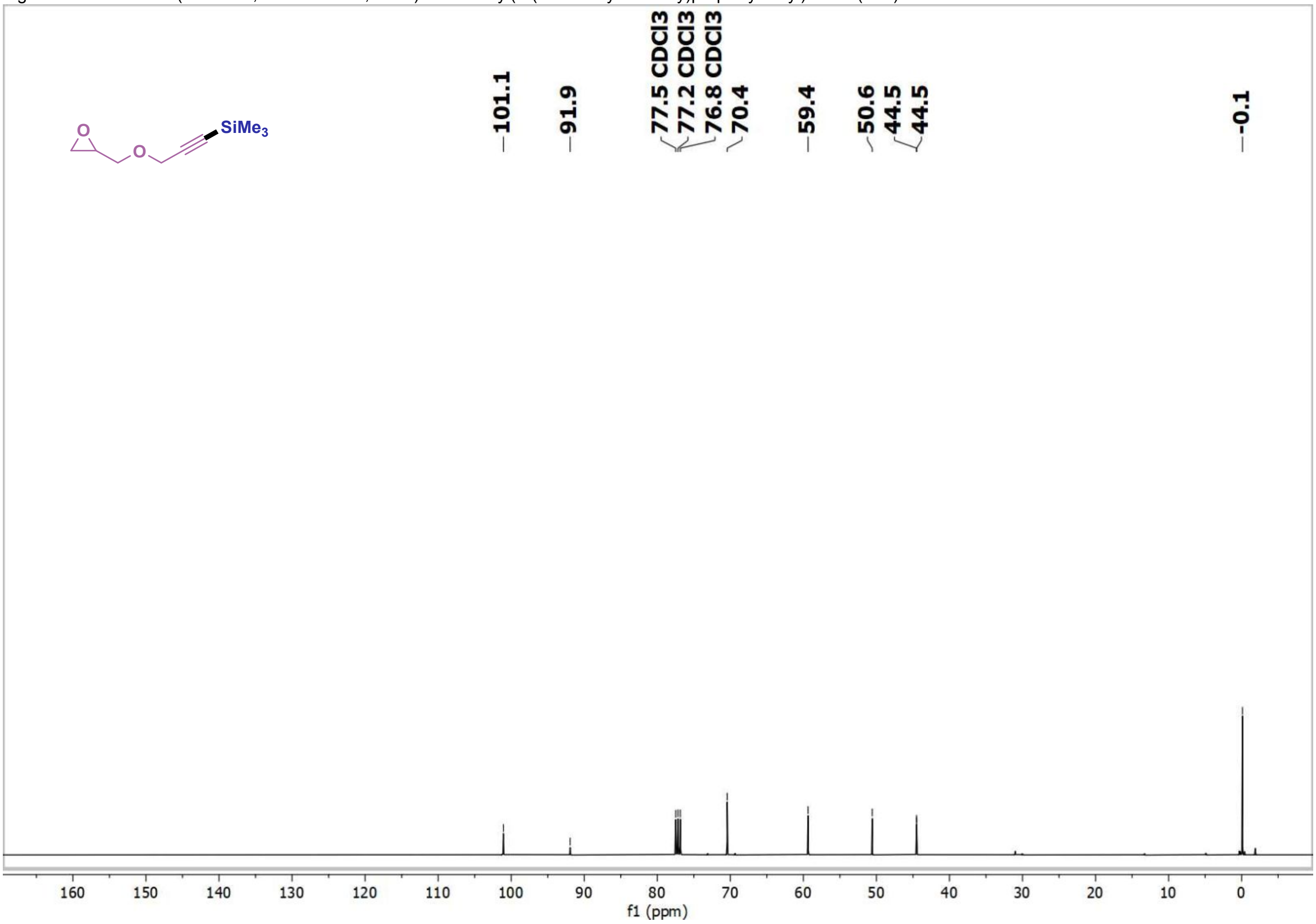


Figure S105. $^{13}$C{$^{1}$H} NMR (101 MHz, Chloroform-d, 25°C) of trimethyl(3-(oxiran-2-ylmethoxy)prop-1-yn-1-yl)silane (**3ah**).

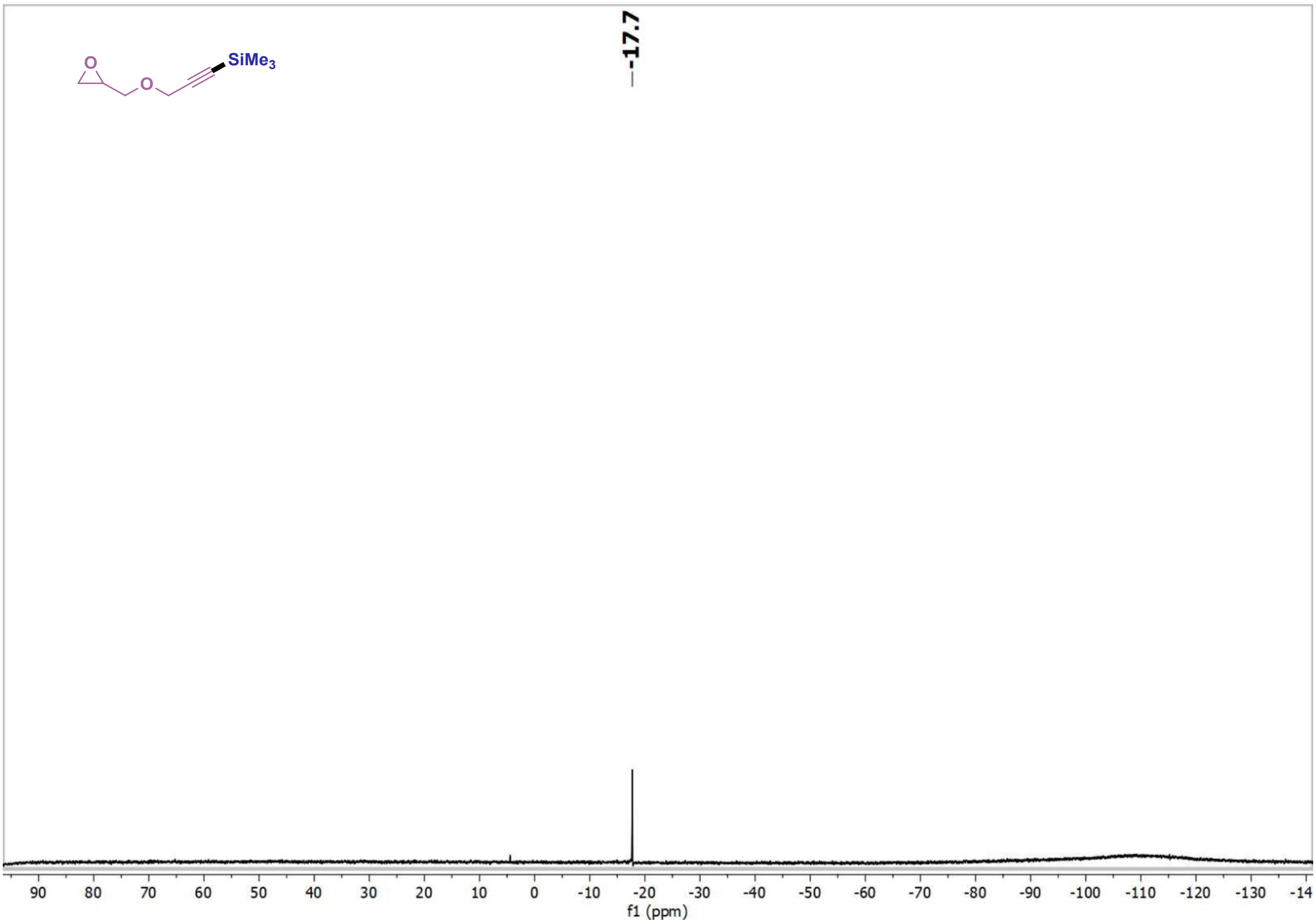


Figure S106. $^{29}Si$ NMR (79 MHz, Chloroform-d, 25°C) of trimethyl(3-(oxiran-2-ylmethoxy)prop-1-yn-1-yl)silane (**3ah**).

# Methyl 4-((trimethylsilyl)ethynyl)benzoate (**3ai**)

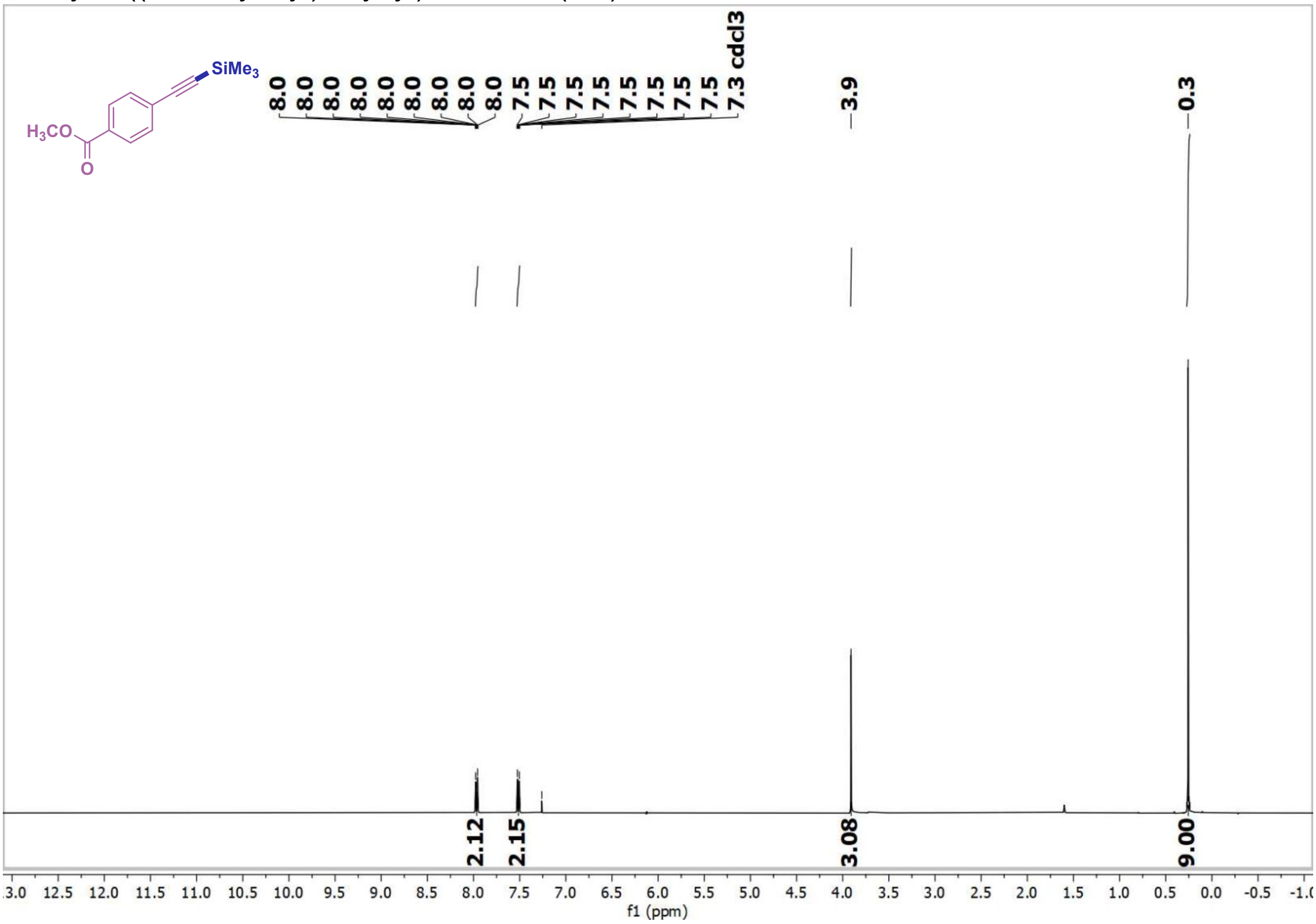


Figure S107. $^{1}$H NMR (400 MHz, Chloroform-d, 25°C) of methyl 4-((trimethylsilyl)ethynyl)benzoate (**3ai**).

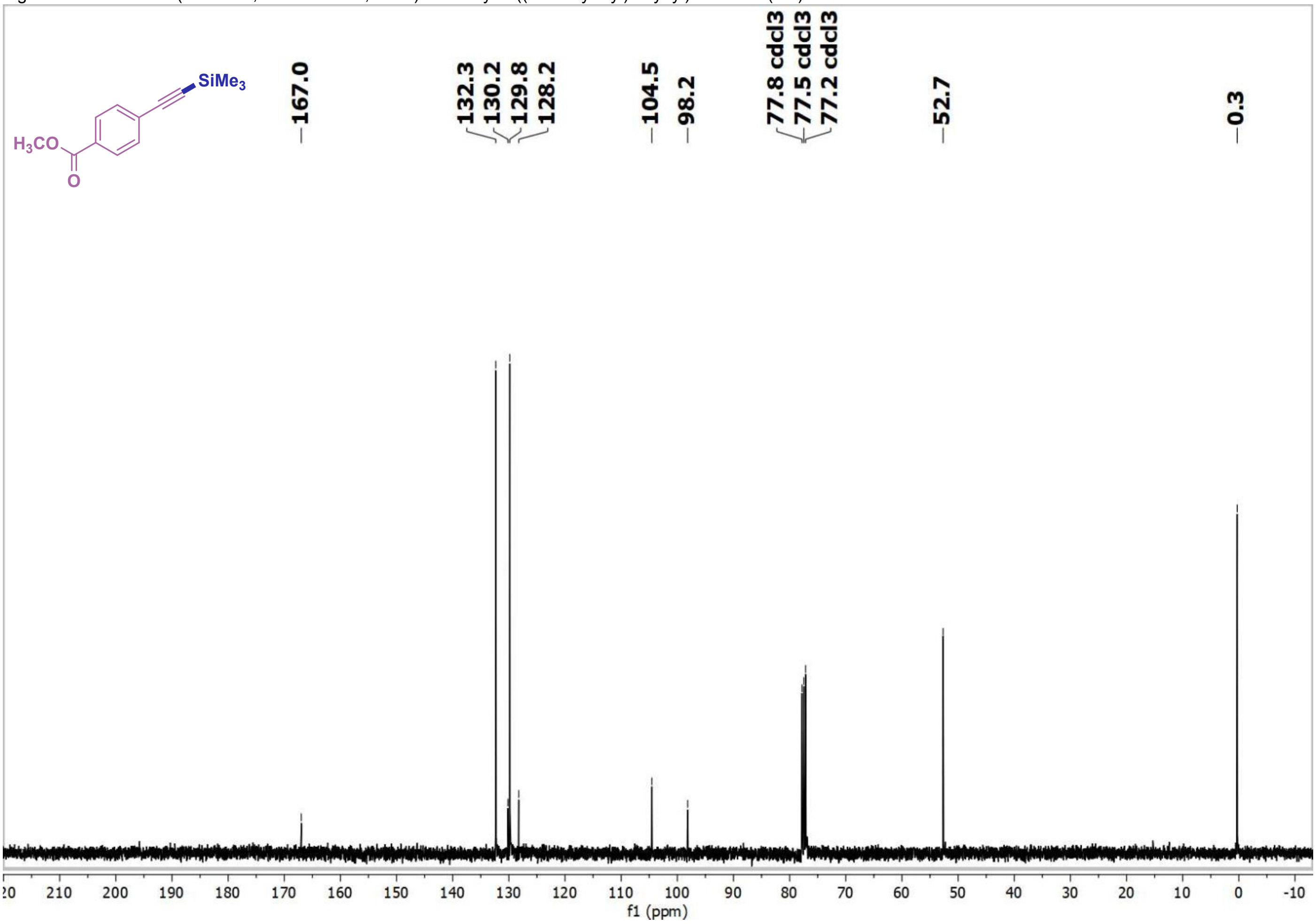


Figure S108. $^{13}$C{$^{1}$H} NMR (101 MHz, Chloroform-d, 25°C) of methyl 4-((trimethylsilyl)ethynyl)benzoate (**3ai**).

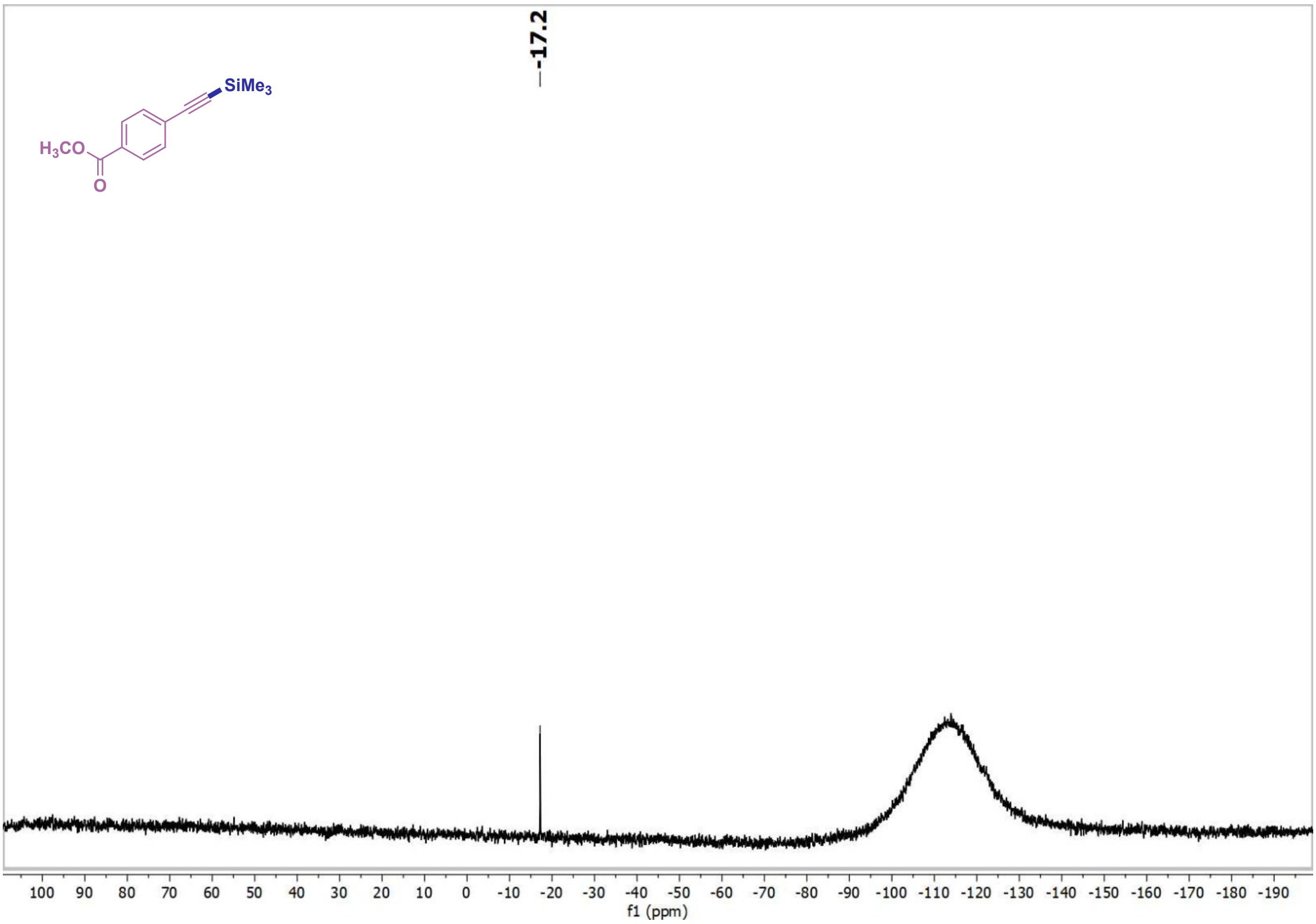


Figure S109. $^{29}Si$ NMR (79 MHz, Chloroform-d, 25°C) of methyl 4-((trimethylsilyl)ethynyl)benzoate (**3ai**).

# (1-(4-Ethynylphenyl)-2,2,2-trifluoroethoxy)trimethylsilane (**3aj**)

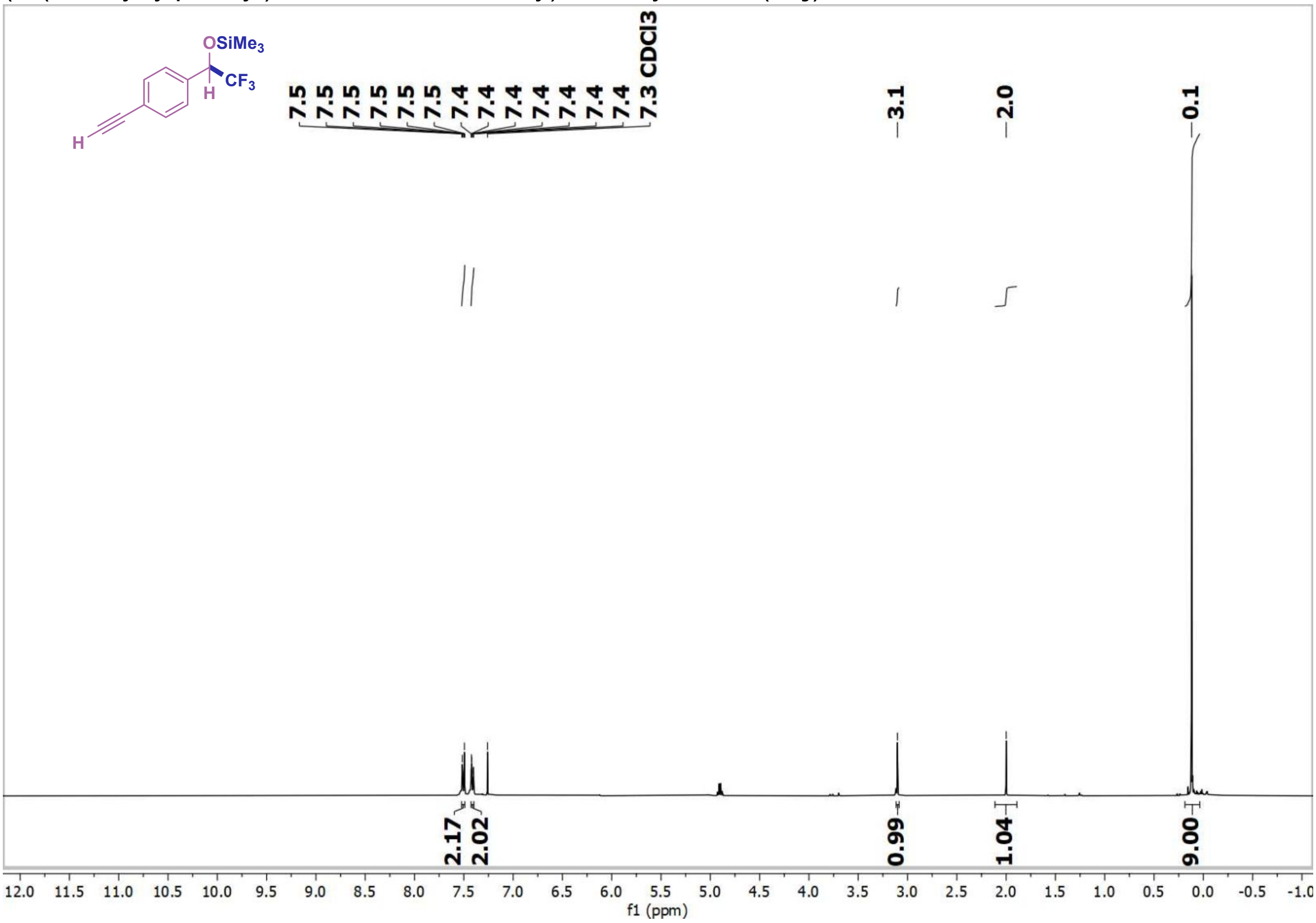


Figure S110. ¹H NMR (400 MHz, Chloroform-d, 25°C) of (1-(4-ethynylphenyl)-2,2,2-trifluoroethoxy)trimethylsilane (**3aj**).

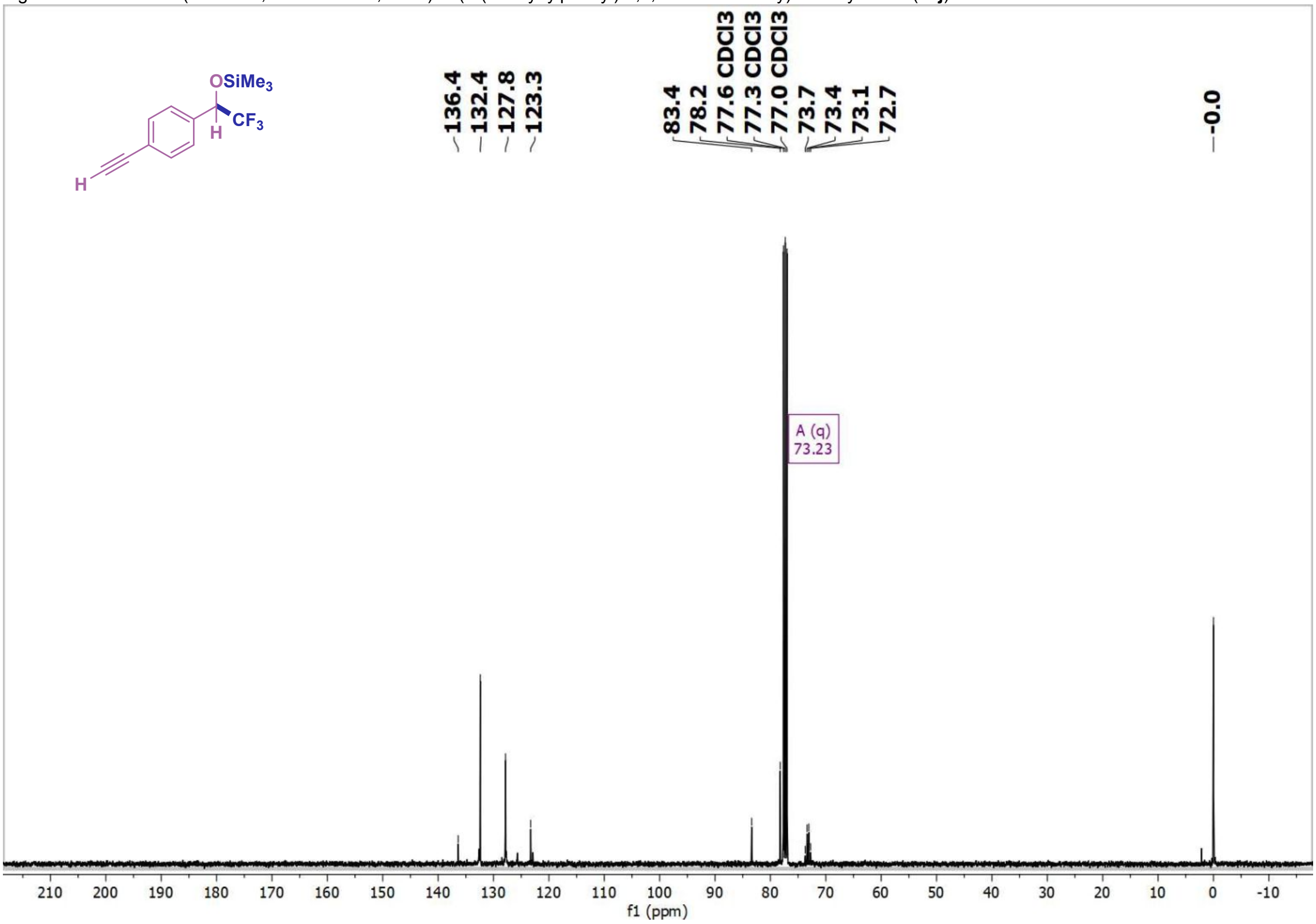


Figure S111. ¹³C{¹H} NMR (101 MHz, Chloroform-d, 25°C) of (1-(4-ethynylphenyl)-2,2,2-trifluoroethoxy)trimethylsilane (**3aj**).

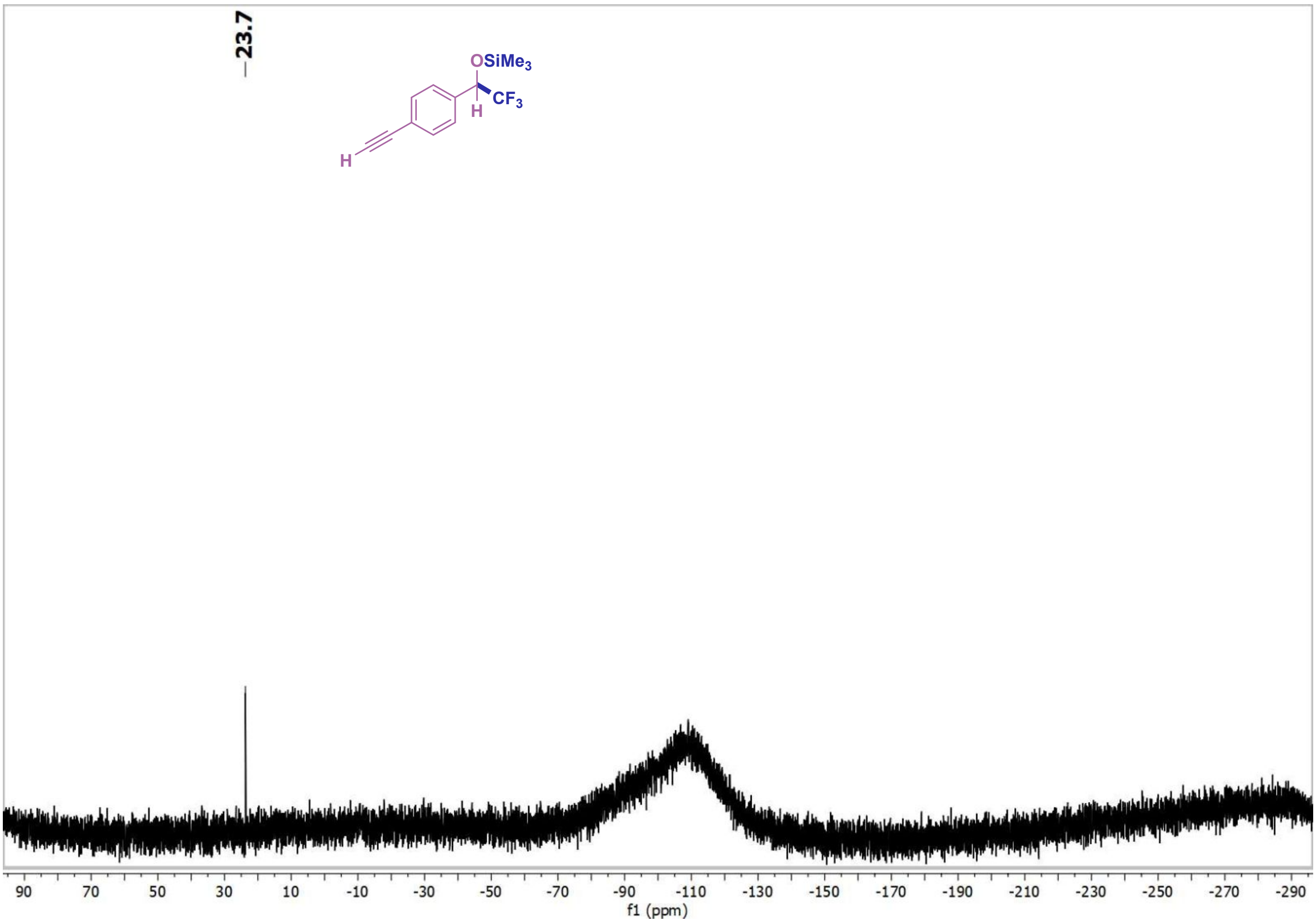


Figure S112. $^{29}Si$ NMR (79 MHz, Chloroform-d, 25°C) of (1-(4-ethynylphenyl)-2,2,2-trifluoroethoxy)trimethylsilane (**3aj**).

## *N*-benzyl-N-methyl-3-(trimethylsilyl)prop-2-yn-1-amine (**3ak**)

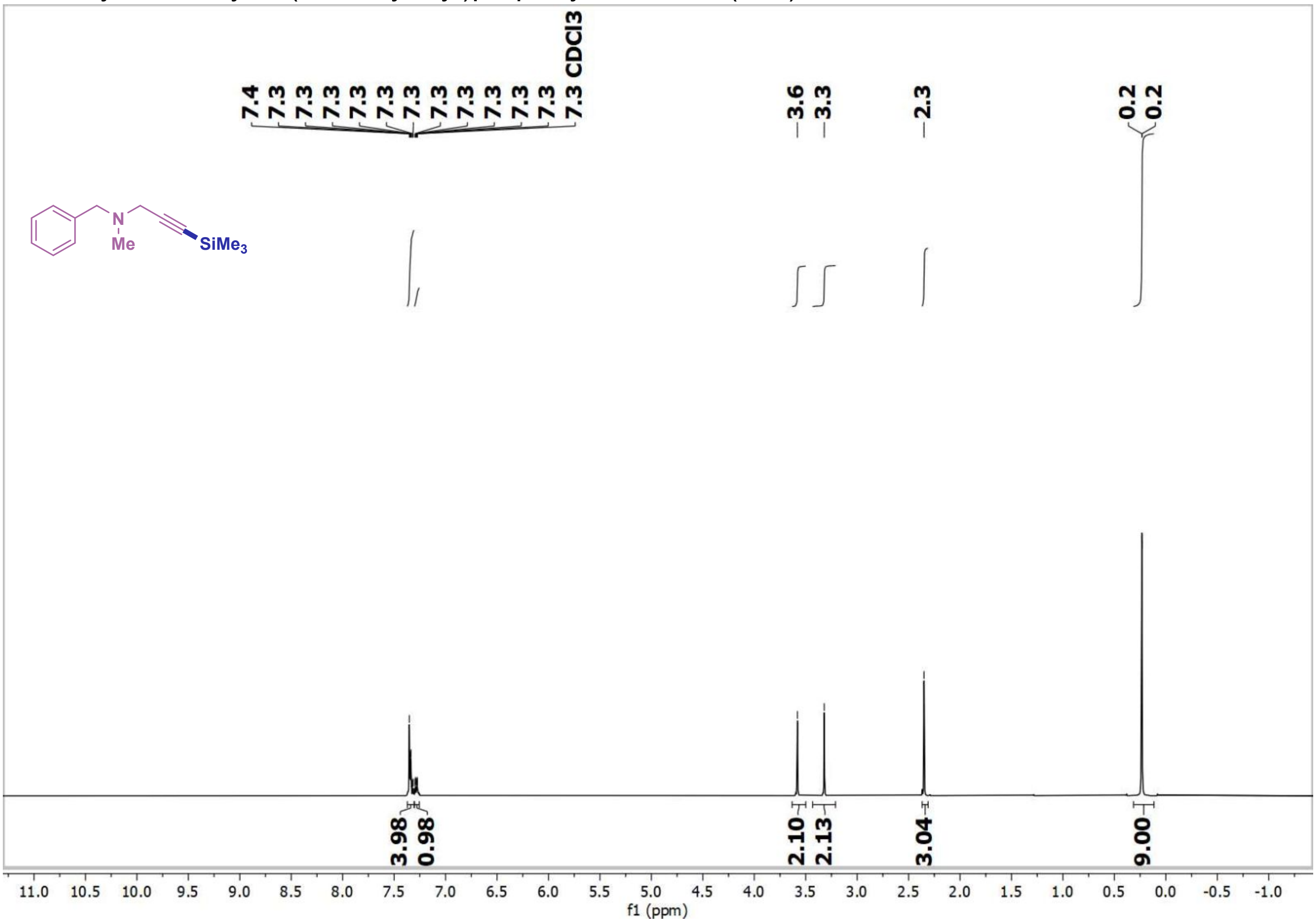


Figure S113. $^1$H NMR (400 MHz, Chloroform-d, 25°C) of *N*-benzyl-N-methyl-3-(trimethylsilyl)prop-2-yn-1-amine (**3ak**).

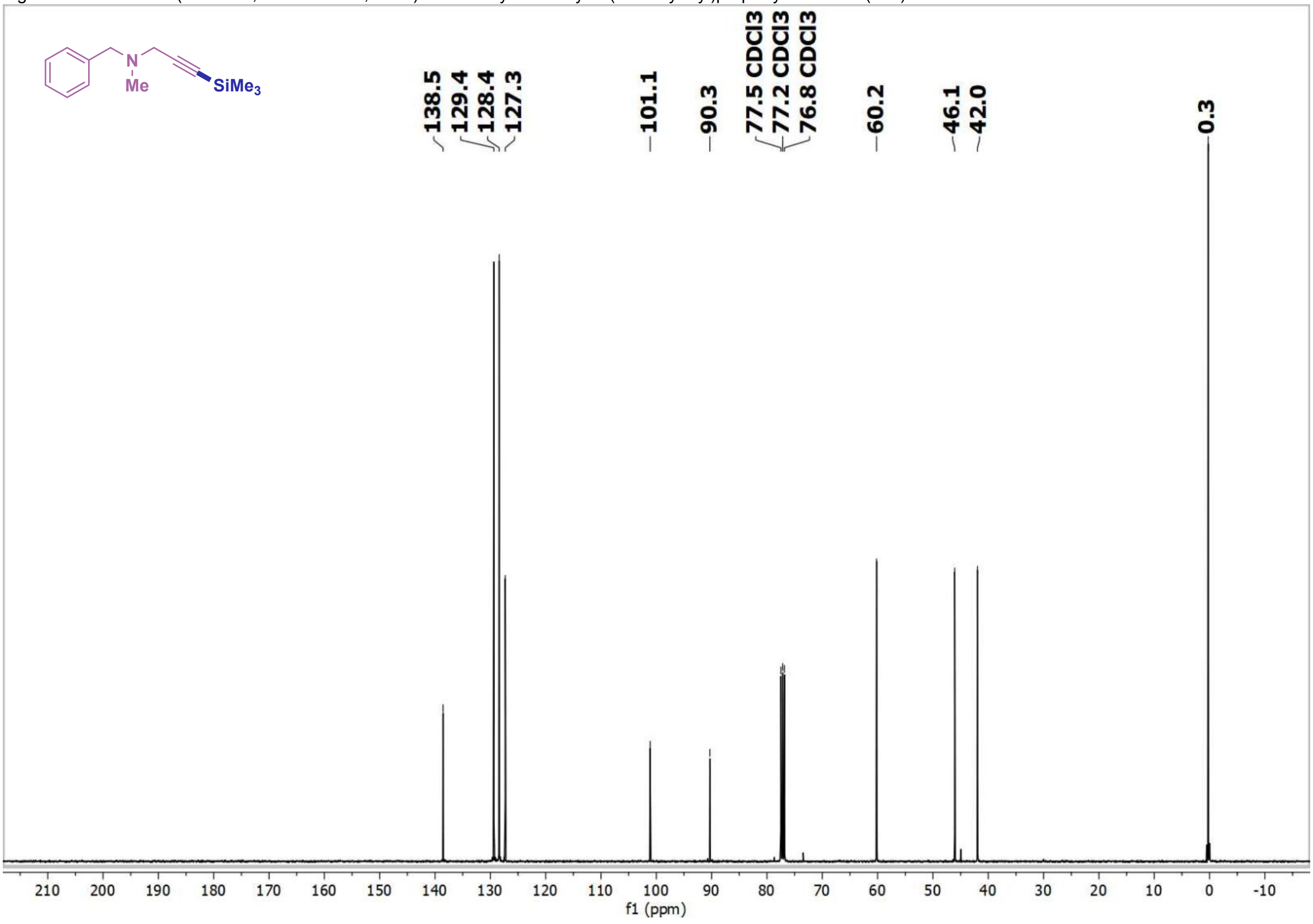


Figure S114. $^{13}$C{$^1$H} NMR (101 MHz, Chloroform-d, 25°C) of *N*-benzyl-N-methyl-3-(trimethylsilyl)prop-2-yn-1-amine (**3ak**).

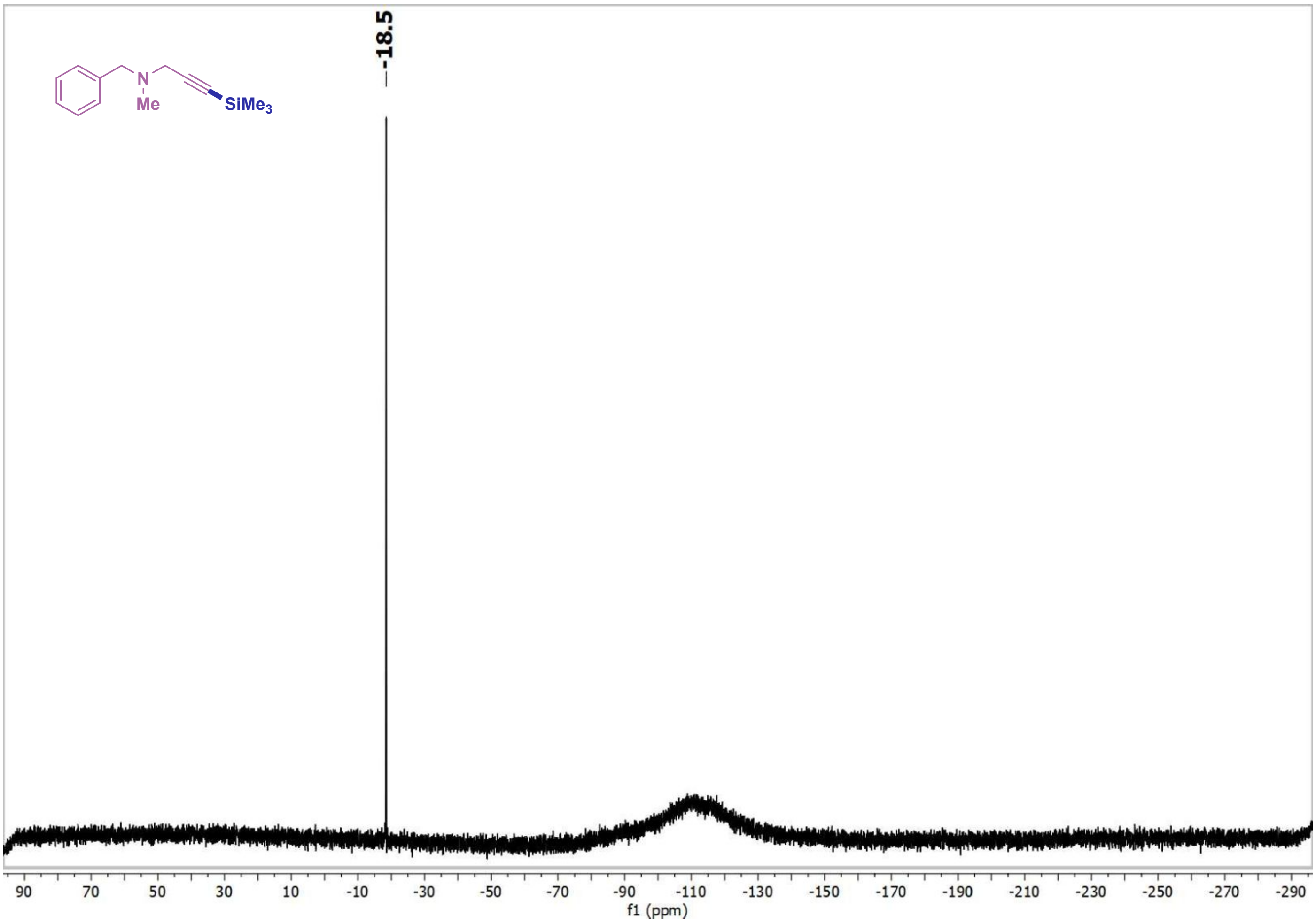


Figure S115. $^{29}Si$ NMR (79 MHz, Chloroform-d, 25°C) of *N*-benzyl-N-methyl-3-(trimethylsilyl)prop-2-yn-1-amine (**3ak**).

(*R*)-*N*-(3-(trimethylsilyl)prop-2-yn-1-yl)-2,3-dihydro-1H-inden-1-amine (**3al**)

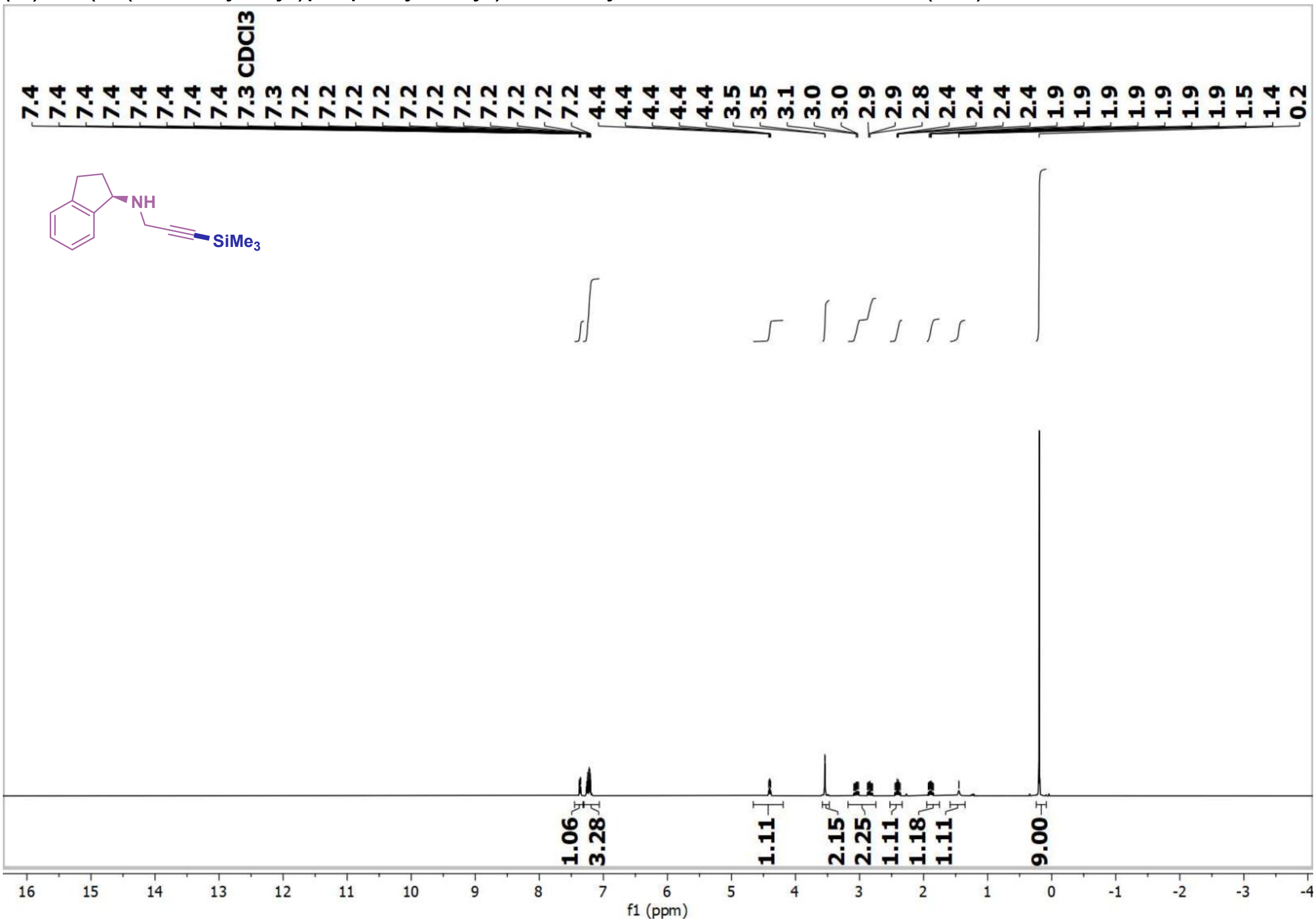


Figure S116. $^{1}$H NMR (400 MHz, Chloroform-d, 25°C) of *(R)*-*N*-(3-(trimethylsilyl)prop-2-yn-1-yl)-2,3-dihydro-1H-inden-1-amine (**3al**).

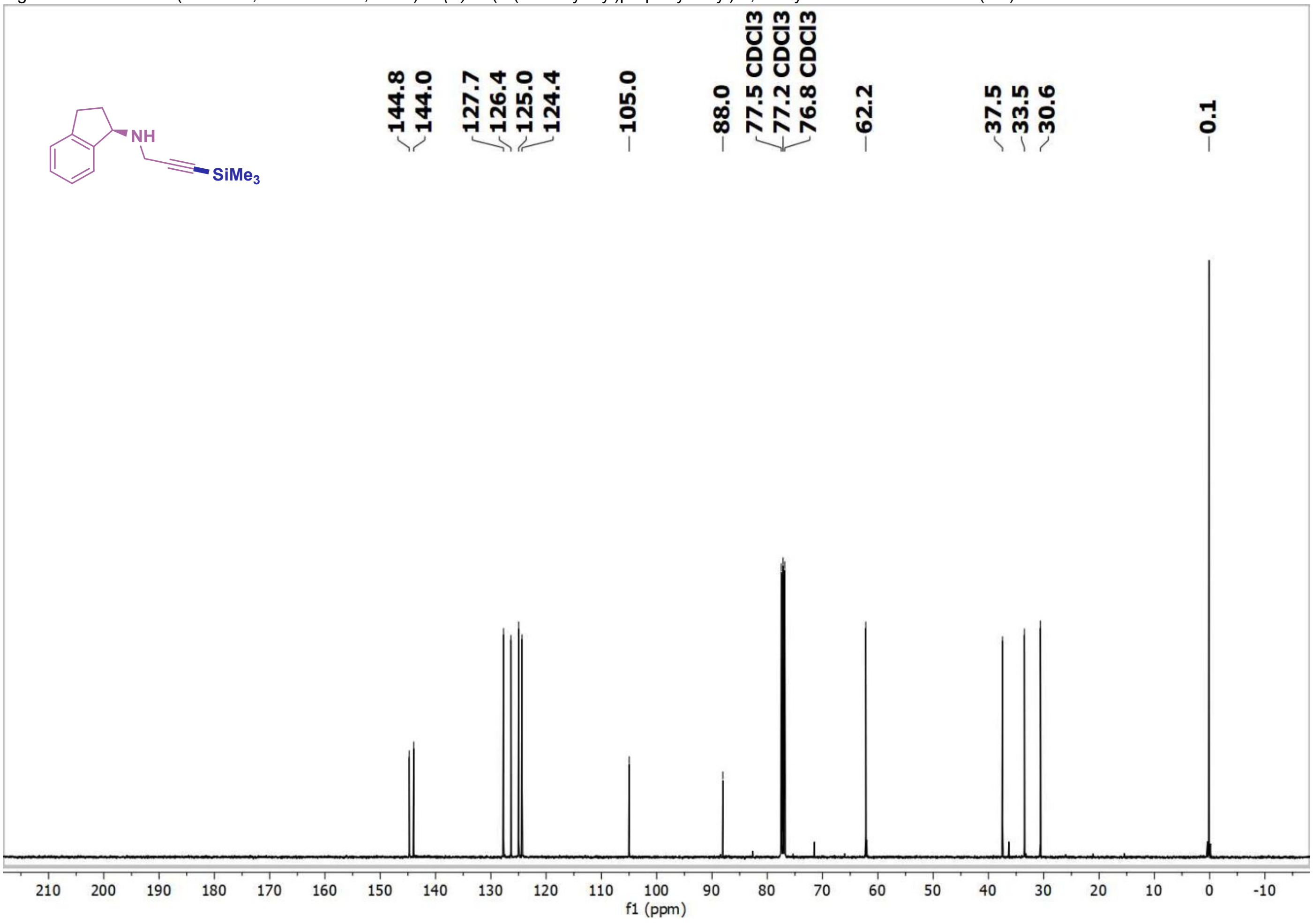


Figure S117. $^{13}$C{$^{1}$H} NMR (101 MHz, Chloroform-d, 25°C) of *(R)*-*N*-(3-(trimethylsilyl)prop-2-yn-1-yl)-2,3-dihydro-1H-inden-1-amine (**3al**).

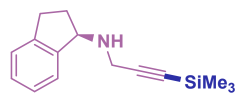


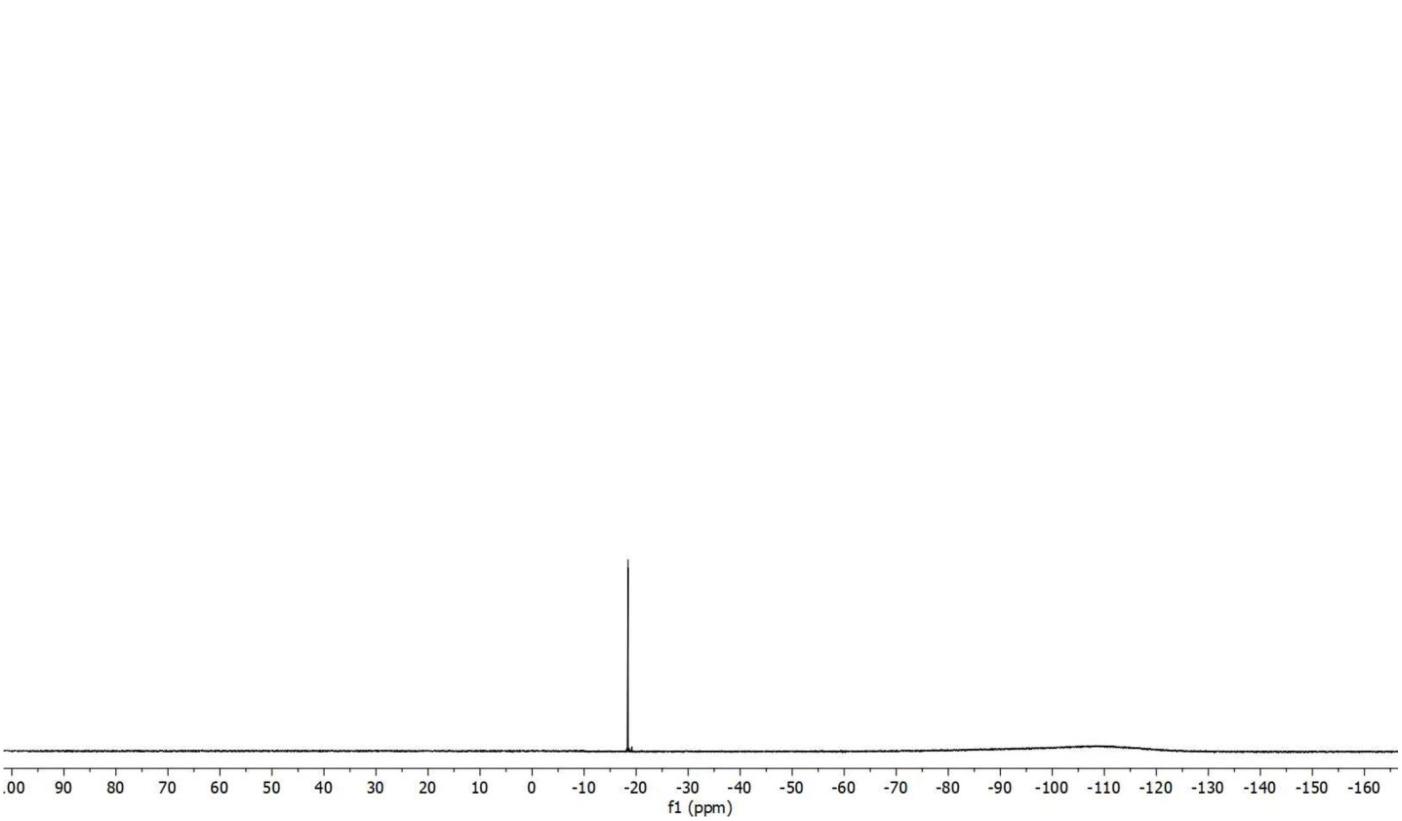


Figure S118. $^{29}Si$ NMR (79 MHz, Chloroform-d, 25°C) of *(R)-N*-(3-(trimethylsilyl)prop-2-yn-1-yl)-2,3-dihydro-1H-inden-1-amine (**3al**).

## Trimethyl(1-(*o*-tolyl)ethoxy)silane (**5a**)

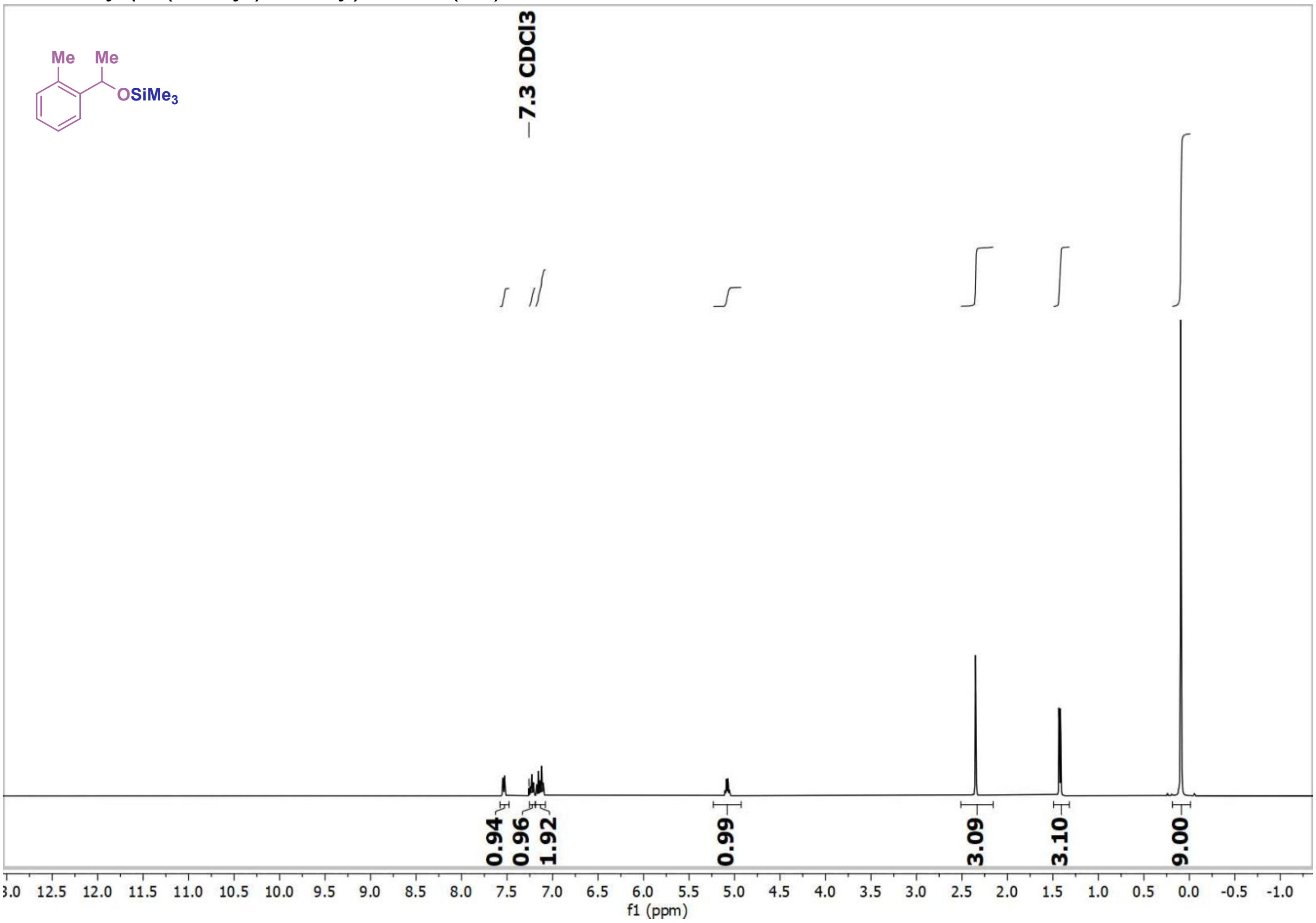


Figure S119. $^{1}$H NMR (400 MHz, Chloroform-d, 25°C) of trimethyl(1-(*o*-tolyl)ethoxy)silane (**5a**).

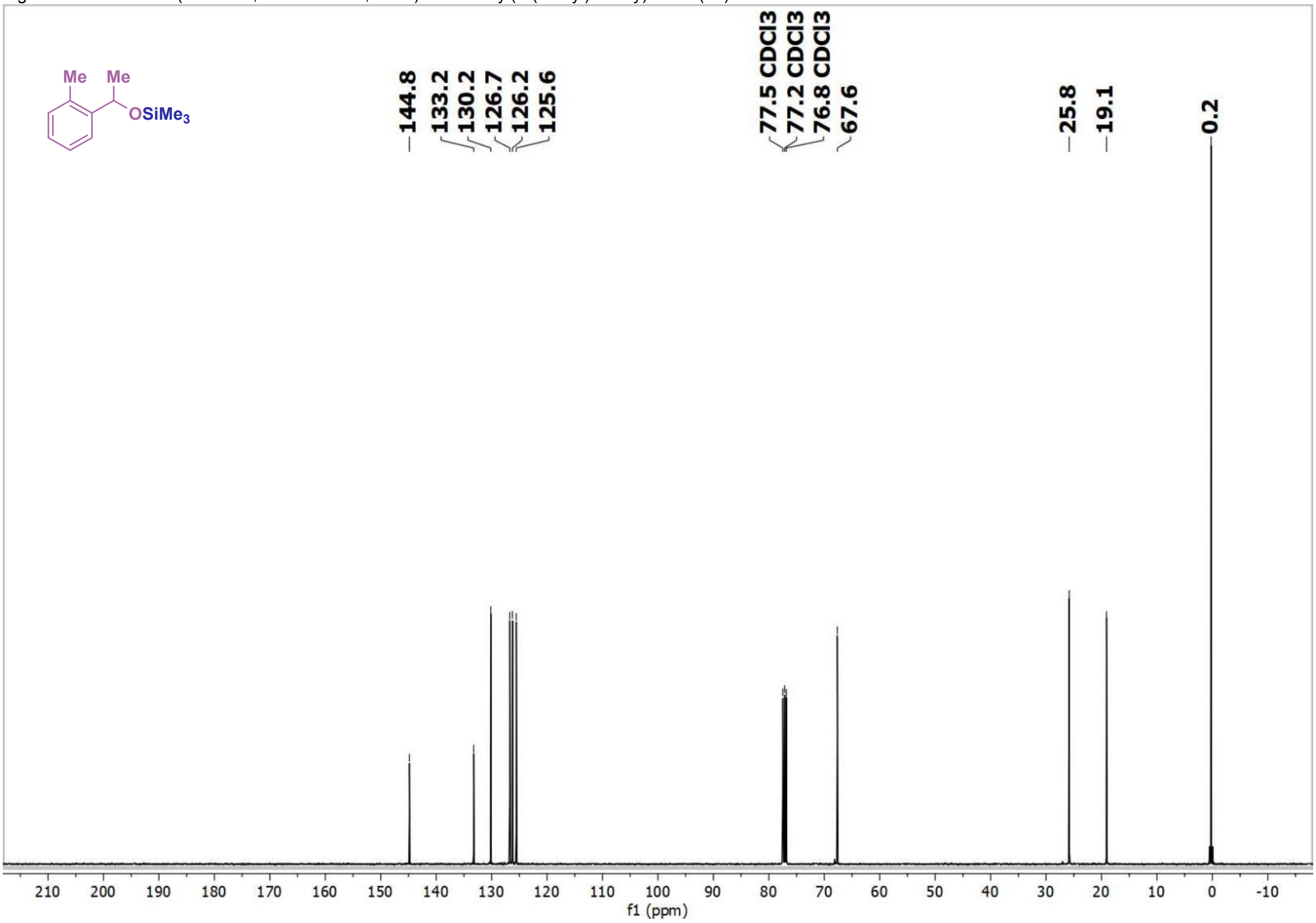


Figure S120. $^{13}C\{^{1}H\}$ NMR (101 MHz, Chloroform-d, 25°C) of trimethyl(1-(*o*-tolyl)ethoxy)silane (**5a**).

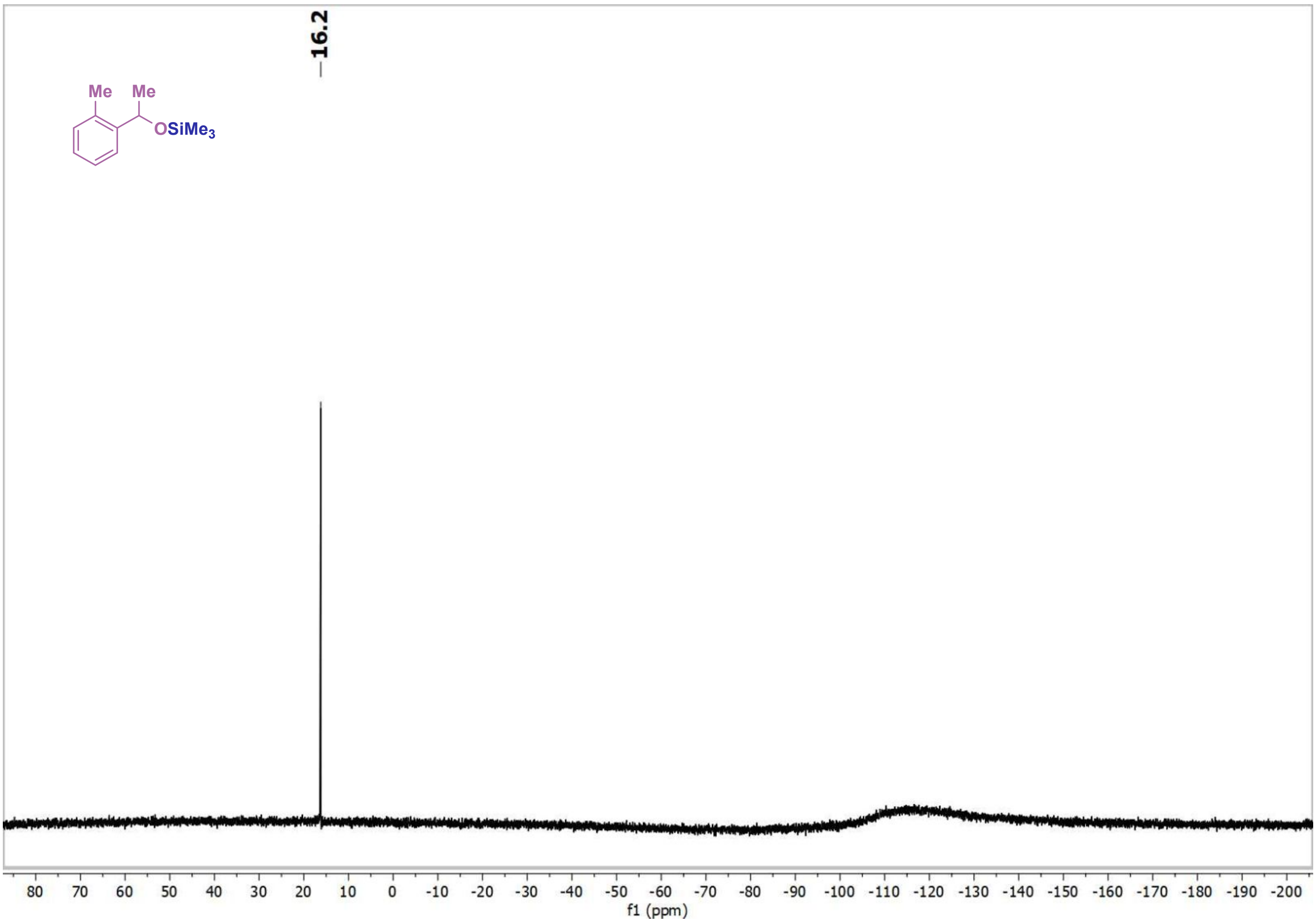


Figure S121. $^{29}Si$ NMR (79 MHz, Chloroform-d, 25°C) of trimethyl(1-(*o*-tolyl)ethoxy)silane (**5a**).

## (1-(3-Methoxyphenyl)ethoxy)trimethylsilane (**5b**)

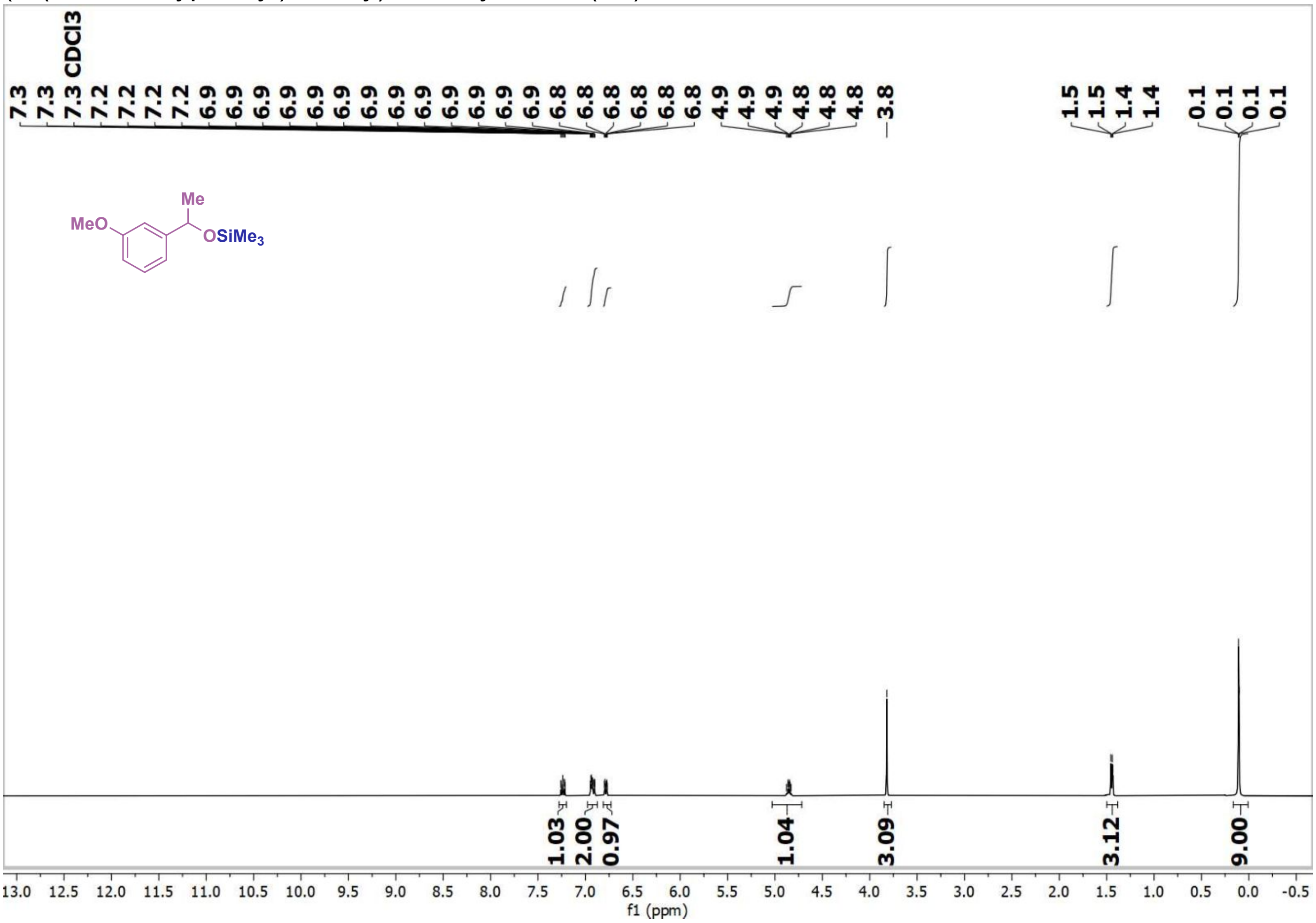


Figure S122. $^{1}H$ NMR (400 MHz, Chloroform-d, 25°C) of (1-(3-methoxyphenyl)ethoxy)trimethylsilane (**5b**).

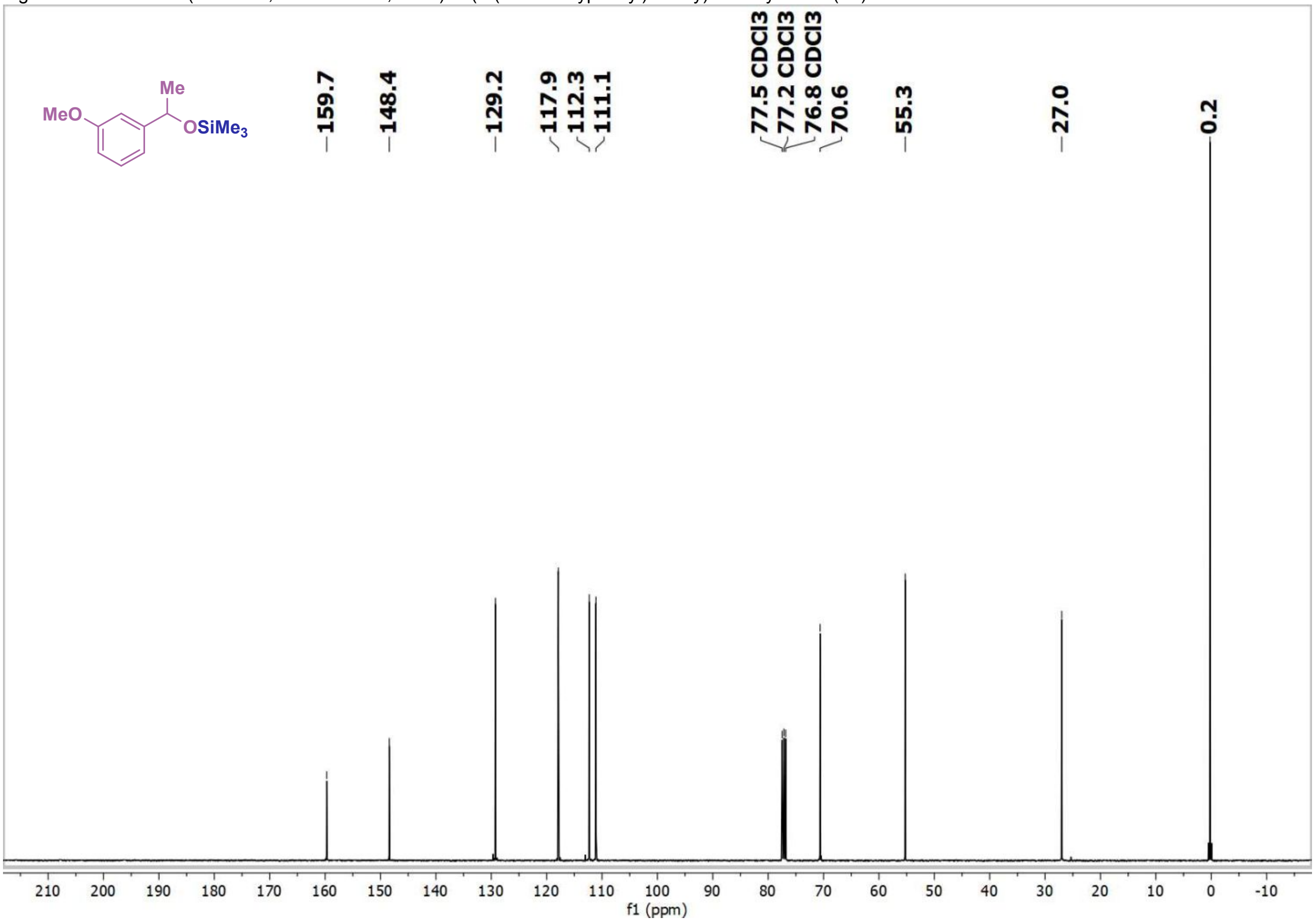


Figure S123. $^{13}C\{^{1}H\}$ NMR (101 MHz, Chloroform-d, 25°C) of (1-(3-methoxyphenyl)ethoxy)trimethylsilane (**5b**).

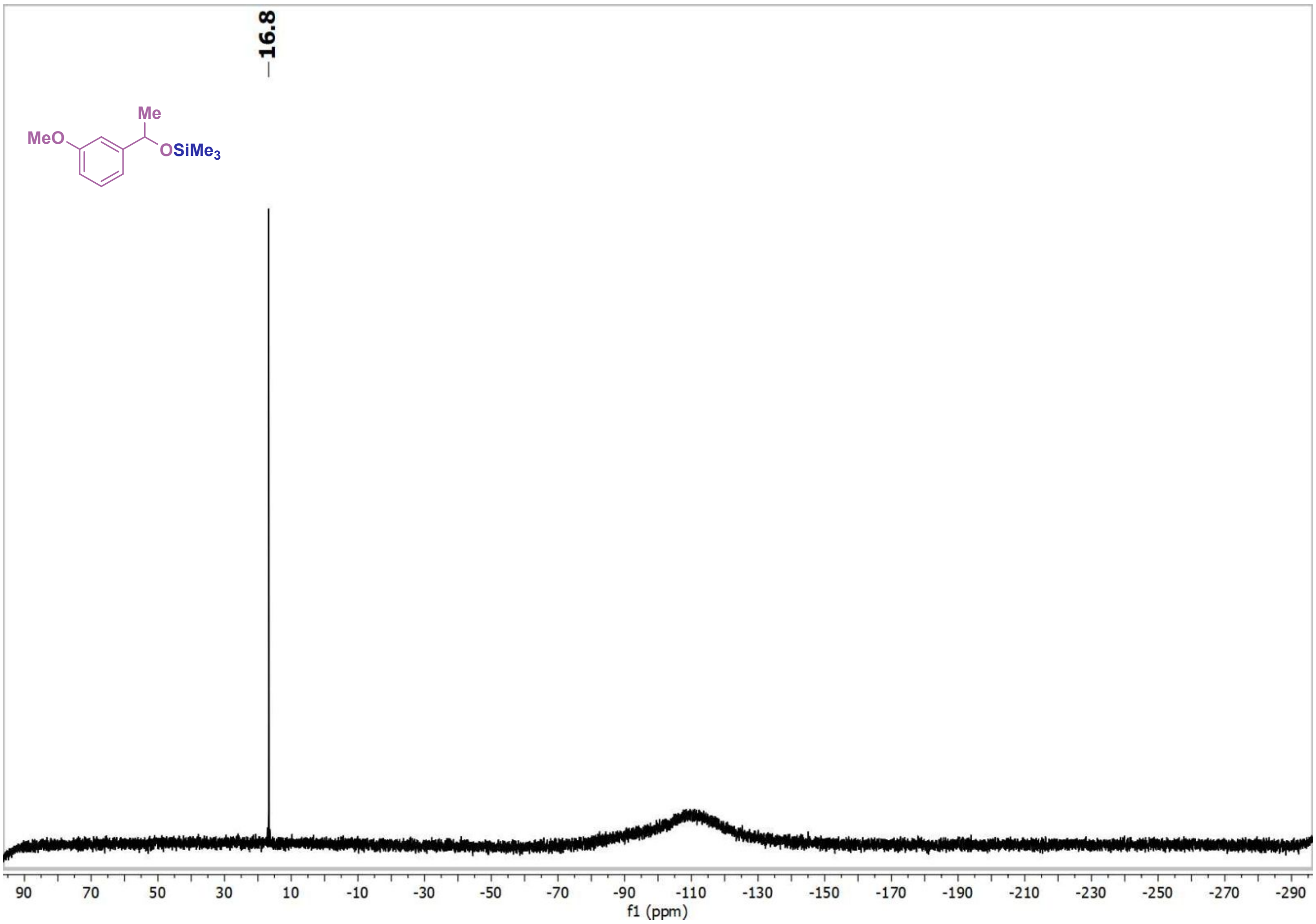


Figure S124. $^{29}Si$ NMR (79 MHz, Chloroform-d, 25°C) of (1-(3-methoxyphenyl)ethoxy)trimethylsilane (**5b**).

# (1-(4-Chlorophenyl)ethoxy)trimethylsilane (**5c**)

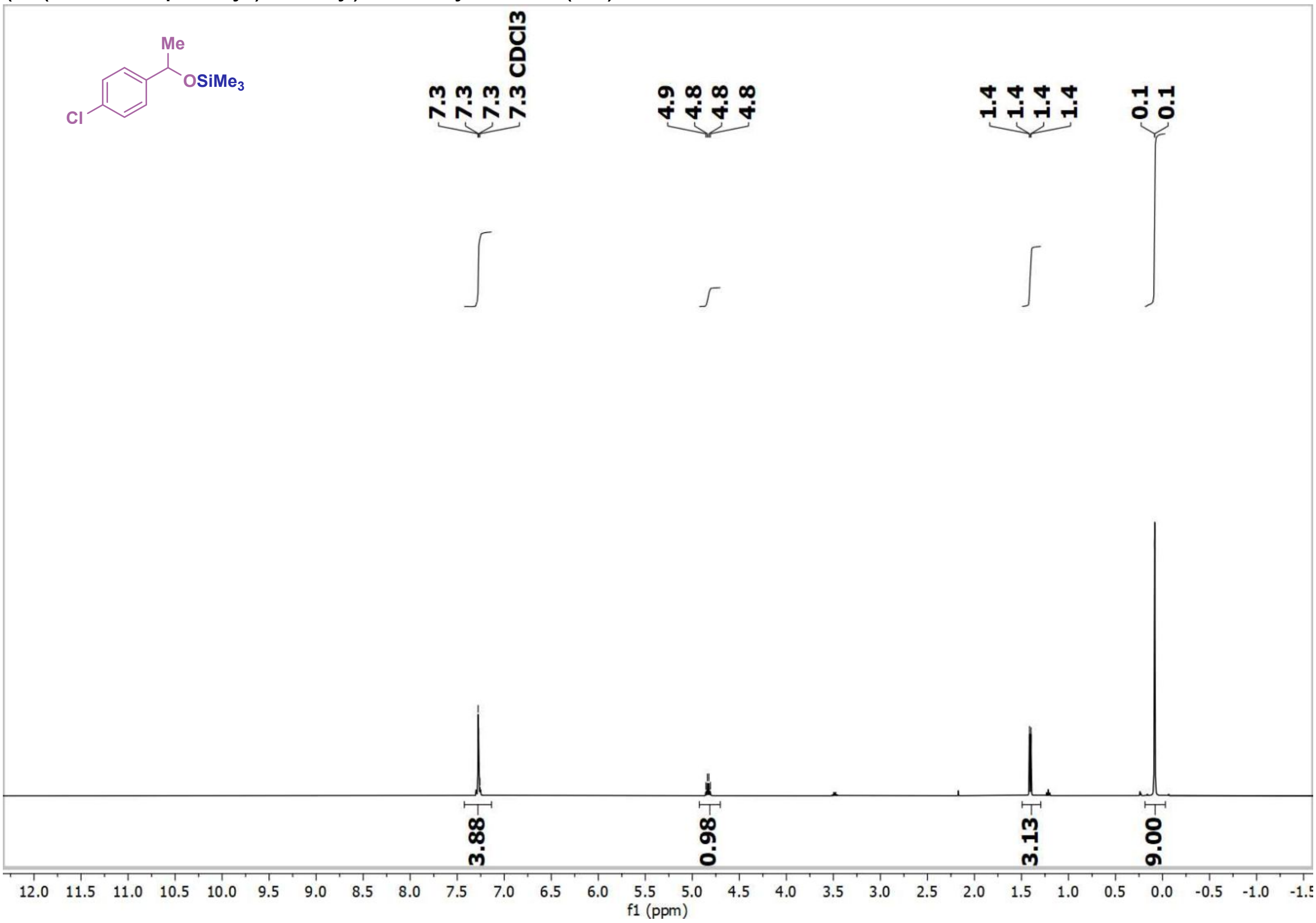


Figure S125. $^{1}H$ NMR (400 MHz, Chloroform-d, 25°C) of (1-(4-chlorophenyl)ethoxy)trimethylsilane (**5c**).

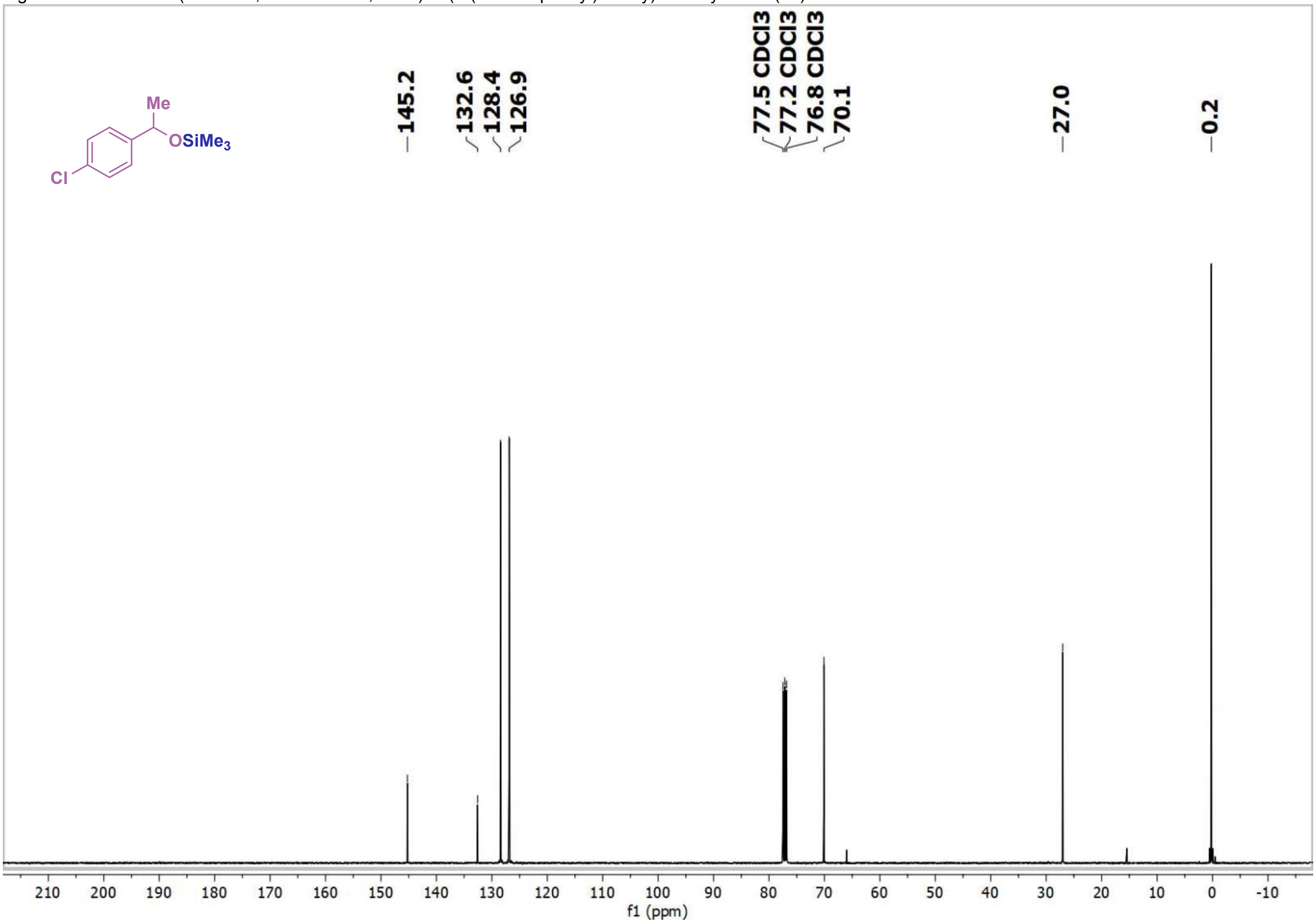


Figure S126. $^{13}C\{^{1}H\}$ NMR (101 MHz, Chloroform-d, 25°C) of (1-(4-chlorophenyl)ethoxy)trimethylsilane (**5c**).

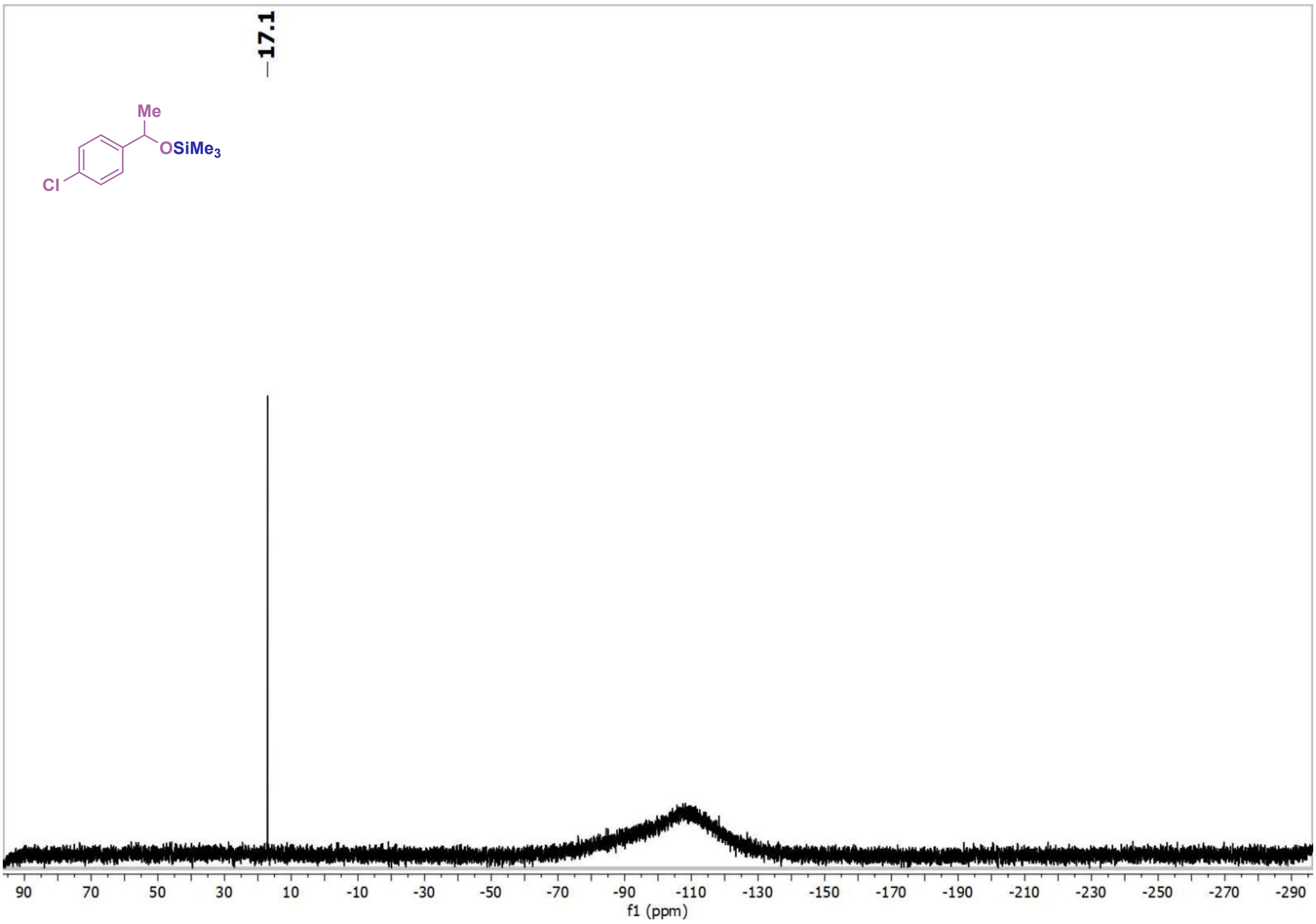


Figure S127. $^{29}$Si NMR (79 MHz, Chloroform-d, 25°C) of (1-(4-chlorophenyl)ethoxy)trimethylsilane (**5c**).

1,1,1-Trimethyl-3,3,3-tripropyldisiloxane (**5d**)

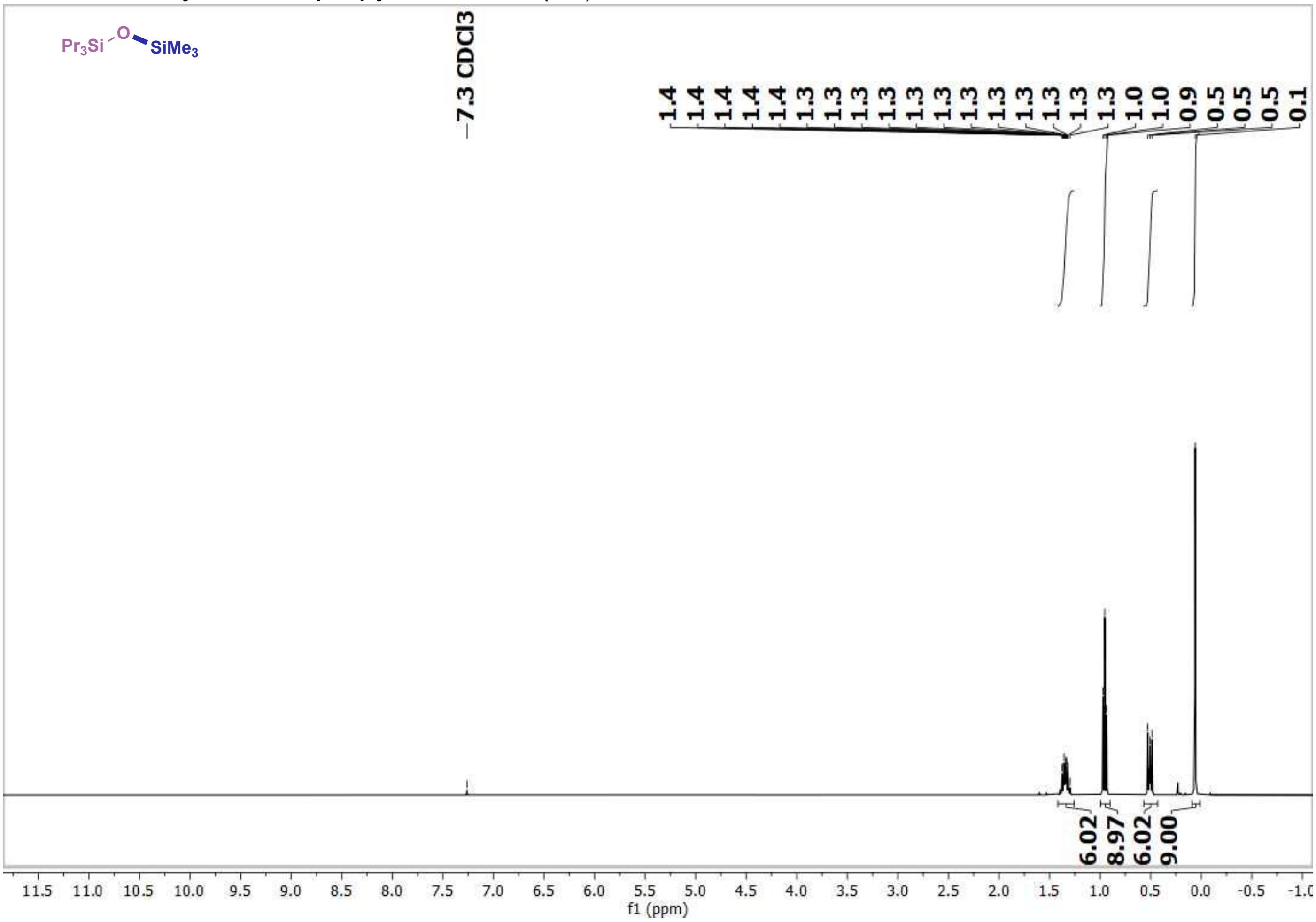


Figure S128. $^{1}H$ NMR (400 MHz, Chloroform-d, 25°C) of 1,1,1-trimethyl-3,3,3-tripropyldisiloxane (**5d**).

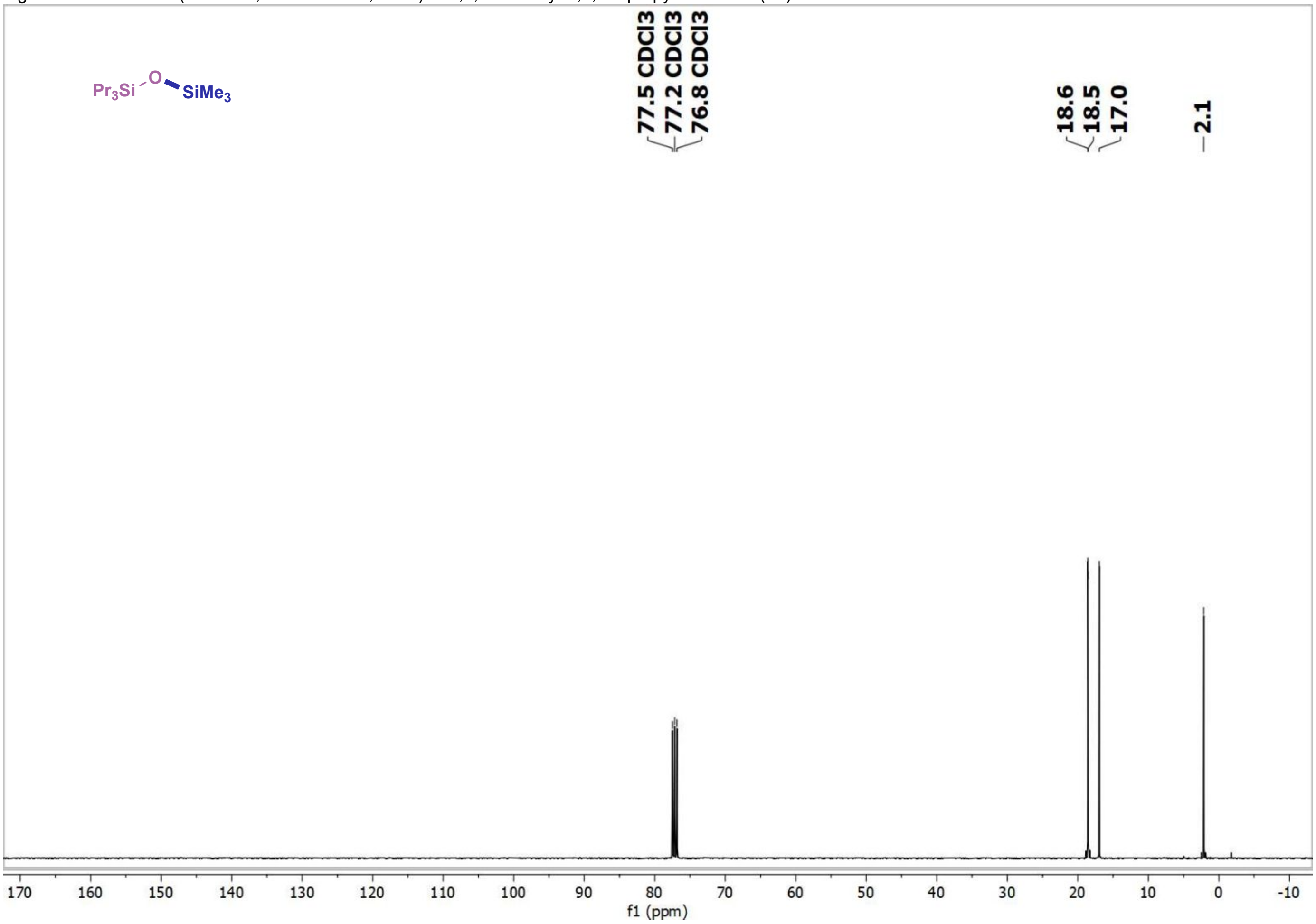


Figure S129. $^{13}C\{^{1}H\}$ NMR (101 MHz, Chloroform-d, 25°C) of 1,1,1-trimethyl-3,3,3-tripropyldisiloxane (**5d**).

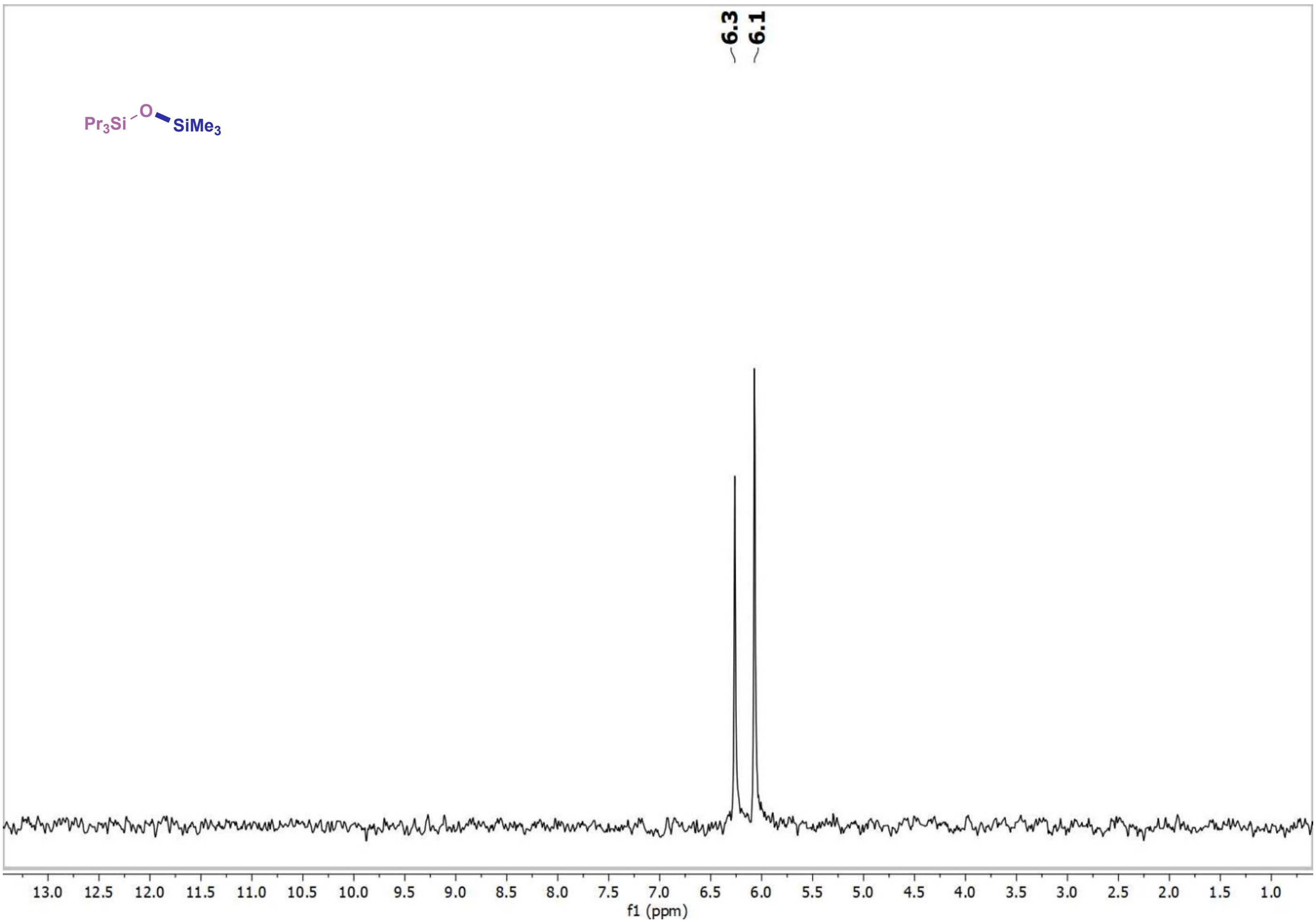


Figure S130. $^{29}$Si NMR (79 MHz, Chloroform-d, 25°C) of 1,1,1-trimethyl-3,3,3-tripropyldisiloxane (**5d**).

1,1,1-Trimethyl-3,3,3-triisopropyldisiloxane (**5e**)

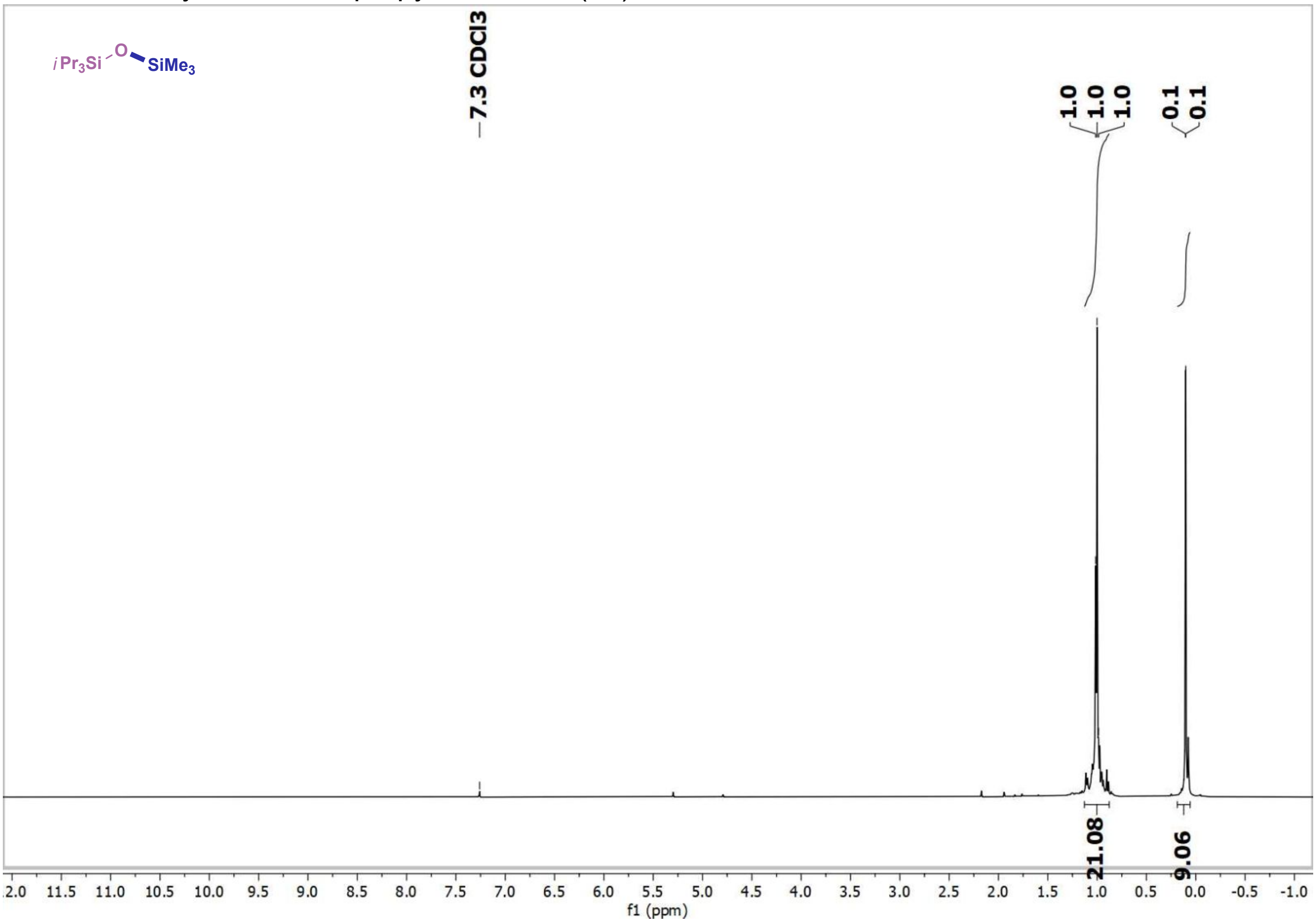


Figure S131. $^{1}$H NMR (400 MHz, Chloroform-d, 25°C) of 1,1,1-trimethyl-3,3,3-triisopropyldisiloxane (**5e**).

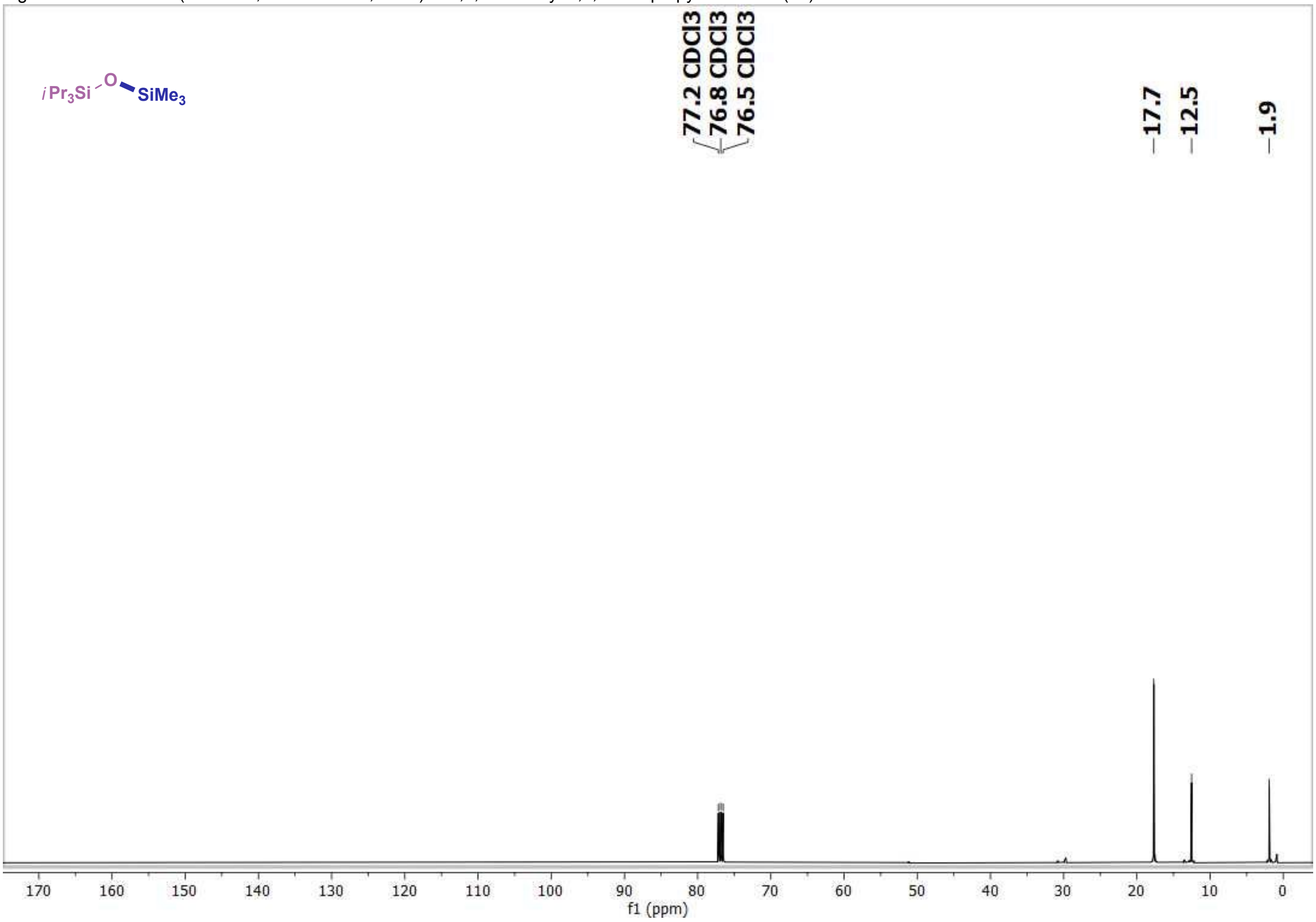


Figure S132. $^{13}$C{$^{1}$H} NMR (101 MHz, Chloroform-d, 25°C) of 1,1,1-trimethyl-3,3,3-triisopropyldisiloxane (**5e**).

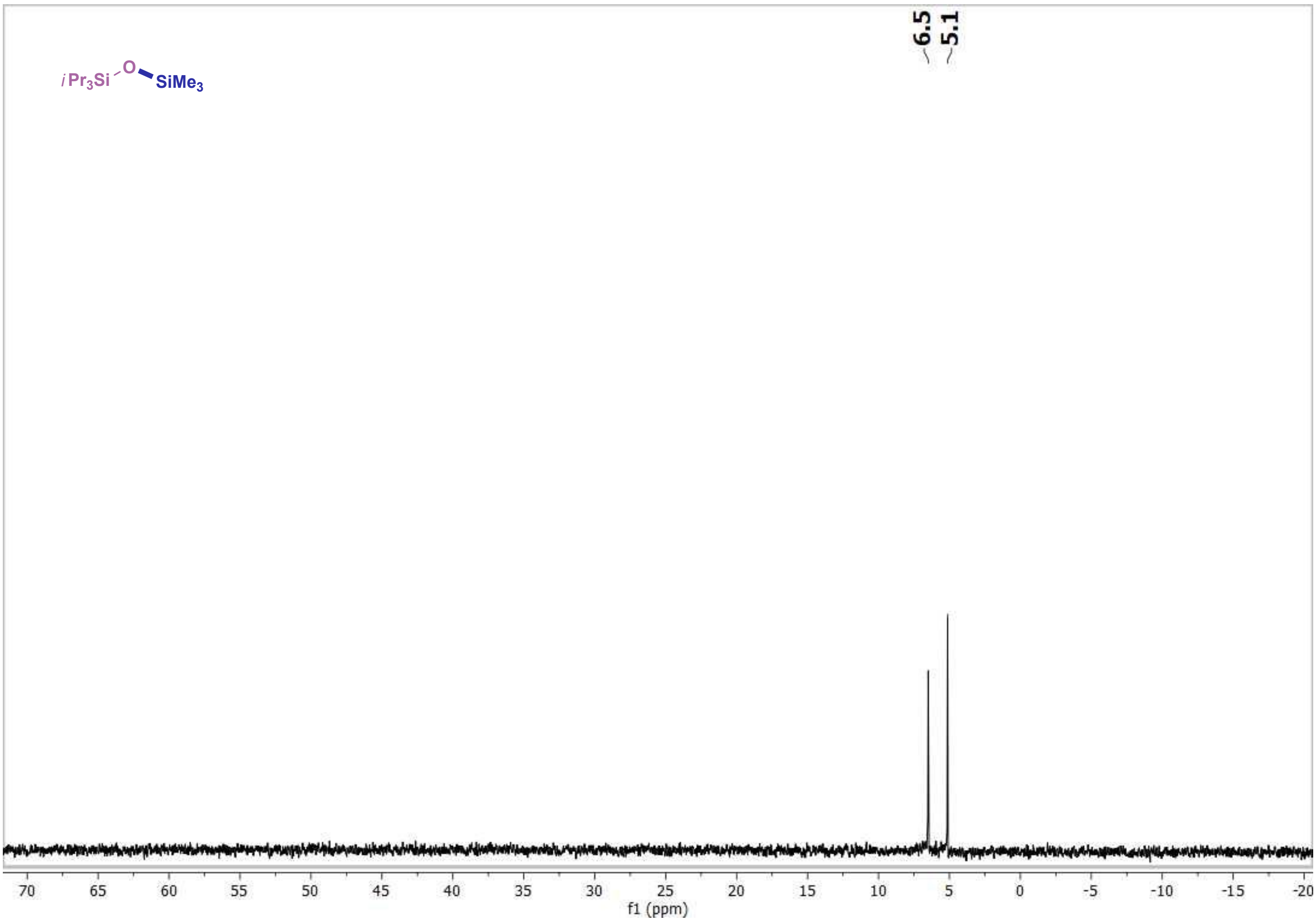


Figure S133. $^{29}$Si NMR (79 MHz, Chloroform-d, 25°C) of 1,1,1-trimethyl-3,3,3-triisopropyldisiloxane (**5e**).

1-(*Tert*-butyl)-3,3,3-trimethyl-1,1-diphenyldisiloxane (**5f**)

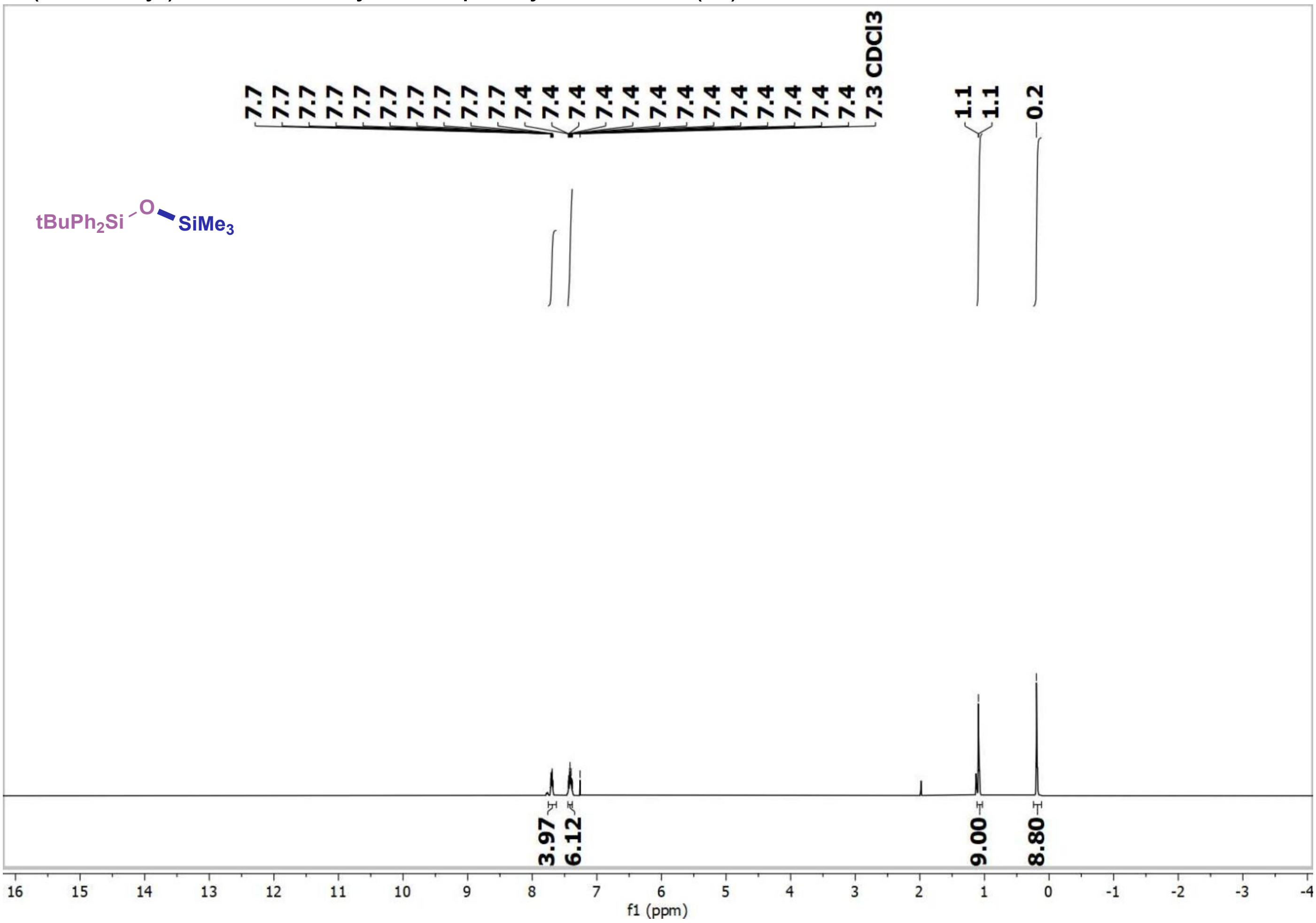


Figure S134. $^{1}H$ NMR (400 MHz, Chloroform-d, 25°C) of 1-(*tert*-butyl)-3,3,3-trimethyl-1,1-diphenyldisiloxane (**5f**).

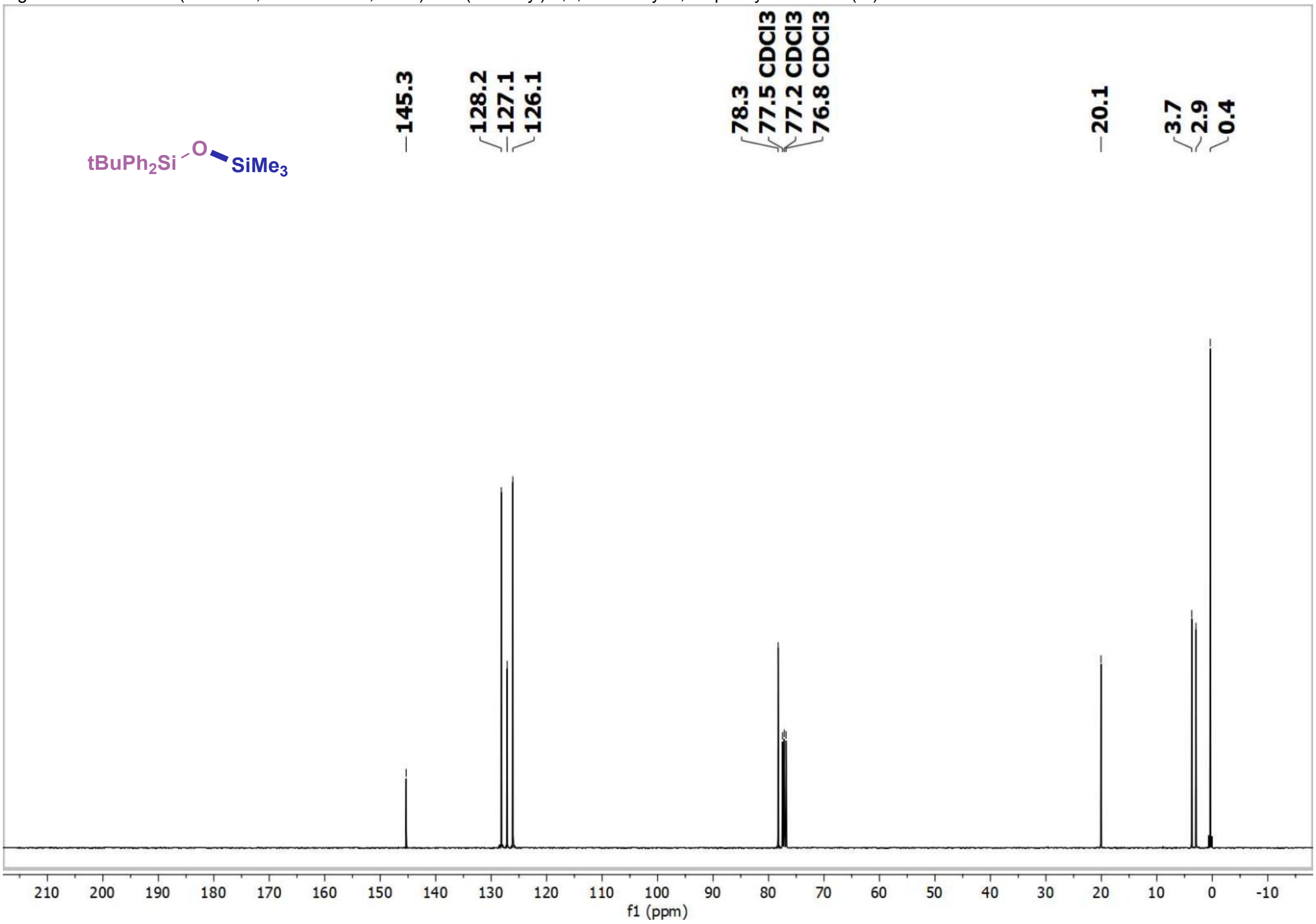


Figure S135. $^{13}C\{^{1}H\}$ NMR (101 MHz, Chloroform-d, 25°C) of 1-(*tert*-butyl)-3,3,3-trimethyl-1,1-diphenyldisiloxane (**5f**).

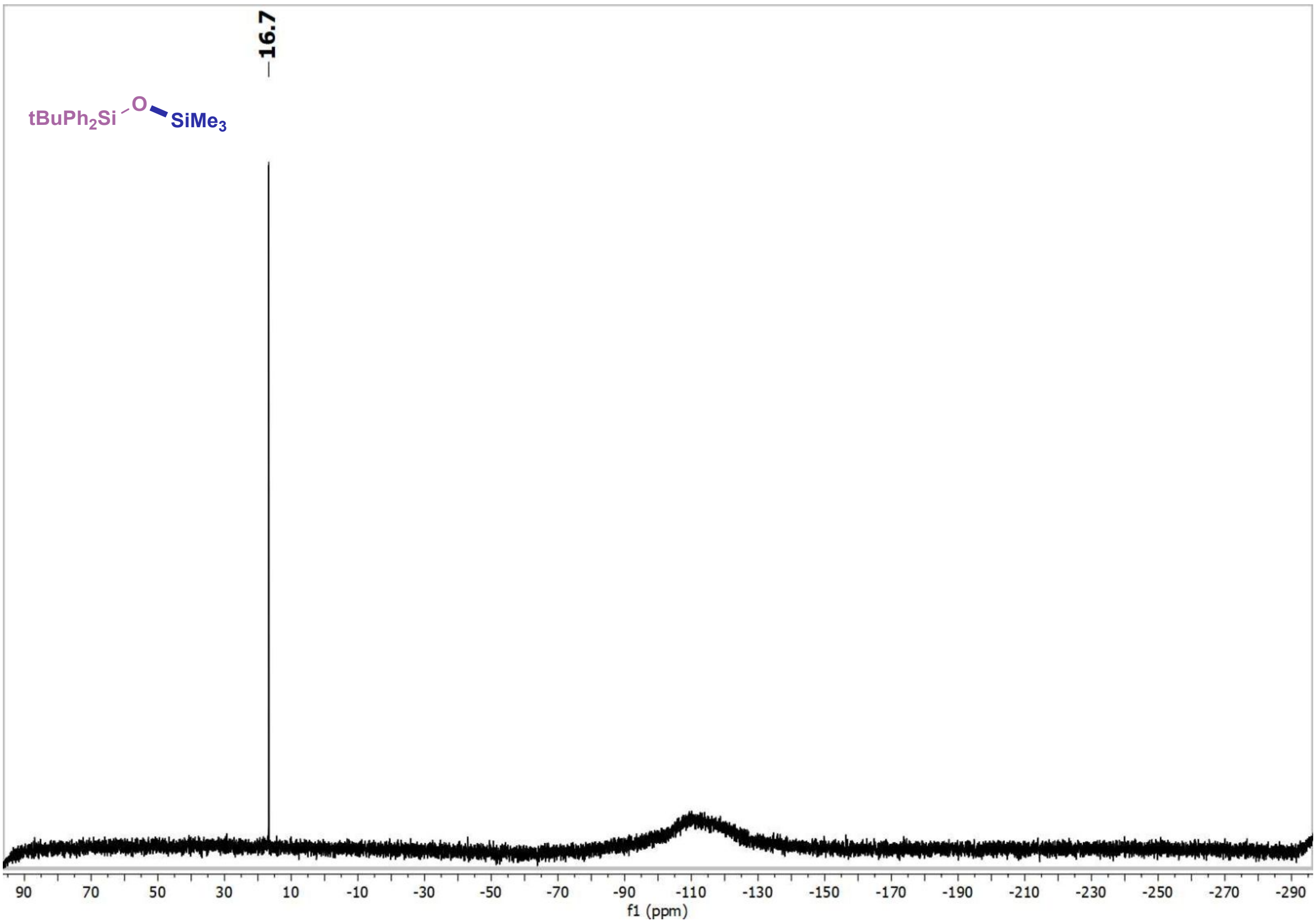


Figure S136. $^{29}Si$ NMR (79 MHz, Chloroform-d, 25°C) of 1-(*tert*-butyl)-3,3,3-trimethyl-1,1-diphenyldisiloxane (**5f**).

## (Cyclopropyl(phenyl)methoxy)trimethylsilane (**5g**)

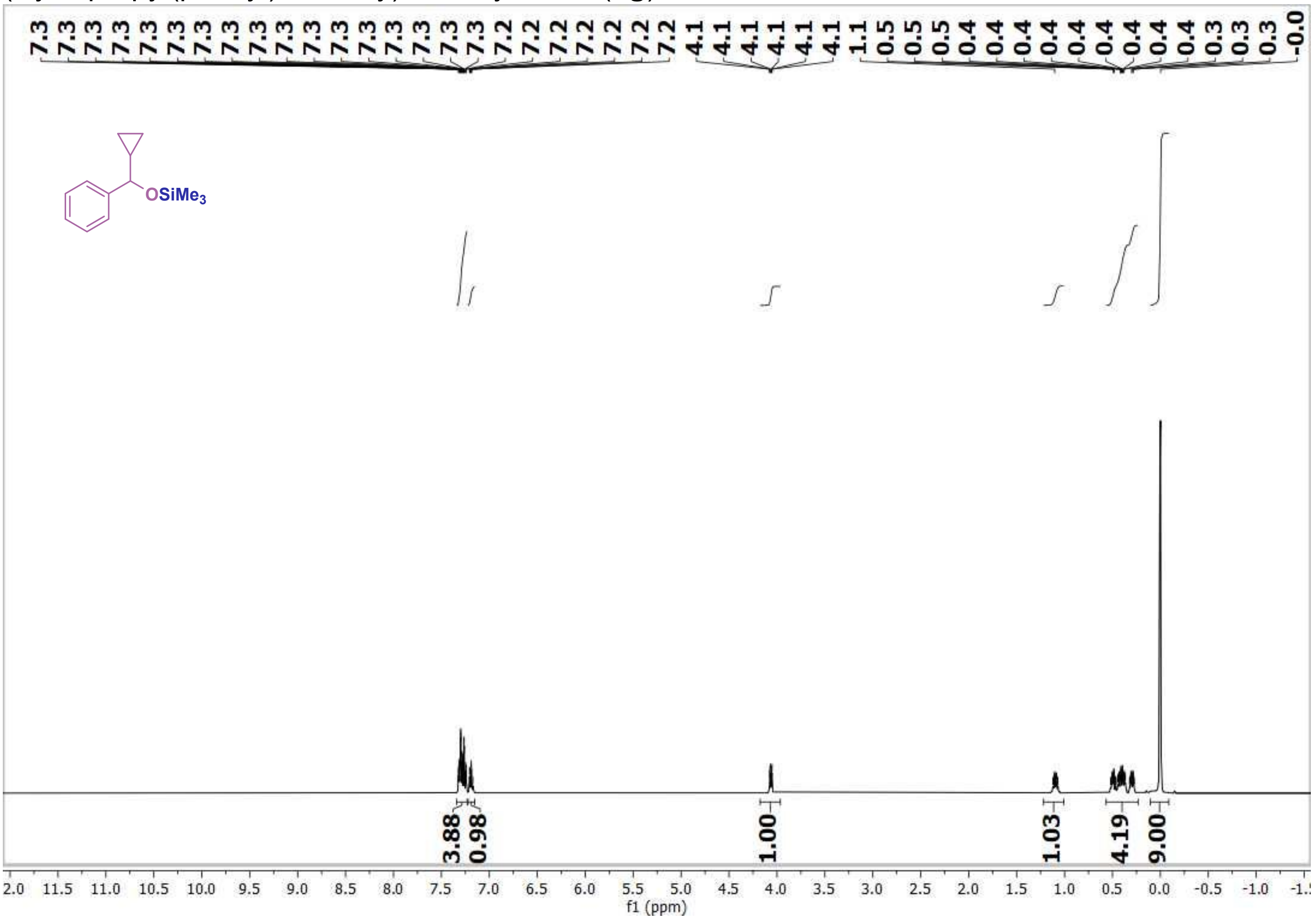


Figure S137. $^{1}$H NMR (400 MHz, Chloroform-d, 25°C) of (cyclopropyl(phenyl)methoxy)trimethylsilane (**5g**).

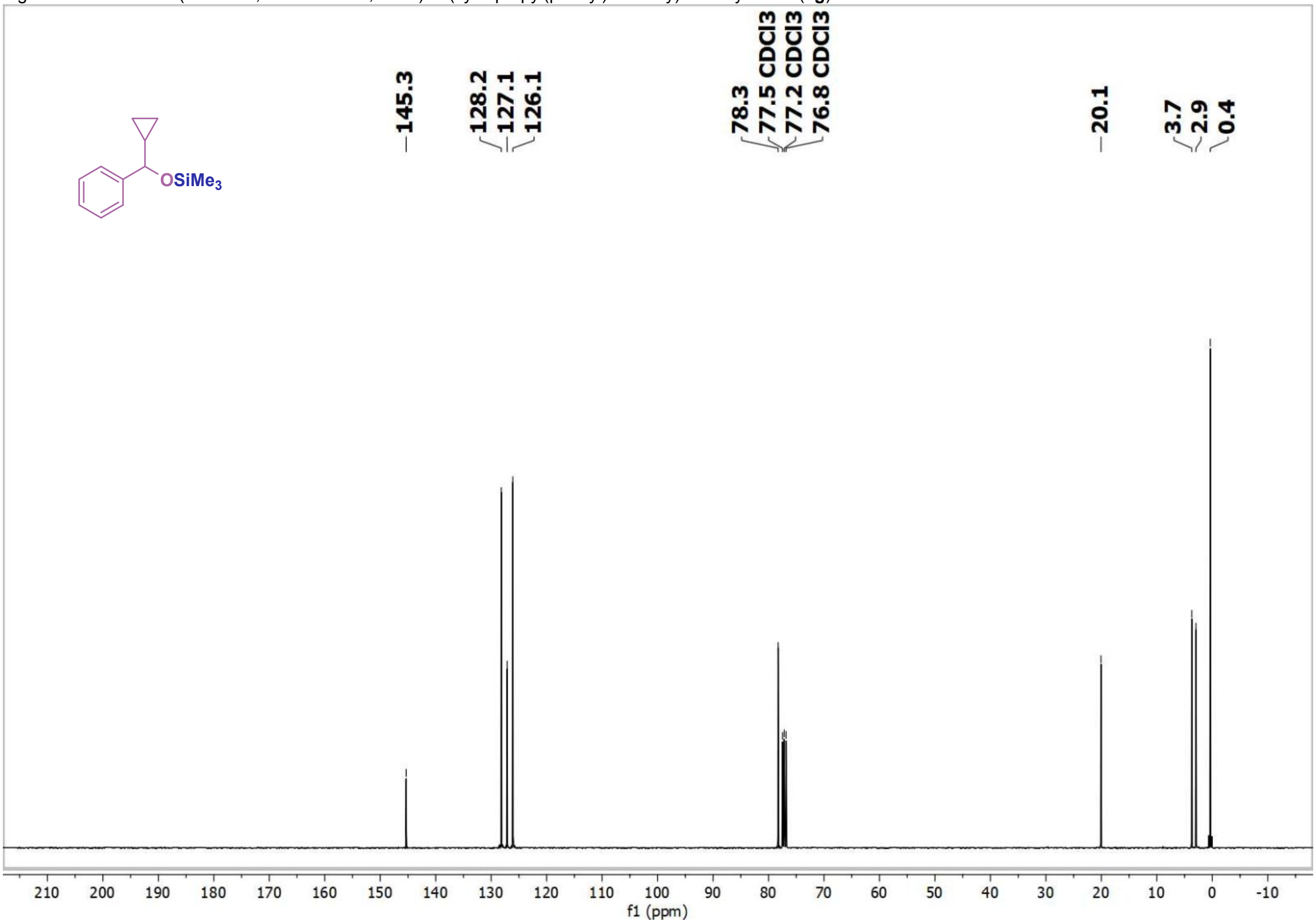


Figure S138. $^{13}$C{$^{1}$H} NMR (101 MHz, Chloroform-d, 25°C) of (cyclopropyl(phenyl)methoxy)trimethylsilane (**5g**).

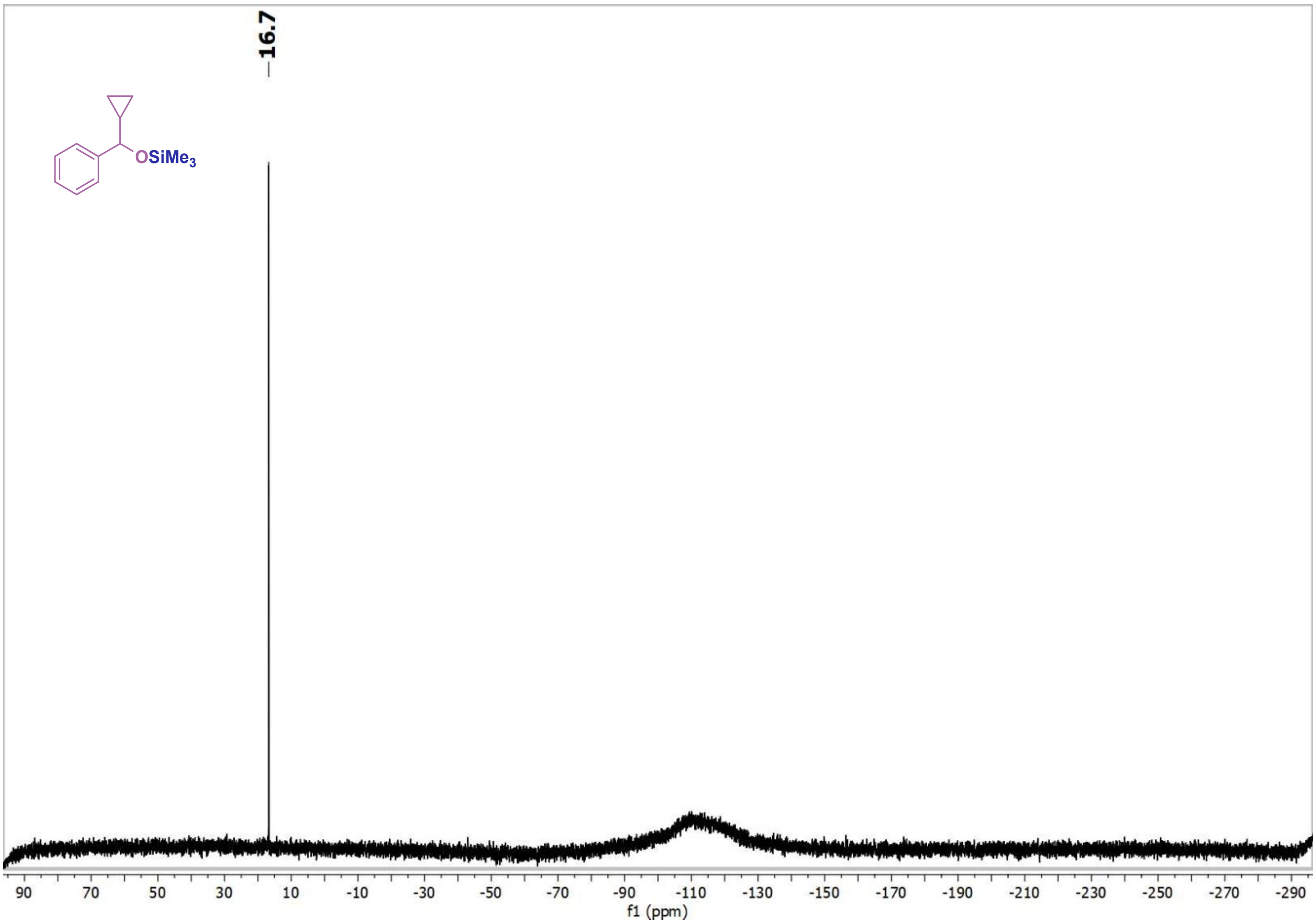


Figure S139. $^{29}$Si NMR (79 MHz, Chloroform-d, 25°C) of (cyclopropyl(phenyl)methoxy)trimethylsilane (**5g**).

# REFERENCES


[1] K. Kuciński, G. Hreczycho, *ChemCatChem* **2022**, *14*, e202200794.
[2] K. Kikuchi-Igarashi, Y. Tahara, H. Hirano, C. Ambe, H. Kinoshita, K. Miura, *Org. Lett.* **2024**, *26*, 5689–5694.
[3] P. Charki, M. Cordier, K. E. O. Ylijoki, D. S. Müller, *Chem. Eur. J.* **2025**, *31*, e202403979.
[4] C. Pfeffer, P. Probst, N. Wannenmacher, W. Frey, R. Peters, *Angew. Chem. Int. Ed.* **2022**, *61*, e202206835.
[5] H. Ruan, D. Zhu, J. Zhao, S. Zhu, *Org. Lett.* **2025**, *27*, 1741–1749.
[6] K. T. Tran, J. S. Pallesen, S. M. Ø. Solbak, D. Narayanan, A. Baig, J. Zang, A. Aguayo-Orozco, R. M. C. Carmona, A. D. Garcia, A. Bach, *J. Med. Chem.* **2019**, *62*, 8028–8052.
[7] Y. Akagi, H. Watanabe, T. Sakami, S. Furumatsu, S. Yamada, R. Maki, Y. Okuda, H. Akashi, K. Wakamatsu, Y. Kusano, A. Orita, *J. Org. Chem.* **2024**, *89*, 17122–17132.
[8] L. Gnägi, S. V. Martz, D. Meyer, R. M. Schärer, P. Renaud, *Chem. Eur. J.* **2019**, *25*, 11646–11649.
[9] K. Kuciński, H. Stachowiak, G. Hreczycho, *Eur. J. Org. Chem.* **2020**, *2020*, 4042–4049.
[10] C. Dallaire, M. A. Brook, *Organometallics* **1990**, *9*, 2873–2874.
[11] M. Rzonsowska, K. Zmudzińska, J. Duszczak, K. Mituła, B. Dudziec, P. Żak, *Organometallics* **2020**, *39*, 74–79.
[12] X. Li, F. Zhang, Y. Zhong, N. Li, J. Xu, B. Fan, *J. Org. Chem.* **2023**, *88*, 11675–11682.
[13] A. Hoppe, A. J. Stepen, L. Köring, J. Paradies, *Adv. Synth. Catal.* **2024**, *366*, 2933–2938.
[14] T. Ishikawa, M. Okano, T. Aikawa, S. Saito, *J. Org. Chem.* **2001**, *66*, 4635–4642.
[15] A. Bannykh, P. M. Pihko, *Org. Lett.* **2024**, *26*, 1991–1995.
[16] H. Yang, Y. Shen, Z. Xiao, C. Liu, K. Yuan, Y. Ding, *Chem. Commun.* **2020**, *56*, 2435–2438.
[17] I. Khan, B. G. Reed-Berendt, R. L. Melen, L. C. Morrill, *Angew. Chem. Int. Ed.* **2018**, *57*, 12356–12359.
[18] K. Kuciński, G. Hreczycho, *Inorgan. Chim. Acta* **2019**, *490*, 261–266.
[19] I. W. J. Still, W. and Daoquan, *Phosphorus, Sulfur, and Silicon Relat. Elem.* **1991**, *62*, 83–89.
[20] S. T. Kadam, S. S. Kim, *J. Organomet. Chem.* **2009**, *694*, 2562–2566.
